# Supplementary material for: Effects of activity-oriented physiotherapy with and without eye movement training on dynamic balance, functional mobility, and eye movements in patients with Parkinson’s disease: An assessor-blinded randomised controlled pilot trial
Source: PLoS One. 2024 Jun 14;19(6):e0304788. doi: 10.1371/journal.pone.0304788 (PMC11178185; doi:10.1371/journal.pone.0304788)
Supplement: S2 File — (DOCX) [file pone.0304788.s002.docx]

**S2 File. Qualitative strand: interview guide, methods, and results.**

**Methods**

**Interview Guide**

**Table S2.1. Interview guide for semi-structured interviews.**

| **Type of question or statement** | **Example phrasing of discussion leader** |
| --- | --- |
| Welcome | Good afternoon. I would like to welcome you to our focus group today. Thank you very much for agreeing to participate. |
| Introduction of the interviewer | My name is....  I am part of the research team.... |
| Describing the interview aims and procedures | Through our clinical trial, we aim to expand our understanding of eye movement training in individuals with Parkinson’s disease. |
|  | This interview is about discussing your satisfaction regarding the training programme. |
|  | There are no right or wrong answers. It is about getting an insight into your world of experience. All opinions are important for this. |
| Recording consent request | Do you agree to the interview being recorded? |
| Introductory question 1 | Please describe your previous physiotherapy experience. |
| Introductory question 2 | Could you describe any changes you have observed in your daily life that can be attributed to your physiotherapy treatment? |
| Intervention 3 | How did you like the 4-week training programme? |
| Intervention 4 | How did you feel after the training? |
| Intervention 5 | Could you detail for me what you particularly liked or disliked? |
| Intervention 6 | When did you become aware of the goals of the training programme? |
| Intervention 7 | Please describe in what ways you were able or incapable to achieve the training programme goals. |
| Intervention 8 | What physical changes have you experienced through the training programme? |
| Intervention 9 | How appropriate were the increases in difficulty of the exercises for you? |
| Intervention 10 | Please describe how easy or difficult it was for you to do all the given repetitions. |
| Intervention 11 | How fatigued did you feel physically following the training session? |
| Intervention 12 | How long did it take for you to recover after a training session? |
| Intervention 13 | What differences have you noticed in your walking balance since completing the training programme? |
| Intervention 14 | How comfortable/confident do you feel when walking at present? |
| Intervention 15 | Would you like to continue this training programme? |
| Organisation of the intervention 16 | How satisfied are you with the organisational process of the study? |
| Organisation of the intervention 17 | How satisfied are you with the organisation of the training sessions? |
| Organisation of the intervention 18 | How satisfied are you with the organisational process of the assessments? |
| Organisation of the intervention 19 | How appropriate did you find the duration of the entire intervention? |
| Outlook 20 | How appropriate did you find the duration of a single session? |
| Outlook 21 | Which specific everyday activities that are important for you could be addressed by this training programme? |
| Outlook 22 | What suggestions for improvement and wishes do you have? |
| Outlook 23 | What elements should be incorporated into the training for you to do it regularly? |
| Outlook 24 | What motor functions (e.g., strength, endurance, …) could be improved by such a training programme? |
| Outlook 25 | In your opinion, to what extent is there a need for additional research into the development of training programmes for individuals with Parkinson's disease? |
| Further questions may arise during the discussion.  Confirmation - Towards the conclusion of the interview, the interviewer confirmed that she had comprehended all statements accurately. | |
| Prompt 1 Why so?  Prompt 2 Could you describe this in more detail?  Prompt 3 Tell me more about that.  Prompt 4 Why is that important to you? | |
| Do you have any additional remarks or comments? | |

**Interview conduct**

Semi-structured interviews based on an interview guide were conducted with participants from AOPT-E [1]. The 45-minute interviews were conducted by three female physiotherapists holding Master's or PhD degrees. Interviews were tape-recorded and semantically transcribed using f4 transcription software and guidelines according to Dresing and Pehl (2). Transcriptions were checked for accuracy by a peer.

**Qualitative data analysis**

Interview data were analysed using inductive reflexive thematic analysis according to Braun & Clarke to facilitate our understanding of the data and of participants´ reality [3]. Thematic analysis (TA) organises the data in themes to answer the research question [3]. For thematic coding, we used f4 software (Dresing & Pehl GmbH, GER). A realist ontological position and social constructionism epistemology were used to generate an understanding of participants´ subjective experiences and their social production of meaning. Therefore, we chose an inductive, data-driven coding approach, to give voice to the participants [4]. Reflexive TA has six phases. The first step was to familiarise ourselves with the data set by reading it carefully and critically several times. Second, we worked systematically through the data set and identified the first codes that might be of interest or significant for the research question. These were revised several times and checked against the entire data set. In the third phase, the first themes were drafted. Themes are intended to represent clusters of codes that share a common meaning. In the fourth phase, the themes were further developed and tested. Phase five was used to refine, name, and define the themes. Finally, the themes were settled and presented in written form to readers, along with data extracts to enhance comprehensibility. This was not a linear process, but was characterised by iterative loops as well as multiple revisions, refinements, deletions, and creation of new codes and themes. During the iterative process, both the codes and the themes were regularly scrutinised through peer debriefing to consolidate the trustworthiness and credibility of our findings [5]. The developed themes and descriptions were discussed among three researchers until an agreement was found. All themes and descriptions were created from the perspective of the researchers and their backgrounds in neurology and physiotherapy.

**Results**

**Coding tree and themes**

We identified 5 unique main codes and 15 subcodes related to participants’ acceptability of the study intervention in the AOPT-E group through thematic analysis in our interview data. The coding tree is presented in Table S2.2.

**Table S2.2. Coding tree.**

| **Codes** | **Subcodes** |
| --- | --- |
| Organisation | In total, the organisation of the study is evaluated as very good |
|  | The training is perceived as varied |
|  | Appropriate adjustment of the level of difficulty |
|  | After intervention, a break is desired |
| Exercise intensity | Training is perceived as intensive |
|  | Continuation of training is desired to a more moderate extent |
|  | Daily therapy/daily training feels stressful |
| Patient education and self-management | Lack of understanding about intervention should be addressed through using patient education tools |
|  | Desire for home exercise opportunities/home exercise programmes |
|  | Subjective self-assessment is limited, therefore assessment of performance by the therapist is desirable |
| Preference in therapy | Reduction of the risk of falls and injuries and increase of gait safety are the main goals of the patients |
|  | Active training is preferred |
|  | Morning as preferred training hours |
|  | Individual 1:1 support is preferred |
|  | (Constant) therapist as motivator |

Using further synthesis, we identified five themes through thematic analysis in our data. Themes comprised topics around training organisation, progression, and variety; high training intensity; targeted patient education; walking safety, coping and motivation; and pleasure and meaning. These themes are described in detail including representative quotes in the following paragraphs.

1. All participants acknowledge the study's excellent organisation, diverse content with an appropriate level of challenge, and express a desire for a break after the intervention.
2. The participants perceive the training as intense, leading them to prefer a future continuation with a more moderate level, including rest days.
3. Participants express a preference for targeted patient education (e.g., teach-back) regarding the study's content, a home exercise programme, and feedback on their progress from the therapists.
4. Increasing walking safety is the main goal. Finding strategies to cope with the disease, the opportunity for active training, considering the person's preferred daily training time and the relationship between patient and therapist encourage motivation to participate in the intervention.
5. The intervention is enjoyable, leading to subjective improvements in activities of daily living, and is deemed meaningful by the participants.

These themes highlight the feasibility of an eye movement training for people with Parkinson’s disease. Below, we present the themes along with key example quotes.

1. **All participants acknowledge the study's excellent organisation, diverse content with an appropriate level of challenge, and express a desire for a break after the intervention.**

The organisation was highly praised, and the majority of participants found the 30-minute duration to be adequate. The 4-week timeframe was also positively received. However, some participants felt that the breaks between study sessions and the daily rehab routine were relatively short.

*"It was fantastic! The frequency didn't bother me at all. I'm quite fit now, so the frequent sessions were manageable, even when there were quick sessions before or after the regular ones. The short break between sessions for the changeover worked well for me. Although it might be more challenging for someone less physically strong, for me it was absolutely perfect ". (ID13, paragraph 10)*

The patients particularly liked the novelty of the training as compared to the therapies they were familiar with.

1. **The participants perceive the training as intense, leading them to prefer a future continuation with a more moderate level, including rest days.**

Some participants described the exercise's intensity and frequency as relatively high, while others found it to be suitable for their needs. Those who viewed it as beneficial emphasised the significance of maintaining a sufficiently high intensity:

*"* *It's a valuable addition... very intense and, therefore, helpful; the intensity was beneficial. (ID7, paragraph 15)*

*“I wouldn't say it was tough on the eyes, but overall, I found the sessions quite intensive due to the concentration required on the dots. (ID7, paragraphs 80-82)*

Nonetheless, participants expressed a desire to continue with the program but at a reduced frequency, allowing for easier integration into their daily routines:

*"Yes, I would like to continue with the training if it doesn't consume too much time." (ID20, paragraph 22).*

Given the diverse functional limitations experienced by patients with Parkinson's disease, they must exert considerable effort to exercise all affected body parts and facilitate activities:

*"* *Because life isn't solely about training. I'd probably have to train all sorts of things". (ID7, paragraph 8)*

1. **Participants express a preference for targeted patient education (e.g., teach-back) regarding the study's content, a home exercise programme, and feedback on their progress from the therapists.**

Eye tracking remains relatively unfamiliar to patients and is not extensively utilised in practical applications. Consequently, emphasising education appears crucial, and patient education tools could serve as a valuable means to achieve this priority.

*"It was interesting, although at times I wasn't fully aware of the context. I was wearing glasses, which allowed my eye movements to be visible. As for the eye movement training, I'm not certain about what you were able to observe." (ID1, paragraph 21)*

*"Well, I didn't have much clarity about the expectations for this, but I'm confident it will become clear." (ID20, paragraph 13)*

Given the limited use of eye movement training in clinical practice, the patients expressed a desire for a home exercise programme or opportunities to continue practicing.

*"I'm not sure how I could continue with these eye movement exercises on my own. Where would I find a wall with figures and pictures for the movements? I'm unsure about what else I could do... alone." (ID1, paragraph 34)*

Participants also requested that the therapist assess their performance, possibly due to a perceived lack of self-awareness or feedback from the eye tracking device.

*"You would be the most qualified person to assess whether I'm doing it correctly or if I'm making mistakes." (ID20, paragraph 29)*

1. **Increasing walking safety was the main goal. Finding strategies to cope with the disease, the opportunity for active training, considering the person's preferred daily training time and the relationship between patient and therapist encourage motivation to participate in the intervention.**

Many participants experienced a worsening in walking, postural balance, and reaction, increasing their vulnerability to falls as a consequence of Parkinson's disease. The majority identified this as their primary concern, as it significantly restricted their daily activities.

*“Yes, the intervention focuses on improving balance, which is personally important to me. Being able to maintain balance in daily life is crucial, especially for activities like grocery shopping or similar tasks." (ID14, paragraphs 127-128)*

The desired training times differed substantially between participants, partly related to medication intake. Therefore, scheduling of the training hours should be adapted to patients’ needs and preferences to achieve the best outcome.

*"Because it was difficult to match up with my intake of medication. For me, it would have been best in the morning. But we talked about it and tried to integrate it into the schedule somehow. However, I think it needs to be different for everyone.” (ID25, paragraph 46)*

The level of motivation for the intervention appeared to be heavily influenced by the therapist-patient relationship. Therapists should consistently introduce new stimuli during therapy to enhance motivation. Furthermore, if the patient and therapist don't have a good fit, it is advisable to consider changing therapists.

*"I don't believe motivation is a one-size-fits-all thing. For me personally, you've always managed to spark my curiosity about what each day holds." (ID25, paragraph 62)*

1. **The intervention is enjoyable, leading to subjective improvements in activities of daily living, and is deemed meaningful by the participants.**

The training was enjoyable for patients, particularly when new movements were introduced. Several patients reported experiencing enhancements in their daily activities, such as walking, dressing, climbing stairs, and gripping objects. These improvements were attributed to the training programme by the patients themselves.

*"Before the eye movement training, I faced difficulties putting on socks, shoes, and even outer clothing, often getting them twisted or struggling to lift my leg high enough to put on a shoe. Now, I can effortlessly slip into trousers, and my foot naturally puts on the shoe without me having to think about it. (ID13, paragraph 24)*

Through adaptive difficulty levels, the participants anticipated being able to notice their own improvements. Many participants viewed this as a motivating factor that enhances performance. In summary, most participants showed a willingness to embrace the eye tracking intervention while also emphasising the importance of integrating the training into real-life inpatient rehabilitation schedules.

In conclusion, themes comprised topics around the training organisation, variety, and challenge; training intensity, and rest; targeted patient education and performance feedback; walking safety, strategies for optimal training, and therapeutic relationship; as well as pleasure and meaning.

**References**

1. Döring N, Bortz J. Forschungsmethoden und Evaluation in den Sozial- und Humanwissenschaften. 5. ed. Berlin, Heidelberg: Springer; 2016.

2. Dresing T, Pehl T. Praxisbuch Interview, Transkription & Analyse. Anleitungen und Regelsystem für qualitativ Forschende (Vol. 8). Selfpublished. 2018.

3. Braun V, Clarke V. Using thematic analysis in psychology. Qualitative Research in Psychology. 2006;3(2):77-101. doi: 10.1191/1478088706qp063oa.

4. Braun V, Clarke V. Thematic Analysis: A Practical Guide. London, Thousand Oaks, New Delhi, Singapore: SAGE Publications Ltd; 2021.

5. Spall S. Peer Debriefing in Qualitative Research: Emerging Operational Models. Qualitative Inquiry. 1998;4(2):280-92. doi: 10.1177/107780049800400208.
