# Supplementary material for: Effects of activity-oriented physiotherapy with and without eye movement training on dynamic balance, functional mobility, and eye movements in patients with Parkinson’s disease: An assessor-blinded randomised controlled pilot trial
Source: PLoS One. 2024 Jun 14;19(6):e0304788. doi: 10.1371/journal.pone.0304788 (PMC11178185; doi:10.1371/journal.pone.0304788)
Supplement: S3 File — (DOCX) [file pone.0304788.s003.docx]

**S3 File. Eye movement training: poster description and further results.**


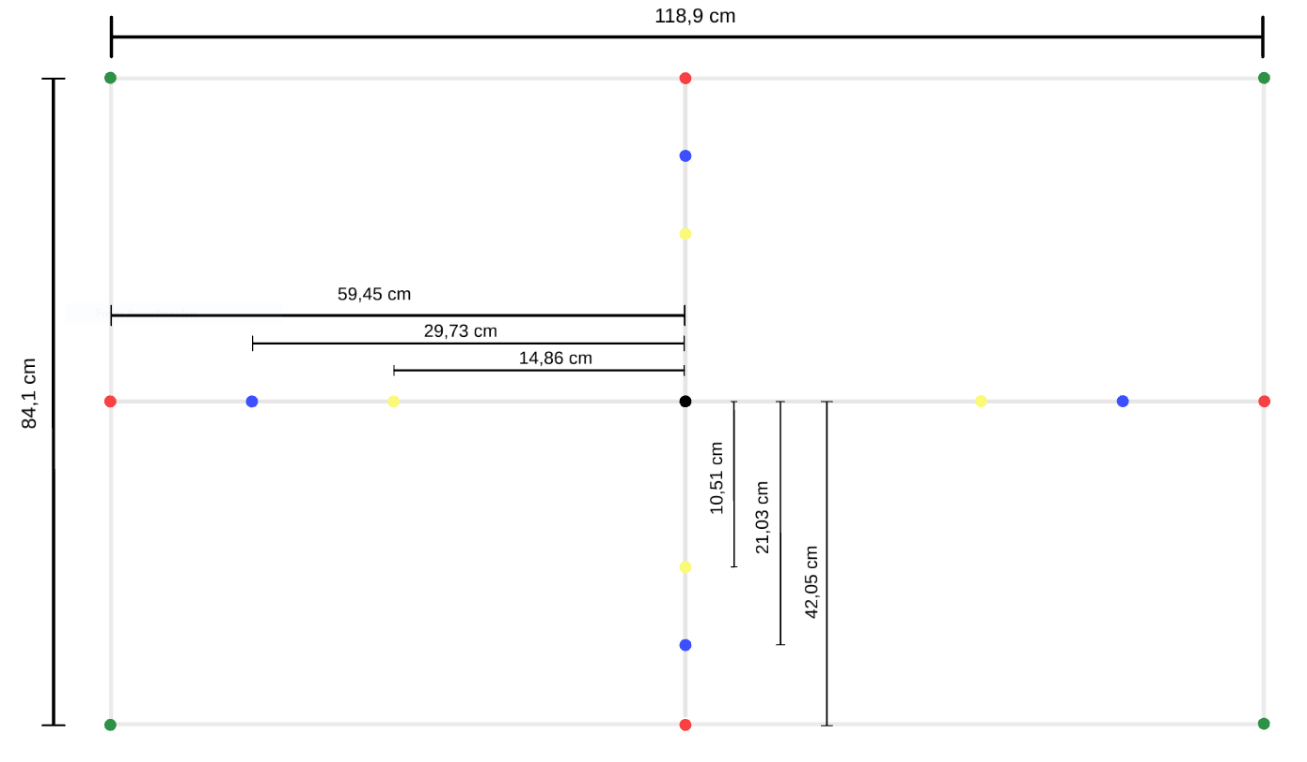


**S3.1 Fig. Eye movement training and assessment poster including dimensions.**


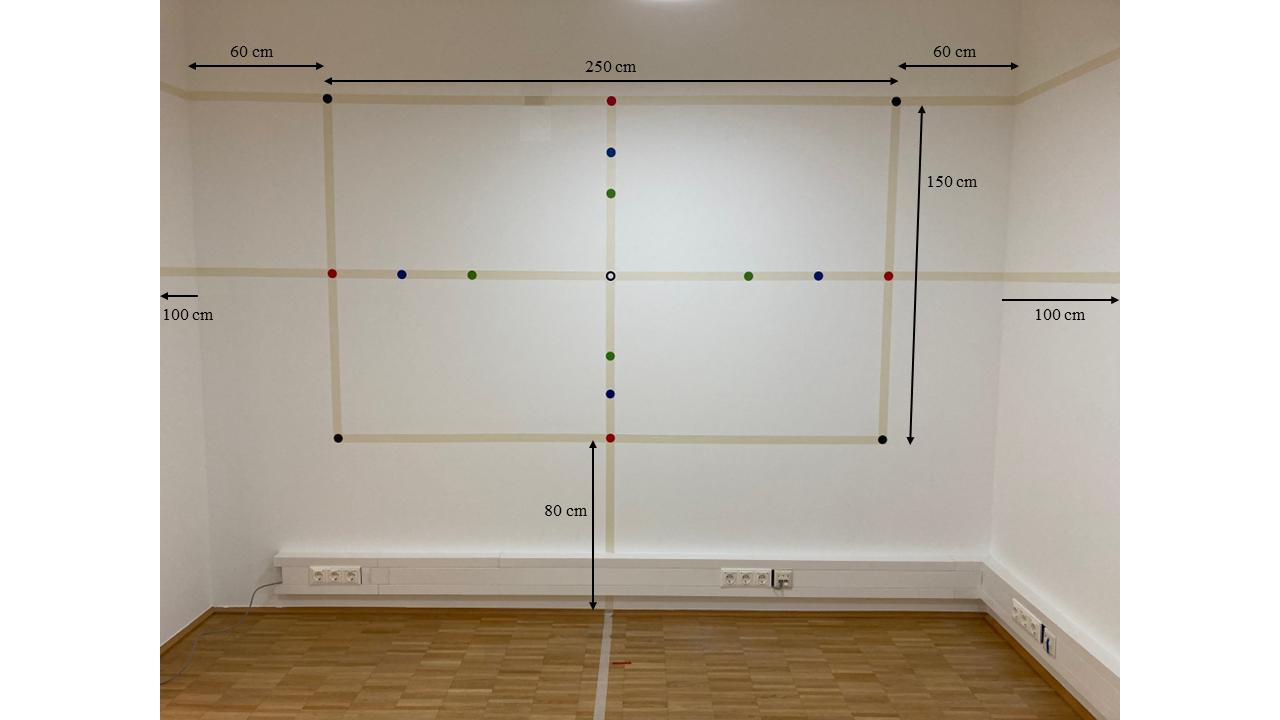


**S3.2 Fig. Eye movement training crosshair through adhesive tape and adhesive dots on the wall including dimensions.**


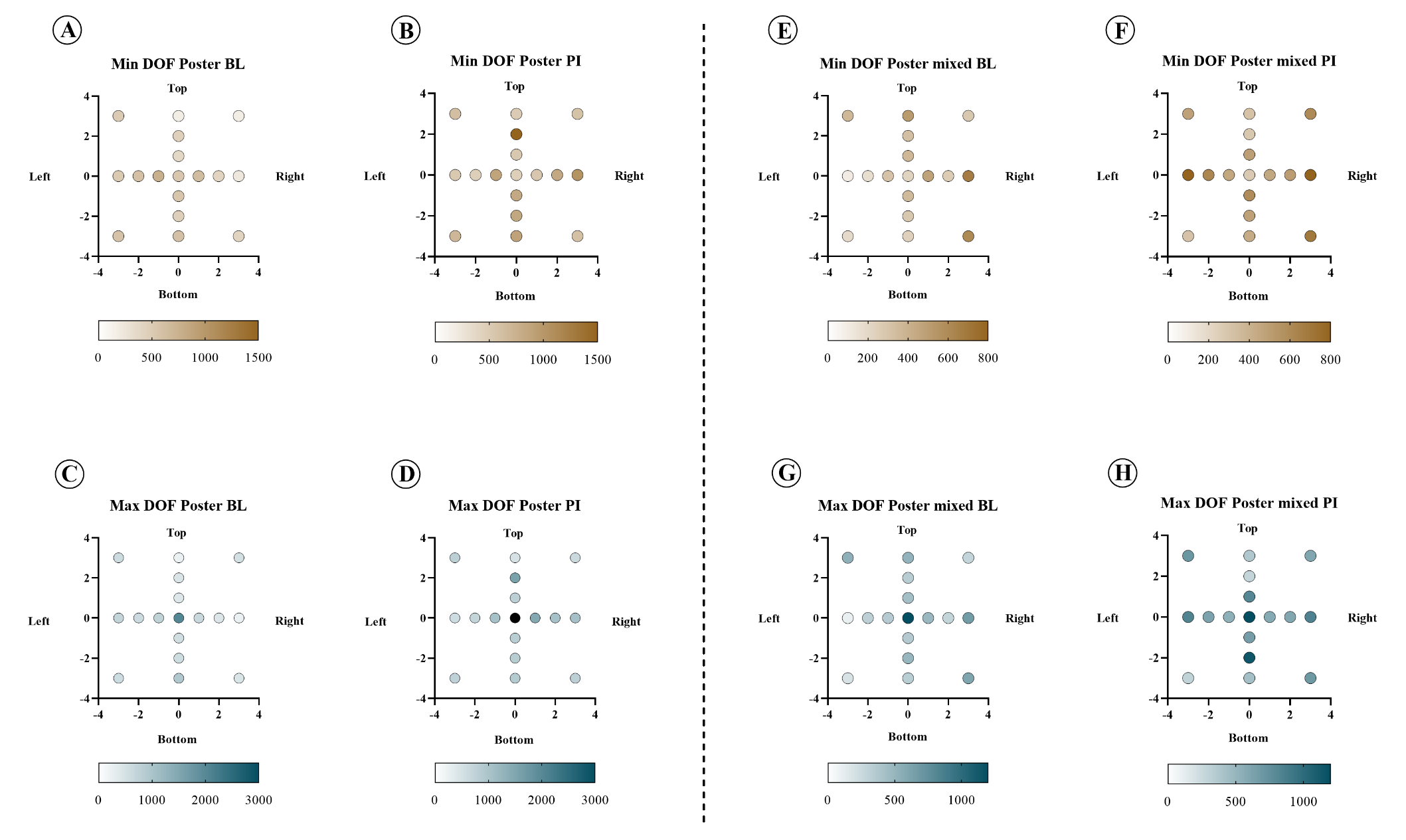


**S3.3 Fig. Effect of activity-oriented physiotherapy with eye movement training on eye movements on a wall poster.**

Bubble plots of the eye movement assessment using the wall poster in the AOPT-E group; figures A-D show results from the eye movement training at fixed sequence, figures E-H indicate results from the training at random sequence. A-B and E-F: minimum duration of fixations in milliseconds (Min DOF) at baseline and post-intervention; C-D and G-H: maximum duration of fixations in milliseconds (Max DOF) at baseline and post-intervention and number of fixations at baseline and post-intervention. Darker colours represent a longer duration of fixations. AOPT-E: activity-oriented physiotherapy plus eye movement training; BL: baseline; PI: post-intervention.
