# Supplementary material for: Effects of activity-oriented physiotherapy with and without eye movement training on dynamic balance, functional mobility, and eye movements in patients with Parkinson’s disease: An assessor-blinded randomised controlled pilot trial
Source: PLoS One. 2024 Jun 14;19(6):e0304788. doi: 10.1371/journal.pone.0304788 (PMC11178185; doi:10.1371/journal.pone.0304788)
Supplement: S1 Table — (DOCX) [file pone.0304788.s004.docx]

**S1 Table. Intervention chart.**

| **ITEM NO** | **ITEM DESCRIPTION** | |
| --- | --- | --- |
| **1 BRIEF NAME** | **Intervention group** | **Control group** |
|  | Activity-oriented physiotherapy plus eye movement training (AOPT-E) | Activity-oriented physiotherapy (AOPT) |
| **2 WHY** | Eye movement training potentially leads to an improved perception of the environment, which could positively influence dynamic balance and walking and reduce the risk of falls. | |
| **3 WHAT MATERIALS** | - Physiotherapy equipment e. g., chairs, stools, gym mats, gym balls, tennis balls, cones, ropes, step stools, dumbbells, balance boards, coordination hoops, rice, and beans bags.  - A darkened room with white light at 300 lux | |
|  | - Tobii Pro Glasses 3 (Tobii AB, Stockholm, Sweden) - Paper poster and a crosshair through adhesive tape and adhesive dots on the wall (Additional Figure 1) - Pre-recorded audio file for the standardised eye movement training | None additional |
| **4 WHAT PROCEDURES** | - Guidance and supervision from a physiotherapist (1:1) - Activity-oriented physiotherapy exercises - Different starting positions (e.g., seated, wide stance, tandem stance) for AOPT such as: - Movement exercises of the whole body, gentle stretching, range of motion exercises, and arm swinging for warm-up - Arm reaching in different positions - Activity-oriented exercises addressing coordination, balance, strength, and movement speed e.g., static balance exercises (from wide, hip-wide and narrow parallel stance, tandem stance, single leg stance, step/staggered stance, step stance with front foot on a step stool), turning exercises, stepping exercises (stepping forward, backward, to the right and left side) increasing movement speed as possible, step-ups (using a step stool), bending over to grab objects, sit-to-stand exercises from different chair height and feet positions - Exercises involved hand-eye coordination activities like tossing and catching a ball, coordination hoop, rice, or beans bags - Walking training with different variations (e.g., heel to toe, lateral walking, walking in reverse, crossing obstacles during walking, walking through a slalom course, changing walking directions and speed) - Stair climbing training - Cool down | |
|  | - Eye movement training using the wall markings combined with AOPT in seated or standing positions or moving (transfers, stepping) - Upright seated position for eye movement training in fixed or random sequence using the poster | None additional |
| **5 WHO PROVIDED** | - Trained neurological physiotherapists with at least 5 years of experience in the treatment of patients with Parkinson’s disease. - Prior to the start of the study, using a theoretical and practical information workshop including role play, the training of the therapists was delivered by two physiotherapist researchers (PhD, MSc) who initiated the study. - All trained physiotherapists followed the same instructor manual. | |
| **6 HOW** | Individually and in person | |
| **7 WHERE** | Clinic for Rehabilitation Münster, Department for Rehabilitation Science, Tirol, Austria | |
| **8 WHEN AND HOW MUCH** | - 30 minutes, 4 times per week, for 4 weeks - The intervention duration was matched across groups | |
| **9 TAILORING** | - Same for all participants - Progression of exercise difficulty according to the patients’ physical performance levels - Periods of rest if desired | |
| **10 MODIFICATIONS** | No modifications | |
| **11 HOW WELL PLANNED** | - Add-on intervention during inpatient rehabilitation (inpatient rehabilitation including medical training therapy, balance and walking training, strength and endurance training, occupational therapy, speech and language therapy, dietetics, psychological counselling, patient education; overall 2240-2600 minutes) - Scheduled number of interventions was 16 - Planned therapy during the 4-week rehabilitation stay, with daily therapy plans provided for the patients - Recording of the adherence rate in the case report form - Recording of performed exercises in Excel spreadsheet by the supervising physiotherapists. | |
| **12 HOW WELL ACTUAL** | - Supervised administration of the intervention - The adherence rate was median 16 (minimum - maximum 14 - 16) or 98% (95% confidence interval 0.95, 0.99) of the scheduled sessions. | |
