# Supplementary material for: Effects of activity-oriented physiotherapy with and without eye movement training on dynamic balance, functional mobility, and eye movements in patients with Parkinson’s disease: An assessor-blinded randomised controlled pilot trial
Source: PLoS One. 2024 Jun 14;19(6):e0304788. doi: 10.1371/journal.pone.0304788 (PMC11178185; doi:10.1371/journal.pone.0304788)
Supplement: S2 Table — (DOCX) [file pone.0304788.s005.docx]

**S2 Table. Changes in dynamic balance during walking compared between the two groups.**

| **Parameter** | **AOPT-E group, n = 12** | **AOPT group, n = 12** | **Effect size r** |
| --- | --- | --- | --- |
| Functional Gait Assessment (FGA)* | | |  |
| Baseline | 21.5 (4.0 - 28.0) | 22.0 (8.0 - 28.0) |  |
| Post-intervention | 26.0 (10.0 - 30.0) | 27.0 (10.0 - 30.0) |  |
| Change from baseline | 4.0 (1.0 to 9.0) | 2.5 (1.0 to 11.0) | 0.216 |

*Higher values indicate improvement.

AOPT: activity-oriented physiotherapy; AOPT-E: activity-oriented physiotherapy with eye movement training; N: number of participants. Values represent median (minimum - maximum).
