# Supplementary material for: Effects of activity-oriented physiotherapy with and without eye movement training on dynamic balance, functional mobility, and eye movements in patients with Parkinson’s disease: An assessor-blinded randomised controlled pilot trial
Source: PLoS One. 2024 Jun 14;19(6):e0304788. doi: 10.1371/journal.pone.0304788 (PMC11178185; doi:10.1371/journal.pone.0304788)
Supplement: S3 Table — (DOCX) [file pone.0304788.s006.docx]

**S3 Table. Changes in walking speed, mobility, balance, and fall risk in the two groups.**

| **Parameter** | **AOPT-E group, n = 12** | **AOPT group, n = 12** | **Effect size r** |
| --- | --- | --- | --- |
| 10-Metre Walk Test (10MWT)* | | | |
| Baseline | 1.50 (0.85 - 2.23) | 1.51 (0.84 - 2.36) |  |
| Post-intervention | 2.06 (0.99 - 3.13) | 1.98 (1.10 - 3.52) |  |
| Change from baseline to post-intervention | 0.30 (0.14 to 1.90) | 0.26 (0.11 to 2.44) | 0.165 |
| Timed Up and Go (TUG)** | | | |
| Baseline | 9.33 (6.78 - 21.98) | 10.53 (6.25 - 18.90) |  |
| Post-intervention | 7.47 (5.00 - 15.04) | 9.11 (5.37 - 17.32) |  |
| Change from baseline to post-intervention | -1.79 (-6.94 to -1.13) | -1.09 (-4.68 to -0.20) | 0.306 |
| Timed Up and Go with a motor task (TUGman)** | | | |
| Baseline | 10.78 (7.34 - 25.02) | 11.41 (6.98 - 21.93) |  |
| Post-intervention | 8.64 (5.62 - 20.21) | 9.67 (5.88 - 20.83) |  |
| Change from baseline to post-intervention | -1.92 (-4.81 to -1.19) | -1.17 (-3.52 to 1.82) | 0.413 |
| Berg Balance Scale (BBS)* | | | |
| Baseline | 52.50 (31.00 - 56.00) | 53.00 (34.00 - 56.00) |  |
| Post-intervention | 56.00 (46.00 - 56.00) | 56.00 (38.00 - 56.00) |  |
| Change from baseline to post-intervention | 4.00 (0.00 to 20.00) | 2.00 (-1.00 to 11.00) | 0.247 |
| Four Square Step Test (FSST)** | | | |
| Baseline | 10.12 (6.38 - 16.19) | 10.30 (6.64 - 20.00) |  |
| Post-intervention | 7.50 (4.63 - 14.23) | 9.00 (6.00 - 19.10) |  |
| Change from baseline to post-intervention | -1.77 (-3.36 to -0.35) | -0.97 (-2.28 to -0.17) | 0.427 |
| Number of non-fallers* : fallers | | | |
| Baseline | 8 : 4 | 12 : 0 |  |
| Post-intervention | 8 : 4 | 11 : 1 |  |
| Change from baseline to post-intervention | 0 : 0 | -1 : 1 |  |

*Higher values indicate improvement.
**Lower values indicate improvement.
AOPT: activity-oriented physiotherapy; AOPT-E: activity-oriented physiotherapy with eye movement training; N: number of participants. Values represent median (minimum - maximum) if not stated otherwise.
