# Supplementary material for: Effects of activity-oriented physiotherapy with and without eye movement training on dynamic balance, functional mobility, and eye movements in patients with Parkinson’s disease: An assessor-blinded randomised controlled pilot trial
Source: PLoS One. 2024 Jun 14;19(6):e0304788. doi: 10.1371/journal.pone.0304788 (PMC11178185; doi:10.1371/journal.pone.0304788)
Supplement: S4 Table — (DOCX) [file pone.0304788.s007.docx]

**S4 Table. Changes in health-related quality of life, falls-related self-efficacy, freezing of gait, and depression in the two groups.**

| **Parameter** | **AOPT-E group, n = 12** | **AOPT group, n = 12** | **Effect size r** |
| --- | --- | --- | --- |
| Parkinson’s Disease Questionnaire (PDQ-39) Mobility** | | | |
| Baseline | 10 (0.00 - 67.50) | 15.00 (0.00 - 55.00) |  |
| Post-intervention | 6.25 (0.00 - 52.50) | 10.00 (0.00 - 45.00) |  |
| Change from baseline to post-intervention | -8.75 (-37.50 to 5.00) | -5.75 (-17.50 to 5.00) | 0.166 |
| Parkinson’s Disease Questionnaire (PDQ-39) Activities of daily living** | | | |
| Baseline | 25.00 (4.17 - 58.33) | 8.33 (0.00 - 79.17) |  |
| Post-intervention | 12.50 (0.00 - 33.33) | 8.33 (0.00 - 33.33) |  |
| Change from baseline to post-intervention | -12.50 (-36,83 to 0.00) | -4.16 (-66.67 to 29.16) | 0.296 |
| Parkinson’s Disease Questionnaire (PDQ-39) Emotional functioning** | | | |
| Baseline | 22.92 (4.17 - 50.00) | 25.00 (0.00 - 58.33) |  |
| Post-intervention | 10.42 (0.00 - 33.33) | 10.42 (0.00 - 38.33) |  |
| Change from baseline to post-intervention | -10.42 (-50.00 to -4.17) | -3.75 (-37.50 to 16.66) | 0.248 |
| Parkinson’s Disease Questionnaire (PDQ-39) Stigma** | | | |
| Baseline | 12.50 (0.00 - 37.50) | 9.38 (0.00 - 56.25) |  |
| Post-intervention | 0.00 (0.00 - 12.50) | 3.13 (0.00 - 12.50) |  |
| Change from baseline to post-intervention | -9.37 (-31.25 to 0.00) | -3.12 (-50.00 to 6.25) | 0.024 |
| Parkinson’s Disease Questionnaire (PDQ-39) Social support** | | | |
| Baseline | 4.17 (0.00 - 50.00) | 4.17 (0.00 - 41.67) |  |
| Post-intervention | 0.00 (0.00 - 41.67) | 0.00 (0.00 - 41.67) |  |
| Change from baseline to post-intervention | -4.16 (-16.67 to 33.33) | 0.00 (-16.67 to 8.34) | 0.123 |
| Parkinson’s Disease Questionnaire (PDQ-39) Cognition** | | | |
| Baseline | 21.88 (6.25 - 68.75) | 25.00 (0.00 - 56.25) |  |
| Post-intervention | 12.50 (0.00 - 50.00) | 25.00 (0.00 - 56.25) |  |
| Change from baseline to post-intervention | -6.25 (-18.75 to -6.25) | 3.13 (-25.00 to 25.00) | 0.441 |
| Parkinson’s Disease Questionnaire (PDQ-39) Communication** | | | |
| Baseline | 25.00 (0.00 - 66.67) | 8.33 (33.33 - 12.50) |  |
| Post-intervention | 8.33 (0.00 - 50.00) | 0.00 (0.00 - 33.33) |  |
| Change from baseline to post-intervention | -12.49 (-20.83 to 0.00) | 0.00 (0.00 to 33.33) | 0.213 |
| Parkinson’s Disease Questionnaire (PDQ-39) Bodily discomfort** | | | |
| Baseline | 45.84 (8.33 - 66.67) | 16.67 (0.00 - 66.67) |  |
| Post-intervention | 25.00 (0.00 - 41.67) | 29.17 (0.00 - 58.33) |  |
| Change from baseline to post-intervention | -12.50 (-50.00 to 0.00) | 0.00 (-33.34 to 41.66) | 0.577 |
| Freezing of Gait Questionnaire (FOGQ)** | | | |
| Baseline | 5.50 (0.00 - 19.00) | 4.50 (0.00 - 19.00) |  |
| Post-intervention | 3.00 (0.00 - 16.00) | 3.00 (0.00 - 16.00) |  |
| Change from baseline to post-intervention | -2.50 (-6.60 to 0.00) | -1.00 (-6.00 to 3.00) | 0.248 |
| Follow-up | 2.50 (0.00 - 17.00) | 7.50 (0.00 - 18.00) |  |
| Change from baseline to follow-up | -2.00 (-7.00 to 0.00) | 1.00 (-2.00 to 6.00) | 0.650 |
| Change from post-intervention to follow-up | 0.00 (-4.00 to 1.00) | 1.50 (-2.00 to 12.00) |  |
| Falls Efficacy Scale International (FES-I)** | | | |
| Baseline | 23.50 (16.00 - 42.00) | 18.00 (16.00 - 40.00) |  |
| Post-intervention | 18.50 (14.00 - 32.00) | 18.00 (16.00 - 40.00) |  |
| Change from baseline to post-intervention | -4.00 (-16.00 to - 1.00) | -0.50 (-4.00 to 1.00) | 0.621 |
| Follow-up | 19.00 (15.00 - 34.00) | 17.50 (16.00 - 39.00) |  |
| Change from baseline to follow-up | -3.00 (-14.00 to 0.00) | -1.50 (-14.00 to 11.00) | 0.369 |
| Changes from post-intervention to follow-up | 1.00 (-3.00 to 8.00) | 0.00 (-10.00 to 13.00) |  |
| Beck Depression Inventory revised (BDI-II)** | | | |
| Baseline | 11.00 (2.00 - 22.00) | 8.00 (1.00 - 13.00) |  |
| Post-intervention | 5.00 (0.00 - 18.00) | 6.00 (1.00 - 13.00) |  |
| Change from baseline to post-intervention | -4.50 (-9.00 to - 1.00) | -2.00 (-9.00 to 2.00) | 0.403 |

*Higher values indicate improvement.
**Lower values indicate improvement.
AOPT: activity-oriented physiotherapy; AOPT-E: activity-oriented physiotherapy with eye movement training; N: number of participants. Values represent median (minimum - maximum) if not stated otherwise.
