# Supplementary material for: Effects of activity-oriented physiotherapy with and without eye movement training on dynamic balance, functional mobility, and eye movements in patients with Parkinson’s disease: An assessor-blinded randomised controlled pilot trial
Source: PLoS One. 2024 Jun 14;19(6):e0304788. doi: 10.1371/journal.pone.0304788 (PMC11178185; doi:10.1371/journal.pone.0304788)
Supplement: S1 Data — (PDF) [file pone.0304788.s008.pdf]

PRÜFPLAN (STUDIENPROTOKOLL)

Version: 1.1, vom 27.03.2021

Ersetzt Version: 1.0

Amendment Nr.:-

Titel der Studie: Effekte eines aktivitätsorientierten physiotherapeutischen Trainingsprogramms mit und ohne Blickbewegungstraining auf die dynamische Balance und das Sturzrisiko bei Personen mit Idiopathischem Parkinsonsyndrom: Eine randomisierte kontrollierte Pilotstudie

Study title Effects of an **a**ctivity-oriented **p**hysiotherapy exercise programme with and without **e**ye **m**ovement training on dynamic balance and fall risk in people with **P**arkinson's **d**isease: a randomized controlled **pilot** trial

Kurztitel: **APEM-PD-Pilot**

Studiendesign: Prospektive doppelblinde randomisierte kontrollierte Pilotstudie

Sponsor: [redacted]

DRKS-ID.: Sobald die Freigabe durch die Ethikkommission erfolgt ist, wird die Registrierung im DRKS oder einem anderen Primären Register gemäß der WHO Kriterien beantragt

Antragstellerin: [redacted], Reha Zentrum Münster, [redacted]

[redacted]

Dokumentenhistorie

Version	Datum	Amendment Nr.	Autor(en)	Änderungen/ Grund
1.0	02.02.2021	-	[redacted]	Erstversion
1.1	27.03.2021	-	[redacted]	Zweitversion

Beteiligte Personen/Institutionen

Funktion	Name & Kontakt
Sponsor	[redacted]
Koordinierender Prüfer	[redacted]
Prüferin	[redacted]
Biometrie	[redacted]

Teilnehmendes Studienzentrum

**A 01**	**Reha Zentrum Münster,** [redacted]

Unterschriftenseite Sponsor

Studientitel:	Effekte eines aktivitätsorientierten physiotherapeutischen Trainingsprogramms mit und ohne Blickbewegungstraining auf die dynamische Balance und das Sturzrisiko bei Personen mit Idiopathischem Parkinsonsyndrom: Eine randomisierte kontrollierte Pilotstudie
Kurztitel	APEM-PD-Pilot

Der vorliegende Prüfplan wurde einer kritischen Überprüfung unterzogen. Der Inhalt ist in Übereinstimmung mit der derzeitigen Nutzen-Risiko-Einschätzung für die beschriebenen Methoden sowie mit den moralischen, ethischen und wissenschaftlichen Prinzipien der letztgültigen Version der Deklaration von Helsinki und den lokalen Gesetzen und Verordnungen.

[redacted]	
**Sponsor**	**Ort, Datum, Unterschrift**

Der oben Unterzeichnende bestätigt, dass er den vorliegenden Prüfplan gelesen hat und bestätigt, dass der Prüfplan alle notwendigen Informationen enthält, die für die Studiendurchführung notwendig sind. Der Unterzeichnende bestätigt darüber hinaus, dass die Studie gemäß dem vorliegenden Prüfplan durchgeführt wird. Es wird vereinbart, dass jede bis dato nicht veröffentlichte Information der strengsten Geheimhaltung obliegt.

Unterschriftenseite Koordinierender Prüfer und Prüferin

Studientitel:	Effekte eines aktivitätsorientierten physiotherapeutischen Trainingsprogramms mit und ohne Blickbewegungstraining auf die dynamische Balance und das Sturzrisiko bei Personen mit Idiopathischem Parkinsonsyndrom: Eine randomisierte kontrollierte Pilotstudie
Kurztitel	APEM-PD-Pilot

Der vorliegende Prüfplan wurde einer kritischen Überprüfung unterzogen. Der Inhalt ist in Übereinstimmung mit der derzeitigen Nutzen-Risiko-Einschätzung für die beschriebenen Methoden sowie mit den moralischen, ethischen und wissenschaftlichen Prinzipien der letztgültigen Version der Deklaration von Helsinki und den lokalen Gesetzen und Verordnungen.

[redacted]<br><br>**Koordinierender Prüfer**	______________________________<br>**Ort, Datum, Unterschrift**
[redacted]<br><br>**Prüferin**	______________________________<br>**Ort, Datum, Unterschrift**

Der oben Unterzeichnende bestätigt, dass er den vorliegenden Prüfplan gelesen hat und bestätigt, dass der Prüfplan alle notwendigen Informationen enthält, die für die Studiendurchführung notwendig sind. Der Unterzeichnende bestätigt darüber hinaus, dass die Studie gemäß dem vorliegenden Prüfplan durchgeführt wird. Es wird vereinbart, dass jede bis dato nicht veröffentlichte Information der strengsten Geheimhaltung obliegt.

Unterschriftenseite Biometrikerin

Studientitel:	Effekte eines aktivitätsorientierten physiotherapeutischen Trainingsprogramms mit und ohne Blickbewegungstraining auf die dynamische Balance und das Sturzrisiko bei Personen mit Idiopathischem Parkinsonsyndrom: Eine randomisierte kontrollierte Pilotstudie
Kurztitel	APEM-PD-Pilot

Der vorliegende Prüfplan wurde einer kritischen Überprüfung unterzogen. Der Inhalt ist in Übereinstimmung mit der derzeitigen Nutzen-Risiko-Einschätzung für die beschriebenen Methoden sowie mit den moralischen, ethischen und wissenschaftlichen Prinzipien der letztgültigen Version der Deklaration von Helsinki und den lokalen Gesetzen und Verordnungen.

[redacted]	
**Biometrikerin**	**Ort, Datum, Unterschrift**

Die oben Unterzeichnende bestätigt, dass sie den vorliegenden Prüfplan gelesen hat und bestätigt, dass der Prüfplan alle notwendigen Informationen enthält, die für die Studiendurchführung notwendig sind. Die Unterzeichnende bestätigt darüber hinaus, dass die Studie gemäß dem vorliegenden Prüfplan durchgeführt wird. Es wird vereinbart, dass jede bis dato nicht veröffentlichte Information der strengsten Geheimhaltung obliegt.

Funding

Diese Studie ist eine akademische Eigenstudie, die keine externe finanzielle Unterstützung erhält.

Inhaltsverzeichnis

Teilnehmendes Studienzentrum 3
Unterschriftenseite Sponsor 4
Unterschriftenseite Koordinierender Prüfer und Prüferin 5
Unterschriftenseite Biometrikerin 6
1. Einleitung 20
1.1 Hintergrund der Studie 20
1.2 Notwendigkeit der Durchführung einer Studie 21
1.3 Risiko- Nutzen Abschätzung 22
2 Studienziele und Hypothesen 23
2.1 Studienzweck 23
2.2 Forschungsfrage 24
2.3 Studienziele 24
2.4 Nullhypothesen 24
2.5 Alternativhypothesen 25
3 Studienbeschreibung 25
3.1 Studiendesign 25
3.2 Studienzentrum 25
3.3 Zuständige Ethikkommission 25
3.4 Registrierung der Studie 26
3.5 Zeitplan 26
4 Methoden 28
4.1 Studienteilnehmer*innen 28
4.1.1 Teilnehmer*innenrekrutierung und Studieneinschluss 28
4.1.2 Studienpopulation 28
4.1.3 Randomisierung und verdeckte Zuteilung 29
4.1.4 Verblindung 30
4.1.5 Entblindung 30
4.2 Intervention 30
4.2.1 Behandlungsschema 30
4.2.2 Intervention 31
4.3 Endpunkte und Datenerhebung 35
4.3.1 Demografische und krankheitsspezifische Daten 35
4.3.2 Datenerhebung und Untersuchende 35
4.3.3 Primäre und sekundäre Endpunkte und Erhebungsinstrumente 35
4.4 Durchführung und psychometrische Kriterien klinischer Assessments 39

4.4.1 Functional Gait Assessment 39
4.4.2 Messung der Augenbewegungen während Items 5, 6 und 10 des FGA 39
4.4.3 Mini-Mental-Status-Test (MMST) 40
4.4.4 Timed-Up-and-Go (TUG) 40
4.4.5 Timed-Up-and-Go with manual dual task (TUGman) 41
4.4.6 Berg Balance Scale (BBS) 42
4.4.7 Four Step Square Test (FSST) 43
4.4.8 10-Meter Gehtest 43
4.4.9 Parkinson's Disease Questionnaire (PDQ-39) 44
4.4.10 Freezing of Gait Questionnaire (FOG-Q) 48
4.4.11 Falls Efficacy Scale- International Version (FES-I) 49
4.4.12 Beck Depression Inventory (BDI-II) 49
4.4.13 Sturzrate 50
4.4.14 Machbarkeit einer größeren Studie 50
4.4.15 Messung der Augenbewegungen während der Intervention 52
4.5 Unerwünschte Ereignisse, Nebenwirkungen 52
4.5.1 Definitionen 52
4.5.2 Beurteilung und Dokumentation 54
4.5.3 Meldung von schwerwiegenden Vorkommnissen (SUE/SNW) 55
5 Datendokumentation, -Management und -Auswertung 56
5.1 Methoden der Datensammlung 56
5.2 Dokumentation 56
5.2.1 Quelldaten und -dokumente 56
5.2.2 Erhebungsbogen (CRF) 56
5.3 Weiterbehandlung der Patient*innen nach Abschluss der Prüfung 57
5.4 Ausscheiden von Patient*innen (Drop-out) 57
5.5 Vorzeitiger Abbruch der klinischen Prüfung 58
5.6 Datenanalyse 58
5.6.1 Statistische Datenanalyse 58
5.6.2 Qualitative Datenanalyse 60
5.7 Prüfordner und Datenmanagement 61
5.7.1 Aufbewahrung d. Studienunterlagen, Datenspeicherung & -löschung 61
5.7.2 Datenmanagement 61
5.7.3 Umgang mit Rückfragen 62
6 Ethische, rechtliche und administrative Aspekte 63
6.1 Einhaltung ethischer und regulatorischer Anforderungen 63
6.2 Votum der Ethikkommission 63

6.3 Patient*inneninformation und Einwilligungserklärung 63
6.4 Patient*innenversicherung 64
6.5 Datenschutz und Schweigepflicht 64
7 Änderungen während der Studiendurchführung 66
7.1 Änderungen des Prüfplans (Amendments) 66
7.2 Abweichungen vom Prüfplan 66
7.3 Information der Studienteilnehmer über die Ergebnisse der Studie 66
8 Funding, Interessenskonflikte und Entschädigung der Teilnehmer 67
8.1 Funding der Studie 67
8.2 Mögliche Interessenkonflikte 67
8.3 Honorar und Entschädigung der Teilnehmer*innen 67
9 Literaturverzeichnis 67

Studiensynopse

**Koordinierender Prüfer**	[redacted]
**Sponsor**	[redacted]
**Titel**	Effekte eines aktivitätsorientierten physiotherapeutischen Trainingsprogramms mit und ohne Blickbewegungstraining auf die dynamische Balance und das Sturzrisiko bei Personen mit Idiopathischem Parkinsonsyndrom: Eine randomisierte kontrollierte Pilotstudie
**Kurztitel**	APEM-PD-Pilot
**DRKS-ID**	Sobald die Freigabe durch die Ethikkommission erfolgt ist, wird die Registrierung im DRKS oder einem anderen Primären Register gemäß der WHO Kriterien beantragt
**Studienziel**	Studienziel ist es, die Effekte eines aktivitätsorientierten physiotherapeutischen Trainingsprogramms mit einem Blickbewegungstraining im Vergleich zu einem aktivitätsorientierten physiotherapeutischen Trainingsprogramm ohne Blickbewegungstraining auf die dynamische Balance, Mobilität mit und ohne Multi-Task, statisches und dynamisches Gleichgewicht Gehgeschwindigkeit, gesundheitsbezogene Lebensqualität, Freezing of Gait, sturzassoziierte Selbstwirksamkeit, Depression und Sturzrate zu untersuchen. Ziel ist außerdem, die Veränderung der Augenbewegungen (Sakkadengeschwindigkeit, -Amplitude, -Latenzzeit, Fixationsdauer) über den Interventionszeitraum hinweg zu explorieren und die Machbarkeit einer größeren randomisierten kontrollierten Studie anhand vordefinierter Parameter zu evaluieren.
**Primäre Endpunkte**	1.1 Dynamische Balance, gemessen anhand des Functional Gait Assessment (FGA)

	1.2 Items 5, 6 und 10 des FGA werden zusätzlich mit einer Eye-Tracking Brille (Tobii Pro Glasses 3) durchgeführt, zur mobilen Messung der Augenbewegungen (Sakkadengeschwindigkeit, -Amplitude, -Latenzzeit, Fixationsdauer)
**Sekundäre Endpunkte**	2.0 Screening für kognitive Funktion bzw. Beeinträchtigung: Mini-Mental State Test (MMST)<br>2.1 Funktionelle Mobilität, gemessen mit dem Timed-Up-and-Go (TUG)<br>2.2 Funktionelle Mobilität mit Dual-Task, gemessen mit dem Timed-Up-and-Go mit manuellem Dual Task (TUGman)<br>2.3 Statisches und dynamisches Gleichgewicht, gemessen anhand der Berg Balance Scale (BBS)<br>2.4 Dynamisches Gleichgewicht, gemessen anhand des Four Square Step Test (FSST)<br>2.5 Gehgeschwindigkeit, gemessen mit dem 10-Metre Walk Test (10MWT)<br>2.6 Gesundheitsbezogene Lebensqualität, gemessen mit dem Parkinson's Disease Questionnaire-39 (PDQ-39)<br>2.7 Freezing of Gait, gemessen mit dem Freezing of Gait Questionnaire (FOGQ)<br>2.8 Sturzassoziierte Selbstwirksamkeit, gemessen mit der Falls Efficacy Scale- Internationale Version (FES-I)<br>2.9 Depression, gemessen anhand des Beck Depression Inventory (BDI-II)<br>2.10 Sturzrate 3 Monate vor Studieneinschluss, während der 4-wöchigen Interventionsdauer und während der 4-wöchigen Follow-up Periode<br>2.11 Mobile Erfassung der Augenbewegungen (Sakkaden-geschwindigkeit, -Amplitude, -Latenzzeit, Fixationsdauer) während der Intervention (1x wöchentlich, jeweils Training am Ende der Woche, für je 12 min, während Übung 2, 3, 4, 6 und 7)<br>2.12 Machbarkeit einer größeren Studie (Rekrutierungs-, Retentions-, Adhärenzrate, Unerwünschte Ereignisse, Akzeptanz

	der Intervention in der Interventionsgruppe (gemessen mittels Fokusgruppen in Interventionsgruppe 1 zu Postintervention)
**Studiendesign**	Prospektive doppelblinde randomisierte kontrollierte Pilotstudie
**Indikation**	Erwachsene mit Idiopathischem Parkinson Syndrom und leichter bis mäßiger Einschränkung (Hoehn & Yahr Stadium 1-3)
**Fallzahl**	34 Personen mit Idiopathischem Parkinsonsyndrom
**Intervention**	Die Intervention findet im Rahmen der stationären Rehabilitation der Patient*innen statt und wird zusätzlich zur üblichen Reha durchgeführt.<br><br>*Interventionsgruppe (Gruppe 1):* Aktivitätsorientiertes physiotherapeutisches Trainingsprogramm mit einem Blickbewegungstraining; 30 min, 4x pro Woche supervidiert durch geschulte Physiotherapeut*innen. Insgesamt 4 Einheiten á 30 Min. pro Woche für 4 Wochen<br><br>*Kontrollgruppe (Gruppe 2):* Physiotherapeutische Standardversorgung: aktivitätsorientiertes, physiotherapeutisches Trainingsprogramm ohne Blickbewegungstraining; 30 min, 4x pro Woche supervidiert, durch geschulte Physiotherapeut*innen. Insgesamt 4 Einheiten á 30 Min. pro Woche für 4 Wochen
**Studienzentrum**	Reha Zentrum Münster [redacted]
**Einschlusskriterien**	Männer und Frauen mit Idiopathischem Parkinsonsyndrom anhand der UK Brain Bank Kriterien (Hughes et al., 1992), Stadium 1-3 nach Hoehn & Yahr) bei ON Medikation (Hoehn and Yahr, 1967), Alter 30-80 Jahre, jeglicher Ethnizität, Gehfähigkeit ohne Hilfsperson, Mini-Mental-Status-Test (MMST) ≥ 24/30 (Tombaugh and McIntyre, 1992), stabile Dosierung dopaminerger Ersatztherapie mind. 3 Wochen vor Studienbeginn oder noch

	ohne dopaminerge Behandlung für die Dauer der Studienintervention, deutschsprachig in Wort und Schrift.
**Ausschlusskriterien**	Konkomitante Erkrankung(en) (wie maligne Erkrankungen, andere neurologische, orthopädische, kardiale oder psychiatrische Erkrankungen, Major Depression, Demenz), Photosensitivität, nicht-parkinsonistische Gangstörung (z.B. aufgrund muskuloskelettaler Symptome), rezenter chirurgischer Eingriff (allgemein und an den Augen), intraoculäre Implantate, Strabismus, Nystagmus, ausgeprägte Schlupflider, unbehandelte Schmerzen, unkorrigierte Visus- oder Hörbeeinträchtigung, die das Training oder die Untersuchungen beeinträchtigen würden, Schwangerschaft, rezente Tiefe Hirnstimulation (THS) oder Änderung der Parameter der THS innerhalb des letzten Jahres, schwere motorische Fluktuationen, Initiierung einer neuen dopaminergen Medikation oder Anpassung derselben innerhalb des Studienzeitraums ist nicht vorgesehen; im Sinn eines Real-Life Settings obliegt diese Entscheidung dem Prüfarzt oder der Prüfärztin.<br><br>Zur Korrektur von Fehlsichtigkeit werden zum mobilen Tobii Eye Tracker passende Korrekturlinsen eingesetzt.
**Statistische Analyse**	Die statistische Datenanalyse erfolgt mit IBM SPSS Software, Version 26.0 (IBM Corporation, Armonk, NY, USA) und Tobii Pro Lab Analyer (Tobii, Danderyd, Sweden). Das statistische Signifikanzniveau ist durch einen p-Wert von <0,05 definiert. Es wird versucht, fehlende Daten zu vermeiden, indem die Fragebögen nach dem Ausfüllen inspiziert und im Falle von unbeantworteten Items die Studienteilnehmer*nnen gebeten werden, diese zu komplettieren. Die Anzahl fehlender Daten wird notiert; aufgrund des wenig sensitiven Untersuchungsthemas wird angenommen, dass fehlende Variablenwerte „Missing completely at random“ bzw. „Missing at random“ zugeordnet werden können. Eine Intention-to-treat

	Analyse wird für alle Fälle durchgeführt, die in der ihnen ursprünglich zugewiesenen Gruppe analysiert werden.<br><br>Deskriptive Statistik wird für die demografischen Daten sowie primären und sekundären Outcomes verwendet. Gezählte und nominalskalierte Daten (Geschlecht, Sturzrate, Rekrutierungs-, Retentions- und Adhärenzrate, ggf. Anzahl fehlende Daten, Anzahl unerwünschte Ereignisse) werden als ganze Zahlen bzw. Prozentsätze angegeben, ordinalskalierte Variablen (MMSE, UPDRS, H & Y, FGA, BBS, PDQ-39, FOGQ, FES-I, BDI-II) mittels des Medians (Minimum, Maximum bzw. Interquartilsabstand) sowie metrische Variablen (TUG, TUGman, FSST, 10MWT, Augenbewegungen: Sakkadengeschwindigkeit, -Amplitude, -Latenzzeit, Fixationsdauer) durch Mittelwert (95% Konfidenzintervall, CI bzw. Standardabweichung, SD). Die Darstellung der Daten erfolgt auch grafisch (z.B. durch Balkendiagramme, Box-Plot oder Liniendiagramme).<br><br>Die Eignungsrate (%) ist der Prozentsatz der für die Studie geeigneten PatientInnen nach Anwendung der Ein- und Ausschlusskriterien aus der im Studienzeitraum am Reha Zentrum Münster behandelten Parkinsonpatient*innen.<br><br>Die Rekrutierungsrate (%) wird folgendermaßen bestimmt: $(N_{Consent}/N_{geeignet})$*100; dabei ist $N_{consent}$ die Anzahl der TeilnehmerInnen, welche die informierte Einwilligungserklärung unterschrieben haben; $N_{geeignet}$ ist die Anzahl der anhand der für die Studie unter Anwendung der Ein- und Ausschlusskriterien geeigneten Patient*nnen.<br><br>Die Retentionsrate (%) wird folgendermaßen berechnet: $(N_{komplettiert}/N_{gesamt})$*100; dabei ist $N_{komplettiert}$ die Anzahl der Teilnehmer*nnen, welche die Studie abgeschlossen haben; N_{gesamt} ist die Gesamtanzahl der StudienteilnehmerInnen.<br><br>Die Adhärenzrate (%) wird wie folgt bestimmt: (Tatsächlich durchgeführte Anzahl an Übungssitzungen/geplante Anzahl an

	Trainingssitzungen)*100 (Osterberg & Blaschke, 2005).<br><br>Die Eignungsrate, Rekrutierungs-, Retentions- und Adhärenzraten werden anhand der von Newcombe propagierten Wilson ‚Score'-Methode samt ihres 95%-igen CI berechnet (Newcombe, 1998); im Fall einer Proportion nahe 0 oder 1 wird eine Poisson Approximation nach Brown verwendet (Brown, Cai, & DasGupta, 2001).<br><br>Es erfolgt eine Überprüfung statistisch signifikanter Unterschiede zwischen den Gruppen zu Baseline: für nominale Daten (Eignungs-, Rekrutierungs-, Retentions-, Adhärenzrate, Geschlecht) wird der Fisher's Exact Test verwendet, für ordinale Daten (Alter, H & Y, UPDRS, FGA, MMST, BBS, PDQ-39, FOGQ, FES-I, BDI-II) der Mann Whitney-U Test und für metrische Daten (TUG, TUGman, FSST, 10MWT, Augenbewegungen: Sakkadengeschwindigkeit, -Amplitude, -Latenzzeit, Fixationsdauer) der T-Test für unabhängige Stichproben. Eine präliminäre inferentielle statistische Analyse zur Detektion von Trends hinsichtlich der Effektivität der Intervention zu erkennen und als Basis für eine Stichprobenberechnung für eine randomisierte kontrollierte Studie mit ausreichender Power von mindestens 80% (anhand der Mittelwertunterschiede (SD) zwischen den Gruppen) wird durchgeführt. Für die inferentielle Statistik werden zunächst metrische Daten mittels Shapiro-Wilk Test, Q-Q Plots und Histogrammen auf Normalverteilung sowie auf signifikante Datenausreißer getestet. Nicht normalverteilte Daten werden mittels geeigneter Transformation (z.B. ln, Quadratwurzel) transformiert, erneut überprüft und bei fehlender Normalverteilung nicht-parametrisch analysiert.<br><br>Unterschiede zwischen den beiden Gruppen und Testzeitpunkten werden für ordinale Daten folgendermaßen berechnet: Aus der Differenz der Postintervention- und Baselinedaten werden neue Variablen generiert. Der Unterschied zwischen Gruppe 1 und 2 wird mittels Mann

	Whitney-U Test bestimmt. Für die ordinalen Variablen FES-I und FOGQ, die auch beim Follow-up Anruf erhoben werden, wird über alle Gruppen und Testzeitpunkte hinweg ein Kruskal Wallis Test durchgeführt. Veränderungen der Sturzrate (nominal) zwischen Baseline, Postintervention und Follow-up werden mittels Chi-Square Test berechnet. Metrische Daten werden auf Homogenität der Varianz (Levene Test) als Grundannahme eines 2 x 2 ANOVA überprüft, mit „Zeit (Baseline, Postintervention)“ als „within-factor“ und „Gruppe (1, 2)“ als „between-factor“. Sollte keine Homogenität der Varianz vorliegen, wird eine alternative F-Statistik (z.B. nach Welch oder Brown-Forsythe) verwendet. Bei Nichterfüllen der Grundannahme der Sphärizität werden korrigierte Werte z.B. nach Greenhouse-Geisser oder Huynh-Feldt verwendet. Zur Berechnung der Unterschiede zwischen den Gruppen und Messzeitpunkten ein 2 x 2 ANOVA (Varianzanalyse) durchgeführt, bei vorliegender statistischer Signifikanz gefolgt von paarweisen Vergleichen (Bonferroni Korrektur für alle Vergleiche) zwischen den Gruppen zu den Messzeitpunkten 1 und 2. Im Fall des Versagens der adaptiven Maßnahmen werden nicht parametrische Tests eingesetzt wie für ordinalskalierte Daten beschrieben. Die Effektstärke wird mittels partiellem Eta Quadrat und standardisierter Effektstärke angegeben.
**Zeitplan**	Studienbezogen:<br>Rekrutierungszeit: 8 Monate<br>15.04.2021 Geplanter Beginn: First Patient First Visit (FPFV)<br>15.11.2021 Last Patient First Visit (LPFV)/Last Subject In (LSI)<br>14.12.2021 Geplantes Ende: Last Patient Last Visit (LPLV)/Last Subject Out (LSO)<br><br>Patient*innenbezogen:<br>Aktive Interventionsdauer: 4 Wochen

	Nach dem Einschluss entspricht die Dauer der Teilnahme jedes Teilnehmenden 8 Wochen (Baseline-Erhebung, 4-Wochen Intervention, Post-intervention Testung, Fokusgruppe nur für Teilnehmer*innen der Interventionsgruppe 1, Follow-up Anruf 4 Wochen nach Interventionsende)

**Funding**	Diese klinische Studie ist eine eigenfinanzierte, akademische Studie des Reha Zentrum Münster, [redacted]
**GCP Statement**	Die Studie wurde in Übereinstimmung mit den Anforderungen des Tiroler Krankenanstaltengesetzes (Tir KAG), der Deklaration von Helsinki und den ICH-E6 Richtlinien, den OeAWI Richtlinien für Gute Wissenschaftliche Praxis sowie den Vorgaben der Datenschutz-Grundverordnung (DSGVO) und des österreichischen Datenschutzgesetzes (DSG) geplant.

Abkürzungsverzeichnis

ABC Scale	Activities-specific Balance Confidence Scale
ADLs	Aktivitäten des täglichen Lebens
BBS	Berg Balance Scale
BDI-II	Beck Depression Inventory
DSG	österreichischen Datenschutzgesetzes
DSGVO	Datenschutz-Grundverordnung
FES-I	Falls Efficacy Scale
FGA	Funktional Gait Assessment
FOGQ	Freezing of Gait Questionnaire
ID	Identifikationsnummer
LPLV	Last Patient Last Visit
LQ	Lebensqualität
LSI	Last subject in
LSO	Last subject out
MDC	Minimum detectable change
MMST	Mini Mental Status Test
ND	Nicht dokumentiert
NW	Nebenwirkung
P	Pause
PDQ-39	Parkinsons Disease Questionnaire-39
PI	Post Intervention
R&TTE	Funkanlagen und Telekommunikationsendeinrichtungen (Radio an Telecommunications Terminal Equipment Directive)
Scr	Screening
SD	Standardabweichung (standard deviation)
SNW	Schwerwiegende Nebenwirkung
SUE	Schwerwiegendes unerwünschtes Ereignis
THS	Tiefe Hirnstimulation
Tir KAG	Tiroler Krankenanstaltengesetz
TUG	Timed-Up-and-Go
TUGman	Timed-Up-and-Go with manual dual task
UE	Unerwünschtes Ereignis
UPDRS	Unified Parkinson's Disease Rating Scale
10MWT	10-Minute Walk Test

1. Einleitung

1.1 Hintergrund der Studie

Das idiopathische Parkinson Syndrom gehört mit einer Prävalenz von weltweit 6,1 Millionen Menschen (Stand 2016) zu einer der häufigsten neurologischen Erkrankungen der Personen über sechzig Jahren. Seit 1990 gab es eine Steigerung um 14,5% (“Global, regional, and national burden of Parkinson’s disease, 1990-2016: a systematic analysis for the Global Burden of Disease Study 2016.,” 2018). Es ist die am schnellsten zunehmende neurologische Erkrankung (Tönges et al., 2019). Bei Personen über 65 Jahren liegt die Prävalenz in Österreich bei 16.226 und in Deutschland bei 260.817 Fällen (Andlin-Sobocki et al., 2005). Die verursachten Kosten pro Fall belaufen sich in Österreich auf 9291 Euro und in Deutschland auf 11138 Euro (Andlin-Sobocki et al., 2005).

Die Kardinalsymptome des idiopathischen Parkinson Syndrom sind Akinese, Rigor, Ruhetremor und posturale Instabilität in unterschiedlichen Ausprägungen. (Keus, S. et al., 2014). Weiterhin können Okulomotorikstörungen wie beispielsweise eine verringerte Sakkadengeschwindigkeit auftreten (Matsumoto et al., 2011). Eine verringerte Sakkadenamplitude kann eine der Hauptursachen für Einschränkungen der Wahrnehmung der Umwelt darstellen (Matsumoto et al., 2011). Veränderungen der Sakkadenparameter in Kombination mit der Bradykinese führen bei den Betroffenen zu einem erhöhten Sturzrisiko im Alltag (Cucca et al., 2018). Dies kann wiederum zu Einschränkungen der Selbstständigkeit in den Aktivitäten des täglichen Lebens (ADLs) führen, was die Partizipation der Betroffenen negativ beeinflusst (Keus, S. et al., 2014).

Alcock und Kolleg*innen (2020) zeigen in ihrer Studie, dass die visuellen Dysfunktionen bei den Personen mit Idiopathischem Parkinsonsyndrom mit dem Sturzrisiko korrelieren und dieses erhöhen. Bei der Therapie der Personen mit Idiopathischem Parkinsonsyndrom sollten die visuellen Dysfunktionen entsprechend adressiert werden, um die Koordination zu fördern und das Sturzrisiko zu verringern (Ambati et al., 2016).
Stuart und Kolleg*innen wiesen 2018 nach, dass visuelle Cues die Sakkadenfrequenz bei Personen mit Idiopathischem Parkinsonsyndrom verbessern. Des Weiteren erreichten Baker und Kolleg*innen (2020) durch den Einsatz von visuellen Cues eine veränderte Abfolge der zuerst reagierenden Körpersegmente bei der Drehung während des Gehens. Dadurch erlangten die Personen mit Idiopathischem Parkinsonsyndrom eine antizipatorische Anpassung der Augenbewegungen wieder (Baker et al., 2020).

Diese Erkenntnisse deuten auf eine positive Beeinflussung der Sakkadenparameter durch ein gezieltes Blickbewegungstraining hin. Ein solches Training führt potentiell auch zu einer verbesserten Wahrnehmung der Umwelt, was die Gangsicherheit positiv beeinflussen und somit die Sturzgefahr reduzieren könnte.

Neurologische Physiotherapie bei Idiopathischem Parkinsonsyndrom im Reha Zentrum Münster ist evidenzbasiert und orientiert sich an internationalen Guidelines für die Rehabilitation (Keus, S. et al., 2014; Grimes et al., 2019; National Institute for Health and Care Excellence (Great Britain), 2017). Nicht untersucht wurde bisher, ob ein zusätzliches Blickbewegungstraining einen zusätzlichen Benefit hinsichtlich der dynamischen Mobilität und Sturzrate der Patient*innen erzielt.

1.2 Notwendigkeit der Durchführung einer Studie

Der bisherige Forschungsstand im Bereich der Interventionen zur Verbesserung der Okulomotorik weist teilweise kontroverse Ergebnisse auf. Matsumoto und Kolleg*innen beschreiben eine stärkere und längere Fixierung auf Hindernisse bei Personen mit Idiopathischem Parkinsonsyndrom im Vergleich zu den gesunden gleichaltrigen Personen der Kontrollgruppe (Matsumoto et al., 2011). Barbieri und Kolleg*innen (2018) beschreiben bei Personen mit Idiopathischem Parkinsonsyndrom eine verminderte Fixation auf ein Hindernis und eine längere Fixation auf den Boden (Barbieri et al., 2018; Reed-Jones & Powell, 2017)(Reed-Jones and Powell, 2017b). Außerdem ermitteln Matsumoto und Kolleg*innen eine geringere Sakkadenamplitude und eine geringere Anzahl der Sakkaden, was auf ein vermindertes Erfassungsvermögen von Gegenständen und der Umwelt hindeutet. Dies hat zur Folge, dass die Partizipation von Personen mit Idiopathischem Parkinsonsyndrom durch das eingeschränkte Erfassungsvermögen reduziert ist (Matsumoto et al., 2011).

Cucca und Kolleg*innen (2018) ermittelten eine Korrelation der Sehfähigkeit mit der Lebensqualität der Studienteilnehmenden. So könnte eine verbesserte Sehfähigkeit auch die Lebensqualität der Personen mit Idiopathischem Parkinsonsyndrom erhöhen (Cucca et al., 2018).
Für das Jahr 2040 werden weltweit bis zu 14,2 Millionen Personen mit Idiopathischem Parkinsonsyndrom prognostiziert. Durch die zunehmende Prävalenz wird der Versorgungsbedarf in stationären und ambulanten Versorgungsstrukturen steigen (Tönges et al., 2019). Deshalb erscheint es besonders wichtig, dass es sowohl für den stationären als

auch für den ambulanten Sektor Interventionen zum Erhalt der längst möglichen Eigenständigkeit der Betroffenen gibt. Um eine Intervention möglichst alltagsnahe zu gestalten muss diese an die gegebenen Versorgungsstrukturen des jeweiligen Settings angepasst werden. Personen mit Idiopathischem Parkinsonsyndrom haben in Österreich die Möglichkeit, jedes Jahr oder alle zwei Jahre eine stationäre Rehabilitation durchzuführen. Deshalb erscheint es sinnvoll, eine Intervention im stationären Rehabilitationssetting für 4 Wochen zu integrieren und so unter „Real-life“ Bedingungen zu erproben (Carpinella et al., 2017; Martinez-Martin et al., 2011; Silva de Lima et al., 2018).

Zur Entwicklung der Intervention dieser klinischen Studie wurden die Richtlinien des *Medical Research Councils* (2006) zur Entwicklung komplexer Interventionen verwendet, um unter anderem geeignete Methoden zur Untersuchung der Forschungsfrage zu finden (Identifikation der Evidenz, Entwicklung einer Theorie und Modellierung des Prozesses und der Outcomes) (Craig, P. et al., 2006). Somit kann diese klinische Studie einen Mehrwert für die zukünftige Versorgung von Personen mit Idiopathischem Parkinsonsyndrom leisten. Der Erhalt der Selbstständigkeit und die Verminderung der Pflegebedürftigkeit von Personen mit Idiopathischem Parkinsonsyndrom sollen als übergeordnete Ziele angestrebt werden.

Mehr als ein Drittel der Stürze bei Personen mit Idiopathischem Parkinsonsyndrom treten als Folge eines Stolperns durch ein eingeschränktes Sehvermögen als beitragender Faktor auf (Alcock et al., 2020). Dies verdeutlicht die Relevanz der Sturzprophylaxe zum Erhalt der Selbstständigkeit bei Personen mit Idiopathischem Parkinsonsyndrom. Durch die Korrelation der visuellen Dysfunktionen und einem erhöhten Sturzrisiko liegt es nahe, dass ein gezieltes Augen- und Blickbewegungstraining die Sturzrate reduzieren kann (Cucca et al., 2018). Eine Verbesserung der Geschwindigkeit der Augen- und der Blickfixation führt zu einer besseren Identifikation von Hindernisse, einer Reduktion der Gangunsicherheit und dadurch zu einer Verminderung des Sturzrisikos (Ambati et al., 2016). Langfristig kann so die Selbstständigkeit der Personen mit Idiopathischem Parkinsonsyndrom erhalten werden (Ambati et al., 2016).

1.3 Risiko- Nutzen Abschätzung

Es handelt sich beim aktivitätsorientierten physiotherapeutischen Programm grundsätzlich um eine evidenzbasierte Behandlungsmethode anhand internationaler Guidelines zur Rehabilitation bei Idiopathischem Parkinsonsyndrom, welche im Zuge der konventionellen Rehabilitation von Physiotherapeut*innen angeleitet wird. Diese physiotherapeutische Maßnahme findet in beiden Gruppen im selben Ausmaß zusätzlich zur üblichen stationären interdisziplinären Rehabilitation am Reha Zentrum Münster statt. Das Gesamttrainingsausmaß

während des Rehabilitationsaufenthaltes und dessen Intensität entspricht den zitierten internationalen Guidelines für Rehabilitation bei Idiopathischem Parkinsonsyndrom. Das Blickbewegungstraining wird mit dem aktivitätsorientierten physiotherapeutischen Programms durchgeführt und ebenso von erfahrenen und geschulten Physiotherapeut*innen angeleitet. Basierend auf der klinischen Erfahrung und Literatur ist davon auszugehen, dass die Studienteilnehmer*innen in der Interventionsgruppe mindestens ebenso von der Behandlung profitieren wie in der Standardtherapiegruppe.

Die Intervention wird zunächst sitzend durchgeführt, um eine Gewöhnung an das Augenbewegungs-Training zu erzielen. Es ist nicht davon auszugehen, dass während der Intervention Stürze auftreten, da die Patient*innen kontinuierlich unter physiotherapeutischer Supervision sind. Schwindel oder Augenermüdung könnten durch das Training initial ausgelöst werden (Camacho et al., 2019a). Dieses Risiko wird durch eine Begrenzung der Behandlungsdauer auf viermal 30 Minuten pro Woche minimiert. Das Augenbewegungstraining wird im 30-Minuten Zeitraum nicht kontinuierlich durchgeführt, sondern intermittierend mit aktivitätsorientiertem Training, um eine Überlastung von vornherein zu vermeiden. Die Intensität und Dauer der Interventionen werden als gering belastend eingestuft. Vor, während und nach der Intervention werden eventuelle Beschwerden und Nebenwirkungen erfragt. Da sich die Patient*innen für den stationären Rehabilitationsaufenthalt im Reha Zentrum Münster befinden und die Intervention in den täglichen Therapieplan integriert werden sollen, fallen keine zusätzlichen Belastungen hinsichtlich einer Anreise an. Dem zu erwartenden Nutzen – die Behandlungsoptimierung und einer effizienteren Rehabilitation – stehen, nach derzeitigem medizinischem Wissen, somit nur ein geringes Gesundheitsrisiko und eine lediglich geringe Belastung der Patient*innen gegenüber.

2 Studienziele und Hypothesen

2.1 Studienzweck

Studienziel ist es, die Effekte eines aktivitätsorientierten physiotherapeutischen Trainingsprogramms mit einem Blickbewegungstraining im Vergleich zu einem aktivitätsorientierten physiotherapeutischen Trainingsprogramm ohne Blickbewegungstraining auf die dynamische Balance, Mobilität, Gehgeschwindigkeit, gesundheitsbezogene Lebensqualität, Freezing of Gait, sturzassoziierte Selbstwirksamkeit, Depression und Sturzrate bei Personen mit Idiopathischem Parkinsonsyndrom zu untersuchen. Ziel ist außerdem, die Veränderung der Augenbewegungen (Sakkaden-geschwindigkeit, -Amplitude,

-Latenzzeit, Fixationsdauer) über den Interventionszeitraum hinweg zu explorieren und die Machbarkeit einer größeren randomisierten kontrollierten Studie anhand vordefinierter Parameter zu evaluieren.

2.2 Forschungsfrage

Die Forschungsfrage, die der Studie zu Grunde liegt, lautet wie folgt:

„Gibt es einen Unterschied zwischen den Effekten eines alltagsorientierten physiotherapeutischen Trainingsprogramms mit versus ohne einem Blickbewegungstraining auf die dynamische Balance, Mobilität, Gehgeschwindigkeit, gesundheitsbezogene Lebensqualität, Freezing of Gait, sturzassoziierte Selbstwirksamkeit, Depression und Sturzrate bei Personen mit Idiopathischem Parkinsonsyndrom?"

2.3 Studienziele

Somit werden zur Vertiefung der Erkenntnisse zur Effektivität vier **Studienziele** verfolgt:

1. Einschätzung der Effekte auf die dynamische Balance (primärer Endpunkt)
2. Untersuchung der Machbarkeit einer größeren randomisierten kontrollierten Studie
3. Einschätzung der Effekte auf die funktionelle Mobilität mit und ohne manuellem Multi Task, statisches und dynamisches Gleichgewicht, die Gehgeschwindigkeit, gesundheitsbezogene Lebensqualität, Freezing of Gait, sturzassoziierte Selbstwirksamkeit, Depression und Sturzrate (sekundäre Endpunkte)
4. Evaluierung der Veränderung der Augenbewegungen während der Intervention, in der Interventionsgruppe 1x wöchentlich gemessen über den Interventionszeitraum hinweg.

2.4 Nullhypothesen

Primäre Nullhypothese: Es gibt keinen Unterschied zwischen den Effekten eines alltagsorientierten physiotherapeutischen Trainingsprogramms mit einem Blickbewegungstraining und einem solchen Trainingsprogramm ohne einem Blickbewegungstraining auf die dynamische Balance bei Personen mit Idiopathischem Parkinsonsyndrom.

Sekundäre Nullhypothesen: Es gibt keinen Unterschied zwischen den Effekten eines alltagsorientierten physiotherapeutischen Trainingsprogramms mit einem

Blickbewegungstraining und einem solchen Trainingsprogramm ohne einem Blickbewegungstraining auf die funktionelle Mobilität mit und ohne manuellem Multi Task, statisches Gleichgewicht, die Gehgeschwindigkeit, gesundheitsbezogene Lebensqualität, Freezing of Gait, sturzassoziierte Selbstwirksamkeit, Depression, Sturzrate und Augenbewegungen während Aufgaben der dynamischen Balance und spezifischer Trainingsitems bei Personen mit Idiopathischem Parkinsonsyndrom.

2.5 Alternativhypothesen

Primäre Alternativhypothese: Es gibt einen Unterschied zwischen den Effekten eines alltagsorientierten physiotherapeutischen Trainingsprogramm mit einem Blickbewegungstraining und einem solchen Trainingsprogramm ohne einem Blickbewegungstraining auf die dynamische Balance bei Personen mit Idiopathischem Parkinsonsyndrom.

Sekundäre Alternativhypothesen: Es gibt einen Unterschied zwischen den Effekten eines alltagsorientierten physiotherapeutischen Trainingsprogramm mit einem Blickbewegungstraining und einem solchen Trainingsprogramm ohne einem Blickbewegungstraining auf die funktionelle Mobilität mit und ohne manuellem Multi Task, statisches Gleichgewicht, die Gehgeschwindigkeit, gesundheitsbezogene Lebensqualität, Freezing of Gait, sturzassoziierte Selbstwirksamkeit, Depression, Sturzrate und Augenbewegungen während Aufgaben der dynamischen Balance und spezifischer Trainingsitems bei Personen mit Idiopathischem Parkinsonsyndrom.

3 Studienbeschreibung

3.1 Studiendesign

Prospektive doppelblinde randomisierte kontrollierte Pilotstudie

3.2 Studienzentrum

Reha Zentrum Münster Betriebs GmbH, Gröben 700, 6232 Münster

3.3 Zuständige Ethikkommission

Die für diese Studie zuständigen Ethikkommission ist die Ethikkommission der Medizinischen Universität Innsbruck

3.4 Registrierung der Studie

Nach Erhalt eines positiven Votums der Ethikkommissionen der Medizinischen Universität Innsbruck wird die Studie prospektiv im DRKS, ISRCTN oder einem vergleichbaren von der WHO anerkannten Register registriert.

3.5 Zeitplan

Studienbezogen:

Rekrutierungsdauer: 8 Monate

15.04.2021 Geplanter Beginn: First Patient First Visit (FPFV)

15.11.2021 Last Patient First Visit (LPFV)/Last Subject In (LSI)

14.12.2021 Geplantes Ende: Last Patient Last Visit (LPLV)/Last Subject Out (LSO)

Patient*innenbezogen:

Aktive Interventionsdauer: 4 Wochen

Nach dem Einschluss entspricht die Dauer der Teilnahme jedes Teilnehmenden 8 Wochen (Baseline-Erhebung, 4-Wochen Intervention, Post-intervention Testung, Fokusgruppe nur für Teilnehmer*innen der Interventionsgruppe 1, Follow-up Anruf 4 Wochen nach Interventionsende). Die Gesamtlaufzeit der Studie wird sich auf 8 Monate belaufen.

Abbildung 1 präsentiert den patient*innenbezogenen Studienablauf.

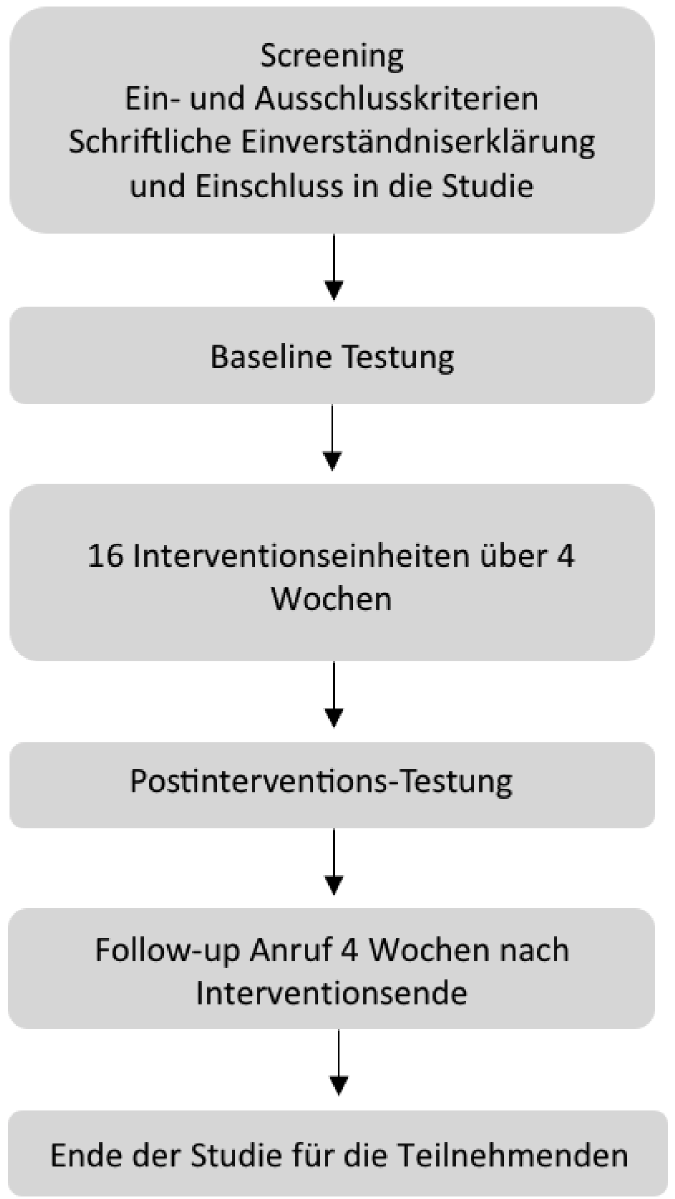

Abbildung 1: Patient*innenbezogener Studienablauf

4 Methoden

4.1 Studienteilnehmer*innen

4.1.1 Teilnehmer*innenrekrutierung und Studieneinschluss

Alle im Rekrutierungszeitraum im Reha Zentrum Münster behandelten Personen mit Idiopathischem Parkinsonsyndrom werden durch das ärztliche Personal hinsichtlich ihrer Eignung für die Studienteilnahme überprüft. Alle Personen mit Idiopathischem Parkinsonsyndrom, die den Einschlusskriterien dieser Studie entsprechen und keines der Ausschlusskriterien erfüllen, werden durch den jeweiligen Prüfarzt oder die jeweilige Prüfärztin mündlich und schriftlich über die Studie aufgeklärt. Nach Feststellen einer prinzipiellen Eignung (Einschlusskriterien) und der Einwilligung der*des Patient*in, das heißt ihrem*seinem Interesse, an der Studie teilzunehmen, wird ein Screening durchgeführt. Unter Verwendung prädefinierter Cut-Off Scores des Mini-Mental-Status-Test (MMST) (Folstein et al., 1975) ≥24/30 Punkten wird eine moderate bis schwere kognitive Einschränkung ausgeschlossen. Im Fall des Fehlens dieser Ausschlusskriterien erfolgt der unten beschriebene Studieneinschluss.

Nach einer angemessenen Bedenkzeit, der Möglichkeit für Rückfragen und der Zustimmung des/r Patient*in zur Teilnahme wird anschließend das Informationsschreiben und die Einwilligungserklärung für Patient*innen sowohl von dem/r Patient*in als auch vom Arzt oder der Ärztin datiert und unterschrieben. Der/die Prüfer*in vermerkt die Teilnahme auf einer speziellen Patient*innenidentifizierungsliste. Sie dient der Möglichkeit der späteren Identifikation der Patient*innen und enthält die Identifikationsnummer (ID), den vollständigen Namen der*des Patient*in, das Geburtsdatum und das Datum der Aufnahme in die klinische Prüfung. Die Patient*innenidentifikationsliste verbleibt nach Abschluss der Prüfung am Prüfzentrum. Die ID wird mit dem Datum des Studieneinschlusses in die beschriebene und von einer unabhängigen Forschungsperson erstellte Randomisierungsliste eingetragen.

4.1.2 Studienpopulation

4.1.2.1 Einschlusskriterien

Männer und Frauen mit Idiopathischem Parkinsonsyndrom anhand der UK Brain Bank Kriterien (Hughes et al., 1992), Stadium 1-3 nach Hoehn & Yahr) bei ON Medikation (Hoehn and Yahr, 1967), Alter 30-80 Jahre, jeglicher Ethnizität, Gehfähigkeit ohne Hilfsperson, Mini-Mental-Status-Test (MMST) ≥ 24/30 (Tombaugh and McIntyre, 1992), stabile Dosierung dopaminerger Ersatztherapie mind. 3 Wochen vor Studienbeginn oder noch ohne

dopaminerge Behandlung für die Dauer der Studienintervention, deutschsprachig in Wort und Schrift.

4.1.2.2 Ausschlusskriterien

Konkomitante Erkrankung(en) (wie maligne Erkrankungen, andere neurologische, orthopädische, kardiale oder psychiatrische Erkrankungen, Major Depression, Demenz), Photosensitivität, nicht-parkinsonistische Gangstörung (z.B. aufgrund muskuloskelettaler Symptome), rezenter chirurgischer Eingriff (allgemein und an den Augen), intraoculäre Implantate, Strabismus, Nystagmus, ausgeprägte Schlupflider, unbehandelte Schmerzen, unkorrigierte Visus- oder Hörbeeinträchtigung, die das Training oder die Untersuchungen beeinträchtigen würden, Schwangerschaft, rezente Tiefe Hirnstimulation (THS) oder Änderung der Parameter der THS innerhalb des letzten Jahres, schwere motorische Fluktuationen, Initiierung einer neuen dopaminergen Medikation oder Anpassung derselben innerhalb des Studienzeitraums ist nicht vorgesehen; im Sinn eines Real-Life Settings obliegt diese Entscheidung dem Prüfarzt oder der Prüfärztin.

Zur Korrektur von Fehlsichtigkeit werden zum mobilen Eye Tracker passende Korrekturlinsen eingesetzt.

4.1.2.3 Anzahl der Teilnehmenden und Stichprobengröße

Für diese Studie sollen 34 Personen mit Idiopathischem Parkinsonsyndrom eingeschlossen werden. Von den 34 Personen mit Idiopathischem Parkinsonsyndrom sollen ja 17 in die Interventionsgruppe 1 und 17 in die Kontrollgruppe 2 randomisiert werden.

Laut Julious werden Teilnehmer*innen pro Gruppe als eine minimale Stichprobengröße für eine Pilotstudie betrachtet (Julious, 2005), während Browne für eine zweiarmige Studie eine Teilnehmer*innenanzahl von 30 angibt (Browne, 1995). Zur Evaluierung der Machbarkeitskriterien und präliminären Effekte experimentellen Intervention im Vergleich zur konventionellen Kontrollintervention wird daher – basierend auf 30 Teilnehmer*innen - inklusive einer erwarteten Ausfallrate von 10% und unter Anwendung der Formel N=n/(1-(z/100)) eine Stichprobengröße von 34 angestrebt. Dabei ist n die Teilnehmer*innenanzahl von 30 und z die erwartete Ausfallrate von 10%.

4.1.3 Randomisierung und verdeckte Zuteilung

34 Patienten mit Idiopathischem Parkinsonsyndrom werden mittels einer 1:1 Zuteilungs-Ratio in eine von zwei Gruppen randomisiert. Dafür wird eine mittels der Randomisierungssoftware „Sealed Envelope“ (https://www.sealedenvelope.com/) generierte Zufallszahlen Sequenz

eingesetzt sowie verschlossene blickdichte Kuverts. Eine stratifizierte Blockrandomisierung (Blöcke zu 4 und 6) mit verdeckter Zuteilung wird durch einen unabhängigen Forscher im Team durchgeführt, welcher nicht in die Teilnehmerrekrutierung, Instruktionen hinsichtlich der Intervention oder Testungen involviert sein wird. Die Stratifizierung wird aufgrund der kleinen Stichprobe nur anhand eines relevanten prädiktiven Faktors für eine Veränderung der dynamischen Mobilität erfolgen, nämlich dem Behinderungsgrad (H & Y Stadien 1 und 2; 3).

4.1.4 Verblindung

Die Rater*innen/Testende der Studie werden zur Gruppenzuweisung der Teilnehmenden verblindet, und die Teilnehmenden werden zu den Studienhypothesen verblindet.

4.1.5 Entblindung

Der geplante Zeitpunkt der regulären Entblindung ist nach Abschluss der Studie, konkret nach Finalisierung der Datenanalyse.

4.2 Intervention

4.2.1 Behandlungsschema

Die Studie umfasst ein Screening, eine Baseline Untersuchung, 16 Interventionseinheiten a‘ 30 min über 4 Wochen, eine Postintervention Testung, eine Fokusgruppe für die Teilnehmenden der Interventionsgruppe und einen Follow-up Anruf 4 Wochen nach Interventionsende.

Die Dauer und Anzahl der Untersuchungen und Behandlungen (Tabelle 1) finden im Kontext der stationären interdisziplinären Rehabilitation der Patient*innen statt. Es wird gewährleistet, dass alle Studienteilnehmenden insgesamt dieselbe Anzahl an Behandlungen erhält. Außerdem müssen alle Teilnehmenden 8 30-min Einheiten Physiotherapie pro Woche erhalten, wovon 4 der Studienintervention in Gruppe 1 oder 2 vorbehalten sind und eine zusätzliche Maßnahme darstellen. Alle Therapeut*innen werden auf die Studieninterventionen und Instruktionen für die Teilnehmenden beider Gruppen eingeschult und arbeiten daher nach denselben Prinzipien.

Tabelle 1: Behandlungsschema eines/r (gescreenten möglichen*) Studienteilnehmenden

Intervention	Dosierung	Zeitpunkt	Dauer

Screening*	1x	Nach Überprüfung auf Eignung durch Prüfärztin/Prüfarzt und Einwilligung des/r Patient*in	10-15 min
Studieneinschluss			
Baseline-Untersuchung	1x	Tag 0 nach Studieneinschluss	75-90 min
Intervention Gruppen 1 und 2	16x	Beginn an Tag 1 nach der Baseline-Untersuchung<br>4 x pro Woche, insges. 4 Wochen	Je 30 min
Postintervention Testung	1x	4 Wochen nach Beginn der Intervention	75-90 min
Fokusgruppe	1x	4 Wochen nach Beginn der Intervention, nur für Interventionsgruppe 1, in zeitlichem Abstand von >2 Stunden von der Postintervention Testung	45-60 min
Follow-up Anruf	1x	4 Wochen nach Interventionsende	15 min

*Sollte nach dem Screening ein oder mehrere Ausschlusskriterien vorliegen, wird der/die Patient*in nicht in die Studie eingeschlossen.

4.2.2 Intervention

Die Intervention wird zusätzlich zu den Therapieeinheiten der stationären Rehabilitation am Reha Zentrum Münster durchgeführt. Alle durch die Studienintervention nicht adressierten Patient*innenziele sowie neurotherapeutischen Therapiemaßnahmen werden somit während der üblichen Therapieeinheiten durchgeführt (z.B. auditives Cueing, kognitive Strategien, Ausdauertraining, spezifisches Haltungstraining).

Interventionsgruppe (Gruppe 1): Aktivitätsorientiertes physiotherapeutisches Trainingsprogramm mit Blickbewegungstraining; das Training ist evidenzbasiert und an Guidelines für die Rehabilitation bei Idiopathischem Parkinsonsyndrom orientiert (Keus, S. et al., 2014; Grimes et al., 2019; National Institute for Health and Care Excellence (Great Britain),

2017): für 30 min, 4x pro Woche, 4 Wochen lang, supervidiert durch die jeweiligen Physiotherapeut*innen.

Kontrollgruppe (Gruppe 2): Aktivitätsorientiertes physiotherapeutisches Trainingsprogramm i. S. der Standardversorgung; die Standardversorgung ist evidenzbasiert und an Guidelines für die Rehabilitation bei Idiopathischem Parkinsonsyndrom orientiert (Keus, S. et al., 2014; Grimes et al., 2019; National Institute for Health and Care Excellence (Great Britain), 2017): für 30 min, 4x pro Woche, 4 Wochen lang, supervidiert durch die jeweiligen Physiotherapeut*innen.

Das Training beinhaltet daher ein hinsichtlich des Hoehn und Yahr Stadiums angepasstes und damit individualisiertes Training, das insgesamt divers und variabel ist. Die im folgenden aufgelisteten Trainingscharakteristika betreffen das Training in beiden Interventionsgruppen. Der Unterschied zwischen den beiden Gruppen ist lediglich das Blickbewegungstraining in Kombination mit den unten näher beschriebenen Übungen.

- Anwendung der Prinzipien des motorischen Lernens (Externer Lernfokus, Repetition, Variabilität, Progression, aktivitätsorientiert, zielorientiert)
- Spezifisches funktionelles Gleichgewichtstraining (statisches und dynamisches Gleichgewichtstraining, z.B. sich zunehmend über Stabilitätsgrenzen des Körpers hinausbewegen, gesichert durch die*den Physiotherapeut*in)
- Transfers wie z.B. Sitz-Stand
- Funktionelle Übungen zur Verbesserung der Gelenksbeweglichkeit (z.B. Rumpfrotation mit progressiver Steigerung in höhere Körperposition)
- Funktionelles Krafttraining
- Koordinationstraining
- Sturzprophylaxe Training
- Training großer Bewegungsamplituden mit hoher Intensität

Das *aktivitätsorientierte, physiotherapeutische Trainingsprogramm mit Blickbewegungstraining (Gruppe 1*) wird aus folgenden Elementen bestehen: Ein evidenzbasiertes Routineprogramm, welches die Parameter des motorischen Lernens (Olson et al., 2019), des spezifischen funktionellen Gleichgewichtstraining, der Verbesserung der Gelenksbeweglichkeit, der Sturzprophylaxe und der funktionellen Selbständigkeit (Keus, S. et al., 2014) adressiert, wird mit einem Blickbewegungstraining kombiniert. Die Auswahl der Parameter des Blickbewegungstrainings basierten auf verschieden Studien, welche ein Blickbewegungstraining bereits an anderen Populationen untersucht haben (Camacho et al.,

2019b; Knox and Wolohan, 2015; Matsumoto et al., 2011). Dazu gehören die Intensität, die Übungszeiten für die Interventionen (in der ON Phase) und der Abstand des Trainierenden von der Wand, an der ein Übungsposter mit einem Fadenkreuz und unterschiedlichen Fixationspunkten angebracht ist.

Das Blickbewegungstraining soll den Fokus des in der Reha durchgeführten evidenzbasierten aktivitätsorientierten Routineprogramms auf die Blickfixation und Erweiterung der Sakkardenamplitude der Personen mit Idiopathischem Parkinsonsyndrom lenken. Dies bedeutet, dass zusätzlich zu den physiotherapeutischen Übungen zur Steigerung der Koordination, Kraft und Kraftausdauer Blickfixations- und Blickbewegungsübungen trainiert werden. Diese werden anhand der Parameter des motorischen Lernens (Olson et al., 2019) progressiv gesteigert und in Kombination mit den Übungen des Gleichgewichts- und Sturzprophylaxetrainings ausgeführt. Die jeweiligen Physiotherapeut*innen erhalten eine Einschulung, um eine standardisierte Durchführung der Intervention zu gewährleisten. Es steht ein Instruktor Manual mit einer Übungssammlung zur Verfügung, welche ein individualisiertes Zuschneiden des Übungsprogramms an den Leistungsstand der Teilnehmenden ermöglicht und trotzdem eine Vergleichbarkeit gewährleistet (siehe Anhang 1 und Anhang 2).

Für das Blickbewegungstraining wird ein Übungsposter mit einem Fadenkreuz und unterschiedlichen Fixationspunkten eingesetzt, dass für diese klinische Studie im interdisziplinären Team entwickelt wurde (siehe Abbildung 2a). Anhand dieses Übungsposters soll dann in Kombination mit unterschiedlichen Übungen für die untere Extremität und des Rumpfes ein standardisiertes, individuell angepasstes, progressiv gesteigertes Trainingsprogramm über den Interventionszeitraum durchgeführt werden. Zusätzlich werden Wandmarkierungen für die mit aktivitätsorientiertem Gleichgewichtstraining kombinierte Blickbewegungsintervention eingesetzt (Abbildung 2b). Die Übungen werden in verschieden Ausgangsstellungen (Sitz, breiten Stand, Tandemstand, etc.) trainiert, um damit verschiedene Bewegungen alltagsnaher Situationen zu trainieren. Die Studienteilnehmer*innen werden im 1:1 Setting im Rahmen der Einzelphysiotherapie viermal wöchentlich begleitet. Die jeweils betreuenden Physiotherapeut*innen stehen den Teilnehmenden wären des gesamten Zeitraums für Fragen bezüglich des Trainings zur Verfügung.

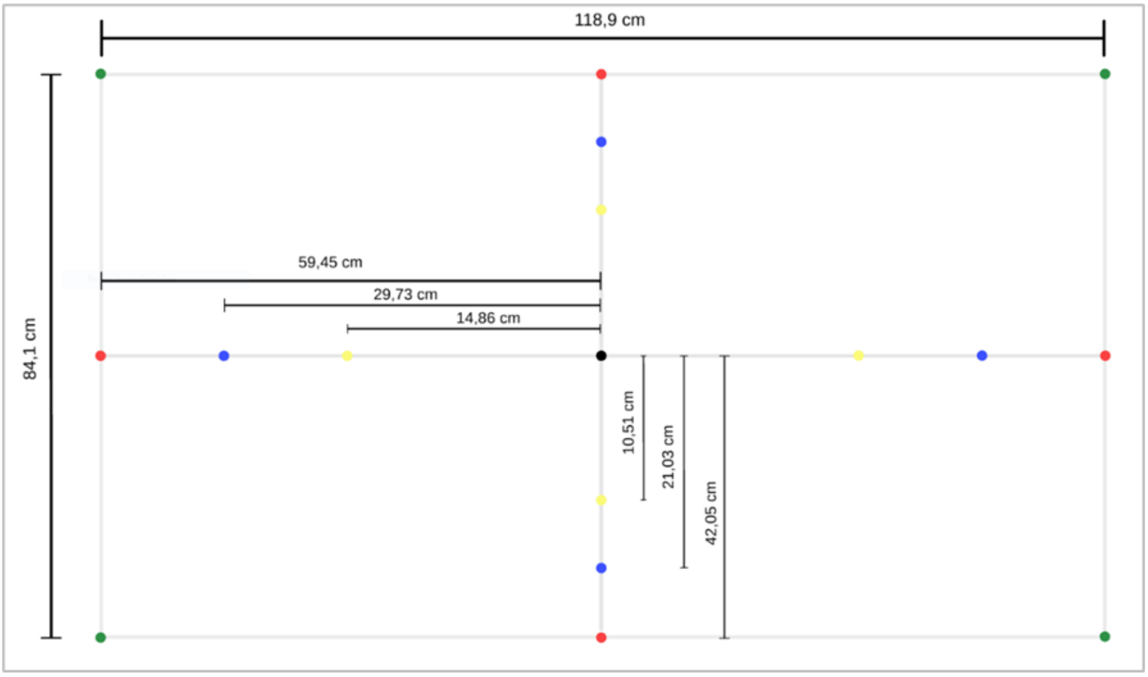

Abbildung 2a: Übungsposter

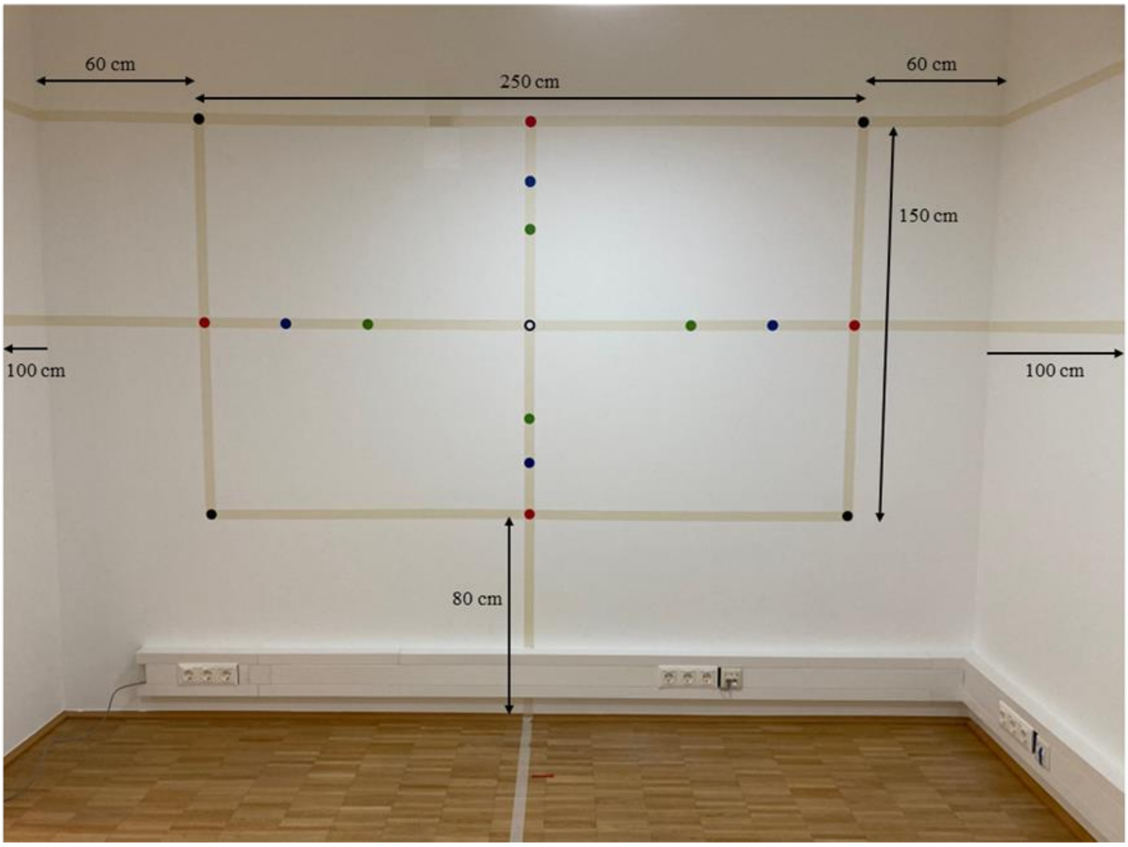

Abbildung 2b: Wandmarkierungen für mit aktivitätsorientierter Physiotherapie kombinierte Blickbewegungstrainingsintervention

Das *aktivitätsorientierte, physiotherapeutische Trainingsprogramm (Gruppe 2)* enthält dieselben evidenzbasierten, standardisierten, physiotherapeutischen Übungen zur Verbesserung der Koordination, Kraft und Kraftausdauer, ohne explizites Blickbewegungstraining. Wie in der Interventionsgruppe führt auch die Kontrollgruppe 4x pro Woche ein durch die jeweiligen Physiotherapeut*innen supervidiertes Training durch.

4.3 Endpunkte und Datenerhebung

4.3.1 Demografische und krankheitsspezifische Daten

Demografische und krankheitsspezifische Daten werden erhoben, wie das Alter und Geschlecht der Teilnehmenden, der Grad der Behinderung (UPDRS inklusive H & Y) und das Datum der Erstdiagnose, die dopaminerge Medikation und Levodopa äquivalente Dosis (mg).

4.3.2 Datenerhebung und Untersuchende

Die Datenerhebung wird zu Baseline, Post-Intervention und beim Follow-up Anruf durch geschulte und verblindete Rater*innen erfolgen (Ärzt*innen, Physiotherapeut*innen). In Fokusgruppen soll nach Interventionsende das Erleben und die Akzeptanz der Intervention der Teilnehmenden erhoben werden. Ein Follow-up Anruf wird einen Monat nach dem Interventionsende stattfinden, d.h. zwei Monate nach dem Baseline Assessment. Diese Untersuchung wird ein sehr kurzes Interview, die Sturzrate im 4-wöchigen Interventionszeitrum und zwei Fragebögen (FOGQ, FES-I) inkludieren.

4.3.3 Primäre und sekundäre Endpunkte und Erhebungsinstrumente

In Tabelle 2 ist eine Auflistung der primären und sekundären Endpunkte der Studie mit den jeweiligen ausgewählten Erhebungsinstrumenten enthalten. Alle Assessments werden in ihrer validierten deutschen Fassung verwendet. Durchführung und psychometrische Kriterien der klinischen Assessments sind in Kapitel 4.4 beschrieben.

Tabelle 2: Primäre und sekundäre Endpunkte und Erhebungsinstrumente

Endpunkt	Erhebung	Scr	BL	Int.	PI	FU An-ruf

	Primäre klinische Endpunkte					
Dynamische Balance	Functional Gait Assessment (FGA)		X		X	
Dynamische Balance mit Augenbewegungen (Sakkadengeschwindigkeit, -Amplitude, -Latenzzeit, Fixationsdauer)	Items 5, 6 und 10 des Functional Gait Assessment (FGA) mit mobile Eye Tracking mittels Tobii Pro Glasses 3		X		X	
	Sekundäre klinische Endpunkte					
Kognitive Funktion	Mini-Mental State Test (MMST)	X				
Funktionelle Mobilität	Timed-Up-and-Go (TUG)		X		X	
Funktionelle Mobilität mit manuellem Dual-Task	Timed-Up-and-Go with manual dual task (TUGman)		X		X	
Statisches und dynamisches Gleichgewicht	Berg Balance Scale (BBS)		X		X	
Dynamisches Gleichgewicht	Four Square Step Test (FSST)		X		X	
Gehgeschwindigkeit	10-Metre Walk Test (10MWT)		X		X	
Gesundheitsbezogene Lebensqualität	Parkinson‘s Disease Questionnaire-39 (PDQ-39)		X		X	
Freezing of Gait	Freezing of Gait Questionnaire (FOGQ)		X		X	X
Sturzassoziierte Selbstwirksamkeit	Falls Efficacy Scale (FES-I)		X		X	X
Depression	Beck Depression Inventory-II (BDI-II)		X		X	
Sturzrate	Anzahl der Stürze (a) im 3 Monatszeitraum vor Studienbeginn (BL), (b) Interventionszeitraum (PI) und (c) 4-Wochen Follow-up		X		X	X

Machbarkeit einer größeren randomisierten kontrollierten Studie						
	Rekrutierungsrate		X			
	Retentionsrate				X	X
	Adhärenzrate			X		
	Unerwünschte Ereignisse			X	X	
	Akzeptanz der Intervention (kontinuierlich: narrativ; Fokusgruppen in Interventionsgruppe 1)			X	X	
Augenbewegungen während der Intervention in Gruppe 1: standardisierte Übungen, jeweils am Ende von Woche 1-4, während Übung 2, 3, 4, 6 und 7	12 min lang gemessen: Sakkadengeschwindigkeit, -Amplitude, -Latenzzeit, Fixationsdauer (Tobii Pro Glasses 3)			X		
	Abkürzungen: Scr=Screening; BL=Baseline-; Int.=Interventionszeitraum; PI=Postintervention-; FU=Follow-up- Testung.					

4.3.3.1 Mobile Eye-Tracking Brille (Tobii Pro Glasses 3)

4.3.3.1.1 Einsatz und Anwendung der mobilen Eye-Tracking Brille

Der Einsatz und die Anwendung der Eye-Tracking Brille Tobii Pro Glasses 3 erfolgt folgendermaßen:

Für das Aufzeichnen der Augenbewegungen wird die Eye-Tracking Brille der Firma Tobii AB eingesetzt. Die Tobii Pro Glasses 3 tragen die CE-Kennzeichnung und erfüllen die geltenden Gesundheits- und Sicherheitsvorschriften der Europäischen Union. Die Tobii Pro Glasses 3 und deren Handhabung entsprechend den folgenden Normen:

- EMC Emission: EN55022:2010 Class B, FCC part 15, Class B
- EMC Immunity: EN55024:2010
- SAR EN62311:2008
- Low Voltage Directive 2006/95/EEG
- EMC Directive 2004/108/EC
- Radio and Telecommunications Terminal Equipment Directive (R&TTE) 1999/5/EC
- RoHS Directive 2002/95/EC

- RoHS2 Directive 2006/121/EC
- WEEE Directive 2002/121/EC
- Reach Directive 2006/121/EC
- IEC/EN/UL60950-1:2005 (Electrical safety for global/EU/US)

(ETL Compliance: Conforms to ANSI/UL Std. 60950-1 Certified to CSA Std. C22.2 No. 60950-1; Canadian Compliance Statement: CAN ICES-3(B)/NMB-3(B))

Die Tobii Pro Glasses 3 werden für die Durchführung das Assessment (Items 5, 6 & 10 des FGA) zu allen Erhebungszeitpunkten und während der Intervention bei der jeweils letzten Therapie von Woche 1-4 für jeweils ca. 12 Min von den Teilnehmenden getragen. Vor Beginn der Testungen werden die Teilnehmenden von den zuständigen Prüfenden bzw. Therapeut*innen über den Einsatz der Eye-Tracking Brille informiert und über mögliche auftretende Nebenwirkungen wie vorübergehender Schwindel aufgeklärt. Die Teilnehmenden werden zu jedem Zeitpunkt von den Physiotherapeut*innen gesichert. Sollte dies zu irgendeinem Zeitpunkt der Fall sein, schalten die zuständigen Prüfenden bzw. Therapeut*innen die Brille umgehend aus und die Teilnehmenden nehmen die Brille ab.

4.3.3.1.2 Technische Daten der Tobii Pro Glasses 3

Die Tobii Pro Glasses 3 verfügen über 16 Illuminatoren und vier Augenkameras (für jedes Auge zwei), die in die kratzfesten Linsen integriert sind und eine optimale Positionierung und ein uneingeschränktes Sichtfeld ermöglichen. Die Abtastrate (Samplingrate) beträgt 50 oder 100 Hz. Zur Kalibrierung wird das Ein-Punkt-Verfahren verwendet. Die Szenenkamera, Videoauflösung beträgt 1920 × 1080 at 25 fps. Das Gesamtgewicht der Brille (incl. Kabel) beträgt 76,5 Gramm. Die Szenenkamera hat ein breites Sichtfeld (106° horizontal: 95°, vertikal: 63°). Mithilfe des eingebautem Beschleunigungssensor, Gyroskop und Magnetometersensoren ist es möglich, separate Informationen zu Kopf- und Augenbewegungen zu erhalten und den Einfluss der Kopfbewegungen auf die Eye-Tracking Daten besser zu filtern. Zusätzlich verfügen die Tobii Pro Glasses 3 über eine große Auswahl an Nasenpads, sowie Korrekturlinsen für Personen mit Sehschwäche, um eine optimale Passform und Tragekomfort zu gewährleisten.

4.3.3.1.3 Sicherheitsvorkehrungen für die Eye-Tracking Untersuchung

Die Eye-Tracking Brille sendet über die 16 Illuminatoren zur Unterstützung der Eye-Tracking Sensoren gepulstes Infrarotlicht (IR) aus. Bestimmte medizinische und andere Geräte sind anfällig für Störungen durch IR-Licht. Deshalb wird vor der Verwendung der Tobii Pro Glasses 3 zu sichergestellt, dass die Proband*innen keine solchen empfindlichen Geräte benötigen, da deren Genauigkeit oder ordnungsgemäße Funktion beeinträchtigt werden könnte.

Die Untersuchung mit den Tobii Pro Glasses 3 dauert während der Intervention 1x/Woche 12 min und während der Baseline und Postintervention-Assessments ca. 5 min.

4.4 Durchführung und psychometrische Kriterien klinischer Assessments

4.4.1 Functional Gait Assessment

Das Functional Gait Assessment (FGA) (Wrisley et al., 2004) wird zur Beurteilung der dynamischen Balance beim Gehen verwendet und bewertet die Fähigkeit einer Person, mehrere motorische Aufgaben während des Gehens auszuführen.

4.4.1.1 Durchführung

Der FGA besteht aus 10 Items. Die Items adressieren das Gehen auf einem Untergrund ohne Steigung mit verschiedenen Geschwindigkeiten, mit Kopfdrehungen, mit schnellen Drehungen und Stoppen, Treppensteigen und das Übersteigen von Hindernissen. Die Bewertung der einzelnen Items erfolgt mit einer Skala von 0-3 Punkten, wobei 3 Punkte die bestmögliche Erfüllung des Items darstellen. Die Gesamtpunktzahl beträgt 30 Punkte. Je mehr Punkte erreicht werden, desto besser ist die dynamische Balance der Proband*innen. Die Dauer der Durchführung beträgt 5-20 Minuten (Thieme et al., 2009).

4.4.1.2 Psychometrische Kriterien

Der MDC beträgt 4 Punkte (Petersen et al., 2017). Bei Personen mit Idiopathischem Parkinsonsyndrom liegt der Cut-Off Wert bei ≤18/30 Punkten für die Identifikation eines erhöhten Sturzrisikos. Die Sensitivität beträgt 80,6% und die Spezifität 80,0% (Yang et al., 2014). Die Inter-Rater Reliabilität (ICC=0.99; 95% CI = 0,99-1,00) und die Intra-Rater Reliabilität (ICC=0,99; 95% CI = 0,99-1,00) sind beide exzellent (Yang et al., 2016). Die Test-Retest Reabilität ist mit einem ICC Wert von 0,86 ebenfalls sehr gut (Petersen et al., 2017). Cronbach´s Alpha liegt bei 0,94 und zeigt somit eine exzellente Konstruktvalidität auf (Yang et al., 2016).

4.4.2 Messung der Augenbewegungen während Items 5, 6 und 10 des FGA

Zur Erfassung der dynamischen Balance und Augenbewegungen (Sakkadengeschwindigkeit, -Amplitude, -Latenzzeit, Fixationsdauer) werden Items 5, 6 und 10 des FGA separat herangezogen. Diese Messung erfolgt mit Tobii Pro Glasses 3 wie oben erläutert.

4.4.3 Mini-Mental-Status-Test (MMST)

Der MMST (Folstein et al., 1975) dient der Beurteilung der kognitiven Funktion (zeitliche und räumliche Orientierung, Merk- und Erinnerungsfähigkeit, Aufmerksamkeit, Sprache und Sprachverständnis, außerdem Lesen, Schreiben, Zeichnen und Rechnen) und dient als Screeningtool für diese klinische Studie.

4.4.3.1 Durchführung

Der Test besteht aus Aufgaben und Fragen zur Beurteilung der kognitiven Funktion der Testperson. Es können maximal 30 Punkt erreicht werden, wobei bei einem Ergebnis ≤20 Punkte von einer Demenz ausgegangen werden kann. Der Cut-off Punkt von 24 Punkten weist reliabel und mit hoher Sensitivität und Spezifität auf eine Demenz hin (Stuss et al., 1996). Die Testdauer beträgt circa 7 bis 10 Minuten (Folstein et al., 1975).

4.4.3.2 Psychometrische Kriterien

Der kleinste messbare Unterschied (MDC) liegt bei Personen mit Parkinson mit milden neurokognitiven Störungen bei 6,43 Punkten, wobei bei der kleinste messbare Unterschied (MDC) bei Personen mit Parkinson mit schwerwiegend kognitiven Störungen bei 6,16 Punkten liegt (Lucza et al., 2015). Die Konstruktvalidität des MMST ist hoch, gezeigt anhand signifikanter Korrelationen mit der Dementia Rating Scale beträgt 0,87 ($p<0,001$) (Aarsland et al., 2004). Studien belegten, dass der MMST den kognitiven Abbau über einen längeren Zeitraum von rund 10 Jahren valide messen kann (Lessig et al., 2012).

4.4.4 Timed-Up-and-Go (TUG)

Der Timed-Up-and-Go wird zur Erhebung der funktionellen Mobilität verwendet, welche ein primärer Endpunkt dieser Studie ist (Podsiadlo and Richardson, 1991). Der TUG wird als Indikator für die Sturzgefahr bei alltäglichen Bewegungsaufgaben verwendet. Getestet wird die Durchführung der Aktivitäten Transfer vom Sitz zum Stand, Gehen, sowie eine Drehung im Raum zu machen, zurück zum Stuhl zu gehen und sich hinzusetzen. Für die Fortbewegung im Alltag und für die Reduktion des Sturzrisikos sind die Fähigkeiten Transfer Sitz-Stand und eine Drehung im Raum zu machen von entscheidender Bedeutung (Cheng et al., 1998; Mong et al., 2010).

4.4.4.1 Durchführung

Der Test beginnt im Sitz, auf einem Stuhl mit einer Sitzhöhe von ca. 46 cm. Für die Durchführung des TUG wir die Zeit gestoppt, die die Proband*innen benötigen, um von dem Stuhl aufzustehen, einen Weg von drei Metern zu gehen, eine Drehung im Raum zu machen, die drei Meter bis zum Stuhl wieder zurückzugehen und sich wieder in die Ausgangsposition zu begeben. Die benötigte Zeit wird in Sekunden dokumentiert. Es dürfen Hilfsmittel, welche die Proband*innen momentan benutzen, verwendet werden. Alle verwendeten Hilfsmittel müssen dokumentiert werden. Die Durchführung erfordert weniger als 5 Min.

4.4.4.2 Psychometrische Kriterien

Der Standard Error of Measurement (SEM) wird von Dal Bello-Haas und Kolleginnen (2011) bei Personen mit Idiopathischem Parkinsonsyndrom der (Hoehn & Yahr Stadien 1-3) mit 1,75 s angegeben. Der kleinste messbare Unterschied (Minimal Detectable Change, MDC) liegt bei 3,5 s (Huang et al., 2011). Dieselben Autor*innen geben eine exzellente Test-Retest Reliabilität bei Personen mit Idiopathischem Parkinsonsyndrom mit einem ICC Wert von 0,80 an. Der TUG weist eine exzellente Inter-Rater Reliabilität und Intra-Rater Reliabilität mit ICC Werten von 0,99 und 0.98 auf (Morris et al., 2001). Bei der Kriteriumsvalidität wurden bei Personen mit Schlaganfall sehr gute Werte im Vergleich mit dem Clinical Guideline System (CGS) (r = -0,86) dem Fast Gait Speed (FGS) (r = - 0,91) und dem 6 Meter Walk Test (r = 0,92) erreicht (Flansbjer et al., 2005). Für die Konstruktvalidität beschrieben Dal Bello-Haas eine exzellente Korrelation mit der Activities-specific Balance Confidence (ABC) Scale (r= -0-44, p= 0.03) und Bennie und Kolleg*innen (2003) eine exzellente Korrelation mit der BBS (r= -0,47, p= 0,04). Bei der prädiktiven Validität bei Personen mit Idiopathischem Parkinsonsyndrom geben Mak and Pang (2009) an, dass bei Werten >16 s ein signifikant erhöhtes Sturzrisiko vorliegt.

4.4.5 Timed-Up-and-Go with manual dual task (TUGman)

Der Timed-Up-and-Go mit manuellem Dual-Task zur Erhebung des Sturzrisikos ist ein sekundärer Endpunkt dieser Studie (Shumway-Cook et al., 2000). Der TUGman wird als Indikator für die Sturzgefahr bei alltäglichen Bewegungsaufgaben mit Dual-Task verwendet.

4.4.5.1 Durchführung

Der Test beginnt im Sitz, auf einem Stuhl mit einer Sitzhöhe von ca. 46 cm. Für die Durchführung des TUGman wir die Zeit gestoppt, die die Proband*innen benötigen, um von einem Stuhl aufzustehen, ein Glas Wasser zu greifen, einen Weg von drei Metern mit dem

Glas in der Hand zu gehen, eine Drehung im Raum mit dem Glas in der Hand zu machen, die drei Meter bis zum Stuhl wieder zurückzugehen, das Glas abzustellen und sich wieder in die Ausgangsposition zu begeben. Die benötigte Zeit wird in Sekunden dokumentiert. Es dürfen Hilfsmittel, welche die Proband*innen momentan benutzen, verwendet werden. Alle verwendeten Hilfsmittel müssen dokumentiert werden. Die Durchführung erfordert 5 Min.

4.4.5.2 Psychometrische Kriterien

Der TUGman weist eine exzellente Inter-Rater Reliabilität (r= 0,98) und Intra-Rater Reliabilität mit einem ICC Wert von 0,99 auf (Hofheinz and Schusterschitz, 2010). Für die Kriteriumsvalidität beschrieben Hofheinz und Schusterschitz (2010) sehr gute Werte im Vergleich mit der BBS (r= -0,72). Bei der prädiktiven Validität bei Personen mit Idiopathischem Parkinsonsyndrom geben Maranhão-Filho und Kolleg*innen (2011) an, dass bei einer Differenz von > 4,5 s zwischen TUG und TUGman ein erhöhtes Sturzrisiko vorliegt. Shumway-Cook und Kolleg*innen (2000) beschreiben eine Prädiktionsrate von 90% bei der Identifikation von älteren Erwachsenen als Stürzende, wenn diese mehr als 14,5 Sekunden für den TUGman benötigen.

4.4.6 Berg Balance Scale (BBS)

Die BBS (Berg et al., 1989) ist ein gängiges Messinstrument in der Physiotherapie, mit dem die Balancefähigkeit von Patient*innen überprüft werden kann.

4.4.6.1 Durchführung

Die BBS umfasst 14 Aktivitäten, welche der Therapeut in Bezug auf die statische und dynamische Balance beobachtet und bewertet. Die Aktivitäten sind an Handlungen des Alltags angelehnt: Transfers vom Sitzen zum Stehen, vom Stehen zum Sitzen und Übersetzen (Item 1, 4 & 5), die statische Balance im Stehen und Sitzen ohne Unterstützung, mit geschlossenen Augen Stehen, mit enger Fußposition stehen, mit beiden Armen nach vorne Reichen, einen Gegenstand vom Boden aufheben, im Stand über die Schulter schauen, im Stand einmal um 360° Drehen, Tandemstand (Ein Fuß direkt vor dem anderen) und Einbeinstand (Item 2, 3, 6, 7, 8, 9, 10, 11, 13 &14) und die dynamische Balance im Stehen indem beiden Füße abwechselnd auf eine Treppenstufe gestellt werden (Item 12) (Scherfer et al., 2006).

Die 14 Items, werden mit den Punkten 0-4 bewertet. Der Wert 0 beschreibt dabei, dass die Patient*innen die Aufgaben nur mit maximaler Hilfe durchführen können. 4 Punkte hingegen bedeuten, dass die Patient*innen die Aufgabe ohne Probleme durchführen können. Es ist also

eine Maximalpunktzahl von 56 Punkten zu erreichen. Berg et al. (Berg et al., 1989) teilen die Patient*innen nach der Auswertung in drei Gruppen ein. Die Punktzahl 0-20 deutet darauf hin, das die Patient*innen einen Rollstuhl benötigen, 21-40 Punkte deuten auf Unterstützung beim Gehen hin und 41-56 Punkte deuten auf ein selbstständiges Gehen hin. Die Zeit der Durchführung beträgt 15-20 Minuten.

4.4.6.2 Psychometrische Kriterien

Der MDC beträgt 5 Punkte (Steffen and Seney, 2008a). Der Cut-Off Wert der BBS für ein erhöhtes Sturzrisiko beträgt ≤52/56 Punkten (Schlenstedt et al., 2016). Die Test-Retest Reliabilität und die Inter-Rater Reliabilität liegt mit ICC Werten von 0,80 und 0,95 in einem sehr guten Bereich (Leddy et al., 2011). Franchignoni und Kolleg*innen eine hohe interne Konsistenz (Cronbach´s Alpha = 0,95) bei 70 Personen mit Idiopathischem Parkinsonsyndrom und einem durchschnittlichen Berg Balance Wert von 46,5 (34-54) Punkten nachweisen (Franchignoni et al., 2005). Für die Kriteriumsvalidität beschreiben Brusse und Kolleg*innen (2005) sehr gute Werte im Vergleich mit dem TUG (r= -0,78) und der UPDRS (r= -0,64).

4.4.7 Four Step Square Test (FSST)

Der Four Step Square Test (FSST) (Dite and Temple, 2002) adressiert das Gleichgewicht und die Fähigkeit vorwärts, seitwärts und rückwärts über Hindernisse zu steigen.

4.4.7.1 Durchführung

Die Proband*innen stehen in einem Quadrat, welches durch ein am Boden liegendes hölzernes Versuchskreuz entsteht. Die Proband*innen werden aufgefordert, einmal im Uhrzeigersinn und danach gegen den Uhrzeigersinn in alle vier Quadrate zu steigen. Hierfür muss ein Schritt nach vorne, zur Seite und nach hinten über das auf dem Boden liegende Versuchskreuz gemacht werden. Es werden immer zwei Durchläufe absolviert, ein Probelauf und ein Durchgang, bei welchem die Zeit gestoppt wird. Die Durchführungszeit beträgt weniger als 5 Minuten.

4.4.8 10-Meter Gehtest

4.4.8.1 Durchführung

Dieser Test der Gehgeschwindigkeit stellt einen wichtigen sekundären Outcome Parameter dar. Er wird von der Parkinson’s Taskforce (PD EDGE) sehr für Patient*innen mit IPD in einem H & Y Stadium 1-3 empfohlen. Für die Überquerung einer Straße mit oder ohne Ampelanlage ist es erforderlich, eine kurze Distanz in möglichst hoher Geschwindigkeit zurückzulegen. Ist

diese Fähigkeit nicht ausreichend vorhanden, sind Aktivitäten im Bereich der Partizipation deutlich eingeschränkt. Es werden bei diesem Test Linien auf dem Boden angebracht, die den Start, 2m, 8m und 10m markieren. Gemessen wird die Zeit zwischen der 2m und 8m Linie, um die Beschleunigungs- und Abbremsphase auszuschließen. Die benötigte Zeit für die Strecke dieser 6m wird auf die hundertstel Sekunde genau aufgeschrieben. Die 6 Meter werden anschließend durch die benötigte Zeit (in Sekunden) geteilt. Daraus ergibt sich die Geschwindigkeit in m/s. Es werden zwei Durchgänge auf diese Weise durchgeführt und daraus der Mittelwert verwendet. Es dürfen Hilfsmittel, die die/ der Patient*in momentan benutzt, verwendet werden. Jegliches verwendetes Hilfsmittel muss dokumentiert werden. Bei der Testanweisung durch die/ den Untersucher*in ist darauf zu achten, dass sich die/ der Untersucher*in nicht vor oder direkt neben der/ dem Patient*in befindet und diese/ diesen bei der Testdurchführung womöglich beeinträchtigt. Die/ der Untersucher*in sollte mindestens in einem halben Meter Abstand hinter der/ dem Patient*in sein.

4.4.8.2 Psychometrische Kriterien

Der Standardmessfehler (Standard Error of Measurement, SEM) wird mit 0,04 m/s und der kleinste klinisch wichtigste Unterschied (Minimal Clinically Important Difference, MCID) bei komfortabler Gehgeschwindigkeit mit 0,18 m/s und bei maximaler Geschwindigkeit mit 0,25 m/s angegeben (Steffen and Seney, 2008). Die Test-Retest Reliabilität des 10MWT ist bei Personen mit PD hervorragend (ICC = 0,96- 0,97) (Steffen and Seney, 2008), ebenso wie die Inter-Rater Reliabilität (ICC=0,93-0,99) (Lindholm et al., 2018). Ein geringer Standardmessfehler von 0,032–0,076 m/s sowie eine hohe prädiktive Validität wurden mit einer Area under the Receiver Operating Characteristic Curve (ROC) von 0,70-0,73 hinsichtlich der Sturzgefahr gezeigt (Lindholm et al., 2018). Cut-off Werte für IPD Patient*innen waren 1,1-1,2 m/s (Lindholm et al., 2018).

4.4.8.3 Psychometrische Kriterien

Duncan and Earhart (2013) evaluierten für Personen mit Idiopathischem Parkinsonsyndrom einen Cut-Off Wert für ein hohes Sturzrisiko bei >9,68 Sekunden. Die Inter-Rater Reliabilität ist mit einem ICC Wert von 0,99 sehr hoch. Für die Kriteriumsvalidität beschreiben Duncan and Earhart (2013) zeigen eine starke Korrelation des FSST im Vergleich mit der UPDRS Skale III (r= 0,61) und eine moderate Korrelation mit dem FOG-Q (r= 0,44).

4.4.9 Parkinson's Disease Questionnaire (PDQ-39)

Der PDQ-39 (Berger et al., 1999; Peto et al., 1995) wird zur Erfassung der gesundheitsbezogenen Lebensqualität verwendet und ist ein sekundärer Endpunkt dieser Studie. Die schriftliche Genehmigung zur Verwendung der deutschen Fassung des PDQ-39 in dieser Studie wurde von Mapi Research Trust eingeholt.

4.4.9.1 Durchführung und Auswertung

Der PDQ-39 ist ein aus 39 Items bestehender Fragebogen zur Selbstevaluation der gesundheitsbezogenen Lebensqualität (Patient Rated Outcome Measure, PROM). Er besteht aus 8 Dimensionen: Mobilität (Item 1 bis 10, max. 40 Punkte), Alltagsaktivitäten (Item 11 bis 16, max. 24 Punkte), emotionales Wohlbefinden (Item 17 bis 22, max. 24 Punkte), Stigma (Item 23 bis 26, max. 16 Punkte), soziale Unterstützung (Item 27 bis 29, max. 12 Punkte), Kognition (Item 30 bis 33, max. 16 Punkte), Kommunikation (Item 34 bis 36, max. 12 Punkte), körperliches Unbehagen (Item 37 bis 39, max. 12 Punkte). Die Auswertung erfolgt anhand einer 5-Punkte Likert Skala, von 0 = „Niemals" bis 4 = „Immer". Ein Umkodieren der Antwortkategorien ist für die Erstellung der Subskalen nicht erforderlich. Alle 8 Domänenscores und der Globale Index werden auf einer 0-100 Skala standardisiert, wobei 0 die beste gesundheitsbezogene LQ bedeutet und 100 die schlechteste. Mapi Trust stellt eine SPSS Syntax zur Auswertung bereit. Der Index kann jeweils nur berechnet werden, wenn der Prozentsatz fehlender Daten für alle Dimensionen ≥50% beträgt. Die Durchführung des PDQ-39 benötigt 10-20 Min.

Die Auswertung erfolgt anhand der Auswertungsanleitung für die deutsche Version des PDQ-39 (Berger et al., 1999).

Subskalen

Die 39 einzelnen Items des PDQ-39 werden zu den folgenden 8 Subskalen addiert: Mobilität, Alltagsaktivitäten, emotionales Wohlbefinden, Stigma, soziale Unterstützung, Kognition, Kommunikation, körperliches Unbehagen. Die Zuordnung der entsprechenden Items zu den 8 Subskalen sind in Tabelle 3 dargestellt.

Tabelle 3: PDQ-39 Subskalen und zugeordnete Items

**Mobilität** (10 Items)<br>Freizeitaktivitäten<br>Haushaltstätigkeiten<br>Einkaufen<br>1 km gehen<br>100 m gehen<br>Im Haus bewegen<br>In der Öffentlichkeit bewegen Begleitperson notwendig Angst vorm Hinfallen<br>Ans Haus gebunden	**Stigma** (4 Items)<br>Krankheit verheimlichen<br>Situationen vermeiden<br>Schämen<br>Sorgen über Reaktionen<br><br>**Soziale Unterstützung** (3 Items)<br>Probleme mit Menschen<br>Unterstützung Ehepartner<br>Unterstützung Freunde
**Alltagsaktivitäten** (6 Items)<br>Probleme beim Waschen<br>Probleme beim Anziehen<br>Probleme beim Knöpfen<br>Undeutliches Schreiben<br>Essen klein schneiden<br>Getränk verschütten<br><br>**Emotionales Wohlbefinden** (6 Items)<br>Deprimiert fühlen<br>Einsamkeit<br>Den Tränen nahe<br>Verärgert sein<br>Ängstlich sein<br>Zukunftssorgen	**Kognition** (4 Items)<br>Tagsüber einschlafen<br>Konzentrationsprobleme<br>Schlechtes Gedächtnis<br>Schlechte Träume<br><br>**Kommunikation** (3 Items)<br>Sprechschwierigkeiten<br>Kommunikationsprobleme<br>Fehlende Beachtung<br><br>**Körperliches Unbehagen** (3 Items)<br>Muskelkrämpfe<br>Gelenkschmerzen<br>Hitze- / Kältegefühl

Die 5 Antwortkategorien erhalten dabei die folgende Kodierung: 0 = niemals, 1 = selten, 2 = manchmal, 3 = häufig, 4 = immer oder kann ich überhaupt nicht. Ein Umkodieren der Antwortkategorien ist für die Erstellung der Subskalen nicht erforderlich.

Für jede Subskala wird zunächst ein Rohwert durch Addition der zugehörigen Einzelitems für jeden Patienten berechnet. Durch eine Transformation der Rohwerte auf eine Skala zwischen 0 (=bester) und 100 (=schlechtester Wert) werden die einzelnen Skalenwerte vergleichbar. Für die Transformation wird folgende Formel benutzt:

$$\underline{\text{(Skalenrohwert x 100)}}$$

maximaler Skalenwert

(Beispiele: Skalenrohwert eines Patienten für ‚Mobilität' = 30 ⇒ 30 x 100 / (4 x 10) = **75**

Skalenrohwert für ‚Stigma' = 4 ⇒ 4 x 100 / (4 x 4)= **25**)

Die durch Transformation erhaltenen Werte entsprechen den PDQ-Werten eines Patienten auf der entsprechenden Subskala. In unserem Beispiel bedeutet ein Wert von 75 für die Subskala ‚Mobilität', dass der Patient einen Wert erreicht hat, der 75 Prozent schlechter als der beste mögliche Wert ist. Entsprechend heißt ein Wert von 25 auf der Skala ‚Stigma', dass dieser Patient einen Wert erreicht hat, der 25 Prozent unter dem besten zu erreichenden Wert liegt.

Missings

Die Behandlung fehlender Antworten (Missings) bedarf besonderer Beachtung. Da sie sich bei Selbstbeantwortung nicht vermeiden lassen wird das folgende Vorgehen empfohlen. Wenn mindestens 50 Prozent der Items einer Subskala beantwortet sind, werden die fehlenden Antworten durch den Mittelwert der vorhandenen Antworten in dieser Skala ersetzt. Daraus ergibt sich, daß in der 10 Item-Skala maximal 5 Fragen ohne Antwort bleiben dürfen, in den 6 Item-Skalen maximal 3, in den 4 Item-Skalen maximal 2 und in den 3 Item-Skalen nur eine Frage. Nach Ersatz der fehlenden Antworten durch die Mittelwerte der vorhandenen innerhalb einer Subskala, werden die Summenscores der acht Subskalen durch Addition und Transformation, wie oben beschrieben, berechnet. Bei Erhebung der PDQ Antworten im Interview, sollten keine Missings vorkommen.

PDQ – 39 Summenscore (PDSI)

Aus den 8 Subskalen läßt sich ein PDQ-39 Summenscore (PDQ-39 SI) berechnen. Er stellt den, nach Anzahl der Items gewichteten, Mittelwert aus den 8 Subskalen dar. Für seine Berechnung werden für jeden Patienten die PDQ-Werte jeder Skala aufaddiert und die Summe durch 8 (=Anzahl der Skalen) dividiert.

[Mobilität + Alltagaktivitäten + Emotionales Wohlbefinden + ... + Körperliches Unbehagen] / 8 = PDQ-39SI

Bei der Interpretation des PDQ-39 SI muß seiner Gewichtung durch die Anzahl der entsprechenden Fragen pro Skala Rechnung getragen werden. Als zusammengesetzter Score ist er in der Regel weniger sensibel gegenüber Veränderungen als die primär interessierenden Subskalen.

4.4.9.2 Psychometrische Kriterien

Alle sieben der acht Dimensionen des PDQ-39 zeigten eine zufriedenstellende diskriminante Validität (Schrag, 2000). Die interne Konsistenz ist exzellent (Cronbach´s Alpha = 0,84-0,94) (Jenkinson et al., 1997). Der Standard Error of Measurement (SEM) wird von XXX für die 8 Dimensionen wie folg angegeben: Mobilität (5,85-11,61), Alltagsaktivitäten (6,96-11,46), emotionales Wohlbefinden (8,26-13,56), Stigma (5,57-13,67), soziale Unterstützung (7,84-17,61), Kognition (8,54-12,34), Kommunikation (6,67-14,95), körperliches Unbehagen (9,94-16,37) (Martinez-Martin et al., 2007). Es wurde eine exzellente konvergente Validität anhand moderater bis starker signifikanter Korrelationen des PDQ-39 mit dem Hoehn & Yahr Stadium (r= 0,60), UPDRS-ME (r= 0,41) und SF-36 (r= 0,34-0,80) festgestellt (Schrag, 2000). Eine zufriedenstellende interne und externe Validität sowie akzeptable Reliabilität wurde für alle acht Dimensionen des PDQ-39 festgestellt (Jenkinson et al., 1997). Laut Jenkinson und Kolleg*innen (1997) stellt der PDQ-39 den Einfluss der LQ bei Personen mit Idiopathischem Parkinsonsyndrom besser da als generische Messinstrumente wie das Short Form-36 Health Survey (SF-36).

4.4.10 Freezing of Gait Questionnaire (FOG-Q)

Der Freezing of Gait Questionnaire (Giladi et al., 2000) ist ein valides Instrument zur Beurteilung des FOG bei Personen mit Idiopathischem Parkinsonsyndrom ohne Demenz. Für den deutschsprachigen Raum wurde der FOG-Q 2015 validiert (Vogler et al., 2015). Er wird zur Quantifizierung des FOG eingesetzt. Durch seine kurze Durchführzeit und eine hohe Praktikabilität ist der FOG-Q sehr gut zur Beurteilung der FOG in der klinischen Praxis geeignet.

4.4.10.1 Durchführung

Der FOG-Q ist ein PROM zur Evaluation des FOG. Er enthält 6 Items, welche von den Personen mit Idiopathischem Parkinsonsyndrom oder, falls nötig, als Interview von den zuständigen Ärzten/Ärztinnen oder Therapeut*innen erhoben wird. Die Auswertung erfolgt anhand einer 4-Punkte Likert Skala, von 0= „Normal“ bis 4= „Gehunfähig“, wobei höhere Scores eine stärker ausgeprägtes Freezing repräsentieren. Die Fragen adressieren, ob ein FOG vorhanden ist, ob das tägliche Leben dadurch beeinträchtigt wird und ob ein Freezing beim Losgehen oder Drehen auftritt. Die Durchführung erfordert 5-10 min.

4.4.10.2 Psychometrische Kriterien

Die Konstruktvalidität der deutschen Version des FOG-Q wurde bei 27 Personen mit Idiopathischem Parkinsonsyndrom (Hoehn & Yahr II-IV) mit FOG geprüft. Der FOG-Q weist eine gute interne Konsistenz auf (Cronbach´s a: 0,83). Es wurde eine signifikante Korrelation zwischen dem Item Freezing (2.13) und der MDS-UPDRS (p=0,002) und der Mobilität bzw. Alltagsaktivitäten des PDQ-39 (p=0,006 bzw. p= 0,035) nachgewiesen. Die Ergebnisse zeigten keinen Zusammenhang zwischen dem FOG-Q und der MDS-UPDRS-Subskala I (p= 0,079), was auf eine gute divergente Validität hinweist (Vogler et al., 2015).

4.4.11 Falls Efficacy Scale- International Version (FES-I)

Eine Vielzahl von älteren Menschen weisen psychologische Probleme auf, die mit Stürzen in Zusammenhang stehen. Dies gilt sowohl bei Personen, welche bereits gestürzt sind als auch bei Personen, die noch nicht gestürzt sind. Um diese Probleme adressieren zu können bedarf es einer Erhebung der Sturzangst und der Selbstwirksamkeit in Bezug auf das Stürzen. Die Falls Efficacy Scale (Tinetti et al., 1990) wurde 2005 von einem Experten-Netzwerk zur Sturzprävention (Prevention of Falls Network Europe, ProFaNE) auf eine Internationale Version (FES-I) erweitert (Yardley et al., 2005; Skelton et al., 2004). Der PROM dient der Erfassung der sturzassoziierten Selbstwirksamkeit bei älteren Menschen. Mit der Erweiterung wurden zusätzlich komplexere, funktionelle Aktivitäten und soziale Aspekte der Selbstwirksamkeit aufgenommen.

4.4.11.1 Durchführung

Die FES-I besteht aus 16 Items, welche die Bedenken in Bezug auf Stürze und verschiedene Aktivitäten bei den bei den Proband*innen evaluieren. Der Fragebogen kann als Selbstauskunftsbogen oder auch als strukturiertes Interview durchgeführt werden. Die Antworten werden anhand einer 4-Punkt Likert-Skala von 1= „keinerlei Bedenken“ zu 4= „sehr große Bedenken“ eingeordnet. Die Dauer des Fragebogens beträgt 10 Minuten.

4.4.11.2 Psychometrische Kriterien

Im Zuge der Weiterentwicklung des FES zu dem FES-I durch das Experten-Netzwerk zur Sturzprävention (Prevention of Falls Network Europe ProFaNE), wurde eine deutsche Übersetzung vorgenommen. Jedoch liegt eine Validierung bisher nur für die englische Version vor (Skelton et al., 2004). Die Ergebnisse der englischen Version zeigen eine hohe Retest Reliabilität für den Gesamtscore (r= 0,96) und eine hohe interne Konsistenz (Cronbach´s

Alpha= 0,96). Weiterhin betrug die Item-Interkorrelation im Mittel r= 0,55 (Range r= 0,29-0,79) (Dias et al., 2006).

4.4.12 Beck Depression Inventory (BDI-II)

Der BDI-II (Beck et al., 1988) wird zur Erfassung des Schweregrads der Depression verwendet und ist ein sekundärer Endpunkt dieser Studie. Die schriftliche Genehmigung zur Verwendung der deutschen Fassung des BBD-II in dieser Studie wurde vom Mapi Research Trust eingeholt (kostenpflichtig; https://mapi-trust.org/).

4.4.12.1 Durchführung

Der BDI-II besteht aus 21 Items und ist ein PROM zur Erfassung des Schweregrad der Depression. Die Auswertung der Items erfolgt anhand einer 4-Punkt Likert Skala von 0 bis 3. Dadurch kann die Summe der Items 0-63 Punkte betragen. Ab einer Punktzahl größer als 10 kann eine Depression identifiziert werden. Die Skala ist ab einem Alter von 13 Jahren validiert. Die Dauer des Fragebogens beträgt 5-10 Minuten.

4.4.12.2 Psychometrische Kriterien

Die interne Konsistenz ist exzellent (Cronbach´s Alpha= 0,89-0,93). Die Reliabilität liegt in einer Stichprobe depressiver Proband*innen bei 0,92. Die Reliabilität für die Gesunden liegt bei 0,80 bzw. 0,82. Die Retest Reliabilität ist für Gesunde als hinreichend stabil anzusehen, da sie über drei Wochen bzw. fünf Monate 0,78 beträgt. Die Retest Reliabilität liegt für Proband*innen mit einem Wert von 0,46 niedriger. Die Kriterumsvalidität in Bezug auf die Korrelation mit der Lebensqualität zeigt Werte zwischen 0,50 und 0,75 auf (Kühner et al., 2007).

Für Proband*innen mit Idiopathischem Parkinsonsyndrom konnte eine Test-Retest Reabilität mit einem ICC Wert von 0,89 ermittelt werden (Visser et al., 2006). Für Personen mit psychiatrischen Erkrankungen konnten folgende Cut-Off Werte bestimmt werden: Eine minimale Depression wird ab einem Wert von 10,9 (SD=8,1) Punkten identifiziert, leichte Depression ab einem Wert von 18,7 (SD=10,2) Punkten, ab einem Wert von 25,4 (SD=9,6) Punkten eine moderate Depression, eine schwere Depression liegt ab einem Wert von 30,00 (SD=10,4) Punkten vor (Beck et al., 1988).

4.4.13 Sturzrate

Es erfolgt eine Erhebung der Anzahl der Stürze

(a) im 3 Monatszeitraum vor Studienbeginn (subjektiv erfragt zu Baseline)
(b) im 4-wöchigen Interventionszeitraum (beobachtet und subjektiv erfragt zu Postintervention)
(c) im 4-Wochen Follow-up Zeitraum (subjektiv erfragt während des Follow-up Anrufs)

Die Anzahl der Stürze wird im CRF dokumentiert.

4.4.14 Machbarkeit einer größeren Studie

4.4.14.1 Machbarkeitskriterien

Die Machbarkeitskriterien für eine größere Studie sind wie folgt definiert:

a) eine Ziel-Rekrutierungsrate von 40% von 85 geeigneten PatientInnen (oder 4-5 TeilnehmerInnen pro Monat); die Gesamtzahl von geeigneten Idiopathischem Parkinsonsyndrom Patient*innen wurde basierend auf der Anzahl der am RZM behandelten geeigneten Patient*innen mit Idiopathischem Parkinsonsyndrom der letzten 3 Jahre geschätzt.

(b) eine Ziel-Retentionsrate von 80%,

(c) eine minimale Ziel-Adhärenzrate von 75% (mindestens 3 von angestrebten 4 Übungssitzungen pro Woche; dokumentiert im CRF)

(d) hohe Sicherheit der Intervention, keine schweren Nebenwirkungen bzw. nur sehr leichte und vorübergehende Nebenwirkungen (kontinuierlich erhoben mittels UE Log im CRF; siehe auch Kapitel 4.5.2)

(e) hohe Akzeptanz der Intervention, kontinuierlich während des Interventionszeitraums narrativ evaluiert tsowie durch Fokusgruppen mit semistrukturierten Fragen (siehe Kapitel 4.4.14 und CRF für eine detaillierte Beschreibung der Fokusgruppen sowie gestellten Fragen)

4.4.14.2 Beurteilung der Sicherheit und Nebenwirkungen

Zur Beurteilung der Sicherheit der Teilnehmenden werden unten angeführte subjektive und objektive Sicherheitsparameter erhoben. Subjektive Parameter sind jene, die durch die Teilnehmenden berichtet oder durch die Therapeut*innen erfragt werden. Beispiele dafür sind allgemeines Wohlbefinden, Belastungszeichen, etc. Die ständige Rücksprache mit den Teilnehmenden bezüglich ebendieser Parameter ist ein Standardprozedere in der Therapie von Personen mit Idiopathischem Parkinsonsyndrom und dient der individuellen Anpassung der Therapieintensität. Objektive Parameter zur Beurteilung der Sicherheit sind jene

schwerwiegenden oder nicht-schwerwiegenden unerwünschten Nebenwirkungen, die im Rahmen der Studie aufgezeichnet und ausgewertet werden (siehe Kapitel 4.5).

Verträglichkeit/ Sicherheitsparameter

- Subjektiv durch den Teilnehmenden:
  Durch enge Absprache und Rückmeldung mit dem/r behandelnden Arzt/Ärztin und Therapeut*in
  (→ anpassen der Therapie, wie es die Situation erfordert beziehungsweise für den Teilnehmenden möglich ist – gewohntes Vorgehen in jedem Therapiealltag)
- Objektive Parameter:
  Zur Objektivierung der Sicherheit dient die Erfassung und Auswertung von Nebenwirkungen (siehe Kapitel 4.5.2).

Es erfolgt eine kontinuierliche Überwachung und Einschätzung der Sicherheit der Intervention zur Erhebung von Nebenwirkungen, „adverse events". Die detaillierte Vorgehensweise ist im Kapitel 4.5.2 beschrieben.

4.4.14.3 Akzeptanz der Intervention: Fokusgruppen

Eine Fokusgruppe ist eine Form des Gruppeninterviews, welche sich auf die Kommunikation zwischen den Teilnehmenden fokussiert (Stalmeijer et al., 2014). Durch diese Art der Datensammlung werden nicht nur die Gedanken und Gefühle der Teilnehmenden erhoben, sondern auch vor welchem Hintergrund diese entstanden sind. Ein Ziel der Fokusgruppe ist das Aufnehmen, Verstehen und Erklären der Bedeutung des Glaubens und der Kultur, welche die Gefühle, das Verhalten und die Einstellung der Teilnehmenden beeinflussten (Rabiee, 2004). Fokusgruppen eignen sich besonders gut für Bereiche, welche bisher nur wenig oder schlecht erforscht sind (Kitzinger, 1995). Sie können zudem genutzt werden, um schon vorhandene Daten zu bestätigen bzw. solche weiter zu unterstützen (Stewart et al., 2007).

Die in den Fokusgruppen zur Diskussion gestellten Fragen sind im CRF aufgelistet.

4.4.15 Messung der Augenbewegungen während der Intervention

Einmal wöchentlich, jeweils bei der letzten Therapiesitzung, werden mittels Tobii Pro Glasses 3 für 12 min die Augenbewegungen gemessen: Sakkadengeschwindigkeit, -Amplitude, -Latenzzeit und Fixationsdauer. Diese Messung wird nach einem 5-minütigen Aufwärmen durchgeführt (Aufwärmen: siehe Instruktor Manual, Anhang 1).

Der 12-Minuten Zeitraum wurde gewählt, da zu erwarten ist, dass die Teilnehmenden eine Gewöhnungsphase an die Tobii Gläser von etwa 5-6 Minuten haben werden (Kommunikation mit Fa. Tobii). Die gewonnenen Daten sind damit zuverlässiger und realitätsnaher. Die Datenerfassung wird während der Durchführung standardisierter Übungen erfolgen, um einen Vergleich zu ermöglichen: Übung 2, 3, 4, 6 und 7.

4.5 Unerwünschte Ereignisse, Nebenwirkungen

4.5.1 Definitionen

Die Durchführung der Testungen und Interventionen (Therapie der Interventionsgruppen) können unerwünschte Ereignisse und Nebenwirkungen auslösen. Während der Therapie können sowohl in der Interventionsgruppe als auch in der Kontrollgruppe Ereignisse wie Schwindel, Kurzatmigkeit oder ähnliche Anzeichen von Belastung als physiologische Reaktion auf Belastung (das Training) auftreten. In der Interventionsgruppe kann eine leichte Augenmüdigkeit auftreten, was aber durch kurze Trainingssequenzen, variable Übungen sowie Pausen und Übungen ohne Blickbewegungstraining gut kontrolliert werden kann.

4.5.1.1 Unerwünschtes Ereignis (UE)

Bei einem unerwünschten Ereignis (UE) handelt es sich um jedes nachteilige Vorkommnis, das einem/r Proband*in während der Teilnahme an dieser Studie widerfährt, und das nicht notwendigerweise in ursächlichem Zusammenhang mit der Intervention steht. Dies können Erkrankungen, Krankheitszeichen und Symptome sein, die nach Einschluss des/ Proband*in in diese Studie auftreten oder vorbestehende Erkrankungen, Krankheitszeichen und Symptome, die sich nach Einschluss in diese Studie verschlechtern.

Ausgenommen sind Vorerkrankungen, die sich im Studienverlauf nicht verschlechtern sowie unerwünschte Ereignisse, die im Zusammenhang mit einer Begleitmedikation stehen.

4.5.1.2 Nebenwirkung (NW)

Eine Nebenwirkung (NW) ist jedes oben definierte unerwünschte Ereignis, bei dem eine Kausalität zur Studienintervention vorliegt.

4.5.1.3 Schwerwiegendes Vorkommnis

Als schwerwiegendes Vorkommnis werden jedes unerwünschte Ereignis bzw. jede Nebenwirkung bezeichnet, die entweder

- Zum Tode führt, oder

- Lebensbedrohlich ist, oder
- Zu einer bleibenden oder schwerwiegenden Behinderung oder Invalidität führt, oder
- Eine stationäre Behandlung oder deren Verlängerung erforderlich macht (außer die stationäre Behandlung war bereits vor der Teilnahme an dieser Studie geplant).

Je nach Kausalität zur Studienintervention unterscheidet man zwischen einem schwerwiegenden unerwünschten Ereignis (SUE) oder einer schwerwiegenden Nebenwirkung (SNW).

4.5.1.4 Schweregrad

Die Schweregrade der Vorkommnisse ((S)UE und (S)NW) werden folgendermaßen definiert:

- Mild – Die Intervention muss aufgrund des Vorkommnisses unterbrochen werden, kann aber am selben oder am darauffolgenden Tag weitergeführt werden. Die Konsultation eines/r Arztes/Ärztin zur Behandlung ist hierbei nicht notwendig;
- Moderat – Die Intervention muss unterbrochen werden, kann an einem anderen Tag, aber nicht unmittelbar am darauffolgenden Tag weitergeführt werden. Die Konsultation eines/r Arztes/Ärztin zur Behandlung ist notwendig.
- Schwer – Die Intervention muss abgebrochen werden und kann aufgrund des Ereignisses nicht weitergeführt werden (im Sinne eines Drop-outs gemäß Kapitel 5.4)

4.5.1.5 Kausalität

Die Kausalität wir folgendermaßen definiert:

- Therapieassoziiert – Es ist entweder bekannt, dass das unerwünschte Vorkommnis durch die Studienintervention verursacht werden kann oder es besteht der begründete Verdacht, dass die Studienintervention das unerwünschte Vorkommnis verursacht hat, oder es besteht ein zeitlicher Zusammenhang zwischen der Studienintervention und dem Auftreten des unerwünschten Vorkommnisses.

- Nicht-Therapieassoziiert – Es gibt keinen begründeten Verdacht, dass das unerwünschte Vorkommnis durch die Studienintervention verursacht wurde, oder es besteht kein zeitlicher Zusammenhang zwischen der Studienintervention und dem Auftreten des unerwünschten Vorkommnisses.

4.5.2 Beurteilung und Dokumentation

Im Rahmen dieser Studie werden alle Unerwünschten Ereignisse sowie Nebenwirkungen systematisch erfasst, bewertet und am Ende der Studie mit ausgewertet.

4.5.2.1.1 Beurteilungsprozess

Ein/e autorisierter Arzt/Ärztin muss die mögliche Assoziation zur Intervention, den Schweregrad (mild, moderat, schwer) sowie die Einstufung des Vorkommnisses als schwerwiegend bzw. nicht-schwerwiegend evaluieren und bestätigen.

4.5.2.1.2 Dokumentation von unerwünschten Vorkommnissen (UE/NW)

Während der Interventionsphase (Woche 1 – Woche 4) wird das Auftreten der o.g. Vorkommnisse durch die verantwortlichen Mitarbeitenden am Studienzentrum erfasst und durch ein Mitglied des ärztlichen Teams beurteilt.

Alle Vorkommnisse und Befunde müssen in der Patientenakte und anschließend im UE-Log des CRF dokumentiert werden.

Folgende Angaben sind nötig:

- Art des Unerwünschten Ereignisses (Anzeichen, Symptom oder Krankheit)
- Beginn und Ende des Auftretens
- Schweregrad
- Kausalität zur Intervention
- Differenzierung (schwerwiegend/nicht schwerwiegend)
- Maßnahmen hinsichtlich der Intervention oder Handlungen zur Wiederherstellung oder Besserung des Wohlbefindens des/r Patient*in
- Ausgang des Ereignisses.

4.5.2.1.3 Dokumentation von schwerwiegenden Vorkommnissen (SUE/SNW)

Schwerwiegende Vorkommnisse müssen zusätzlich zur Dokumentation im UE-Log des CRF in das Formblatt zur Meldung schwerwiegender Vorkommnisse eingetragen werden.

Bei der Dokumentation der schwerwiegenden Vorkommnisse sind folgende Punkte strikt einzuhalten:

- Jedes SUE/SNW muss so vollständig wie möglich gemeldet werden.
- Befunde sind der Meldung pseudonymisiert (unter Verwendung des Codes der Patient*innen) beizulegen

Der Prüfarzt oder die Prüfärztin des jeweiligen Prüfzentrums muss das Meldeformular für schwerwiegende Vorkommnisse auf Vollständigkeit überprüfen und sicherstellen, dass die

eingetragenen Informationen mit denjenigen im UE-Log des CRF und weiteren Datenquellen übereinstimmen.

4.5.3 Meldung von schwerwiegenden Vorkommnissen (SUE/SNW)

4.5.3.1 Mitteilungspflichten des Prüfenden

Alle während der Studie auftretenden schwerwiegenden Vorkommnisse müssen unverzüglich nach Kenntnisnahme (≤ 24 Stunden/1 Arbeitstag) durch Übermittlung des Formblattes für schwerwiegende Vorkommnisse an den Koordinierenden Prüfarzt und Sponsor (E-Mail: christian.brenneis@reha-muenster.at) gemeldet werden.

Zu beachten: Personenbezogene Daten (z.B. Befunde) sind vor ihrer Übermittlung (DSVO-konform: persönlich, Analogfax) unter Verwendung des Codes der Patient*innen zu pseudonymisieren.

4.5.3.2 Mitteilungspflichten des Sponsors

Der Sponsor hat die Ethikkommission der Medizinischen Universität Innsbruck über alle schwerwiegenden Vorkommnisse, die während des Verlaufes dieser klinischen Studie auftreten unverzüglich nach Bekanntwerden (≤ 24 Stunden/1 Arbeitstag) schriftlich zu informieren.

Bei (nicht erwarteten) Vorkommnissen, die möglicherweise zu einer Erhöhung des Risikos führen, ist die Ethikkommission neu zu befassen.

5 Datendokumentation, -Management und -Auswertung

5.1 Methoden der Datensammlung

Die Datenerhebung zur Auswertung der Endpunkte wird ausschließlich mit validen, reliablen und responsiven Instrumenten patientenorientiert erhoben, welche in Kapitel 4.4 gelistet und beschrieben sind.

5.2 Dokumentation

5.2.1 Quelldaten und -dokumente

Als Quelldaten werden alle Informationen aus originalen Dokumenten und Berichte sowie deren zertifizierten Kopien angesehen, die zur Rekonstruktion und Beurteilung der Studie notwendig sind. Quelldaten sind in Quelldokumenten enthalten.

Quelldokumente in dieser Studie sind:

z.B. Krankenakten, Einwilligungserklärungen, Therapiedokumentationen, etc.

5.2.2 Erhebungsbogen (CRF)

Sämtliche Daten der Patient*innen und Untersuchungsergebnisse werden in die speziell für diese Studie erstellten CRFs (Case Report Forms) eingetragen.

Die Erhebungsbögen dürfen nur mit einem **Kugelschreiber oder Fineliner (schwarz oder blau)** ausgefüllt werden. Korrekturen sind so vorzunehmen, dass die alte Eintragung lesbar bleibt (das Verwenden von Korrekturmitteln ist nicht erlaubt). Korrekturen müssen von der autorisierten Person, die diese vornimmt, signiert und datiert werden. Daten, die nicht verfügbar sind oder die nicht erhoben wurden, müssen als solche klar erkenntlich sein (F oder ND). Die Gründe dafür sollten ggf. dokumentiert werden.

Der Prüfer stellt sicher, dass sämtliche Daten der Patienten unverzüglich, leserlich, vollständig, korrekt und in Übereinstimmung mit den Patientenakten in die CRFs eingetragen werden.

Die ausgefüllten Originalseiten werden den zuständigen Personen zur Dateneingabe und Auswertung der Daten zugestellt.

5.3 Weiterbehandlung der Patient*innen nach Abschluss der Prüfung

Die Patient*innen unterliegen dem regulären Versorgungsschema im Rahmen ihrer Behandlung des Idiopathischen Parkinsonsyndroms, nach welchem auch die Weiterversorgung nach Abschluss der Studie erfolgt.

5.4 Ausscheiden von Patient*innen (Drop-out)

Patienten können jederzeit auf eigenen Wunsch vorzeitig und ohne Angabe von Gründen sowie ohne Folgen für ihre zukünftige Behandlung aus der klinischen Studie ausscheiden. Außerdem können Patienten durch den Prüfer aus Gründen der Gesundheitsgefährdung aus der Studie ausgeschlossen werden.

Folgende Punkte bzw. Ereignisse führen nach initialer Aufnahme der Versuchsperson in die Studie ebenfalls zum Ausscheiden derselben (im Sinne einer Einstufung als Drop-out für die biometrische Analyse):

- Keine Fragebogen-Scores oder Assessment-Scores von interpretierbarer Qualität.
- Zurückziehen der Einwilligung zur Studienteilnahme durch den Studienteilnehmer innerhalb dessen Studienintegration.
- Jegliche Veränderung bzw. Anpassung der dopaminergen Medikation, die laut Entscheidung des Prüfarztes nicht mehr mit den Studienzielen zu vereinbaren ist – siehe Ausschlusskriterien.

Kommt es nach Beginn der Studienintervention zu einer Gesundheitsgefährdung des/r Patient*in aufgrund einer unerwarteten Verschlechterung des Gesundheitszustandes oder des Allgemeinbefindens und/oder einer absoluten Kontraindikation gegen körperliches Training, wird die Teilnahme an der Studie unverzüglich beendet und ein Facharzt herangezogen.

Der Grund für das Ausscheiden des/r Patient*in aus der Studie wird im CRF dokumentiert. Die Studie ist mit der nächsten Versuchspersonennummer fortzusetzen.
Alle vorzeitig aus der Studie ausscheidenden Patient*innen werden gebeten, sich -falls im Rehabilitationszeitraum und gesundheitlich möglich- zum nächstmöglichen Zeitpunkt einer Abschlussuntersuchung i.S. der Postintervention Testung zu unterziehen, deren Ergebnisse im CRF dokumentiert werden.

5.5 Vorzeitiger Abbruch der klinischen Prüfung

Der Sponsor ist berechtigt, die Studie aufgrund relevanter medizinischer/administrativer Ursachen vorzeitig zu beenden. Die Gründe für die Einstellung der Studie werden detailliert dokumentiert. Patient*innen, die zum Zeitpunkt des Abbruchs der Prüfung noch in Behandlung stehen, werden gebeten, sich zum nächstmöglichen Zeitpunkt einer Abschlussuntersuchung i.S. der Postintervention Testung zu unterziehen, die im CRF dokumentiert wird. Bestehen bei

einem Prüfer ethische Bedenken bezüglich der Weiterführung der Studie, so muss dies unverzüglich dem Sponsor angezeigt werden.

Der Sponsor ist berechtigt, die klinische Prüfung vorzeitig zu beenden, wenn

- die Teilnehmerrekrutierungsrate unzulänglich ist,
- gravierende, nicht zu klärende Probleme mit der Qualität der erhobenen Daten auftreten,
- unvorhersehbare Umstände im jeweiligen Prüfzentrum eingetreten sind, die eine Weiterführung der klinischen Studie nicht zulassen,
- unvertretbare Risiken aufgetreten sind (Entscheidung nach erfolgter neuer Nutzen-Risiko-Abwägung),
- neue wissenschaftliche Erkenntnisse während der Laufzeit der Studie die Weiterführung der Studie nicht erlauben,

Über den Abbruch der Studie kann die Studienleitung in Abstimmung mit dem Sponsor entscheiden.

Bestehen bei einem Prüfer ethische Bedenken bezüglich der Weiterführung der Studie, so muss dies unverzüglich dem Sponsor angezeigt werden.

5.6 Datenanalyse

5.6.1 Statistische Datenanalyse

Die statistische Datenanalyse erfolgt mit IBM SPSS Software, Version 26.0 (IBM Corporation, Armonk, NY, USA) und Tobii Pro Lab Analyer (Tobii, Danderyd, Sweden). Das statistische Signifikanzniveau ist durch einen p-Wert von <0,05 definiert. Es wird versucht, fehlende Daten zu vermeiden, indem die Fragebögen nach dem Ausfüllen inspiziert und im Falle von unbeantworteten Items die Studienteilnehmer*nnen gebeten werden, diese zu komplettieren. Die Anzahl fehlender Daten wird notiert; aufgrund des wenig sensitiven Untersuchungsthemas wird angenommen, dass fehlende Variablenwerte „Missing completely at random“ bzw. „Missing at random“ zugeordnet werden können. Eine Intention-to-treat Analyse wird für alle Fälle durchgeführt, die in der ihnen ursprünglich zugewiesenen Gruppe analysiert werden.

Deskriptive Statistik wird für die demografischen Daten sowie primären und sekundären Outcomes verwendet. Gezählte und nominalskalierte Daten (Geschlecht, Sturzrate, Rekrutierungs-, Retentions- und Adhärenzrate, ggf. Anzahl fehlende Daten, Anzahl unerwünschte Ereignisse) werden als ganze Zahlen bzw. Prozentsätze angegeben, ordinalskalierte Variablen (MMSE,

UPDRS, H & Y, FGA, BBS, PDQ-39, FOGQ, FES-I, BDI-II) mittels des Medians (Minimum, Maximum bzw. Interquartilsabstand) sowie metrische Variablen (TUG, TUGman, FSST, 10MWT, Augenbewegungen: Sakkadengeschwindigkeit, -Amplitude, -Latenzzeit, Fixationsdauer) durch Mittelwert (95% Konfidenzintervall, CI bzw. Standardabweichung, SD). Die Darstellung der Daten erfolgt auch grafisch (z.B. durch Balkendiagramme, Box-Plot oder Liniendiagramme).

Die Eignungsrate (%) ist der Prozentsatz der für die Studie geeigneten PatientInnen nach Anwendung der Ein- und Ausschlusskriterien aus der im Studienzeitraum am Reha Zentrum Münster behandelten Parkinsonpatient*innen.

Die Rekrutierungsrate (%) wird folgendermaßen bestimmt: $(N_{Consent}/N_{geeignet})*100$; dabei ist $N_{consent}$ die Anzahl der TeilnehmerInnen, welche die informierte Einwilligungserklärung unterschrieben haben; $N_{geeignet}$ ist die Anzahl der anhand der für die Studie unter Anwendung der Ein- und Ausschlusskriterien geeigneten Patient*nnen.

Die Retentionsrate (%) wird folgendermaßen berechnet: $(N_{komplettiert}/N_{gesamt})*100$; dabei ist $N_{komplettiert}$ die Anzahl der Teilnehmer*nnen, welche die Studie abgeschlossen haben; N_{gesamt} ist die Gesamtanzahl der StudienteilnehmerInnen.

Die Adhärenzrate (%) wird wie folgt bestimmt: (Tatsächlich durchgeführte Anzahl an Übungssitzungen/geplante Anzahl an Trainingssitzungen)*100 (Osterberg & Blaschke, 2005).

Die Eignungsrate, Rekrutierungs- und Adhärenzraten werden anhand der von Newcombe propagierten Wilson ‚Score'-Methode samt ihres 95%-igen CI berechnet (Newcombe, 1998); im Fall einer Proportion nahe 0 oder 1 wird eine Poisson Approximation nach Brown verwendet (Brown, Cai, & DasGupta, 2001).

Es erfolgt eine Überprüfung statistisch signifikanter Unterschiede zwischen den Gruppen zu Baseline: für nominale Daten wird der Fisher's Exact Test verwendet, für ordinale Daten der Mann Whitney-U Test und für metrische Daten der T-Test für unabhängige Stichproben. Eine präliminäre inferentielle statistische Analyse zur Detektion von Trends hinsichtlich der Effektivität der Intervention zu erkennen und als Basis für eine Stichprobenberechnung für eine randomisierte kontrollierte Studie mit ausreichender Power von mindestens 80% (anhand der Mittelwertunterschiede (SD) zwischen den Gruppen) wird durchgeführt. Für die inferentielle Statistik werden zunächst metrische Daten mittels Shapiro-Wilk Test, Q-Q Plots und Histogrammen auf Normalverteilung sowie auf signifikante Datenausreißer getestet. Nicht normalverteilte Daten werden mittels geeigneter Transformation (z.B. ln, Quadratwurzel) transformiert, erneut überprüft und bei fehlender Normalverteilung nicht-parametrisch analysiert.

Unterschiede zwischen den beiden Gruppen und Testzeitpunkten werden für ordinale Daten folgendermaßen berechnet: Aus der Differenz der Postintervention- und Baselinedaten werden neue Variablen generiert. Der Unterschied zwischen Gruppe 1 und 2 wird mittels Mann Whitney-U Test bestimmt. Für die ordinalen Variablen FES-I und FOGQ, die auch beim Follow-up Anruf erhoben werden, wird über alle Gruppen und Testzeitpunkte hinweg ein Kruskal Wallis Test durchgeführt. Veränderungen der Sturzrate (nominal) zwischen Baseline, Postintervention und Follow-up werden mittels Chi-Square Test berechnet. Metrische Daten werden auf Homogenität der Varianz (Levene Test) als Grundannahme eines 2 x 2 ANOVA überprüft, mit „Zeit (Baseline, Postintervention)“ als „within-factor“ und „Gruppe (1, 2)“ als „between-factor“. Sollte keine Homogenität der Varianz vorliegen, wird eine alternative F-Statistik (z.B. nach Welch oder Brown-Forsythe) verwendet. Bei Nichterfüllen der Grundannahme der Sphärizität werden korrigierte Werte z.B. nach Greenhouse-Geisser oder Huynh-Feldt verwendet. Zur Berechnung der Unterschiede zwischen den Gruppen und Messzeitpunkten ein 2 x 2 ANOVA (Varianzanalyse) durchgeführt, bei vorliegender statistischer Signifikanz gefolgt von paarweisen Vergleichen (Bonferroni Korrektur für alle Vergleiche) zwischen den Gruppen zu den Messzeitpunkten 1 und 2. Im Fall des Versagens der adaptiven Maßnahmen werden nicht parametrische Tests eingesetzt wie für ordinalskalierte Daten beschrieben.

Die Effektstärke wird mittels partiellem Eta Quadrat und standardisierter Effektstärke angegeben (Cohen’s d).

5.6.2 Qualitative Datenanalyse

Die Interviewdaten hinsichtlich der Akzeptanz der Intervention werden mit einer Qualitativen Inhaltsanalyse analysiert (Berelson, 1952; Lasswell, 1948; Mayring, 2003). Der Vorteil dieser Methode ist die Möglichkeit einer anfänglich qualitativen und anschließend quantitativen Datenanalyse. beinhaltet folgende Arbeitsschritte (Bryman, 2012, 2007; Denzin et al. 2018; Schreier, 2012):

- Wortgetreue Transkription der semistrukturierten Interviews (f4 Transkript Software)
- Ausarbeiten einer Liste von Themen und objektiven Kategorien (unabhängig, vollständig, sich gegenseitig ausschließend und adäquat, um die Forschungsfragen zu beantworten)
- Entwicklung eines Codierrahmens
- Markierung und Segmentierung der Textabschnitte anhand thematischer Kriterien
- Markierung des Inhalts zur Identifikation von Hauptkategorien
- Progressive Summierung der Daten durch Gruppierung der Codes in Subkategorien, Klassifizierung und Gegenüberstellung
- Doppelcodierung des Datensatzes nach 10-14 Tagen (Reliabilitätstestung)

- Veranschaulichung des Inhalts der Haupt- und Subkategorien durch Zitate
- Erstellen einer Datenmatrix, auch quantitative Analyse der Daten (Häufigkeiten, deskriptive Statistik)
- Erstellung des Reports

5.7 Prüfordner und Datenmanagement

Im Prüfordner werden diejenigen Dokumente aufbewahrt, die für die klinische Prüfung erforderlich sind. Von Seiten des Sponsors und Prüfers sind geeignete Maßnahmen für eine sorgfältige und vertrauliche Handhabung aller im Rahmen einer klinischen Prüfung anfallenden Daten zu setzen.

5.7.1 Aufbewahrung d. Studienunterlagen, Datenspeicherung & -löschung

Aufzeichnungen und Dokumente, die im Zusammenhang mit der Prüfung stehen (z. B. Einwilligungserklärungen, Erhebungsbögen, und andere relevante Unterlagen) müssen von den Prüfern mindestens 15 Jahre nach Ende oder Abbruch der klinischen Prüfung aufbewahrt werden. Der Prüfer sowie der Koordinierende Prüfer trägt dafür Sorge, dass die Unterlagen betreffend die Pseudonymisierung für einen Zeitraum von 15 Jahren nach Abschluss oder Abbruch der klinischen Prüfung aufbewahrt werden. Die prüfungsrelevanten Daten, Unterlagen und Dokumente werden in gesicherten Räumlichkeiten und auf gesicherten Servern aufbewahrt. Die Krankenblätter und andere Originaldaten müssen über den längst möglichen Zeitraum, den die Institution gestattet, aufbewahrt werden. Der Zugang zu den Dokumenten muss auf die Mitarbeiter des Studienteams beschränkt sein. Nach Ablauf der 15-Jahres Frist werden die Unterlagen vernichtet.

5.7.2 Datenmanagement

5.7.2.1 Dateneingabe

Für die statistische Analyse werden die Daten aus Fragebogen und Erhebungsprotokollen zusammengetragen und durch eine autorisierte und geschulte Person in den Datenerhebungsbogen (CRF) für jeden Messzeitpunkt (Baseline, Postinterventions Testung, Fokusgruppen (siehe Qualitative Datenanalyse), Follow-up Anrufe) eingetragen. Im CRF werden nur Daten erhoben, die im Prüfplan angeführt sind, und die zur Interpretation der Studienergebnisse benötigt werden. Anschließend werden die Daten durch zwei Studienmitarbeiter unabhängig voneinander in die Excel Datenbank eingepflegt, von der aus sie in das Statistikprogramm eingespielt werden können.

5.7.2.2 Qualitätssichernde Maßnahmen

Zur Sicherstellung der Plausibilität und Integrität der Daten werden die folgenden Maßnahmen durchgeführt:

- Dokumentation von Protokollabweichungen und deren Bewertung, ob ein Einfluss auf die Datenqualität gegeben ist
- Datenverifizierung – Bei bestimmten Datenfeldern werden stichprobenartig Wertebereich- und Feldtypkontrollen sowie logische Kontrollen durchgeführt. Bei den Wertebereichkontrollen werden die Felder auf zulässige Werte oder erlaubte Anzahl an Antwortmöglichkeiten (bei Mehrfachantworten) überprüft. Bei der Feldtypenkontrolle versteht man die Kontrolle, ob die eingegeben Werte zur Definition des Felds (bspw. Numerische Felder) passt. Logische Kontrollen überprüfen Zeitverläufe (bspw. die Reihenfolge von Visiten/Therapieeinheiten) oder die Therapie- und Protokoll-Compliance (u.a. durch Überprüfung der Freitextfelder).
- Doppelte Dateneingabe – Die Daten der CRFs werden durch zwei unabhängige Prüfer in die Datenbank übertragen. Anschließend findet ein Datenabgleich statt, um mögliche Übertragungsfehler bei der manuellen Eingabe sowie fehlende Daten aufzudecken. Die identifizierten Diskrepanzen werden jeweils von einer dritten autorisierten Person beseitigt, bis keine Unterschiede mehr vorliegen.
- Datenkontrolle durch den PI – vor der Datenanalyse bezüglich des primären Endpunkts und der sekundären Endpunkte findet eine erneute Kontrolle hinsichtlich Plausibilität statt.

Die Überprüfung der Daten konzentriert sich auf die Daten zu Zielkriterien, Patient*innensicherheit und Protokollabweichungen.

5.7.3 Umgang mit Rückfragen

Die Überprüfung der Daten erfolgt durch die o.g. Überprüfungen auf Vollständigkeit und medizinische Plausibilität. Ggf. können Rückfragen (Queries) entstehen, die in strukturierter Form an die Prüfer weitergeleitet werden. Anhand der Fragebögen muss der Prüfer die entstandenen Diskrepanzen überprüfen und beantworten. Diese Bögen werden anschließend wieder an das Datenmanagement Team weitergeleitet, wo in der Datenbank diese Diskrepanzen entsprechend korrigiert werden. Die Fragebögen werden gemeinsam mit den Erhebungsbögen am Prüfzentrum und beim Datenmanagement Team aufbewahrt.

Am Studienende wird nach Eingabe aller Eintragungen und Klärung der Rückfragen die Datenbank geschlossen. Dieser Vorgang wird dokumentiert.

6 Ethische, rechtliche und administrative Aspekte

6.1 Einhaltung ethischer und regulatorischer Anforderungen

Die Studie wurde in Übereinstimmung mit den Anforderungen des Tiroler Krankenanstaltengesetzes (Tir KAG), der Deklaration von Helsinki und den ICH-E6 Richtlinien, den OeAWI Richtlinien für Gute Wissenschaftliche Praxis sowie den Vorgaben der Datenschutz-Grundverordnung (DSGVO) und des österreichischen Datenschutzgesetzes (DSG) geplant.

Das Protokoll wurde in Anlehnung an die SPIRIT 2013-Richtlinien (Standard Protocol Items: Recommendations for Interventional Trials) und den TIDieR 2014-Richtlinien (Template for Intervention Description and Replication) verfasst.

6.2 Votum der Ethikkommission

Vor Beginn der Studie wird die Genehmigung des Studienplans, der Einverständniserklärung, der Änderungen dieser Dokumente und anderer relevanter Studiendokumente von der Ethikkommission (EK) der Medizinischen Universität Innsbruck eingeholt.

Über jegliche nachträgliche Änderung an den o.g. Dokumenten muss die EK informiert werden.

6.3 Patient*inneninformation und Einwilligungserklärung

Vor Beginn der Prüfung müssen alle Patient*innen den Prüfer*innen schriftlich seine/ihre Einwilligung erklären, nachdem sie zuvor in mündlicher und schriftlicher Form vollständig in für ihn verständlicher Weise über Wesen, Bedeutung und Tragweite der klinischen Prüfung aufgeklärt worden sind. Der Inhalt dieser Aufklärungsinformation wird auf der Einwilligungserklärung dokumentiert. Die Patient*innen werden in Kenntnis gesetzt, falls während der Studie wesentliche neue Erkenntnisse über die geprüfte Intervention auftreten.

Die Einwilligungserklärung der Patient*innen zur Teilnahme an der klinischen Prüfung erfolgt mit Datum und Unterschrift der Patient*innen sowie der Ärztin/des Arztes. Den Patient*innen wird ein Exemplar des unterschriebenen Informationsschreiben und Einwilligungserklärung zur Teilnahme an einer klinischen Studie für Patient*innen ausgehändigt. Das zweite Exemplar legt die Ärztin/der Arzt im Prüfordner ab.

Ebenso wird den Teilnehmer*innen eine Einwilligungserklärung für die Tonaufzeichnungen bei den Fokusgruppen vorgelegt. Sollten mehrere Teilnehmer*innen diese ablehnen, wird eine oder mehrere Fokusgruppen ohne Tonaufzeichnungen durchgeführt, nur mit Feldnotizen.

Es wird ausdrücklich darauf hingewiesen, dass bis zum Vorliegen einer rechtsgültigen Einwilligungserklärung der Patient*innen keinerlei Untersuchungen im Zusammenhang mit der Studie durchgeführt werden dürfen.

6.4 Patient*innenversicherung

Im Auftrag des Sponsors wurde für alle Patient*innen eine Personenschadenversicherung abgeschlossen. Diese Versicherung deckt alle eventuellen Schädigungen, die den Patient*innen direkt oder indirekt durch die Studienintervention oder Eingriffe im Zusammenhang mit der klinischen Studie erleiden, ab.

Um den Versicherungsschutz nicht zu gefährden, müssen sich die Patient*innen genau an die Anweisungen des Prüfpersonals halten. Weiterhin dürfen sich die Patient*innen während der klinischen Studie nicht ohne Zustimmung des/r Prüfer*in einer anderen medizinischen Behandlung unterziehen (ausgenommen sind Notfälle). Über eine Notfallbehandlung müssen die Patient*innen die Prüfer*innen unverzüglich informieren. Eine Gesundheitsschädigung, die als Folge der klinischen Studie eingetreten sein könnte, muss durch die Patient*innen unverzüglich den Prüfer*innen und der Versicherung angezeigt werden. Außerdem sind durch die Patient*innen alle zweckmäßigen Maßnahmen zu treffen, die der Aufklärung der Ursache und des Umfangs des eingetretenen Schadens dienen.

Die Patient*innen können die Versicherungsbedingungen bei den Prüfer*innen einsehen und auf Wunsch in Kopie erhalten.

6.5 Datenschutz und Schweigepflicht

Die Erhebung, Weitergabe, Speicherung und Auswertung persönlicher Daten innerhalb dieser klinischen Prüfung erfolgt nach gesetzlichen Bestimmungen der Datenschutz-Grundverordnung (EU) 20216/679 (DSGVO) und des österreichischen Datenschutzgesetzes (DSG). Voraussetzung dafür ist die freiwillige Zustimmung der Patient*innen im Rahmen der Einwilligungserklärung vor Teilnahme an der klinischen Prüfung. Die Patient*innen werden dazu innerhalb der Aufklärung über diese klinische Prüfung über Folgendes informiert:

1. Im Rahmen dieser klinischen Prüfung erhobene Daten werden auf Papiererhebungsbögen oder elektronischen Datenträgern aufgezeichnet, streng vertraulich behandelt und ohne Namensnennung (pseudonymisiert) nur weitergegeben an

- die (koordinierenden) Prüfer*innen der Studie zur Bewertung von unerwünschten Ereignissen,
- den Sponsor der Studie im Falle von schweren unerwünschten Ereignissen,
- die Biometrikerin der Studie zur wissenschaftlichen Auswertung,
- die zuständigen Ethikkommission der medizinischen Universität Innsbruck zur Überprüfung der ordnungsgemäßen Durchführung der Studie sowie zur Bewertung von Studienergebnissen und unerwünschten Ereignissen.

2. Dokumente, welche personenbezogene Daten enthalten (z.B. die unterschriebene Informationsschreiben und Einwilligungserklärung zur Teilnahme an einer klinischen Studie für Patient*innen, Identifikationsliste für Patient*innen) verbleibt am Studienzentrum und unterliegen der Schweigepflicht der Prüfärztin/des Prüfarztes.

3. Soweit dies für die Überprüfung der klinischen Prüfung notwendig ist, können autorisierte und zur Verschwiegenheit verpflichtete Beauftragte der zuständigen Überwachungsbehörde in die bei den Prüfer*innen vorhandenen personenbezogenen Daten Einsicht nehmen. Für diese Maßnahme werden die Prüfer*innen von der ärztlichen Schweigepflicht entbunden.

4. Die Einwilligung zur Erhebung und Verarbeitung der personenbezogenen Daten im Rahmen dieser klinischen Prüfung ist unwiderruflich. Die Patient*innen werden darüber aufgeklärt, dass sie jederzeit die Teilnahme an der klinischen Prüfung – ohne Angabe von Gründen und ohne irgendwelche folgenden Nachteile – beenden kann. Im Falle des Widerrufs der Einwilligungserklärung, werden die bis zu diesem Zeitpunkt gespeicherten Daten ohne Namensnennung weiterhin verwendet, soweit dies erforderlich ist, um Wirkungen der Studienintervention festzustellen und zu sichern, dass schutzwürdige Interessen der betroffenen Person nicht beeinträchtigt werden.

7 Änderungen während der Studiendurchführung

7.1 Änderungen des Prüfplans (Amendments)

Zur Sicherstellung vergleichbarer Bedingungen sowie im Interesse einer einwandfreien Datenauswertung ist eine Änderung der vereinbarten und im Prüfplan niedergelegten Prüfungsbedingungen nicht vorgesehen.

In Ausnahmefällen sind jedoch Änderungen der Prüfungsbedingungen möglich. Diese erfolgen nur nach gegenseitiger Abstimmung zwischen dem Koordinierenden Prüfer und dem Sponsor. Jede Änderung der im Prüfplan vorgesehenen Studienprozedur muss schriftlich,

unter Angabe der jeweiligen Gründe, erfolgen und von allen Studienverantwortlichen unterschrieben werden. Die Änderungen gelten dann als Bestandteil des Prüfplans. Sofern erforderlich (bei z. B. bedeutsamen Veränderungen, die einen direkten Einfluss auf die Sicherheit der Teilnehmer*innen der Studie erkennen lassen), ist die Zustimmung der zuständigen Ethikkommission und/oder Behörden sowie der Patient*innen zu den Prüfplanänderungen einzuholen.

Der Prüfer besitzt die Möglichkeit, in dringenden Situationen zwischen dem Antrag auf eine Prüfplanänderung und deren Ablehnung oder Inkrafttreten einen generellen Studienstopp zu verfügen. Dieser ist obligatorisch, wenn eine Prüfplanänderung oder eine Änderung des Wissensstandes über die studienspezifischen Untersuchungen mit potenzieller Neubewertung der Nutzen-Risiko-Kalkulation für die Versuchsperson erfolgt. In diesem Fall wird die Studie erst nach neuerlichem Votum der Ethikkommission fortgeführt.

7.2 Abweichungen vom Prüfplan

Abweichungen vom Prüfplan sollten möglichst vermieden werden. Falls dennoch Abweichungen vorkommen sollten, müssten diese unverzüglich dokumentiert und dem Sponsor sowie dem Förderer gemeldet werden. Die Abweichung muss vom Sponsor hinsichtlich ihrer Auswirkungen auf die Sicherheit der Patient*innen und Datenqualität bewertet und, falls notwendig, entsprechende Maßnahmen eingeleitet werden. Eine Liste der Protokollabweichungen wird der Person, welche die Datenanalyse durchführt, gemeinsam mit dem Datenexport mitgeliefert.

7.3 Information der Studienteilnehmer über die Ergebnisse der Studie

Nach Beendigung der Studie, der abschließenden Datenanalyse, dem Verfassen des initialen und finalen Studienberichts werden die Teilnehmer*innen der Studie postalisch über die Ergebnisse der Studie informiert. Diese Informationen enthalten ausschließlich anonymisierte Daten.

8 Funding, Interessenskonflikte und Entschädigung der Teilnehmer

8.1 Funding der Studie

Bei der geplanten Studie handelt es sich um eine akademische Eigenstudie ohne externes Funding.

8.2 Mögliche Interessenkonflikte

Die Interessenskonflikte der Prüfärzt*innen sind in einem gesonderten Formular beigelegt. Es handelt sich um eine prospektive randomisierte kontrollierte Pilotstudie mit physiotherapeutischer Intervention und einer Datenerhebung mit Verblindung. Keiner der der Prüfärzt*innen erhält ein Honorar für seine Tätigkeit.

8.3 Honorar und Entschädigung der Teilnehmer*innen

Die Teilnehmer*innen der Studie erhalten kein Honorar und keine Entschädigung für ihre Teilnahme an der Studie.

9 Literaturverzeichnis

Alcock, L., Galna, B., Hausdorff, J.M., Lord, S., Rochester, L., 2020. Enhanced Obstacle Contrast to Promote Visual Scanning in Fallers with Parkinson’s Disease: Role of Executive Function. Neuroscience 436, 82–92. https://doi.org/10.1016/j.neuroscience.2020.03.032

Ambati, V.N.P., Saucedo, F., Murray, N.G., Powell, D.W., Reed-Jones, R.J., 2016. Constraining eye movement in individuals with Parkinson’s disease during walking turns. Exp. Brain Res. 234, 2957–2965. https://doi.org/10.1007/s00221-016-4698-1

Andlin-Sobocki, P., Jonsson, B., Wittchen, H.-U., Olesen, J., 2005. Cost of disorders of the brain in Europe. Eur. J. Neurol. 12, 1–27. https://doi.org/10.1111/j.1468-1331.2005.01202.x

Baker, T., Pitman, J., MacLellan, M.J., Reed-Jones, R.J., 2020. Visual Cues Promote Head First Strategies During Walking Turns in Individuals With Parkinson’s Disease. Front. Sports Act. Living 2, 22. https://doi.org/10.3389/fspor.2020.00022

Barbieri, F.A., Polastri, P.F., Gobbi, L.T.B., Simieli, L., Pereira, V.I.A., Baptista, A.M., Moretto, G.F., Fiorelli, C.M., Imaizumi, L.F.I., Rodrigues, S.T., 2018. Obstacle circumvention and eye coordination during walking to least and most affected side in people with Parkinson’s disease. Behav. Brain Res. 346, 105–114. https://doi.org/10.1016/j.bbr.2017.11.032

Beck, A.T., Steer, R.A., Carbin, M.G., 1988. Psychometric properties of the Beck Depression Inventory: Twenty-five years of evaluation. Clin. Psychol. Rev. 8, 77–100. https://doi.org/10.1016/0272-7358(88)90050-5

Bennie, S., Bruner, K., Dizon, A., Fritz, H., Goodman, B., Peterson, S., 2003. Measurements of Balance: Comparison of the Timed “Up and Go” Test and Functional Reach Test with the Berg Balance Scale. J. Phys. Ther. Sci. 15, 93–97. https://doi.org/10.1589/jpts.15.93

Berg, K., Wood-Dauphine, S., Williams, J.I., Gayton, D., 1989. Measuring balance in the elderly: preliminary development of an instrument. Physiother. Can. 41, 304–311. https://doi.org/10.3138/ptc.41.6.304

Berger, K., Broll, S., Winkelmann, J., Heberlein, I., Müller, T., Ries, V., 1999. Untersuchung zur Reliabilität der deutschen Version des PDQ-39: Ein krankheitsspezifischer Fragebogen zur Erfassung der Lebensqualität von Parkinson-Patienten. Aktuelle Neurol. 26, 180–184. https://doi.org/10.1055/s-2007-1017628

Brusse, K.J., Zimdars, S., Zalewski, K.R., Steffen, T.M., 2005. Testing functional performance in people with Parkinson disease. Phys. Ther. 85, 134–141.

Camacho, P.B., Carbonari, R., Shen, S., Zadikoff, C., Kramer, A.F., López-Ortiz, C., 2019a. Voluntary Saccade Training Protocol in Persons With Parkinson’s Disease and Healthy Adults. Front. Aging Neurosci. 11, 77. https://doi.org/10.3389/fnagi.2019.00077

Camacho, P.B., Carbonari, R., Shen, S., Zadikoff, C., Kramer, A.F., López-Ortiz, C., 2019b. Voluntary Saccade Training Protocol in Persons With Parkinson’s Disease and Healthy Adults. Front. Aging Neurosci. 11, 77. https://doi.org/10.3389/fnagi.2019.00077

Carpinella, I., Cattaneo, D., Bonora, G., Bowman, T., Martina, L., Montesano, A., Ferrarin, M., 2017. Wearable Sensor-Based Biofeedback Training for Balance and Gait in Parkinson Disease: A Pilot Randomized Controlled Trial. Arch. Phys. Med. Rehabil. 98, 622-630.e3. https://doi.org/10.1016/j.apmr.2016.11.003

Cheng, P.-T., Liaw, M.-Y., Wong, M.-K., Tang, F.-T., Lee, M.-Y., Lin, P.-S., 1998. The sit-to-stand movement in stroke patients and its correlation with falling. Arch. Phys. Med. Rehabil. 79, 1043–1046. https://doi.org/10.1016/S0003-9993(98)90168-X

Craig, P., Dieppe, P., Macintyre, S., Michie, s., Nazareth, I., Petticrew, M., 2006. Developing and evaluating complex interventions: new guidance.

Cucca, A., Acosta, I., Berberian, M., Lemen, A.C., Rizzo, J.R., Ghilardi, M.F., Quartarone, A., Feigin, A.S., Di Rocco, A., Biagioni, M.C., 2018. Visuospatial exploration and art therapy intervention in patients with Parkinson’s disease: an exploratory therapeutic protocol. Complement. Ther. Med. 40, 70–76. https://doi.org/10.1016/j.ctim.2018.07.011

Dal Bello-Haas, V., Klassen, L., Sheppard, M.S., Metcalfe, A., 2011. Psychometric Properties of Activity, Self-Efficacy, and Quality-of-Life Measures in Individuals with Parkinson Disease. Physiother. Can. 63, 47–57. https://doi.org/10.3138/ptc.2009-08

Dias, N., Kempen, G.I.J.M., Todd, C.J., Beyer, N., Freiberger, E., Piot-Ziegler, C., Yardley, L., Hauer, K., 2006. Die Deutsche Version der Falls Efficacy Scale-International Version (FES-I). Z. Für Gerontol. Geriatr. 39, 297–300. https://doi.org/10.1007/s00391-006-0400-8

Dite, W., Temple, V.A., 2002. A clinical test of stepping and change of direction to identify multiple falling older adults. Arch. Phys. Med. Rehabil. 83, 1566–1571. https://doi.org/10.1053/apmr.2002.35469

Duncan, R.P., Earhart, G.M., 2013. Four Square Step Test Performance in People With

Parkinson Disease. J. Neurol. Phys. Ther. 37, 2–8. https://doi.org/10.1097/NPT.0b013e31827f0d7a

Flansbjer, U.-B., Lexell, J., Holmbäck, A.M., Downham, D., Patten, C., 2005. RELIABILITY OF GAIT PERFORMANCE TESTS IN MEN AND WOMEN WITH HEMIPARESIS AFTER STROKE. J. Rehabil. Med. 37, 75–82. https://doi.org/10.1080/16501970410017215

Franchignoni, F., Martignoni, E., Ferriero, G., Pasetti, C., 2005. Balance and fear of falling in Parkinson's disease. Parkinsonism Relat. Disord. 11, 427–433. https://doi.org/10.1016/j.parkreldis.2005.05.005

Giladi, N., Shabtai, H., Simon, E.S., Biran, S., Tal, J., Korczyn, A.D., 2000. Construction of freezing of gait questionnaire for patients with Parkinsonism. Parkinsonism Relat. Disord. 6, 165–170. https://doi.org/10.1016/S1353-8020(99)00062-0

Global, regional, and national burden of Parkinson's disease, 1990-2016: a systematic analysis for the Global Burden of Disease Study 2016., 2018. . Lancet Neurol. 17, 939–953. https://doi.org/10.1016/S1474-4422(18)30295-3

Grimes, D., Fitzpatrick, M., Gordon, J., Miyasaki, J., Fon, E.A., Schlossmacher, M., Suchowersky, O., Rajput, A., Lafontaine, A.L., Mestre, T., Appel-Cresswell, S., Kalia, S.K., Schoffer, K., Zurowski, M., Postuma, R.B., Udow, S., Fox, S., Barbeau, P., Hutton, B., 2019. Canadian guideline for Parkinson disease. Can. Med. Assoc. J. 191, E989–E1004. https://doi.org/10.1503/cmaj.181504

Hoehn, M.M., Yahr, M.D., 1967. Parkinsonism: onset, progression, and mortality. Neurology 17, 427–427. https://doi.org/10.1212/WNL.17.5.427

Hofheinz, M., Schusterschitz, C., 2010. Dual task interference in estimating the risk of falls and measuring change: a comparative, psychometric study of four measurements. Clin. Rehabil. 24, 831–842. https://doi.org/10.1177/0269215510367993

Huang, S.-L., Hsieh, C.-L., Wu, R.-M., Tai, C.-H., Lin, C.-H., Lu, W.-S., 2011. Minimal Detectable Change of the Timed "Up & Go" Test and the Dynamic Gait Index in People With Parkinson Disease. Phys. Ther. 91, 114–121. https://doi.org/10.2522/ptj.20090126

Hughes, A.J., Daniel, S.E., Kilford, L., Lees, A.J., 1992. Accuracy of clinical diagnosis of idiopathic Parkinson's disease: a clinico-pathological study of 100 cases. J. Neurol. Neurosurg. Psychiatry 55, 181–184. https://doi.org/10.1136/jnnp.55.3.181

Jenkinson, C., Fitzpatrick, R., Peto, V., Greenhall, R., Hyman, N., 1997. The Parkinson's Disease Questionnaire (PDQ-39): development and validation of a Parkinson's disease summary index score. Age Ageing 26, 353–357. https://doi.org/10.1093/ageing/26.5.353

Keus, S., Munneke, M., Graziano, M., Paltamaa, J., Pelosin, E., Domingos, J., Brühlmann, S., Ramaswamy, B., Prins, J., Struiksma, C., Rochester, L., Nieuwboer, A., Bloem, B., 2014. Europäische Physiotherapie-Leitlinie beim idiopathischen Parkinson-Syndrom.

Knox, P.C., Wolohan, F.D.A., 2015. Temporal Stability and the Effects of Training on Saccade Latency in "Express Saccade Makers." PLOS ONE 10, e0120437. https://doi.org/10.1371/journal.pone.0120437

Kühner, C., Bürger, C., Keller, F., Hautzinger, M., 2007. Reliabilität und Validität des revidierten Beck-Depressionsinventars (BDI-II): Befunde aus deutschsprachigen

Stichproben. Nervenarzt 78, 651–656. https://doi.org/10.1007/s00115-006-2098-7

Leddy, A.L., Crowner, B.E., Earhart, G.M., 2011. Functional Gait Assessment and Balance Evaluation System Test: Reliability, Validity, Sensitivity, and Specificity for Identifying Individuals With Parkinson Disease Who Fall. Phys. Ther. 91, 102–113. https://doi.org/10.2522/ptj.20100113

Lindholm, B., Nilsson, M.H., Hansson, O., Hagell, P., 2018. The clinical significance of 10-m walk test standardizations in Parkinson’s disease. J. Neurol. 265, 1829–1835. https://doi.org/10.1007/s00415-018-8921-9

Mak, M.K.Y., Pang, M.Y.C., 2009. Fear of falling is independently associated with recurrent falls in patients with Parkinson’s disease: a 1-year prospective study. J. Neurol. 256, 1689–1695. https://doi.org/10.1007/s00415-009-5184-5

Maranhão-Filho, P.A., Maranhão, E.T., Lima, M.A., Silva, M.M. da, 2011. Rethinking the neurological examination II: dynamic balance assessment. Arq. Neuropsiquiatr. 69, 959–963. https://doi.org/10.1590/S0004-282X2011000700022

Martinez-Martin, P., Reddy, P., Antonini, A., Henriksen, T., Katzenschlager, R., Odin, P., Todorova, A., Naidu, Y., Tluk, S., Chandiramani, C., Martin, A., Chaudhuri, K.R., 2011. Chronic Subcutaneous Infusion Therapy with Apomorphine in Advanced Parkinson’s Disease Compared to Conventional Therapy: A Real Life Study of Non Motor Effect. J. Park. Dis. 1, 197–203. https://doi.org/10.3233/JPD-2011-11037

Martinez-Martin, P., Serrano-Dueñas, M., Forjaz, M.J., Serrano, M.S., 2007. Two questionnaires for Parkinson’s disease: are the PDQ-39 and PDQL equivalent? Qual. Life Res. 16, 1221–1230. https://doi.org/10.1007/s11136-007-9224-2

Matsumoto, H., Terao, Y., Furubayashi, T., Yugeta, A., Fukuda, H., Emoto, M., Hanajima, R., Ugawa, Y., 2011. Small saccades restrict visual scanning area in Parkinson’s disease. Mov. Disord. Off. J. Mov. Disord. Soc. 26, 1619–1626. https://doi.org/10.1002/mds.23683

Mong, Y., Teo, T.W., Ng, S.S., 2010. 5-Repetition Sit-to-Stand Test in Subjects With Chronic Stroke: Reliability and Validity. Arch. Phys. Med. Rehabil. 91, 407–413. https://doi.org/10.1016/j.apmr.2009.10.030

Morris, S., Morris, M.E., Iansek, R., 2001. Reliability of Measurements Obtained With the Timed “Up & Go” Test in People With Parkinson Disease. Phys. Ther. 81, 810–818. https://doi.org/10.1093/ptj/81.2.810

National Institute for Health and Care Excellence (Great Britain), 2017. Parkinson’s disease in adults: diagnosis and management : full guideline.

Olson, M., Lockhart, T.E., Lieberman, A., 2019. Motor Learning Deficits in Parkinson’s Disease (PD) and Their Effect on Training Response in Gait and Balance: A Narrative Review. Front. Neurol. 10, 62. https://doi.org/10.3389/fneur.2019.00062

Petersen, C., Steffen, T., Paly, E., Dvorak, L., Nelson, R., 2017. Reliability and Minimal Detectable Change for Sit-to-Stand Tests and the Functional Gait Assessment for Individuals With Parkinson Disease. J. Geriatr. Phys. Ther. 40, 223–226. https://doi.org/10.1519/JPT.0000000000000102

Peto, V., Jenkinson, C., Fitzpatrick, R., Greenhall, R., 1995. The development and validation

of a short measure of functioning and well being for individuals with Parkinson’s disease. Qual. Life Res. 4, 241–248. https://doi.org/10.1007/BF02260863

Podsiadlo, D., Richardson, S., 1991. The Timed “Up & Go”: A Test of Basic Functional Mobility for Frail Elderly Persons. J. Am. Geriatr. Soc. 39, 142–148. https://doi.org/10.1111/j.1532-5415.1991.tb01616.x

Reed-Jones, R.J., Powell, D.W., 2017a. The effects of gaze stabilization on gait parameters in individuals with Parkinson’s disease. Neurosci. Lett. 655, 156–159. https://doi.org/10.1016/j.neulet.2017.07.013

Reed-Jones, R.J., Powell, D.W., 2017b. The effects of gaze stabilization on gait parameters in individuals with Parkinson’s disease. Neurosci. Lett. 655, 156–159. https://doi.org/10.1016/j.neulet.2017.07.013

Scherfer, E., Bohls, C., Freiberger, E., Heise, K.-F., Hogan, D., 2006. Berg-Balance-Scale - deutsche Version. physioscience 2, 59–66. https://doi.org/10.1055/s-2006-926833

Schlenstedt, C., Brombacher, S., Hartwigsen, G., Weisser, B., Möller, B., Deuschl, G., 2016. Comparison of the Fullerton Advanced Balance Scale, Mini-BESTest, and Berg Balance Scale to Predict Falls in Parkinson Disease. Phys. Ther. 96, 494–501. https://doi.org/10.2522/ptj.20150249

Schrag, A., 2000. What contributes to quality of life in patients with Parkinson’s disease? J. Neurol. Neurosurg. Psychiatry 69, 308–312. https://doi.org/10.1136/jnnp.69.3.308

Shumway-Cook, A., Brauer, S., Woollacott, M., 2000. Predicting the Probability for Falls in Community-Dwelling Older Adults Using the Timed Up & Go Test. Phys. Ther. 80, 896–903. https://doi.org/10.1093/ptj/80.9.896

Silva de Lima, A.L., Evers, L.J.W., Hahn, T., de Vries, N.M., Daeschler, M., Boroojerdi, B., Terricabras, D., Little, M.A., Bloem, B.R., Faber, M.J., 2018. Impact of motor fluctuations on real-life gait in Parkinson’s patients. Gait Posture 62, 388–394. https://doi.org/10.1016/j.gaitpost.2018.03.045

Skelton, D.A., Becker, C., Lamb, S.E., Close, J.C.T., Zijlstra, W., Yardley, L., Todd, C.J., 2004. Prevention of Falls Network Europe: a thematic network aimed at introducing good practice in effective falls prevention across Europe. Eur. J. Ageing 1, 89–94. https://doi.org/10.1007/s10433-004-0008-z

Steffen, T., Seney, M., 2008a. Test-Retest Reliability and Minimal Detectable Change on Balance and Ambulation Tests, the 36-Item Short-Form Health Survey, and the Unified Parkinson Disease Rating Scale in People With Parkinsonism. Phys. Ther. 88, 733–746. https://doi.org/10.2522/ptj.20070214

Steffen, T., Seney, M., 2008b. Test-Retest Reliability and Minimal Detectable Change on Balance and Ambulation Tests, the 36-Item Short-Form Health Survey, and the Unified Parkinson Disease Rating Scale in People With Parkinsonism. Phys. Ther. 88, 733–746. https://doi.org/10.2522/ptj.20070214

Stuart, S., Lord, S., Galna, B., Rochester, L., 2018. Saccade frequency response to visual cues during gait in Parkinson’s disease: the selective role of attention. Eur. J. Neurosci. 47, 769–778. https://doi.org/10.1111/ejn.13864

Thieme, H., Ritschel, C., Zange, C., 2009. Functional Gait Assessment – deutsche Version. physioscience 5, 5–11. https://doi.org/10.1055/s-0028-1109151

Tinetti, M.E., Richman, D., Powell, L., 1990. Falls Efficacy as a Measure of Fear of Falling. J. Gerontol. 45, P239–P243. https://doi.org/10.1093/geronj/45.6.P239

Tombaugh, T.N., McIntyre, N.J., 1992. The Mini-Mental State Examination: A Comprehensive Review. J. Am. Geriatr. Soc. 40, 922–935. https://doi.org/10.1111/j.1532-5415.1992.tb01992.x

Tönges, L., Bartig, D., Muhlack, S., Jost, W., Gold, R., Krogias, C., 2019. Charakteristika und Dynamik der stationären Behandlung von Parkinson-Patienten in Deutschland: Analyse von 1,5 Mio. Patientenfällen aus den Jahren 2010 bis 2015. Nervenarzt 90, 167–174. https://doi.org/10.1007/s00115-018-0590-5

Visser, M., Leentjens, A.F.G., Marinus, J., Stiggelbout, A.M., van Hilten, J.J., 2006. Reliability and validity of the Beck depression inventory in patients with Parkinson’s disease. Mov. Disord. 21, 668–672. https://doi.org/10.1002/mds.20792

Vogler, A., Janssens, J., Nyffeler, T., Bohlhalter, S., Vanbellingen, T., 2015. German translation and validation of the “freezing of gait questionnaire” in patients with Parkinson’s disease. Park. Dis. 2015, 982058. https://doi.org/10.1155/2015/982058

Wrisley, D.M., Marchetti, G.F., Kuharsky, D.K., Whitney, S.L., 2004. Reliability, internal consistency, and validity of data obtained with the functional gait assessment. Phys. Ther. 84, 906–918.

Yang, Y., Wang, Y., Zhou, Y., Chen, C., Xing, D., 2016. Reliability of functional gait assessment in patients with Parkinson disease: Interrater and intrarater reliability and internal consistency. Medicine (Baltimore) 95, e4545. https://doi.org/10.1097/MD.0000000000004545

Yang, Y., Wang, Y., Zhou, Y., Chen, C., Xing, D., Wang, C., 2014. Validity of the Functional Gait Assessment in Patients With Parkinson Disease: Construct, Concurrent, and Predictive Validity. Phys. Ther. 94, 392–400. https://doi.org/10.2522/ptj.20130019

Yardley, L., Beyer, N., Hauer, K., Kempen, G., Piot-Ziegler, C., Todd, C., 2005. Development and initial validation of the Falls Efficacy Scale-International (FES-I). Age Ageing 34, 614–619. https://doi.org/10.1093/ageing/afi196

Effekte eines aktivitätsorientierten, physiotherapeutischen Trainingsprogramms mit und Blickbewegungstraining auf die dynamische Balance und das Sturzrisiko bei Personen mit Idiopathischem Parkinson Syndrom:

Eine randomisierte kontrollierte Pilotstudie

Instruktor Manual

für Physiotherapeut*innen

Interventionsgruppe

REHA ZENTRUM MÜNSTER
Klinikum für Rehabilitation in Tirol

Instruktor Manual für Physiotherapeut*innen

Allgemeine Hinweise:

- Vor Beginn des Programms erfragen Sie den aktuellen Gesundheitszustand der Teilnehmenden.
- Die Übungen sollen mit dem Ziel der Erreichung einer größtmöglichen Amplitude ausgeführt werden.
- Die Teilnehmenden dürfen jederzeit eine Pause einlegen.
- Verwenden Sie die verfügbaren Physiotherapiegeräte, z. B. Stühle, Hocker, Gymnastikmatten, Gymnastikbälle, Tennisbälle, Kegel, Seile, Tritthocker, Hanteln, Balancebretter, Koordinationsreifen, Reis- und Bohnensäcke.
- Fügen Sie ein Gehtraining mit verschiedenen Variationen hinzu (z. B. Fersengang, seitliches Gehen, Rückwärtsgehen, Überqueren von Hindernissen während des Gehens, Gehen durch einen Slalomparcours, Wechsel der Gehrichtung und Geschwindigkeit)
- Verwenden Sie auch Treppensteigen als Training
- Die durchzuführenden Übungen werden im Verlauf der 4-wöchigen Therapie stetig individuell im vorgegebenen standardisierten Rahmen an die Teilnehmenden angepasst und in der Schwierigkeit gesteigert, wie es für die Patient*innen angemessen ist.
- Die Dokumentationsvorlage beginnt auf Seite 24. Bitte dokumentieren Sie als durchführende*r Physiotherapeut*in hier die in den jeweiligen Wochen ausgeführten Übungen mit der ausgeführten Steigerung (I-III) und den ausgeführten Blickfixationspunkten (A-C).
- Falls die Teilnehmenden sich unsicher fühlen, können Sie sich jederzeit an der Wand oder an einem Stuhl festhalten. Bei Bedarf kann Hilfestellung gegeben werden.

Aufwärmen

Übung 1: Kopfflexion und -extension im Sitz

**Übungssetting**		**Blickfixationspunkt**
Startposition	Sitz auf einem Hocker/Stuhl im vorderen Bereich;<br>Füße hüftbreit auf dem Boden aufgestellt	Mitte des Fadenkreuzes
Endposition	Maximale Flexion der HWS<br>Maximale Extension der HWS	Blick Richtung Dreieck (unten)<br>Blick Richtung Dreieck (oben)
Steigerungs-optionen	I: Zusätzlich die Arme mit der Bewegung auf 90° (GHG) anheben und absenken<br>II: Zusätzlich die Arme auf 90° (GHG) anheben und halten<br>III: Zusätzlich die Arme auf 180° (GHG) anheben und halten	A: Blick Richtung Viereck (unten & oben)<br>B: Blick Richtung Punkt (unten & oben)
Wiederholungs-zahl	5 x jede Richtung; jeweils 5 Sek. in der Endposition halten	

Übung 2: Kopfrotation im Sitz

**Übungssetting**		**Blickfixationspunkt**
Startposition	Sitz auf einem Hocker/Stuhl im vorderen Bereich;<br>Füße hüftbreit auf dem Boden aufgestellt	Mitte des Fadenkreuzes
Endposition	Rotation des Kopfes nach rechts<br>Rotation des Kopfes nach links	Blick Richtung Dreieck (rechts)<br>Blick Richtung Dreieck (links)
Steigerungs-optionen	I: Zusätzlich die Arme mit der Bewegung auf 90° (GHG) anheben und absenken<br>II: Zusätzlich die Arme auf 90° (GHG) anheben und halten<br>III: Zusätzlich die Arme auf 180° (GHG) anheben und halten	A: Blick Richtung Viereck (rechts & links)<br>B: Blick Richtung Punkt (rechts & links)
Wiederholungs-zahl	5 x jede Seite; jeweils 5 Sek. in der Endposition halten	

Hauptteil

Übung 3: Sit-to-Stand

**Übungssetting**		**Blickfixationspunkt**
Startposition	Sitz auf einem Hocker/Stuhl im vorderen Bereich;<br>Füße hüftbreit auf dem Boden aufgestellt	Mitte des Fadenkreuzes
Endposition	Stehende Position vor dem Stuhl	Mitte des Fadenkreuzes
Steigerungs-optionen	I: rechter Fuß nach vorne, linker Fuß weiter zurück<br>II: linker Fuß nach vorne, rechter Fuß weiter zurück<br>III: beide Füße weiter zurück	A: Dreieck<br>B: Viereck<br>C: Punkt
Wiederholungs-zahl	10 x; jeweils 5 Sek. in der Endposition halten	

Übung 4: Kopfflexion und -extension im Stand

**Übungssetting**		**Blickfixationspunkt**
Startposition	Stand: Füße hüftbreit auf dem Boden aufgestellt, Knie leicht gebeugt	Mitte des Fadenkreuzes
Endposition	Maximale Flexion der HWS<br>Maximale Extension der HWS	Blick Richtung Dreieck (unten)<br>Blick Richtung Dreieck (oben)
Steigerungs-optionen	I: Zusätzlich die Arme mit der Bewegung auf 90° (GHG) anheben und absenken<br>II: Zusätzlich die Arme auf 90° (GHG) anheben und halten<br>III: Zusätzlich die Arme auf 180° (GHG) anheben und halten	A: Blick Richtung Viereck (unten & oben)<br>B: Blick Richtung Punkt (unten & oben)
Wiederholungs-zahl	5 x jede Seite; jeweils 5 Sek. in der Endposition halten	

Übung 5: Kopfrotation im Stand

**Übungssetting**		**Blickfixationspunkt**
Startposition	Stand: Füße hüftbreit auf dem Boden aufgestellt, Knie leicht gebeugt	Mitte des Fadenkreuzes
Endposition	Rotation der HWS nach rechts<br>Rotation der HWS nach links	Blick Richtung Dreieck (rechts)<br>Blick Richtung Dreieck (links)
Steigerungs-optionen	I: Zusätzlich die Arme mit der Bewegung auf 90° (GHG) anheben und absenken<br>II: Zusätzlich die Arme mit der Bewegung auf 90° (GHG) anheben und absenken und den Rumpf mitrotieren<br>III: Zusätzlich die Arme mit der Bewegung auf 180° (GHG) anheben und absenken	A: Blick Richtung Viereck (rechts & links)<br>B: Blick Richtung Punkt (rechts & links)
Wiederholungs-zahl	5 x jede Seite; jeweils 5 Sek. in der Endposition halten	

Übung 6: Schritt nach vorne

**Übungssetting**		**Blickfixationspunkt**
Startposition	Stand: Füße hüftbreit auf dem Boden aufgestellt, Knie leicht gebeugt	Mitte des Fadenkreuzes
Endposition	Rechter Fuß nach vorne in Schrittstellung;<br>Linker Fuß nach vorne in Schrittstellung	Mitte des Fadenkreuzes
Steigerungs-optionen	I: größerer Schritt<br>II: Ausfallschritt nach vorne mit Armen 90° ABD (GHG)<br>III: Ausfallschritt mit Armen 90° Flexion (GHG) nach oben bringen und die Geschwindigkeit steigern	A: Dreieck (oben)<br>B: Viereck (oben)<br>C: Punkt (oben)
Wiederholungs-zahl	5 x jede Seite; jeweils 5 Sek. in der Endposition halten	

Übung 7: Schritt zur Seite

**Übungssetting**		**Blickfixationspunkt**
Startposition	Stand: Füße hüftbreit auf dem Boden aufgestellt, Knie leicht gebeugt	Mitte des Fadenkreuzes
Endposition	Schritt zur rechten Seite mit dem rechten Fuß;<br>Schritt zur linken Seite mit dem linken Fuß	Blick Richtung Dreieck (rechts)<br>Blick Richtung Dreieck (links)
Steigerungs-optionen	I: größerer Schritt zur Seite<br>II: Ausfallschritt Zur Seite mit Armen 90° ABD (GHG)<br>III: Ausfallschritt zur Seite mit Armen 90° ABD (GHG) und Fuß zeigt in die Richtung des Ausfallschritts	A: Viereck (rechts & links)<br>B: Punkt (rechts & links)
Wiederholungs-zahl	5 x jede Seite; jeweils 5 Sek. in der Endposition halten	

Übung 8: Schritt nach hinten

**Übungssetting**		**Blickfixationspunkt**
Startposition	Stand: Füße hüftbreit auf dem Boden aufgestellt, Knie leicht gebeugt	Mitte des Fadenkreuzes
Endposition	Rechter Fuß nach hinten,<br>linker Fuß nach hinten	Mitte des Fadenkreuzes
Steigerungs-optionen	I: größerer Schritt nach hinten<br>II: Ausfallschritt nach hinten mit den Armen 90° ABD (GHG)<br>III: Ausfallschritt nach hinten dabei die Arme 180° Flexion (GHG) bringen	A: Dreieck (oben)<br>B: Viereck (oben)<br>C: Punkt (oben)
Wiederholungs-zahl	5 x jede Seite; jeweils 5 Sek. in der Endposition halten	

Übung 9: Schritt diagonal nach vorne und hinten (Linke Diagonale)

Übungssetting		Blickfixationspunkt
Startposition	Stand: Füße hüftbreit auf dem Boden aufgestellt, Knie leicht gebeugt	Dreieck links
Endposition	Rechter Fuß diagonal nach vorne (links); rechter Fuß diagonal nach hinten (rechts)	Dreieck links
Steigerungs-optionen	I: größerer Schritt nach vorne bzw. hinten<br>II: Ausfallschritt diagonal nach vorne bzw. hinten mit den Armen 90° ABD (GHG)<br>III: Ausfallschritt nach hinten dabei die Arme 180° Flexion (GHG) bringen	A: Viereck (links)<br>B: Punkt (links)<br>C: Kreuz grün (links)
Wiederholungs-zahl	5 x jede Seite; jeweils 5 Sek. in der Endposition halten	

Übung 10: Schritt diagonal nach vorne und hinten (Rechte Diagonale)

Übungssetting		Blickfixationspunkt
Startposition	Stand: Füße hüftbreit auf dem Boden aufgestellt, Knie leicht gebeugt	Dreieck rechts
Endposition	Linker Fuß diagonal nach vorne (rechts); linker Fuß diagonal nach hinten (links)	Dreieck rechts
Steigerungs-optionen	I: größerer Schritt nach vorne bzw. hinten<br>II: Ausfallschritt nach vorne bzw. hinten mit den Armen 90° ABD (GHG)<br>III: Ausfallschritt nach hinten dabei die Arme 180° Flexion (GHG) bringen	A: Viereck (rechts)<br>B: Punkt (rechts)<br>C: Kreuz grün (rechts)
Wiederholungs-zahl	5 x jede Seite; jeweils 5 Sek. in der Endposition halten	

Übung 11: Kniebeugen

**Übungssetting**		**Blickfixationspunkt**
Startposition	Stand: Füße breitbeinig auf dem Boden aufgestellt, Knie gebeugt	Mitte Fadenkreuz
Endposition	Gesäß tief bringen, Rücken in aufrechter Position	Auf der Mitte halten
Steigerungs-optionen	I: tiefer in die Kniebeuge gehen<br>II: Arme in U-Halte (GHG: 90° ABD, HUG: 90° Flex) seitlich neben dem Körper und mit der Kniebeuge nach vorne strecken (GHG 90°)<br>III: Schmalspuriger Stand in Kombination mit II	A: Dreieck unten<br>B: Blick von Dreieck (rechts) zu Dreieck (links)<br>C: Blick von Rechteck (rechts) zu Rechteck (links)
Wiederholungs-zahl	10 x; jeweils 5 Sek. in der Endposition halten	

Übung 12: Bogenschießen seitlich

**Übungssetting**		**Blickfixationspunkt**
Startposition	Stand: Füße hüftbreit auf dem Boden aufgestellt, Knie gebeugt	Mitte Fadenkreuz
Endposition	Rechter Arm in 90° ABD (GHG), linker Arm berührt die rechte Hand und geht langsam wieder zurück; linker Arm in 90° ABD (GHG), rechter Arm berührt die linke Hand und geht langsam wieder zurück	Dreieck (rechts & links)
Steigerungs-optionen	I: tiefer in die Knie<br>II: schmalspurig<br>III: Tandemstand	A: Viereck (rechts & links)<br>B: Blick von Viereck (rechts) zu Viereck (links)<br>C: Blick von Kreis (rechts) zu Kreis (links)
Wiederholungs-zahl	5 x jede Seite; jeweils 5 Sek. in der Endposition halten	

Übung 13: Kniebeugen breitbeinig, Kopf Flexion, Extension, Rotation

**Übungssetting**		**Blickfixationspunkt**
Startposition	Stand: Füße breitbeinig auf dem Boden aufgestellt, Knie gebeugt	Mitte Fadenkreuz
Endposition	Gesäß tief bringen, Rücken in aufrechter Position, Flexion und Extension des Kopfes	Dreieck (oben& unten)
Steigerungs-optionen	I: Arme 180° ABD (GHG), Flexion und Extension Kopf<br>II: Rechten Arm in 90° ABD (GHG) führen, linken Arm in 180° ABD (GHG) halten; Linken Arm in 90° ABD (GHG) führen, rechten Arm in 180° ABD (GHG) halten	A: Viereck (oben & unten)<br>B: Blick von Viereck (oben) zu Viereck (rechts)
Wiederholungs-zahl	5 x mit jedem Arm; jeweils 5 Sek. in der Endposition halten	

Übung 14: Kniebeugen schmalspurig, Kopf Flexion und Extension, Rotation

**Übungssetting**		**Blickfixationspunkt**
Startposition	Stand: Füße schmalspurig auf dem Boden aufgestellt, Knie gebeugt	Mitte Fadenkreuz
Endposition	Gesäß tief bringen, Rücken in aufrechter Position, Flexion und Extension des Kopfes	Dreieck (oben& unten)
Steigerungs-optionen	I: Arme 180° ABD (GHG), Flexion und Extension Kopf<br>II: Rechten Arm in 90° ABD (GHG) führen, linken Arm in 180° ABD (GHG) halten; Linken Arm in 90° ABD (GHG) führen, rechten Arm in 180° ABD (GHG) halten	A: Viereck (oben & unten)<br>B: Blick von Viereck (oben) zu Viereck (rechts)
Wiederholungs-zahl	5 x mit jedem Arm; jeweils 5 Sek. in der Endposition halten	

Übung 15: Knie anheben

**Übungssetting**		**Blickfixationspunkt**
Startposition	Stand: Füße hüftbreit auf dem Boden aufgestellt, Knie leicht gebeugt	Mitte Fadenkreuz
Endposition	Standbein links stabil, rechtes Bein in 90° Hüftflexion bringen;<br>Standbein rechts stabil, linkes Bein in 90° Hüftflexion bringen	Vom Dreieck (unten) zur Fadenkreuzmitte
Steigerungs-optionen	I: beide Arme in 90° ABD (GHG) bringen<br>II: zusätzlich mit der Bewegung den Kopf in Flexion bringen<br>III: die Geschwindigkeit erhöhen	A: Dreieck (unten) zu Dreieck (oben)<br>B: Viereck (unten) zu Viereck (oben)<br>C: Kreis (unten) zu Kreis (oben)
Wiederholungs-zahl	5 x jede Seite; jeweils 5 Sek. in der Endposition halten	

Übung 16: Füße vom Boden abheben

**Übungssetting**		**Blickfixationspunkt**
Startposition	Stand: Füße in einen breitbeinigen Stand bringen, Knie leicht gebeugt	Mitte Fadenkreuz
Endposition	Standbein links stabil, rechten Fuß leicht vom Boden abheben;<br>Standbein rechts stabil, linken Fuß leicht vom Boden abheben	Mitte Fadenkreuz
Steigerungs-optionen	I: beide Arme in 90° (GHG) ABD bringen, mit der Bewegung den Kopf nach rechts bzw. links rotieren<br>II: mit der Bewegung die Arme weit nach oben schwingen (GHG: 180° Flex)<br>III: mit der Bewegung die Arme versetzt nach vorne bzw. hinten schwingen (rechter Fuß, linker Arm nach vorne, rechter Arm nach hinten und umgekehrt)	A: Dreieck (rechts & links)<br>B: Viereck (rechts & links)<br>C: Kreis (rechts & links)
Wiederholungs-zahl	5 x jede Seite; jeweils 5 Sek. in der Endposition halten	

Übung 17: Buchstabe Z

**Übungssetting**		**Blickfixationspunkt**
Startposition	Stand: Füße in einen hüftbreiten Stand bringen, Knie leicht gebeugt	Grünes Kreuz (links oben)
Endposition	Entlang des Rasters eine gedachtes Z mit den Augen und der Kopfbewegung fahren	Grünes Kreuz (rechts unten)
Steigerungs-optionen	I: Stehen im Ausfallschritt<br>II: An Endpunkt kehren und rückwärts abfahren<br>III: Zusätzlich auf dem Boden das gedachte Z gehen; Schritt nach rechts, zweites Bein dazustellen; Schritt diagonal nach hinten links, zweites Bein dazustellen; Schritt nach rechts, zweites Bein dazustellen	
Wiederholungs-zahl	5 x	

Übung 18: Buchstabe M

**Übungssetting**		**Blickfixationspunkt**
Startposition	Stand: Füße in einen hüftbreiten Stand bringen, Knie leicht gebeugt	Grünes Kreuz (links unten)
Endposition	Entlang des Rasters eine gedachtes M mit den Augen und der Kopfbewegung fahren	Grünes Kreuz (rechts unten)
Steigerungs-optionen	I: Stehen im Ausfallschritt<br>II: An Endpunkt kehren und rückwärts abfahren<br>III: Zusätzlich auf dem Boden das gedachte M gehen; Schritt nach vorne, zweites Bein dazustellen; Schritt diagonal nach hinten rechts, zweites Bein dazustellen; Schritt diagonal nach vorne rechts, zweites Bein dazustellen; Schritt nach hinten, zweites Bein dazustellen	
Wiederholungs-zahl	5 x	

Übung 19: Buchstabe N

**Übungssetting**		**Blickfixationspunkt**
Startposition	Stand: Füße in einen hüftbreiten Stand bringen, Knie leicht gebeugt	Grünes Kreuz (links unten)
Endposition	Entlang des Rasters eine gedachtes N mit den Augen und der Kopfbewegung fahren	Roter Punkt (oben)
Steigerungs-optionen	I: Stehen im Ausfallschritt<br>II: An Endpunkt kehren und rückwärts abfahren<br>III: Zusätzlich auf dem Boden das gedachte N gehen; Schritt nach vorne, zweites Bein dazustellen; Schritt diagonal nach hinten rechts, zweites Bein dazustellen; Schritt nach vorne, zweites Bein dazustellen	
Wiederholungs-zahl	5 x	

Übung 20: Buchstabe W

**Übungssetting**		**Blickfixationspunkt**
Startposition	Stand: Füße in einen hüftbreiten Stand bringen, Knie leicht gebeugt	Grünes Kreuz (links oben)
Endposition	Entlang des Rasters eine gedachtes W mit den Augen und der Kopfbewegung fahren	Grünes Kreuz (rechts oben)
Steigerungs-optionen	I: Stehen im Ausfallschritt<br>II: An Endpunkt kehren und rückwärts abfahren<br>III: Zusätzlich auf dem Boden das gedachte W gehen; Schritt diagonal nach hinten rechts, zweites Bein dazustellen; Schritt diagonal nach vorne rechts, zweites Bein dazustellen; Schritt diagonal nach hinten rechts, zweites Bein dazustellen; Schritt diagonal nach vorne rechts, zweites Bein dazustellen	
Wiederholungs-zahl	5 x	

Cool-Down/Abwärmen

Übung 21: Schultern kreisen

**Übungssetting**	
Startposition	Entspannt auf dem Stuhl sitzen
Endposition	Schultern vorwärts und rückwärts kreisen

Übung 22: Arme lockern

**Übungssetting**	
Startposition	Stand: Füße in einen hüftbreiten Stand bringen, Knie leicht gebeugt<br>Ggf. Sitz auf einem Hocker/Stuhl im vorderen Bereich;<br>Schultern locker, Arme neben dem Körper
Endposition	Arme mit der Einatmung weit nach oben anheben; mit der Ausatmung locker nach unten schwingen

Übung 23: BWS lösen

**Übungssetting**	
Startposition	Entspannt auf dem Stuhl sitzen
Endposition	Arme 90° (GHG) ABD; Rotation nach rechts; Rotation links

Übung 24: Kopf drehen

**Übungssetting**	
Startposition	Entspannt auf dem Stuhl sitzen, Augen geschlossen
Endposition	Kopf entspannt nach vorne, hinten, zur Seite rotieren

Übung 25: Augen schließen

Übungssetting	
Startposition	Entspannt auf dem Stuhl sitzen
Endposition	Augen schließen, entspannt tief Ein- und Ausatmen

Übung 26: Augen Entspannung

Übungssetting	
Startposition	Sitz auf einem Hocker/Stuhl im vorderen Bereich;<br>Augen geschlossen
Endposition	Die Handflächen schnell aneinander reiben und die warmen Handflächen auf die geschlossenen Augen legen

Studientagebuch – Woche 1

Vom ………………. bis ……………….

TN-ID: …………………………………..

	Mo	Di	Mi	Do	Fr
**An welchen Tagen hat das Training stattgefunden?**					
	☐	☐	☐	☐	☐

	Übung (Nummer)	Schwierigkeitsgrad (I-III, A-C)	Übung (Nummer)	Schwierigkeitsgrad (I-III, A-C)
**Welche Übungen wurden durchgeführt? Bitte die Nummer der Übungen angeben**<br>**(z.B.: Übung 1, I, A)**				

	ja	nein
**Gab es beim Ausführen der Übungen Schwierigkeiten?**		
	☐	☐

Wenn ja:

- **Notieren Sie die Nummer der Übung**
- **Notieren Sie kurz die Schwierigkeit**

**Angaben zu eventuellen Stürzen**	Anzahl der Stürze	Grund des Sturzes (z.B. Schwindel, Glatteis, Stolpern, etc.)
**Während des Trainings**		
**Außerhalb des Trainings**		

Studientagebuch – Woche 2

Vom ………………. bis ……………….

TN-ID: …………………………………..

**An welchen Tagen hat das Training stattgefunden?**	**Mo**	**Di**	**Mi**	**Do**	**Fr**
	☐	☐	☐	☐	☐

**Welche Übungen wurden durchgeführt? Bitte die Nummer der Übungen angeben** (z.B.: Übung 1, I, A)	Übung (Nummer)	Schwierigkeitsgrad (I-III, A-C)	Übung (Nummer)	Schwierigkeitsgrad (I-III, A-C)

**Gab es beim Ausführen der Übungen Schwierigkeiten?**	ja	nein
	☐	☐

Wenn ja:

- **Notieren Sie die Nummer der Übung**
- **Notieren Sie kurz die Schwierigkeit**

**Angaben zu eventuellen Stürzen**<br>**Während des Trainings**	Anzahl der Stürze	Grund des Sturzes (z.B. Schwindel, Glatteis, Stolpern, etc.)

Außerhalb des Trainings

Studientagebuch – Woche 3

Vom ………………. bis ……………….

TN-ID: …………………………………..

**An welchen Tagen hat das Training stattgefunden?**	**Mo**	**Di**	**Mi**	**Do**	**Fr**
	☐	☐	☐	☐	☐

**Welche Übungen wurden durchgeführt? Bitte die Nummer der Übungen angeben**	Übung (Nummer)	Schwierigkeitsgrad (I-III, A-C)	Übung (Nummer)	Schwierigkeitsgrad (I-III, A-C)
**(z.B.: Übung 1, I, A)**				

**Gab es beim Ausführen der Übungen Schwierigkeiten?**	ja	nein
	☐	☐

Wenn ja:

- **Notieren Sie die Nummer der Übung**
- **Notieren Sie kurz die Schwierigkeit**

**Angaben zu eventuellen Stürzen**	Anzahl der Stürze	Grund des Sturzes (z.B. Schwindel, Glatteis, Stolpern, etc.)

Während des Trainings

Außerhalb des Trainings

Studientagebuch – Woche 4

Vom ………………. bis ……………….

TN-ID: …………………………………..

**An welchen Tagen hat das Training stattgefunden?**	**Mo**	**Di**	**Mi**	**Do**	**Fr**
	☐	☐	☐	☐	☐

**Welche Übungen wurden durchgeführt? Bitte die Nummer der Übungen angeben**	Übung (Nummer)	Schwierigkeitsgrad (I-III, A-C)	Übung (Nummer)	Schwierigkeitsgrad (I-III, A-C)
**(z.B.: Übung 1, I, A)**				

**Gab es beim Ausführen der Übungen Schwierigkeiten?**	ja	nein
	☐	☐

Wenn ja:

- **Notieren Sie die Nummer der Übung**
- **Notieren Sie kurz die Schwierigkeit**

	Anzahl der Stürze	Grund des Sturzes (z.B. Schwindel, Glatteis, Stolpern, etc.)
**Angaben zu eventuellen Stürzen**		
**Während des Trainings**		
**Außerhalb des Trainings**		

Effekte eines aktivitätsorientierten, physiotherapeutischen Trainingsprogramms mit und ohne Blickbewegungstraining auf die dynamische Balance und das Sturzrisiko bei Personen mit Idiopathischem Parkinson Syndrom:

Eine randomisierte kontrollierte Pilotstudie

Instruktor Manual

für Physiotherapeut*innen

Kontrollgruppe

REHA ZENTRUM MÜNSTER
Klinikum für Rehabilitation in Tirol

Instruktor Manual für Physiotherapeut*innen

Allgemeine Hinweise:

- Vor Beginn des Programms erfragen Sie den aktuellen Gesundheitszustand der Teilnehmenden.
- Die Übungen sollen mit dem Ziel der Erreichung einer größtmöglichen Amplitude ausgeführt werden.
- Die Teilnehmenden dürfen jederzeit eine Pause einlegen.
- Verwenden Sie die verfügbaren Physiotherapiegeräte, z. B. Stühle, Hocker, Gymnastikmatten, Gymnastikbälle, Tennisbälle, Kegel, Seile, Tritthocker, Hanteln, Balancebretter, Koordinationsreifen, Reis- und Bohnensäcke.
- Fügen Sie ein Gehtraining mit verschiedenen Variationen hinzu (z. B. Fersengang, seitliches Gehen, Rückwärtsgehen, Überqueren von Hindernissen während des Gehens, Gehen durch einen Slalomparcours, Wechsel der Gehrichtung und Geschwindigkeit)
- Verwenden Sie auch Treppensteigen als Training
- Die durchzuführenden Übungen werden im Verlauf der 4-wöchigen Therapie stetig individuell im vorgegebenen standardisierten Rahmen an die Teilnehmenden angepasst und in der Schwierigkeit gesteigert, wie es für die Patient*innen angemessen ist.
- Die Dokumentationsvorlage beginnt auf Seite 24. Bitte dokumentieren Sie als durchführende*r Physiotherapeut*in hier die in den jeweiligen Wochen ausgeführten Übungen mit der ausgeführten Steigerung (I-III) und den ausgeführten Blickfixationspunkten (A-C).
- Falls die Teilnehmenden sich unsicher fühlen, können Sie sich jederzeit an der Wand oder an einem Stuhl festhalten. Bei Bedarf kann Hilfestellung gegeben werden.

Aufwärmen

Übung 1: Kopfflexion und -extension im Sitz

**Übungssetting**	
Startposition	Sitz auf einem Hocker/Stuhl im vorderen Bereich;<br>Füße hüftbreit auf dem Boden aufgestellt
Endposition	Maximale Flexion der HWS<br>Maximale Extension der HWS
Steigerungs-optionen	I: Zusätzlich die Arme mit der Bewegung auf 90° (GHG) anheben und absenken<br>II: Zusätzlich die Arme auf 90° (GHG) anheben und halten<br>III: Zusätzlich die Arme auf 180° (GHG) anheben und halten
Wiederholungs-zahl	5 x jede Richtung; jeweils 5 Sek. in der Endposition halten

Übung 2: Kopfrotation im Sitz

**Übungssetting**	
Startposition	Sitz auf einem Hocker/Stuhl im vorderen Bereich;<br>Füße hüftbreit auf dem Boden aufgestellt
Endposition	Rotation der HWS nach rechts<br>Rotation der HWS nach links
Steigerungs-optionen	I: Zusätzlich die Arme mit der Bewegung auf 90° (GHG) anheben und absenken<br>II: Zusätzlich die Arme auf 90° (GHG) anheben und halten<br>III: Zusätzlich die Arme auf 180° (GHG) anheben und halten
Wiederholungs-zahl	5 x jede Seite; jeweils 5 Sek. in der Endposition halten

Hauptteil

Übung 3: Sit-to-Stand

**Übungssetting**	
Startposition	Sitz auf einem Hocker/Stuhl im vorderen Bereich;<br>Füße hüftbreit auf dem Boden aufgestellt
Endposition	Stehende Position vor dem Stuhl
Steigerungs-optionen	I: rechter Fuß nach vorne, linker Fuß weiter zurück<br>II: linker Fuß nach vorne, rechter Fuß weiter zurück<br>III: beide Füße weiter zurück
Wiederholungs-zahl	10 x; jeweils 5 Sek. in der Endposition halten

Übung 4: Diagonale mit den Armen

**Übungssetting**	
Startposition	Stand: Füße hüftbreit auf dem Boden aufgestellt, Knie leicht gebeugt;<br>Rechter bzw. linker Arm am gegenüberliegenden Becken (Ex/ADD/IR)
Endposition	Rechter Bzw. linker Arm gestreckt nach oben (Flex/ABD/AR)
Steigerungs-optionen	I: Knie tiefer gebeugt<br>II: Zusätzlich mit kleiner Hantel / Gewicht<br>III: Stehen im Tandemstand
Wiederholungs-zahl	5 x jede Richtung; jeweils 5 Sek. in der Endposition halten

Übung 5: Rotation im Stand

**Übungssetting**	
Startposition	Stand: Füße hüftbreit auf dem Boden aufgestellt, Knie leicht gebeugt; Arme auf 90° (GHG) abduziert
Endposition	Rotation des Oberkörpers und der Arme soweit es geht nach rechts<br>Rotation des Oberkörpers und der Arme soweit es geht nach links
Steigerungs-optionen	I: Die Arme in U-Haltung (GHG: 90° ABD, HUG: 90° Flex)<br>II: Stehen im Ausfallschritt<br>III: Arme zu Beginn 180° Flexion (GHG); mit der Drehung auf 90° ABD (GHG)
Wiederholungs-zahl	5 x jede Seite; jeweils 5 Sek. in der Endposition halten

Übung 6: Schritt nach vorne

**Übungssetting**	
Startposition	Stand: Füße hüftbreit auf dem Boden aufgestellt, Knie leicht gebeugt
Endposition	Rechter Fuß nach vorne in Schrittstellung;<br>Linker Fuß nach vorne in Schrittstellung
Steigerungs-optionen	I: größerer Schritt<br>II: Ausfallschritt nach vorne mit Armen 90° ABD (GHG)<br>III: Ausfallschritt mit Armen 90° Flexion (GHG) nach oben bringen und die Geschwindigkeit steigern
Wiederholungs-zahl	5 x jede Seite; jeweils 5 Sek. in der Endposition halten

Übung 7: Schritt zur Seite

**Übungssetting**	
Startposition	Stand: Füße hüftbreit auf dem Boden aufgestellt, Knie leicht gebeugt
Endposition	Rechter Fuß Schritt zur Seite rechts;<br>linker Fuß Schritt zur Seite links;
Steigerungs-optionen	I: größerer Schritt zur Seite<br>II: Ausfallschritt Zur Seite mit Armen 90° ABD (GHG)<br>III: Ausfallschritt zur Seite mit Armen 90° ABD (GHG) und Fuß zeigt in die Richtung des Ausfallschritts
Wiederholungs-zahl	5 x jede Seite; jeweils 5 Sek. in der Endposition halten

Übung 8: Schritt nach hinten

**Übungssetting**	
Startposition	Stand: Füße hüftbreit auf dem Boden aufgestellt, Knie leicht gebeugt
Endposition	Rechter Fuß Schritt nach hinten;<br>linker Fuß Schritt nach hinten
Steigerungs-optionen	I: größerer Schritt nach hinten<br>II: Ausfallschritt nach hinten mit den Armen 90° ABD (GHG)<br>III: Ausfallschritt nach hinten dabei die Armen in 180° Flexion (GHG) bringen
Wiederholungs-zahl	5 x jede Seite; jeweils 5 Sek. in der Endposition halten

Übung 9: Schritt diagonal nach vorne und hinten (Linke Diagonale)

**Übungssetting**	
Startposition	Stand: Füße hüftbreit auf dem Boden aufgestellt, Knie leicht gebeugt
Endposition	Rechter Fuß nach vorne; rechter Fuß nach hinten
Steigerungs-optionen	I: größerer Schritt nach vorne bzw. hinten<br>II: Ausfallschritt diagonal nach vorne bzw. hinten mit den Armen 90° ABD (GHG)<br>III: Ausfallschritt nach hinten dabei die Armen in 180° Flexion (GHG) bringen
Wiederholungs-zahl	5 x jede Seite; jeweils 5 Sek. in der Endposition halten

Übung 10: Schritt diagonal nach vorne und hinten (Rechte Diagonale)

**Übungssetting**	
Startposition	Stand: Füße hüftbreit auf dem Boden aufgestellt, Knie leicht gebeugt
Endposition	Linker Fuß nach vorne; linker Fuß nach hinten
Steigerungs-optionen	I: größerer Schritt nach vorne bzw. hinten<br>II: Ausfallschritt nach vorne bzw. hinten mit den Armen 90° ABD (GHG)<br>III: Ausfallschritt nach hinten dabei die Armen in 180° Flexion (GHG) bringen
Wiederholungs-zahl	5 x jede Seite; jeweils 5 Sek. in der Endposition halten

Übung 11: Kniebeugen

**Übungssetting**	
Startposition	Stand: Füße breitbeinig auf dem Boden aufgestellt, Knie gebeugt
Endposition	Gesäß tief bringen, Rücken aufrechte Position, Rotation Kopf (rechts & links)
Steigerungs-optionen	I: tiefer in die Kniebeuge gehen<br>II: Arme in U-Halte (GHG: 90° ABD, HUG: 90° Flex) seitlich neben dem Körper und mit der Kniebeuge nach vorne strecken (GHG: 90° Flex)<br>III: Schmalspuriger Stand in Kombination mit II
Wiederholungs-zahl	10 x; jeweils 5 Sek. in der Endposition halten

Übung 12: Bogenschießen seitlich

**Übungssetting**	
Startposition	Stand: Füße hüftbreit auf dem Boden aufgestellt, Knie gebeugt
Endposition	Rechter Arm in 90° ABD (GHG), linker Arm berührt die rechte Hand und geht langsam wieder zurück; linker Arm in 90° ABD (GHG), rechter Arm berührt die linke Hand und geht langsam wieder zurück
Steigerungs-optionen	I: weiter in die Knie<br>II: schmalspurig<br>III: Tandemstand
Wiederholungs-zahl	5 x jede Seite; jeweils 5 Sek. in der Endposition halten

Übung 13: Kniebeugen breitbeinig, Kopf Flexion, Extension, Rotation

**Übungssetting**	
Startposition	Stand: Füße breitbeinig auf dem Boden aufgestellt, Knie gebeugt
Endposition	Gesäß tief bringen, Rücken in aufrechter Position, Flexion und Extension des Kopfes
Steigerungs-optionen	I: Arme 180° ABD (GHG), Flexion und Extension Kopf<br>IIa): Rechten Arm in 90° ABD (GHG) führen, linken Arm in 180° ABD (GHG) halten<br>IIb): Linken Arm in 90° ABD (GHG) führen, rechten Arm in 180° ABD (GHG) halten
Wiederholungs-zahl	5 x mit jedem Arm; jeweils 5 Sek. in der Endposition halten

Übung 14: Kniebeugen schmalspurig, Kopf Flexion und Extension, Rotation

**Übungssetting**	
Startposition	Stand: Füße schmalspurig auf dem Boden aufgestellt, Knie gebeugt
Endposition	Gesäß tief bringen, Rücken in aufrechter Position, Flexion und Extension des Kopfes
Steigerungs-optionen	I: Arme 180° ABD (GHG), Flexion und Extension Kopf<br>IIa): Rechten Arm in 90° ABD (GHG) führen, linken Arm in 180° ABD (GHG) halten<br>IIb): Linken Arm in 90° ABD (GHG) führen, rechten Arm in 180° ABD (GHG) halten
Wiederholungs-zahl	5 x mit jedem Arm; jeweils 5 Sek. in der Endposition halten

Übung 15: Knie anheben

**Übungssetting**	
Startposition	Stand: Füße hüftbreit auf dem Boden aufgestellt, Knie leicht gebeugt
Endposition	Standbein links stabil, rechtes Bein in 90° Hüftflexion bringen;<br>Standbein rechts stabil, linkes Bein in 90° Hüftflexion bringen
Steigerungs-optionen	I: beide Arme in 90° ABD (GHG) bringen<br>II: zusätzlich mit der Bewegung den Kopf in Flexion bringen<br>III: die Geschwindigkeit erhöhen
Wiederholungs-zahl	5 x jede Seite; jeweils 5 Sek. in der Endposition halten

Übung 16: Füße vom Boden abheben

**Übungssetting**	
Startposition	Stand: Füße in einen breitbeinigen Stand bringen, Knie leicht gebeugt
Endposition	Standbein links stabil, rechten Fuß leicht vom Boden abheben;<br>Standbein rechts stabil, linken Fuß leicht vom Boden abheben;
Steigerungs-optionen	I: beide Arme in 90° (GHG) ABD bringen, mit der Bewegung den Kopf nach rechts bzw. links rotieren<br>II: mit der Bewegung die Arme weit nach oben schwingen (GHG: 180° Flex)<br>III: mit der Bewegung die Arme versetzt nach vorne bzw. hinten schwingen (rechter Fuß, linker Arm nach vorne, rechter Arm nach hinten und umgekehrt)
Wiederholungs-zahl	5 x jede Seite; jeweils 5 Sek. in der Endposition halten

Übung 17: Buchstabe Z

**Übungssetting**	
Startposition	Stand: Füße in einen hüftbreiten Stand bringen, Knie leicht gebeugt
Endposition	Auf dem Boden eine gedachtes Z gehen; Schritt nach rechts, zweites Bein dazustellen; Schritt diagonal nach hinten links, zweites Bein dazustellen; Schritt nach rechts, zweites Bein dazustellen
Steigerungs-optionen	I: gedachtes Z in umgekehrter Schrittfolge abgehen
Wiederholungs-zahl	5 x

Übung 18: Buchstabe M

**Übungssetting**	
Startposition	Stand: Füße in einen hüftbreiten Stand bringen, Knie leicht gebeugt
Endposition	Auf dem Boden eine gedachtes M gehen; Schritt nach vorne, zweites Bein dazustellen; Schritt diagonal nach hinten rechts, zweites Bein dazustellen; Schritt diagonal nach vorne rechts, zweites Bein dazustellen; Schritt nach hinten, zweites Bein dazustellen
Steigerungs-optionen	I: gedachtes M in umgekehrter Schrittfolge abgehen
Wiederholungs-zahl	5 x

Übung 19: Buchstabe N

**Übungssetting**	
Startposition	Stand: Füße in einen hüftbreiten Stand bringen, Knie leicht gebeugt
Endposition	Auf dem Boden eine gedachtes N gehen; Schritt nach vorne, zweites Bein dazustellen; Schritt diagonal nach hinten rechts, zweites Bein dazustellen; Schritt nach vorne, zweites Bein dazustellen
Steigerungs-optionen	I: gedachtes N in umgekehrter Schrittfolge abgehen
Wiederholungs-zahl	5 x

Übung 20: Buchstabe W umgedreht

**Übungssetting**	
Startposition	Stand: Füße in einen hüftbreiten Stand bringen, Knie leicht gebeugt
Endposition	Auf dem Boden eine gedachtes W gehen; Schritt diagonal nach hinten rechts, zweites Bein dazustellen; Schritt diagonal nach vorne rechts, zweites Bein dazustellen; Schritt diagonal nach hinten rechts, zweites Bein dazustellen; Schritt diagonal nach vorne rechts, zweites Bein dazustellen
Steigerungs-optionen	I: gedachtes W in umgekehrter Schrittfolge abgehen
Wiederholungs-zahl	5 x

Cool-Down/Abwärmen

Übung 21: Schultern kreisen

**Übungssetting**	
Startposition	Entspannt auf dem Stuhl sitzen
Endposition	Schultern vorwärts und rückwärts kreisen

Übung 22: Arme lockern

**Übungssetting**	
Startposition	Stand: Füße in einen hüftbreiten Stand bringen, Knie leicht gebeugt<br>Ggf. Sitz auf einem Hocker/Stuhl im vorderen Bereich;<br>Schultern locker, Arme neben dem Körper
Endposition	Arme mit der Einatmung weit nach oben anheben; mit der Ausatmung locker nach unten schwingen

Übung 23: BWS lösen

**Übungssetting**	
Startposition	Entspannt auf dem Stuhl sitzen
Endposition	Arme 90° (GHG) ABD; Rotation nach rechts; Rotation links

Übung 24: Kopf drehen

**Übungssetting**	
Startposition	Entspannt auf dem Stuhl sitzen, Augen geschlossen
Endposition	Kopf entspannt nach vorne, hinten, zur Seite rotieren

Studientagebuch – Woche 1

Vom ………………. bis ……………….

TN-ID: …………………………………..

**An welchen Tagen hat das Training stattgefunden?**	**Mo**	**Di**	**Mi**	**Do**	**Fr**
	☐	☐	☐	☐	☐

**Welche Übungen wurden durchgeführt? Bitte die Nummer der Übungen angeben**<br>**(z.B.: Übung 1, I, A)**	Übung (Nummer)	Schwierigkeitsgrad (I-III, A-C)	Übung (Nummer)	Schwierigkeitsgrad (I-III, A-C)

**Gab es beim Ausführen der Übungen Schwierigkeiten?**	ja	nein
	☐	☐

Wenn ja:

- **Notieren Sie die Nummer der Übung**
- **Notieren Sie kurz die Schwierigkeit**

**Angaben zu eventuellen Stürzen**	Anzahl der Stürze	Grund des Sturzes (z.B. Schwindel, Glatteis, Stolpern, etc.)
**Während des Trainings**		
**Außerhalb des Trainings**		

Studientagebuch – Woche 2

Vom ………………. bis ………………

TN-ID: …………………………………..

An welchen Tagen hat das Training stattgefunden?	**Mo**	**Di**	**Mi**	**Do**	**Fr**
	☐	☐	☐	☐	☐

Welche Übungen wurden durchgeführt? Bitte die Nummer der Übungen angeben<br>(z.B.: Übung 1, I, A)	Übung (Nummer)	Schwierigkeitsgrad (I-III, A-C)	Übung (Nummer)	Schwierigkeitsgrad (I-III, A-C)

Gab es beim Ausführen der Übungen Schwierigkeiten?	ja	nein
	☐	☐

Wenn ja:

- **Notieren Sie die Nummer der Übung**
- **Notieren Sie kurz die Schwierigkeit**

**Angaben zu eventuellen Stürzen**	Anzahl der Stürze	Grund des Sturzes (z.B. Schwindel, Glatteis, Stolpern, etc.)
**Während des Trainings**		
**Außerhalb des Trainings**		

Studientagebuch – Woche 3

Vom ………………. bis ……………….

TN-ID: …………………………………..

**An welchen Tagen hat das Training stattgefunden?**	**Mo**	**Di**	**Mi**	**Do**	**Fr**
	☐	☐	☐	☐	☐

**Welche Übungen wurden durchgeführt? Bitte die Nummer der Übungen angeben**<br>**(z.B.: Übung 1, I, A)**	Übung (Nummer)	Schwierigkeitsgrad (I-III, A-C)	Übung (Nummer)	Schwierigkeitsgrad (I-III, A-C)

**Gab es beim Ausführen der Übungen Schwierigkeiten?**	ja	nein
	☐	☐

Wenn ja:

- **Notieren Sie die Nummer der Übung**
- **Notieren Sie kurz die Schwierigkeit**

	Anzahl der Stürze	Grund des Sturzes (z.B. Schwindel, Glatteis, Stolpern, etc.)
**Angaben zu eventuellen Stürzen**		
**Während des Trainings**		
**Außerhalb des Trainings**		

Studientagebuch – Woche 4

Vom ………………. bis ………………

TN-ID: …………………………………..

	**Mo**	**Di**	**Mi**	**Do**	**Fr**
**An welchen Tagen hat das Training stattgefunden?**	☐	☐	☐	☐	☐

	Übung (Nummer)	Schwierigkeitsgrad (I-III, A-C)	Übung (Nummer)	Schwierigkeitsgrad (I-III, A-C)
**Welche Übungen wurden durchgeführt? Bitte die Nummer der Übungen angeben**<br>**(z.B.: Übung 1, I, A)**				

	ja	nein

**Gab es beim Ausführen der Übungen Schwierigkeiten?**		
**Wenn ja:**<br>- **Notieren Sie die Nummer der Übung**<br>- **Notieren Sie kurz die Schwierigkeit**		
**Angaben zu eventuellen Stürzen**	Anzahl der Stürze	Grund des Sturzes (z.B. Schwindel, Glatteis, Stolpern, etc.)
**Während des Trainings**		
**Außerhalb des Trainings**		
