# Supplementary material for: Effects of activity-oriented physiotherapy with and without eye movement training on dynamic balance, functional mobility, and eye movements in patients with Parkinson’s disease: An assessor-blinded randomised controlled pilot trial
Source: PLoS One. 2024 Jun 14;19(6):e0304788. doi: 10.1371/journal.pone.0304788 (PMC11178185; doi:10.1371/journal.pone.0304788)
Supplement: S2 Data — (PDF) [file pone.0304788.s009.pdf]

TEST PLAN (STUDY PROTOCOL)

Version: 1.1, from 27.03.2021

Replaces version: 1.0

Amendment No.:-

Study title: Effects of an activity-based physiotherapy training programme with and without gaze movement training on dynamic balance and fall risk in people with Idiopathic Parkinson's Syndrome: a pilot randomised controlled trial.

Study title Effects of an activity-oriented physiotherapy exercise programme with and without eye movement training on dynamic balance and fall risk in people with Parkinson's disease: a randomized controlled pilot trial

Short title: **APEM-PD Pilot**

Study design: Prospective double-blind randomised controlled pilot study

Sponsor: [redacted]

DRKS ID.: As soon as the approval by the Ethics Committee has been given, registration in the DRKS or another primary register according to the WHO criteria is applied for.

Applicant: [redacted]

Document history

Version	Date	Amendment no.	Author(s)	Changes/ Reason
1.0	02.02.2021	-	[redacted]	First version
1.1	27.03.2021	-	[redacted]	Second version

Persons/institutions involved

Function	Name & Contact
Sponsor	[redacted]
Principal Investigator	[redacted]
Investigator	[redacted]
Biometrician	[redacted]

Participating study centre

**A 01**	**Clinic for Rehabilitation Münster,** [redacted]

Signature page sponsor

Study title:	Effects of an activity-based physiotherapy training programme with and without gaze movement training on dynamic balance and fall risk in people with Idiopathic Parkinson's Syndrome: a pilot randomised controlled trial.
Short title	APEM-PD Pilot

The present protocol has been subjected to a critical review. The content is in accordance with the current risk-benefit assessment for the methods described and with the moral, ethical and scientific principles of the latest version of the Declaration of Helsinki and local laws and regulations.

[redacted]	
**Sponsor**	**Place, date, signature**

The above signatory confirms that he/she has read the present protocol and confirms that the protocol contains all the necessary information required for the conduct of the study. The undersigned further confirms that the study will be conducted in accordance with this protocol. It is agreed that any information not previously disclosed will be held in the strictest confidence.

Signature Page Principal Investigator and Investigator

Study title:	Effects of an activity-based physiotherapy training programme with and without gaze movement training on dynamic balance and fall risk in people with Idiopathic Parkinson's Syndrome: a pilot randomised controlled trial.
Short title	APEM-PD Pilot

The present protocol has been subjected to a critical review. The content is in accordance with the current risk-benefit assessment for the methods described and with the moral, ethical and scientific principles of the latest version of the Declaration of Helsinki and local laws and regulations.

[redacted]<br><br>**Principal Investigator**	______________________<br>**Place, date, signature**
[redacted]<br><br>**Investigator**	______________________<br>**Place, date, signature**

The above signatory confirms that he/she has read the present protocol and confirms that the protocol contains all the necessary information required for the conduct of the study. The undersigned further confirms that the study will be conducted in accordance with this protocol. It is agreed that any information not previously disclosed will be held in the strictest confidence.

Signature page Biometrician

Study title:	Effects of an activity-based physiotherapy training programme with and without gaze movement training on dynamic balance and fall risk in people with Idiopathic Parkinson's Syndrome: a pilot randomised controlled trial.
Short title	APEM-PD Pilot

The present protocol has been subjected to a critical review. The content is in accordance with the current risk-benefit assessment for the methods described and with the moral, ethical and scientific principles of the latest version of the Declaration of Helsinki and local laws and regulations.

[redacted]	
**Biometrician**	______________________________ **Place, date, signature**

The undersigned confirms that he/she has read the present protocol and confirms that the protocol contains all the necessary information required for the conduct of the study. The undersigned further confirms that the study will be conducted in accordance with this protocol. It is agreed that any information not previously disclosed will be held in the strictest confidence.

Funding

This study is an academic self-study that receives no external financial support.

Table of contents

Participating study centre 3
Signature Page Sponsor 4
Signature Page Coordinating Examiner and Examiner 5
Signature page Biometrician 6
1. Introduction 20
1.1 Background to the study 20
1.2 Necessity of conducting a study 21
1.3 Risk-benefit assessment 22
2 Study objectives and hypotheses 23
2.1 Purpose of study 23
2.2 Research question 23
2.3 Study objectives 23
2.4 Null hypotheses 24
2.5 Alternative hypotheses 24
3 Study description 24
3.1 Study design 24
3.2 Study centre 24
3.3 Competent Ethics Committee 25
3.4 Registration of the study 25
3.5 Schedule 25
4 Methods 27
4.1 Study participants 27
4.1.1 Participant recruitment and study inclusion 27
4.1.2 Study population 27
4.1.3 Randomisation and concealed allocation 28
4.1.4 Blinding 29
4.1.5 Unblinding 29
4.2 Intervention 29
4.2.1 Treatment scheme 29
4.2.2 Intervention 30
4.3 Endpoints and data collection 33
4.3.1 Demographic and disease-specific data 33
4.3.2 Data collection and investigators 34
4.3.3 Primary and secondary endpoints and survey instruments 34
4.4 Implementation and psychometric criteria of clinical assessments 37

4.4.1 Functional Gait Assessment 37
4.4.2 Measurement of eye movements during items 5, 6 and 10 of the FGA . 38
4.4.3 Mini Mental Status Test (MMST) 38
4.4.4 Timed-Up-and-Go (TUG) 38
4.4.5 Timed-Up-and-Go with manual dual task (TUGman) 39
4.4.6 Berg Balance Scale (BBS) 40
4.4.7 Four Step Square Test (FSST) 41
4.4.8 10-metre walk test 41
4.4.9 Parkinson's Disease Questionnaire (PDQ-39) 42
4.4.10 Freezing of Gait Questionnaire (FOG-Q) 45
4.4.11 Falls Efficacy Scale- International Version (FES-I) 46
4.4.12 Beck Depression Inventory (BDI-II) 47
4.4.13 Fall rate 47
4.4.14 Feasibility of a larger study 48
4.4.15 Measurement of eye movements during the intervention 49
4.5 Adverse events, side effects 49
4.5.1 Definitions 49
4.5.2 Assessment and documentation 51
4.5.3 Reporting of serious incidents (SUE/SNW) 52
5 Data documentation, management and evaluation 52
5.1 Methods of data collection 52
5.2 Documentation 53
5.2.1 Source data and documents 53
5.2.2 Survey questionnaire (CRF) 53
5.3 Follow-up treatment of patients after completion of the trial 53
5.4 Drop-out of patients (drop-out) 53
5.5 Premature termination of the clinical trial 54
5.6 Data analysis 55
5.6.1 Statistical data analysis 55
5.6.2 Qualitative data analysis 57
5.7 Auditor and data management 57
5.7.1 Retention of study records, data storage & deletion 57
5.7.2 Data management 58
5.7.3 Dealing with queries 59
6 Ethical, legal and administrative aspects 59
6.1 Compliance with ethical and regulatory requirements 59
6.2 Vote of the Ethics Committee 59

6.3 Patient information and consent form ... 59
6.4 Patient insurance ... 60
6.5 Data protection and confidentiality ... 60
7 Changes during the course of the study ... 61
7.1 Amendments to the protocol ... 61
7.2 Deviations from the test plan ... 62
7.3 Informing the study participants about the results of the study ... 62
8 Funding, conflicts of interest and compensation of participants ... 62
8.1 Funding of the study ... 62
8.2 Possible conflicts of interest ... 62
8.3 Fees and compensation for participants ... 62
9 Bibliography ... **Fehler! Textmarke nicht definiert.**

Study synopsis

**Principal Investigator**	[redacted]
**Sponsor**	[redacted]
**Title**	Effects of an activity-oriented physiotherapy training programme with and without gaze movement training on dynamic balance and fall risk in people with Idiopathic Parkinson's Syndrome: a randomised controlled pilot study
**Short title**	APEM-PD Pilot
**DRKS ID**	As soon as the Ethics Committee has given its approval, registration in the DRKS or another primary registry is applied for in accordance with the WHO criteria.
**Study objective**	The study objective is to investigate the effects of an activity-based physiotherapy training programme with eye movement training compared to an activity-based physiotherapy training programme without eye movement training on dynamic balance, mobility with and without multi-task, static and dynamic balance walking speed, health-related quality of life, freezing of gait, fall-associated self-efficacy, depression and fall rate. The aim is also to explore the change in eye movements (saccade velocity, amplitude, latency, fixation duration) over the intervention period and to evaluate the feasibility of a larger randomised controlled trial using predefined parameters.
**Primary endpoints**	1.1 Dynamic balance measured by the Functional Gait Assessment (FGA)<br>1.2 Items 5, 6 and 10 of the FGA are additionally performed with eye-tracking glasses (Tobii Pro Glasses 3) for mobile measurement of eye movements (saccade velocity, amplitude, latency, fixation duration).
**Secondary endpoints**	2.0 Screening for cognitive function or impairment: Mini-Mental State Test (MMST)

	2.1 Functional mobility measured with the Timed-Up-and-Go (TUG)<br>2.2 Functional mobility with dual task measured with the Timed-Up-and-Go with Manual Dual Task (TUGman)<br>2.3 Static and dynamic balance measured by the Berg Balance Scale (BBS)<br>2.4 Dynamic equilibrium measured by the Four Square Step Test (FSST)<br>2.5 Walking speed measured with the 10-Metre Walk Test (10MWT)<br>2.6 Health-related quality of life measured with the Parkinson's Disease Questionnaire-39 (PDQ-39)<br>2.7 Freezing of Gait, measured with the Freezing of Gait Questionnaire (FOGQ)<br>2.8 Falls-associated self-efficacy measured with the Falls Efficacy Scale- International Version (FES-I)<br>2.9 Depression measured by the Beck Depression Inventory (BDI-II)<br>2.10 Fall rate 3 months before study inclusion, during the 4-week intervention period and during the 4-week follow-up period<br>2.11 Mobile recording of eye movements (saccade velocity, amplitude, latency, fixation duration) during the intervention (1x weekly, training at the end of the week, for 12 min each, during exercise 2, 3, 4, 6 and 7)<br>2.12 Feasibility of a larger study (recruitment, retention, adherence rates, adverse events, acceptance of the intervention in the intervention group (measured by focus groups in intervention group 1 on postintervention).
**Study design**	Prospective double-blind randomised controlled pilot study
**Study population**	Adults with Idiopathic Parkinson's Syndrome and Mild to Moderate Limitation (Hoehn & Yahr Stages 1-3)
**Number of cases**	34 People with Idiopathic Parkinson's Syndrome

**Intervention**	The intervention takes place within the framework of the inpatient rehabilitation of the patients and is carried out in addition to the usual rehabilitation.<br><br>*Intervention group (group 1):* Activity-oriented physiotherapeutic training programme with eye movement training; 30 min, 4x per week supervised by trained physiotherapists. A total of 4 units á 30 min. per week for 4 weeks.<br><br>*Control group (group 2):* Standard physiotherapeutic care: activity-oriented physiotherapeutic training programme without gaze movement training; 30 min, supervised 4x per week, by trained physiotherapists. A total of 4 units á 30 min. per week for 4 weeks.
**Study Centre**	Reha Zentrum Münster Betriebs GmbH, Gröben 700, 6232 Münster
**Inclusion criteria**	Men and women with Idiopathic Parkinson's Syndrome using the UK Brain Bank criteria. (Hughes et al., 1992)(stages 1-3 according to Hoehn & Yahr) with ON medication. (Hoehn and Yahr, 1967), age 30-80 years, any ethnicity, ability to walk without an assistant, Mini-Mental Status Test (MMST) ≥ 24/30 (Tombaugh and McIntyre, 1992)Stable dosage of dopaminergic replacement therapy at least 3 weeks before the start of the study or still without dopaminergic treatment for the duration of the study intervention, written and spoken German.
**Exclusion criteria**	Concomitant disease(s) (such as malignant diseases, other neurological, orthopaedic, cardiac or psychiatric diseases, major depression, dementia), photosensitivity, non-parkinsonian gait disorder (e.g. due to musculoskeletal symptoms), recent surgery (general and ocular), intraocular implants, strabismus, nystagmus, prominent drooping eyelids, untreated pain, uncorrected visual or auditory impairment that would interfere with training or examinations, Pregnancy, recent deep brain stimulation (THS) or change in parameters of THS within the last

	year, severe motor fluctuations, initiation of new dopaminergic medication or adjustment of the same within the study period is not provided; In the sense of a real-life setting, this decision is incumbent on the investigator.<br><br>For the correction of defective vision, suitable corrective lenses are used with the mobile Tobii Eye Tracker.
**Statistical analysis**	Statistical data analysis is performed using IBM SPSS software, version 26.0 (IBM Corporation, Armonk, NY, USA) and Tobii Pro Lab Analyer (Tobii, Danderyd, Sweden). The statistical significance level is defined by a p-value of <0.05. An attempt is made to avoid missing data by inspecting the questionnaires after completion and, in case of unanswered items, asking the study participants to complete them. The number of missing data is noted; due to the less sensitive topic of the study, it is assumed that missing variable values can be assigned to "missing completely at random" or "missing at random". An intention-to-treat analysis is performed for all cases analysed in their originally assigned group.<br><br>Descriptive statistics are used for demographic data and primary and secondary outcomes. Counted and nominal scaled data (gender, fall rate, recruitment, retention and adherence rate, number of missing data if applicable, number of adverse events) are reported as whole numbers or percentages. Ordinal scaled variables (MMSE, UPDRS, H & Y, FGA, BBS, PDQ-39, FOGQ, FES-I, BDI-II) are expressed as median (minimum, maximum or interquartile range) and metric variables (TUG, TUGman, FSST, 10MWT, eye movements: Saccade velocity, amplitude, latency, fixation duration) by mean (95% confidence interval, CI or standard deviation, SD). The data is also presented graphically (e.g. by bar charts, box plot or line graphs).<br><br>The eligibility rate (%) is the percentage of patients suitable for the study after applying the inclusion and exclusion criteria from the Parkinson's patients treated at the Reha Zentrum Münster

during the study period.

The recruitment rate (%) is determined as follows: $(N_{Consent}/N_{suitable})*100$; where $N_{consent}$ is the number of participants who signed the informed consent form; $N_{suitable}$ is the number of patients suitable for the study based on the inclusion and exclusion criteria.

The retention rate (%) is calculated as follows: $(N_{completed/Total})*100$; where $N_{completed}$ is the number of participants who completed the study; N_{total} is the total number of participants in the study.

The adherence rate (%) is determined as follows: (Actual number of exercise sessions/planned number of training sessions)*100 (Osterberg & Blaschke, 2005).

Eligibility, recruitment, retention and adherence rates are calculated using the Wilson 'score' method propagated by Newcombe, together with its 95% CI (Newcombe, 1998)In the case of a proportion close to 0 or 1, a Poisson approximation according to Brown is used. (Brown, Cai, & DasGupta, 2001)..

A test for statistically significant differences between groups at baseline is performed: for nominal data (eligibility, recruitment, retention, adherence rates, gender) the Fisher's Exact Test is used, for ordinal data (age, H & Y, UPDRS, FGA, MMST, BBS, PDQ-39, FOGQ, FES-I, BDI-II) the Mann Whitney-U test and for metric data (TUG, TUGman, FSST, 10MWT, eye movements: Saccade velocity, amplitude, latency, fixation duration) the T-test for independent samples. Preliminary inferential statistical analysis to detect trends in intervention effectiveness and as a basis for sample size calculation for a randomised controlled trial with sufficient power of at least 80% (using mean (SD) differences between groups) will be performed. For inferential statistics, metric data are first tested for normal distribution and significant data outliers using the Shapiro-Wilk test, Q-Q plots and histograms. Non-normally distributed data are transformed using suitable transformation (e.g. ln, square root), checked

	again and analysed non-parametrically if normal distribution is missing.<br><br>Differences between the two groups and test times are calculated for ordinal data as follows: New variables are generated from the difference between the post-intervention and baseline data. The difference between group 1 and 2 is determined using the Mann Whitney-U test. For the ordinal variables FES-I and FOGQ, which are also collected at the follow-up call, a Kruskal Wallis test is performed across all groups and test time points. Changes in fall rate (nominal) between baseline, post-intervention and follow-up will be calculated using Chi-Square test. Metric data will be tested for homogeneity of variance (Levene test) as the basic assumption of a 2 x 2 ANOVA, with "time (baseline, post-intervention)" as the within-factor and "group (1, 2)" as the between-factor. If there is no homogeneity of variance, an alternative F-statistic (e.g. according to Welch or Brown-Forsythe) is used. If the basic assumption of sphericity is not fulfilled, corrected values are used, e.g. according to Greenhouse-Geisser or Huynh-Feldt. To calculate the differences between the groups and measurement times, a 2 x 2 ANOVA (analysis of variance) is performed, followed by pairwise comparisons (Bonferroni correction for all comparisons) between the groups at measurement times 1 and 2 if statistical significance is present. In the case of failure of the adaptive measures, non-parametric tests are used as described for ordinal scaled data. Effect size is reported using partial Eta squared and standardised effect size.
**Timetable**	Study-related:<br>Recruitment time: 8 months<br>15.04.2021 Planned start: First Patient First Visit (FPFV)<br>15.11.2021 Last Patient First Visit (LPFV)/Last Subject In (LSI)<br>14.12.2021 Planned end: Last Patient Last Visit (LPLV)/Last Subject Out (LSO)

	Patient-related:<br>Active intervention duration: 4 weeks<br>After inclusion, the duration of each participant's participation corresponds to 8 weeks (baseline survey, 4-week intervention, post-intervention testing, focus group only for participants in intervention group 1, follow-up call 4 weeks after end of intervention).

**Funding**	This clinical study is a self-funded, academic study of the Reha Zentrum Münster, [redacted], [redacted].
**GCP Statement**	The study was planned in accordance with the requirements of the Tyrolean Hospitals Act (Tir KAG), the Declaration of Helsinki and the ICH-E6 guidelines, the OeAWI guidelines for good scientific practice, as well as the requirements of the General Data Protection Regulation (DSGVO) and the Austrian Data Protection Act (DSG).

List of abbreviations

ABC Scale	Activities-specific Balance Confidence Scale
ADLs	Activities of daily living
BBS	Berg Balance Scale
BDI-II	Beck Depression Inventory
DSG	Austrian Data Protection Act
GDPR	General Data Protection Regulation
FES-I	Falls Efficacy Scale
FGA	Functional Gait Assessment
FOGQ	Freezing of Gait Questionnaire
ID	Identification number
LPLV	Last Patient Last Visit
QL	Quality of life
LSI	Last subject in
LSO	Last subject out
MDC	Minimum detectable change
MMST	Mini Mental Status Test
ND	Not documented
NW	Side effect
P	Break
PDQ-39	Parkinson's Disease Questionnaire-39
PI	Post Intervention
R&TTE	Radio equipment and telecommunications terminal equipment (Radio and Telecommunications Terminal Equipment Directive)
Scr	Screening
SD	Standard deviation
SNW	Serious side effect
SUE	Serious adverse event
THS	Deep brain stimulation
Tir CISA	Tyrolean Hospitals Act
TUG	Timed-Up-and-Go
TUGman	Timed-Up-and-Go with manual dual task
UE	Adverse event
UPDRS	Unified Parkinson's Disease Rating Scale
10MWT	10-Minute Walk Test

1. Introduction

1.1 Background of the study

Idiopathic Parkinson's syndrome is one of the most common neurological conditions affecting people over the age of sixty, with a prevalence of 6.1 million people worldwide (as of 2016). Since 1990, there has been an increase of 14.5%. ("Global, regional, and national burden of Parkinson's disease, 1990-2016: a systematic analysis for the Global Burden of Disease Study 2016.," 2018).. It is the fastest growing neurological disease in the world (Tönges et al., 2019). In people over 65 years of age, the prevalence is 16,226 cases in Austria and 260,817 cases in Germany (Andlin-Sobocki et al., 2005).. The costs per case amount to 9291 euros in Austria and 11138 euros in Germany (Andlin-Sobocki et al., 2005). (Andlin-Sobocki et al., 2005)..

The cardinal symptoms of idiopathic Parkinson's syndrome are akinesia, rigour, tremor at rest and postural instability in varying degrees. (Keus, S. et al., 2014). Furthermore, oculomotor disorders such as reduced saccadic velocity may occur. (Matsumoto et al., 2011).. Decreased saccade amplitude may be one of the main causes of limitations in the perception of the environment. (Matsumoto et al., 2011).. Changes in saccade parameters in combination with bradykinesis lead to an increased risk of falls in everyday life in those affected (Cucca et al., 2011). (Cucca et al., 2018). This, in turn, can lead to limitations in independence in the activities of daily living (ADLs), which negatively influences the participation of those affected (Keus, S. et al., 2014)..

Alcock and colleagues (2020) show in their study that visual dysfunction correlates with and increases the risk of falls in people with Parkinson's disease. In the treatment of people with Parkinson's disease, visual dysfunction should be appropriately addressed to promote coordination and reduce the risk of falls. (Ambati et al., 2016).
Stuart and colleagues (2018) demonstrated that visual cues improve saccade frequency in people with idiopathic Parkinson's disease. Furthermore, Baker and colleagues (2020) achieved a change in the sequence of first-response body segments when turning during walking through the use of visual cues. As a result, the subjects with idiopathic Parkinson's syndrome regained anticipatory adaptation of eye movements. (Baker et al., 2020).
These findings suggest a positive influence on saccade parameters through targeted eye movement training. Such training potentially also leads to an improved perception of the environment, which could positively influence gait safety and thus reduce the risk of falls.

Neurological physiotherapy for Idiopathic Parkinson's Syndrome at the Reha Zentrum Münster is evidence-based and follows international guidelines for rehabilitation (Keus, S. et al., 2014; Grimes et al., 2019; National Institute for Health and Care Excellence (Great Britain), 2017).. It has not yet been investigated whether additional gaze movement training achieves an additional benefit with regard to the dynamic mobility and fall rate of patients.

1.2 Necessity of conducting a study

The current state of research in the field of interventions to improve oculomotor function shows partly controversial results. Matsumoto and colleagues describe a stronger and longer fixation on obstacles in people with idiopathic Parkinson's syndrome compared to healthy people of the same age in the control group. (Matsumoto et al., 2011).. Barbieri and colleagues (2018) describe reduced fixation on an obstacle and prolonged fixation on the ground in individuals with Idiopathic Parkinson's Syndrome. (Barbieri et al., 2018; Reed-Jones & Powell, 2017)(Reed-Jones and Powell, 2017b). In addition, Matsumoto and colleagues find a lower saccade amplitude and a lower number of saccades, indicating a reduced ability to grasp objects and the environment. As a result, the participation of individuals with idiopathic Parkinson's syndrome is reduced due to impaired sensing ability. (Matsumoto et al., 2011)..

Cucca and colleagues (2018) found a correlation of visual ability with the quality of life of the study participants. Thus, improved visual ability could also increase the quality of life of people with idiopathic Parkinson's syndrome. (Cucca et al., 2018).
By 2040, up to 14.2 million people worldwide are predicted to have Idiopathic Parkinson's Syndrome. Due to the increasing prevalence, the need for care in inpatient and outpatient care structures will rise. (Tönges et al., 2019).. Therefore, it seems particularly important that there are interventions for both the inpatient and the outpatient sector to maintain the longest possible independence of those affected. In order to make an intervention as close to everyday life as possible, it must be adapted to the given care structures of the respective setting. In Austria, people with idiopathic Parkinson's syndrome have the option of inpatient rehabilitation every year or every two years. Therefore, it seems reasonable to integrate an intervention in the inpatient rehabilitation setting for 4 weeks and thus test it under "real-life" conditions. (Carpinella et al., 2017; Martinez-Martin et al., 2011; Silva de Lima et al., 2018)..

To develop the intervention for this clinical trial, the *Medical Research Council*'s (2006) guidelines for developing complex interventions were used to identify, among other things,

appropriate methods for investigating the research question (identifying the evidence, developing a theory and modelling the process and outcomes). (Craig, P. et al., 2006). Thus, this clinical study can add value to the future care of people with Idiopathic Parkinson's Syndrome. The preservation of independence and the reduction of the need for care of persons with Idiopathic Parkinson's Syndrome should be the overarching goals.

More than one third of falls in people with Idiopathic Parkinson's Syndrome occur as a result of tripping due to impaired vision as a contributing factor (Alcock et al., 2020). This highlights the relevance of fall prevention in maintaining independence in people with Idiopathic Parkinson's Syndrome. The correlation of visual dysfunction and an increased risk of falls suggests that targeted eye and gaze movement training can reduce the rate of falls. (Cucca et al., 2018). Improving the speed of eye and gaze fixation leads to better identification of obstacles, a reduction in gait insecurity and thereby a reduction in the risk of falls (Ambati et al., 2016). In the long term, the independence of people with idiopathic Parkinson's syndrome can be maintained in this way. (Ambati et al., 2016).

1.3 Risk-benefit assessment

The activity-oriented physiotherapeutic programme is basically an evidence-based treatment method based on international guidelines for the rehabilitation of idiopathic Parkinson's syndrome, which is guided by physiotherapists in the course of conventional rehabilitation. This physiotherapeutic measure takes place in both groups to the same extent in addition to the usual inpatient interdisciplinary rehabilitation at the Reha Zentrum Münster. The total amount of training during the rehabilitation stay and its intensity correspond to the cited international guidelines for rehabilitation in idiopathic Parkinson's syndrome. The eye movement training is carried out with the activity-oriented physiotherapeutic programme and is also guided by experienced and trained physiotherapists. Based on clinical experience and literature, it can be assumed that the study participants in the intervention group will benefit at least as much from the treatment as in the standard therapy group.

The intervention is initially carried out in a sitting position in order to achieve habituation to the eye movement training. It is not expected that falls will occur during the intervention as the patients are continuously under physiotherapeutic supervision. Dizziness or eye fatigue could be initially triggered by the training. (Camacho et al., 2019a). This risk is minimised by limiting the duration of treatment to four times 30 minutes per week. Eye movement training will not be performed continuously during the 30-minute period, but intermittently with activity-based training to avoid overload from the outset. The intensity and duration of the interventions are considered low impact. Before, during and after the intervention, any complaints and side

effects are asked about. Since the patients are in the Reha Zentrum Münster for the inpatient rehabilitation stay and the intervention is to be integrated into the daily therapy schedule, there is no additional burden in terms of travel. The expected benefit - the optimisation of treatment and a more efficient rehabilitation - is therefore, according to current medical knowledge, only a low health risk and only a low burden for the patients.

2 Study objectives and hypotheses

2.1 Purpose of study

The aim of the study is to investigate the effects of an activity-based physiotherapy training programme with eye movement training compared to an activity-based physiotherapy training programme without eye movement training on dynamic balance, mobility, walking speed, health-related quality of life, freezing of gait, fall-associated self-efficacy, depression and fall rate in people with Idiopathic Parkinson's Syndrome. The aim is also to explore the change in eye movements (saccade speed, amplitude, latency, fixation duration) over the intervention period and to evaluate the feasibility of a larger randomised controlled trial using predefined parameters.

2.2 Research question

The research question underlying the study is as follows:

"Is there a difference between the effects of an everyday physiotherapy exercise programme with versus without gaze movement training on dynamic balance, mobility, walking speed, health-related quality of life, freezing of gait, fall-associated self-efficacy, depression and fall rate in people with Idiopathic Parkinson's Syndrome?"

2.3 Study objectives

Thus, four **study objectives** are pursued to deepen the knowledge on effectiveness:

1. Assessment of the effects on dynamic balance (primary endpoint)
2. Investigating the feasibility of a larger randomised controlled trial
3. assessment of the effects on functional mobility with and without manual multi-task, static and dynamic balance, walking speed, health-related quality of life, freezing of gait, fall-associated self-efficacy, depression and fall rate (secondary endpoints).

4. evaluation of the change in eye movements during the intervention, measured once a week in the intervention group over the intervention period.

2.4 Null hypotheses

Primary null hypothesis: There is no difference between the effects of an everyday physiotherapy training programme with eye movement training and such a training programme without eye movement training on dynamic balance in people with idiopathic Parkinson's syndrome.

Secondary null hypotheses: There is no difference between the effects of an everyday physiotherapy training programme with gaze movement training and such a training programme without gaze movement training on functional mobility with and without manual multi-task, static balance, walking speed, health-related quality of life, freezing of gait, fall-associated self-efficacy, depression, fall rate and eye movements during dynamic balance tasks and specific training items in individuals with Idiopathic Parkinson's Syndrome.

2.5 Alternative hypotheses

Primary alternative hypothesis: There is a difference between the effects of an everyday physiotherapy training programme with eye movement training and such a training programme without eye movement training on dynamic balance in people with idiopathic Parkinson's syndrome.

Secondary alternative hypotheses: There is a difference between the effects of an everyday physiotherapy training programme with gaze movement training and such a training programme without gaze movement training on functional mobility with and without manual multi-task, static balance, walking speed, health-related quality of life, freezing of gait, fall-associated self-efficacy, depression, fall rate and eye movements during dynamic balance tasks and specific training items in individuals with Idiopathic Parkinson's Syndrome.

3 Study description

3.1 Study design

Prospective double-blind randomised controlled pilot study

3.2 Study Centre

Reha Zentrum Münster Betriebs GmbH, Gröben 700, 6232 Münster

3.3 Competent Ethics Committee

The ethics committee responsible for this study is the Ethics Committee of the Medical University of Innsbruck

3.4 Study registration

After receiving a positive vote from the ethics committees of the Medical University of Innsbruck, the study will be prospectively registered in the DRKS, ISRCTN or a comparable WHO-recognised registry.

3.5 Timetable

<u>Study-related:</u>
Recruitment period: 8 months
15.04.2021 Planned start: First Patient First Visit (FPFV)
15.11.2021 Last Patient First Visit (LPFV)/Last Subject In (LSI)
14.12.2021 Planned end: Last Patient Last Visit (LPLV)/Last Subject Out (LSO)

<u>Patient-related:</u>
Active intervention duration: 4 weeks

After inclusion, the duration of each participant's participation will be 8 weeks (baseline survey, 4-week intervention, post-intervention testing, focus group for intervention group 1 participants only, follow-up call 4 weeks after the end of the intervention). The total duration of the study will be 8 months.

Figure 1 presents the patient-related study process.

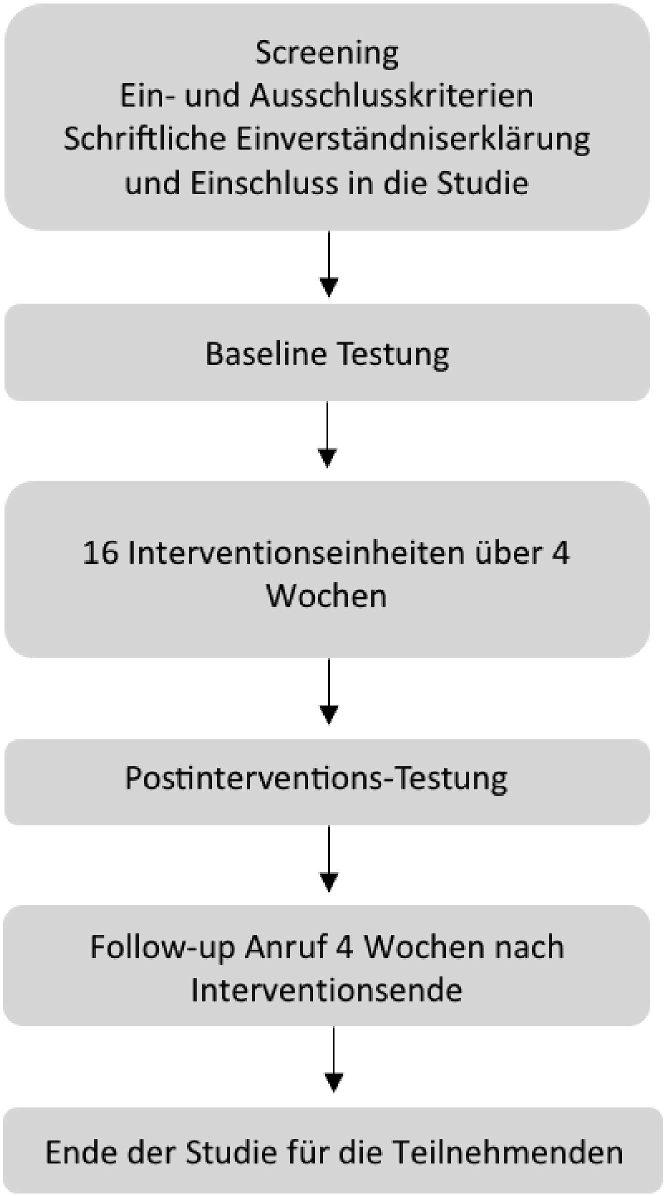

Figure 1: Patient-related study procedure

4 Methods

4.1 Study participants

4.1.1 Participant recruitment and study inclusion

All persons with Idiopathic Parkinson's Syndrome treated at the Reha Zentrum Münster during the recruitment period will be reviewed by the medical staff with regard to their suitability for study participation. All persons with idiopathic Parkinson's syndrome who meet the inclusion criteria of this study and do not fulfil any of the exclusion criteria will be informed about the study orally and in writing by the respective investigator. After determining a basic suitability (inclusion criteria) and the patient's consent, i.e. his/her interest in participating in the study, a screening is carried out. Using predefined cut-off scores of the Mini-Mental-Status-Test (MMST) (Folstein et al., 1975) ≥24/30 points, moderate to severe cognitive impairment is excluded. In the case of the absence of these exclusion criteria, study inclusion will be as described below.

After an appropriate reflection period, opportunity for questions and the patient's consent to participate, the information letter and patient consent form is then dated and signed by both the patient and the doctor. The investigator records the participation on a special patient identification list. This list is used to identify the patient at a later date and contains the patient's identification number (ID), full name, date of birth and date of inclusion in the clinical trial. The patient identification list remains at the trial site after completion of the trial. The ID will be entered with the date of inclusion in the randomisation list as described and prepared by an independent researcher.

4.1.2 Study population

4.1.2.1 Inclusion criteria

Men and women with Idiopathic Parkinson's Syndrome using the UK Brain Bank criteria. (Hughes et al., 1992)(stages 1-3 according to Hoehn & Yahr) with ON medication. (Hoehn and Yahr, 1967), age 30-80 years, any ethnicity, ability to walk without an assistant, Mini-Mental Status Test (MMST) ≥ 24/30 (Tombaugh and McIntyre, 1992)Stable dosage of dopaminergic

replacement therapy at least 3 weeks before the start of the study or still without dopaminergic treatment for the duration of the study intervention, written and spoken German.

4.1.2.2 Exclusion criteria

Concomitant disease(s) (such as malignant diseases, other neurological, orthopaedic, cardiac or psychiatric diseases, major depression, dementia), photosensitivity, non-parkinsonian gait disorder (e.g. due to musculoskeletal symptoms), recent surgery (general and ocular), intraocular implants, strabismus, nystagmus, prominent drooping eyelids, untreated pain, uncorrected visual or auditory impairment that would interfere with training or examinations, Pregnancy, recent deep brain stimulation (THS) or change in parameters of THS within the last year, severe motor fluctuations, initiation of new dopaminergic medication or adjustment of the same within the study period is not provided; In the sense of a real-life setting, this decision is incumbent on the investigator.

Corrective lenses that match the mobile eye tracker are used to correct defective vision.

4.1.2.3 Number of participants and sample size

For this study, 34 people with Idiopathic Parkinson's Syndrome will be included. Of the 34 people with Parkinson's disease, 17 will be randomised to intervention group 1 and 17 to control group 2.

According to Julious, participants per group are considered a minimum sample size for a pilot study. (Julious, 2005)while Browne specifies a number of participants of 30 for a two-arm study. (Browne, 1995). Therefore, to evaluate the feasibility criteria and preclinical effects of the experimental intervention compared to the conventional control intervention, a sample size of 34 is aimed for, based on 30 participants, including an expected failure rate of 10% and using the formula N=n/(1-(z/100)). Here, n is the number of participants (30) and z is the expected failure rate (10%).

4.1.3 Randomisation and concealed allocation

34 patients with Idiopathic Parkinson's Syndrome will be randomised into one of two groups using a 1:1 allocation ratio. For this purpose, a random number sequence generated by the randomisation software "Sealed Envelope" (https://www.sealedenvelope.com/) and sealed opaque envelopes are used. A stratified block randomisation (blocks of 4 and 6) with concealed allocation will be conducted by an independent researcher in the team who will not be involved in participant recruitment, instructions regarding the intervention or testing.

Stratification will be based on only one relevant predictive factor for change in dynamic mobility, namely degree of disability (H & Y stages 1 and 2; 3), due to the small sample size.

4.1.4 Blinding

The raters/test takers of the study will be blinded to the group assignment of the participants, and the participants will be blinded to the study hypotheses.

4.1.5 Unblinding

The planned time of regular unblinding is after completion of the study, specifically after finalisation of the data analysis.

4.2 Intervention

4.2.1 Treatment scheme

The study includes a screening, a baseline examination, 16 intervention sessions a' 30 min over 4 weeks, a post-intervention testing, a focus group for the participants of the intervention group and a follow-up call 4 weeks after the end of the intervention.

The duration and number of examinations and treatments (Table 1) will take place in the context of the patients' inpatient interdisciplinary rehabilitation. It is ensured that all study participants receive the same number of treatments in total. In addition, all participants must receive 8 30-min sessions of physiotherapy per week, of which 4 are reserved for the study intervention in group 1 or 2 and represent an additional measure. All therapists will be trained on the study interventions and instructions for participants in both groups and will therefore work according to the same principles.

Table 1: Treatment regimen of a (screened possible*) study participant

**Intervention**	**Dosage**	**Time**	**Duration**
Screening*	1x	After verification of suitability by the investigator and patient consent	10-15 min
Study inclusion			
Baseline investigation	1x	Day 0 after study inclusion	75-90 min
Intervention groups 1 and 2	16x	Start on day 1 after the baseline examination	30 min each

		4 x per week, 4 weeks in total	
Postintervention testing	1x	4 weeks after the start of the intervention	75-90 min
Focus group	1x	4 weeks after the start of the intervention, only for intervention group 1, at a time interval of >2 hours from post-intervention testing	45-60 min
Follow-up call	1x	4 weeks after end of intervention	15 min

*If one or more exclusion criteria are present after screening, the patient will not be included in the study.

4.2.2 Intervention

The intervention is carried out in addition to the therapy units of the inpatient rehabilitation at the Reha Zentrum Münster. All patient goals not addressed by the study intervention as well as neurotherapeutic therapy measures are thus carried out during the usual therapy units (e.g. auditory cueing, cognitive strategies, endurance training, specific posture training).

Intervention group (Group 1): Activity-oriented physiotherapeutic training programme with eye movement training; the training is evidence-based and oriented towards guidelines for rehabilitation in idiopathic Parkinson's syndrome (Keus, S. et al., 2014; Grimes et al., 2019; National Institute for Health and Care Excellence (Great Britain), 2017): for 30 min, 4x per week, for 4 weeks, supervised by the respective physiotherapists.

Control group (group 2): Activity-oriented physiotherapeutic training programme i. The standard care is evidence-based and oriented towards guidelines for rehabilitation in idiopathic Parkinson's syndrome (Keus, S. et al., 2014; Grimes et al., 2019; National Institute for Health and Care Excellence (Great Britain), 2017): for 30 min, 4x per week, for 4 weeks, supervised by the respective physiotherapists.

The training therefore includes an individualised training adapted to the Hoehn and Yahr stage, which is overall diverse and variable. The training characteristics listed below apply to the training in both intervention groups. The difference between the two groups is only the eye movement training in combination with the exercises described in more detail below.

- Application of the principles of motor learning (external learning focus, repetition, variability, progression, activity-oriented, goal-oriented)
- Specific functional balance training (static and dynamic balance training, e.g. increasingly moving beyond the body's stability limits, secured by the physiotherapist).
- Transfers such as seat-stand
- Functional exercises to improve joint mobility (e.g. trunk rotation with progressive increase to higher body position).
- Functional strength training
- Coordination training
- Fall prevention training
- Training large movement amplitudes with high intensity

The *activity-based physiotherapy training programme with eye movement training (group 1*) will consist of the following elements: An evidence-based routine programme that includes the parameters of motor learning. (Olson et al., 2019), specific functional balance training, improvement of joint mobility, fall prevention and functional independence (Keus, S. et al., 2014) is combined with gaze movement training. The selection of the parameters of gaze movement training was based on various studies that have already investigated gaze movement training in other populations. (Camacho et al., 2019b; Knox and Wolohan, 2015; Matsumoto et al., 2011).. These include the intensity, the practice times for the interventions (in the ON phase) and the distance of the trainee from the wall where a practice poster with a crosshair and different fixation points is placed.

The gaze movement training is intended to direct the focus of the evidence-based activity-oriented routine programme carried out in rehab towards gaze fixation and expansion of saccade amplitude of persons with Idiopathic Parkinson's Syndrome. This means that in addition to physiotherapy exercises to increase coordination, strength and strength endurance, gaze fixation and gaze movement exercises will be trained. These will be based on the parameters of motor learning. (Olson et al., 2019) These exercises are progressively increased using the parameters of motor learning (Olson et al., 2019) and are carried out in combination with the exercises of balance and fall prevention training. The respective physiotherapists receive training to ensure standardised implementation of the intervention. An instructor manual with a collection of exercises is available, which enables individualised tailoring of the exercise programme to the performance level of the participants and still ensures comparability (see Appendix 1 and Appendix 2).

For gaze movement training, an exercise poster with a crosshair and different fixation points is used, which was developed for this clinical study in an interdisciplinary team (see Figure

2a). Using this exercise poster in combination with different exercises for the lower extremity and the trunk, a standardised, individually adapted, progressively increased training programme is then to be carried out over the intervention period. In addition, wall markers are used for eye movement intervention combined with activity-based balance training (Figure 2b). The exercises will be trained in different starting positions (sitting, wide stance, tandem stance, etc.) in order to train different movements in everyday situations. The study participants are accompanied in a 1:1 setting within the framework of individual physiotherapy four times a week. The physiotherapists in charge of each case are available to the participants for questions regarding the training throughout the entire period.

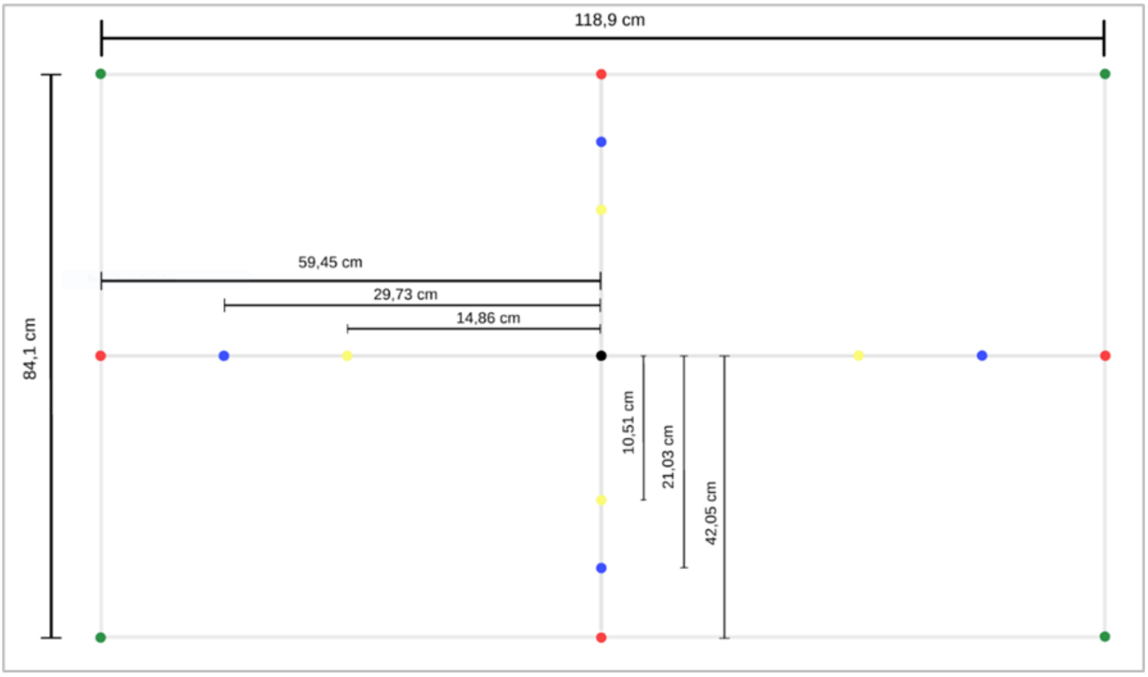

Figure 2a: Exercise poster

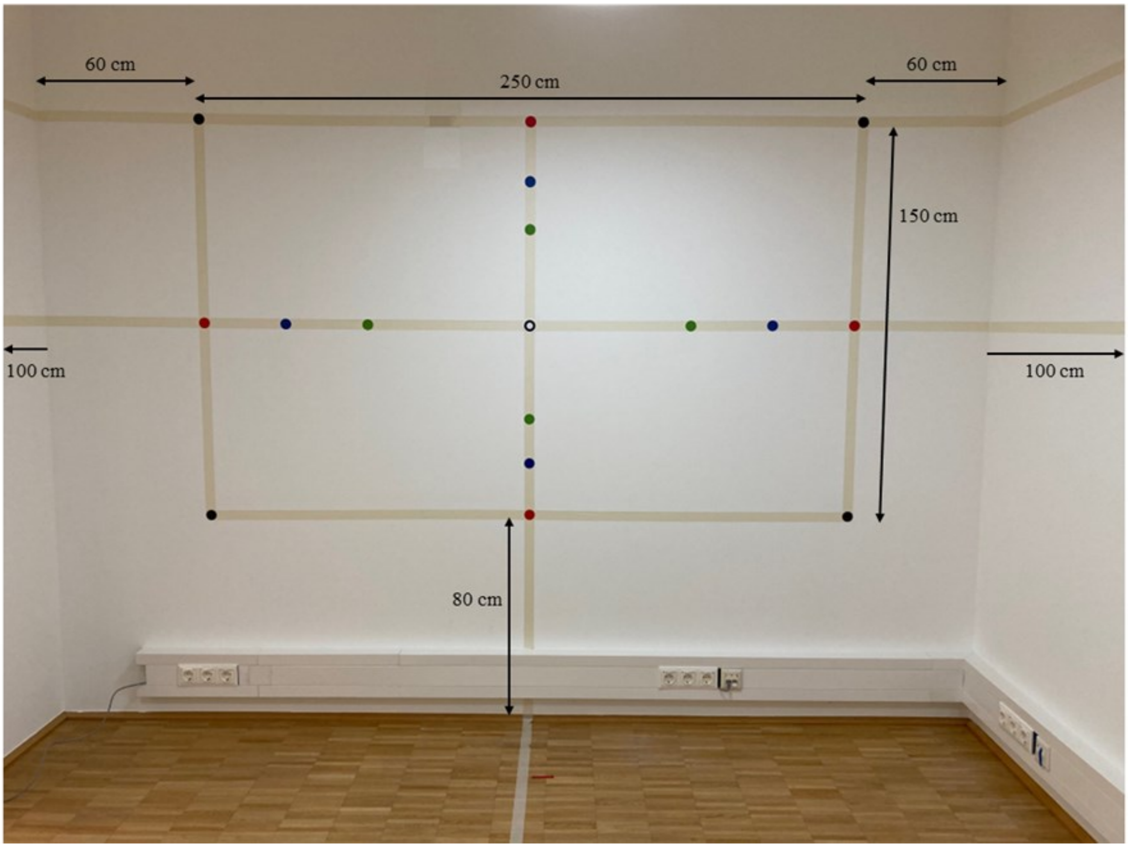

Figure 2b: Wall markings for the combined activity oriented physiotherapy and eye movement training intervention

The *activity-based physiotherapy training programme (group 2)* contains the same evidence-based, standardised physiotherapy exercises to improve coordination, strength and strength endurance, without explicit gaze movement training. As in the intervention group, the control group also carries out training supervised by the respective physiotherapists 4 times a week.

4.3 Endpoints and data collection

4.3.1 Demographic and disease-specific data

Demographic and disease-specific data will be collected, such as participants' age and gender, level of disability (UPDRS including H & Y) and date of initial diagnosis, dopaminergic medication and levodopa equivalent dose (mg).

4.3.2 Data collection and investigators

The data collection will be carried out by trained and blinded raters (doctors, physiotherapists) at baseline, post-intervention and at the follow-up call. After the end of the intervention, the participants' experience and acceptance of the intervention will be assessed in focus groups. A follow-up call will take place one month after the end of the intervention, i.e. two months after the baseline assessment. This survey will include a very short interview, the fall rate during the 4-week intervention period and two questionnaires (FOGQ, FES-I).

4.3.3 Primary and secondary endpoints and survey instruments

Table 2 lists the primary and secondary endpoints of the study with the respective survey instruments selected. All assessments are used in their validated German version. The implementation and psychometric criteria of the clinical assessments are described in chapter 4.4.

Table 2: Primary and secondary endpoints and survey instruments

Endpoint	Survey	Scr	BL	Int.	PI	FU Call
Primary clinical endpoints						
Dynamic balance	Functional Gait Assessment (FGA)		X		X	
Dynamic balance with eye movements (saccade speed, amplitude, latency, fixation duration)	Items 5, 6 and 10 of the Functional Gait Assessment (FGA) with mobile eye tracking using Tobii Pro Glasses 3		X		X	
Secondary clinical endpoints						
Cognitive function	Mini-Mental State Test (MMST)	X				
Functional mobility	Timed-Up-and-Go (TUG)		X		X	
Functional mobility with manual dual task	Timed-Up-and-Go with manual dual task (TUGman)		X		X	
Static and dynamic equilibrium	Berg Balance Scale (BBS)		X		X	
Dynamic balance	Four Square Step Test (FSST)		X		X	

Walking speed	10-Metre Walk Test (10MWT)		X		X	
Health-related quality of life	Parkinson's Disease Questionnaire-39 (PDQ-39)		X		X	
Freezing of Gait	Freezing of Gait Questionnaire (FOGQ)		X		X	X
Fall-associated self-efficacy	Falls Efficacy Scale (FES-I)		X		X	X
Depression	Beck Depression Inventory-II (BDI-II)		X		X	
Fall rate	Number of falls (a) in the 3-month period before study entry (BL), (b) intervention period (PI) and (c) 4-week follow-up.		X		X	X
Feasibility of a larger randomised controlled trial						
	Recruitment rate		X			
	Retention rate				X	X
	Adherence rate			X		
	Adverse events			X	X	
	Acceptance of the intervention (continuous: narrative; focus groups in intervention group 1)			X	X	
Eye movements during the intervention in group 1: standardised exercises, each at the end of week 1-4, during exercise 2, 3, 4, 6 and 7.	Measured for 12 min: saccade velocity, amplitude, latency, fixation time (Tobii Pro Glasses 3)			X		
	Abbreviations: Scr=Screening; BL=Baseline; Int.=Intervention period; PI=Post-intervention-; FU=Follow-up- testing.					

4.3.3.1 Mobile eye-tracking glasses (Tobii Pro Glasses 3)

4.3.3.1.1 Use and application of the mobile eye-tracking glasses

The use and application of the Tobii Pro Glasses 3 eye-tracking glasses is as follows:

Eye-tracking glasses from Tobii AB are used to record the eye movements. The Tobii Pro Glasses 3 carry the CE marking and comply with the applicable health and safety regulations of the European Union. The Tobii Pro Glasses 3 and their handling comply with the following standards:

- EMC Emission: EN55022:2010 Class B, FCC part 15, Class B
- EMC Immunity: EN55024:2010
- SAR EN62311:2008
- Low Voltage Directive 2006/95/EEG
- EMC Directive 2004/108/EC
- Radio and Telecommunications Terminal Equipment Directive (R&TTE) 1999/5/EC
- RoHS Directive 2002/95/EC
- RoHS2 Directive 2006/121/EC
- WEEE Directive 2002/121/EC
- Reach Directive 2006/121/EC
- IEC/EN/UL60950-1:2005 (Electrical safety for global/EU/US)

(ETL Compliance: Conforms to ANSI/UL Std. 60950-1 Certified to CSA Std. C22.2 No. 60950-1; Canadian Compliance Statement: CAN ICES-3(B)/NMB-3(B))
The Tobii Pro Glasses 3 will be worn by the participants for the assessment (items 5, 6 & 10 of the FGA) at all assessment times and during the intervention at the last therapy session from week 1-4 for about 12 minutes each. Before the start of the tests, the participants are informed by the responsible examiners or therapists about the use of the eye-tracking glasses and about possible side effects such as temporary dizziness. The participants will be secured by the physiotherapists at all times. Should this be the case at any time, the examiner or therapist in charge will immediately switch off the glasses and the participants will remove them.

4.3.3.1.2 Technical data of the Tobii Pro Glasses 3

The Tobii Pro Glasses 3 have 16 illuminators and four eye cameras (two for each eye) integrated into the scratch-resistant lenses for optimal positioning and an unrestricted field of vision. The sampling rate is 50 or 100 Hz. The one-point method is used for calibration. The scene camera, video resolution is 1920 × 1080 at 25 fps. The total weight of the glasses (incl. cable) is 76.5 grams. The scene camera has a wide field of view (106° horizontal: 95°, vertical: 63°). With the help of the built-in accelerometer, gyroscope and magnetometer sensors, it is possible to obtain separate information on head and eye movements and to better filter the influence of head movements on the eye-tracking data. In addition, the Tobii Pro Glasses 3 have a wide range of nose pads, as well as corrective lenses for people with low vision, to ensure an optimal fit and wearing comfort.

4.3.3.1.3 *Safety precautions for the eye-tracking examination*

The eye-tracking glasses emit pulsed infrared (IR) light via the 16 illuminators to support the eye-tracking sensors. Certain medical and other devices are susceptible to interference from IR light. Therefore, before using the Tobii Pro Glasses 3, it is important to ensure that subjects do not need to use such sensitive devices, as their accuracy or proper functioning may be affected.

The examination with the Tobii Pro Glasses 3 lasts 12 min during the intervention 1x/week and about 5 min during the baseline and post-intervention assessments.

4.4 Implementation and psychometric criteria of clinical assessments

4.4.1 Functional Gait Assessment

The Functional Gait Assessment (FGA) (Wrisley et al., 2004) is used to assess dynamic balance during walking and evaluates a person's ability to perform multiple motor tasks while walking.

4.4.1.1 Implementation

The FGA consists of 10 items. The items address walking on a surface without a slope at different speeds, with head turns, with quick turns and stops, climbing stairs and climbing over obstacles. Individual items are scored on a scale of 0-3 points, with 3 points representing the best possible performance of the item. The total score is 30 points. The more points achieved, the better the dynamic balance of the test persons. The duration of the test is 5-20 minutes (Thieme et al., 2009).

4.4.1.2 Psychometric criteria

The MDC is 4 points (Petersen et al., 2017). For individuals with Idiopathic Parkinson's Syndrome, the cut-off value is ≤18/30 points for identifying an increased risk of falls. The sensitivity is 80.6% and the specificity 80.0%. (Yang et al., 2014). The inter-rater reliability (ICC=0.99; 95% CI = 0.99-1.00) and intra-rater reliability (ICC=0.99; 95% CI = 0.99-1.00) are both excellent (Yang et al., 2016). Test-retest reliability is also very good with an ICC value of 0.86 (Petersen et al., 2017). Cronbach's alpha is 0.94, indicating excellent construct validity. (Yang et al., 2016).

4.4.2 Measurement of eye movements during items 5, 6 and 10 of the FGA

Items 5, 6 and 10 of the FGA are used separately to record dynamic balance and eye movements (saccade speed, amplitude, latency, fixation duration). This measurement is done with Tobii Pro Glasses 3 as explained above.

4.4.3 Mini Mental Status Test (MMST)

The MMST (Folstein et al., 1975) is used to assess cognitive function (temporal and spatial orientation, memory, attention, speech and language comprehension, also reading, writing, drawing and arithmetic) and serves as a screening tool for this clinical study.

4.4.3.1 Implementation

The test consists of tasks and questions to assess the test person's cognitive function. A maximum of 30 points can be achieved, whereby a result ≤20 points can be assumed to indicate dementia. The cut-off point of 24 points reliably indicates dementia with high sensitivity and specificity. (Stuss et al., 1996). The test takes about 7 to 10 minutes to complete. (Folstein et al., 1975).

4.4.3.2 Psychometric criteria

The smallest measurable difference (MDC) for people with PD with mild neurocognitive impairment is 6.43 points, with the smallest measurable difference (MDC) for people with PD with severe cognitive impairment is 6.16 points (Lucza et al., 2015). The construct validity of the MMST is high, shown by significant correlations with the Dementia Rating Scale is 0.87 ($p<0.001$) (Aarsland et al., 2004). Studies have shown that the MMST can validly measure cognitive decline over a longer period of about 10 years. (Lessig et al., 2012).

4.4.4 Timed-Up-and-Go (TUG)

The Timed-Up-and-Go is used to assess functional mobility, which is a primary endpoint of this study. (Podsiadlo and Richardson, 1991). The TUG is used as an indicator of the risk of falling during everyday movement tasks. The performance of the activities transfer from sitting to standing, walking, as well as making a turn in space, going back to the chair and sitting down is tested. For locomotion in everyday life and for the reduction of the risk of falling, the abilities to transfer from sitting to standing and to make a turn in space are of decisive importance. (Cheng et al., 1998; Mong et al., 2010)..

4.4.4.1 Implementation

The test starts in the seat, on a chair with a seat height of about 46 cm. To perform the TUG, the time it takes the test persons to stand up from the chair, walk a distance of three metres, turn around in the room, walk back the three metres to the chair and return to the starting position is recorded in seconds. The time required is documented in seconds. Aids that the test persons are currently using may be used. All aids used must be documented. The performance requires less than 5 minutes.

4.4.4.2 Psychometric criteria

The Standard Error of Measurement (SEM) is defined by Dal Bello-Haas and colleagues (2011) in people with idiopathic Parkinson's syndrome (Hoehn & Yahr stages 1-3) with 1.75 s. The smallest measurable difference (MDC) is 3.5 s. The smallest measurable difference (Minimal Detectable Change, MDC) is 3.5 s (Huang et al., 2011). The same authors report excellent test-retest reliability in people with idiopathic Parkinson's syndrome, with an ICC value of 0.80. The TUG has excellent test-retest reliability in people with idiopathic Parkinson's syndrome. The TUG has excellent inter-rater reliability and intra-rater reliability with ICC values of 0.99 and 0.98 respectively (Morris et al., 2001). In terms of criterion validity, very good values were achieved in persons with stroke in comparison with the Clinical Guideline System (CGS) (r = -0.86), the Fast Gait Speed (FGS) (r = - 0.91) and the 6 Meter Walk Test (r = 0.92). (Flansbjer et al., 2005).. For construct validity, Dal Bello-Haas described an excellent correlation with the Activities-specific Balance Confidence (ABC) Scale (r= -0-44, p= 0.03), and Bennie and colleagues (2003) an excellent correlation with the BBS (r= -0.47, p= 0.04). For predictive validity in individuals with Idiopathic Parkinson's Syndrome, Mak and Pang (2009) give Mak and Pang (2009) state that there is a significantly increased risk of falling with values >16 s.

4.4.5 Timed-Up-and-Go with manual dual task (TUGman)

The timed-up-and-go with manual dual-task to assess fall risk is a secondary endpoint of this study. (Shumway-Cook et al., 2000). The TUGman is used as an indicator of the risk of falling during daily movement tasks with dual-task.

4.4.5.1 Implementation

The test starts in a seated position, on a chair with a seat height of about 46 cm. To carry out the TUGman, the time it takes the test persons to stand up from a chair, grab a glass of water, walk a distance of three metres with the glass in their hands, turn around in the room with the

glass in their hands, walk the three metres back to the chair, put the glass down and return to the starting position is recorded in seconds. The time taken is recorded in seconds. Aids that the test persons are currently using may be used. All aids used must be documented. The test takes 5 minutes to complete.

4.4.5.2 Psychometric criteria

The TUGman has excellent inter-rater reliability (r= 0.98) and intra-rater reliability with an ICC value of 0.99. (Hofheinz and Schusterschitz, 2010). For criterion validity, Hofheinz and Schusterschitz (2010) described very good values in comparison with the BBS (r= -0.72). For predictive validity in persons with idiopathic Parkinson's syndrome, the following values are given Maranhão-Filho and colleagues (2011) state that a difference of > 4.5 s between TUG and TUGman indicates an increased risk of falling. Shumway-Cook and colleagues (2000) describe a prediction rate of 90% in the identification of older adults as fallers if they take more than 14.5 seconds to complete the TUGman.

4.4.6 Berg Balance Scale (BBS)

The BBS (Berg et al., 1989) is a common measurement tool in physiotherapy that can be used to check the balance ability of patients.

4.4.6.1 Implementation

The BBS includes 14 activities which the therapist observes and evaluates in terms of static and dynamic balance. The activities are based on actions of everyday life: Transfers from sitting to standing, standing to sitting and transferring (items 1, 4 & 5), static balance in standing and sitting without support, standing with eyes closed, standing with close foot position, reaching forward with both arms, picking up an object from the floor, looking over the shoulder while standing, turning once 360° while standing, tandem stand (One foot directly in front of the other) and single leg stand (items 2, 3, 6, 7, 8, 9, 10, 11, 13 &14) and dynamic balance in standing by placing both feet alternately on a step (item 12). (Scherfer et al., 2006).

The 14 items are rated with the points 0-4. The value 0 describes that the patients can only perform the tasks with maximum help. 4 points, on the other hand, mean that the patients can perform the task without any problems. A maximum score of 56 points is therefore to be achieved. Berg et al. (Berg et al., 1989) divide the patients into three groups after the evaluation. A score of 0-20 indicates that the patient needs a wheelchair, 21-40 points indicate assistance with walking and 41-56 points indicate independent walking. The time required to complete the test is 15-20 minutes.

4.4.6.2 Psychometric criteria

The MDC is 5 points (Steffen and Seney, 2008a). The cut-off value of the BBS for an increased risk of falling is ≤52/56 points. (Schlenstedt et al., 2016).. The test-retest reliability and the inter-rater reliability are in a very good range with ICC values of 0.80 and 0.95, respectively. (Leddy et al., 2011). Franchignoni and colleagues demonstrated a high internal consistency (Cronbach's alpha = 0.95) in 70 people with idiopathic Parkinson's syndrome and an average Berg Balance score of 46.5 (34-54) points. (Franchignoni et al., 2005).. For criterion validity, Brusse and colleagues describe Brusse and colleagues (2005) describe very good values in comparison with the TUG (r= -0.78) and the UPDRS (r= -0.64).

4.4.7 Four Step Square Test (FSST)

The Four Step Square Test (FSST) (Dite and Temple, 2002) addresses balance and the ability to step forward, sideways and backward over obstacles.

4.4.7.1 Implementation

The test persons stand in a square, which is formed by a wooden cross lying on the floor. The test persons are asked to step clockwise and then counterclockwise into all four squares. To do this, they must step forwards, sideways and backwards over the cross lying on the floor. There are always two runs, a trial run and a run in which the time is stopped. The execution time is less than 5 minutes.

4.4.8 10-metre walk test

4.4.8.1 Implementation

This test of walking speed is an important secondary outcome parameter. It is highly recommended by the Parkinson's Taskforce (PD EDGE) for patients with IPD in a H & Y stage 1-3. Crossing a road with or without traffic lights requires the ability to travel a short distance at the highest possible speed. If this ability is not sufficiently present, activities in the area of participation are significantly limited. In this test, lines are placed on the ground marking the start, 2m, 8m and 10m. The time between the 2m and 8m line is measured to exclude the acceleration and deceleration phases. The time taken to cover these 6m is written down to the hundredth of a second. The 6m is then divided by the time taken (in seconds). This gives the speed in m/s. Two runs are made in this way and the average is used. Aids that the patient is currently using may be used. Any aid used must be documented. When the examiner is giving the test instructions, it must be ensured that the examiner is not in front of or directly next to

the patient and that he/she does not interfere with the patient's performance of the test. The examiner should be at least half a metre behind the patient.

4.4.8.2 Psychometric criteria

The Standard Error of Measurement (SEM) is reported to be 0.04 m/s and the Minimal Clinically Important Difference (MCID) is reported to be 0.18 m/s at comfortable walking speed and 0.25 m/s at maximum speed. (Steffen and Seney, 2008). The test-retest reliability of the 10MWT is excellent in individuals with PD (ICC = 0.96- 0.97). (Steffen and Seney, 2008)as is the inter-rater reliability (ICC=0.93-0.99). (Lindholm et al., 2018). A low standard error of measurement of 0.032-0.076 m/s and high predictive validity were shown with an Area under the Receiver Operating Characteristic Curve (ROC) of 0.70-0.73 with respect to the risk of falling (Lindholm et al., 2018). Cut-off values for IPD patients were 1.1-1.2 m/s (Lindholm et al., 2018).

4.4.8.3 Psychometric criteria

Duncan and Earhart (2013) evaluated a cut-off value for a high risk of falling at >9.68 seconds for people with idiopathic Parkinson's syndrome. The inter-rater reliability is very high with an ICC value of 0.99. For criterion validity describe Duncan and Earhart (2013) show a strong correlation of the FSST compared with the UPDRS Scale III (r= 0.61) and a moderate correlation with the FOG-Q (r= 0.44).

4.4.9 Parkinson's Disease Questionnaire (PDQ-39)

The PDQ-39 (Berger et al., 1999; Peto et al., 1995) is used to assess health-related quality of life and is a secondary endpoint of this study. Written permission to use the German version of the PDQ-39 in this study was obtained from Mapi Research Trust.

4.4.9.1 Implementation and evaluation

The PDQ-39 is a 39-item questionnaire for self-evaluation of health-related quality of life (Patient Rated Outcome Measure, PROM). It consists of 8 dimensions: Mobility (items 1 to 10, max. 40 points), daily activities (items 11 to 16, max. 24 points), emotional well-being (items 17 to 22, max. 24 points), stigma (items 23 to 26, max. 16 points), social support (items 27 to 29, max. 12 points), cognition (items 30 to 33, max. 16 points), communication (items 34 to 36, max. 12 points), physical discomfort (items 37 to 39, max. 12 points). The evaluation is based on a 5-point Likert scale, from 0 = "Never" to 4 = "Always". Recoding of the response categories is not necessary for the creation of the subscales. All 8 domain scores and the Global Index are standardised on a 0-100 scale, with 0 representing the best health-related

QoL and 100 the worst. Mapi Trust provides an SPSS syntax for scoring. The index can only be calculated in each case if the percentage of missing data for all dimensions is ≥50%. The PDQ-39 takes 10-20 min to complete.

The evaluation is based on the evaluation instructions for the German version of the PDQ-39 (Berger et al., 1999).

Subscales

The 39 individual items of the PDQ-39 are added to the following 8 subscales: Mobility, Activities of Daily Living, Emotional Well-being, Stigma, Social Support, Cognition, Communication, Physical Discomfort. The assignment of the corresponding items to the 8 subscales are shown in table 3.

Table 3: PDQ-39 subscales and assigned items

**Mobility** (10 items)<br>Leisure activities<br>Household activities<br>Shopping<br>Walk 1 km<br>Walk 100 m<br>Move around the house Move around in public Escort needed Fear of falling<br>Tied to the house	**Stigma** (4 items)<br>Hide illness<br>Avoid situations<br>Shame<br>Worries about reactions<br><br>**Social support** (3 items)<br>Problems with people<br>Support spouse<br>Support friends
**Everyday activities** (6 items)<br>Washing problems<br>Tightening problems<br>Buttoning problems<br>Indistinct writing<br>Cut food into small pieces<br>Spill a drink<br><br>**Emotional well-being** (6 items) Feeling depressed<br>Loneliness Near tears<br>Being angry<br>Be anxious	**Cognition** (4 items)<br>Fall asleep during the day<br>Concentration problems<br>Poor memory<br>Bad dreams<br><br>**Communication** (3 items) Speech difficulties Communication problems<br>Lack of attention<br><br>**Physical discomfort** (3 items) Muscle cramps<br>Joint pain Feeling hot/cold

Worries about the future	

The 5 answer categories are coded as follows: 0 = never, 1 = rarely, 2 = sometimes, 3 = often, 4 = always or I cannot do it at all. Recoding of the answer categories is not necessary for the creation of the subscales.

For each subscale, a raw score is first calculated by adding the associated individual items for each patient. By transforming the raw scores to a scale between 0 (=best) and 100 (=worst), the individual scale scores become comparable. The following formula is used for the transformation:

$$\frac{\text{(raw scale value x 100)}}{\text{maximum scale value}}$$

(examples: Raw scale value of a patient for 'Mobility' = 30 ⇒ 30 x 100 / (4 x 10) = **75**

Raw scale value for 'Stigma' = 4 ⇒ 4 x 100 / (4 x 4)= **25**)

The values obtained by transformation correspond to a patient's PDQ scores on the corresponding subscale. In our example, a score of 75 on the 'Mobility' subscale means that the patient has achieved a score that is 75 per cent worse than the best possible score. Similarly, a score of 25 on the 'Stigma' scale means that this patient has achieved a score that is 25 per cent below the best achievable score.

Missings

The handling of missing answers (missings) requires special attention. Since they cannot be avoided in self-answering, the following procedure is recommended. If at least 50 per cent of the items of a subscale have been answered, the missing answers are replaced by the mean value of the existing answers in this scale. This means that in the 10 item scale a maximum of 5 questions may remain without an answer, in the 6 item scales a maximum of 3, in the 4 item scales a maximum of 2 and in the 3 item scales only one question. After replacing the missing answers with the mean values of the existing ones within a subscale, the sum scores of the eight subscales are calculated by addition and transformation as described above. When collecting the PDQ answers in the interview, no missings should occur.

PDQ - 39 Total Score (PDSI)

A PDQ-39 sum score (PDQ-39 SI) can be calculated from the 8 subscales. It represents the mean value of the 8 subscales, weighted by the number of items. For its calculation, the

PDQ values of each scale are added up for each patient and the sum divided by 8 (=number of scales).

[Mobility + Activities of daily living + Emotional well-being + ... + Physical Discomfort] / 8 = PDQ-39SI

When interpreting the PDQ-39 SI, its weighting by the number of corresponding questions per scale must be taken into account. As a composite score, it is generally less sensitive to change than the subscales of primary interest.

4.4.9.2 Psychometric criteria

All seven of the eight dimensions of the PDQ-39 showed satisfactory discriminant validity (Schrag, 2000). Internal consistency is excellent (Cronbach's alpha = 0.84-0.94). (Jenkinson et al., 1997).. The Standard Error of Measurement (SEM) is reported by XXX for the 8 dimensions as follows: Mobility (5.85-11.61), Activities of daily living (6.96-11.46), Emotional well-being (8.26-13.56), Stigma (5.57-13.67), Social support (7.84-17.61), Cognition (8.54-12.34), Communication (6.67-14.95), Physical discomfort (9.94-16.37). (Martinez-Martin et al., 2007). Excellent convergent validity was found with moderate to strong significant correlations of the PDQ-39 with Hoehn & Yahr stage (r= 0.60), UPDRS-ME (r= 0.41) and SF-36 (r= 0.34-0.80). (Schrag, 2000). Satisfactory internal and external validity as well as acceptable reliability were found for all eight dimensions of the PDQ-39 (Jenkinson et al., 1997).. According to Jenkinson and colleagues (1997), the PDQ-39 better represents the impact of QoL in people with Parkinson's disease than generic measures such as the Short Form-36 Health Survey (SF-36).

4.4.10 Freezing of Gait Questionnaire (FOG-Q)

The Freezing of Gait Questionnaire (Giladi et al., 2000) is a valid instrument for assessing FOG in people with idiopathic Parkinson's syndrome without dementia. The FOG-Q was validated for German-speaking countries in 2015. (Vogler et al., 2015). It is used to quantify the FOG. Due to its short implementation time and high practicability, the FOG-Q is very well suited for assessing FOG in clinical practice.

4.4.10.1 Implementation

The FOG-Q is a PROM for the evaluation of FOG. It contains 6 items, which are collected from the persons with Idiopathic Parkinson's Syndrome or, if necessary, as an interview from the responsible doctors or therapists. The evaluation is based on a 4-point Likert scale, from 0=

"normal" to 4= "unable to walk", with higher scores representing more pronounced freezing. The questions address whether FOG is present, whether daily life is affected by it and whether freezing occurs when walking or turning. The procedure requires 5-10 min.

4.4.10.2 Psychometric criteria

The construct validity of the German version of the FOG-Q was tested in 27 persons with Idiopathic Parkinson's Syndrome (Hoehn & Yahr II-IV) with FOG. The FOG-Q has good internal consistency (Cronbach's a: 0.83). A significant correlation was demonstrated between the Freezing item (2.13) and the MDS-UPDRS (p=0.002) and mobility or activities of daily living of the PDQ-39 (p=0.006 and p= 0.035, respectively). The results showed no association between the FOG-Q and the MDS-UPDRS subscale I (p= 0.079), indicating good divergent validity. (Vogler et al., 2015).

4.4.11 Falls Efficacy Scale- International Version (FES-I)

A large number of older people have psychological problems that are related to falls. This applies both to people who have already fallen and to people who have not yet fallen. In order to address these problems, it is necessary to assess fear of falling and self-efficacy in relation to falling. The Falls Efficacy Scale (Tinetti et al., 1990) was extended to an international version (FES-I) in 2005 by an expert network on fall prevention (Prevention of Falls Network Europe, ProFaNE). (Yardley et al., 2005; Skelton et al., 2004). The PROM is used to assess fall-associated self-efficacy in older people. With the extension, more complex, functional activities and social aspects of self-efficacy were included.

4.4.11.1 Implementation

The FES-I consists of 16 items that evaluate the concerns about falls and various activities in the subjects. The questionnaire can be administered as a self-report questionnaire or as a structured interview. The answers are ranked on a 4-point Likert scale from 1= "no concerns" to 4= "very great concerns". The duration of the questionnaire is 10 minutes.

4.4.11.2 Psychometric criteria

In the course of the further development of the FES into the FES-I by the expert network on fall prevention (Prevention of Falls Network Europe ProFaNE), a German translation was made. However, validation is only available for the English version. (Skelton et al., 2004). The results of the English version show a high retest reliability for the total score (r= 0.96) and a

high internal consistency (Cronbach's alpha= 0.96). Furthermore, the item intercorrelation was on average r= 0.55 (range r= 0.29-0.79). (Dias et al., 2006).

4.4.12 Beck Depression Inventory (BDI-II)

The BDI-II (Beck et al., 1988) is used to assess the severity of depression and is a secondary endpoint of this study. Written permission to use the German version of the BBD-II in this study was obtained from the Mapi Research Trust (fee required; https://mapi-trust.org/).

4.4.12.1 Implementation

The BDI-II consists of 21 items and is a PROM for assessing the severity of depression. The items are evaluated on a 4-point Likert scale from 0 to 3, which means that the sum of the items can be 0-63 points. A depression can be identified if the score is greater than 10. The scale is validated from the age of 13. The duration of the questionnaire is 5-10 minutes.

4.4.12.2 Psychometric criteria

The internal consistency is excellent (Cronbach's alpha = 0.89-0.93). The reliability for a sample of depressed test persons is 0.92. The reliability for healthy persons is 0.80 and 0.82. The retest reliability for healthy persons can be regarded as sufficiently stable, as it is 0.78 over three weeks and five months. The retest reliability is lower for test persons with a value of 0.46. The criterion validity in relation to the correlation with quality of life shows values between 0.50 and 0.75. (Kühner et al., 2007).

For subjects with idiopathic Parkinson's syndrome, a test-retest stability with an ICC value of 0.89 could be determined. (Visser et al., 2006). For persons with psychiatric disorders, the following cut-off values could be determined: Minimal depression is identified from a score of 10.9 (SD=8.1) points, mild depression from a score of 18.7 (SD=10.2) points, moderate depression from a score of 25.4 (SD=9.6) points, severe depression is present from a score of 30.00 (SD=10.4) points. (Beck et al., 1988).

4.4.13 Fall rate

There is a survey of the number of falls
(a) in the 3-month period before the start of the study (subjectively asked at baseline)
(b) in the 4-week intervention period (observed and subjectively asked about post-intervention)
(c) in the 4-week follow-up period (subjectively asked during the follow-up call)

The number of falls is documented in the CRF.

4.4.14 Feasibility of a larger study

4.4.14.1 Feasibility criteria

The feasibility criteria for a larger study are defined as follows:

a) a target recruitment rate of 40% of 85 eligible patients (or 4-5 participants per month); the total number of eligible PD patients was estimated based on the number of eligible PD patients treated at RZM in the last 3 years.

(b) a target retention rate of 80%,

(c) a minimum target adherence rate of 75% (at least 3 out of targeted 4 practice sessions per week; documented in the CRF).

(d) high safety of the intervention, no severe side effects or only very mild and transient side effects (continuously collected by means of UE log in CRF; see also chapter 4.5.2)

(e) high acceptance of the intervention, continuously evaluated narratively during the intervention period and through focus groups with semi-structured questions (see chapter 4.4.14 and CRF for a detailed description of the focus groups and the questions asked).

4.4.14.2 Assessment of safety and side effects

In order to assess the safety of the participants, the following subjective and objective safety parameters are collected. Subjective parameters are those reported by the participants or asked by the therapists. Examples are general well-being, signs of stress, etc. Constant consultation with the participants regarding these parameters is a standard procedure in the therapy of people with idiopathic Parkinson's syndrome and serves to individually adjust the intensity of the therapy. Objective parameters for assessing safety are those serious or non-serious adverse events that are recorded and evaluated during the study (see chapter 4.5).

Compatibility/ safety parameters

- Subjective by the participant:
  Through close consultation and feedback with the treating doctor and therapist
  (→ adapt the therapy as the situation requires or as is possible for the participant - usual procedure in every daily therapy routine)
- Objective parameters:
  The recording and evaluation of side effects serves to objectify safety (see chapter 4.5.2).

There is continuous monitoring and assessment of the safety of the intervention to detect adverse events. The detailed procedure is described in chapter 4.5.2.

4.4.14.3 Acceptance of the intervention: focus groups

A focus group is a form of group interview that focuses on the communication between the participants (Stalmeijer et al., 2014). Through this type of data collection, not only the thoughts and feelings of the participants are collected, but also the background against which they arose. One aim of the focus group is to record, understand and explain the meaning of the beliefs and culture that influenced the participants' feelings, behaviour and attitudes (Rabiee, 2004). Focus groups are particularly well suited to areas that have been little or poorly researched (Kitzinger, 1995). They can also be used to confirm or further support existing data (Stewart et al., 2007).

The questions posed for discussion in the focus groups are listed in the CRF.

4.4.15 Measurement of eye movements during the intervention

Once a week, at the last therapy session, the eye movements are measured for 12 minutes using Tobii Pro Glasses 3: saccade speed, amplitude, latency and fixation time. This measurement is done after a 5-minute warm-up (warm-up: see Instructor Manual, Appendix 1).

The 12-minute period was chosen because it is expected that the participants will have a familiarisation phase with the Tobii glasses of about 5-6 minutes (communication with Tobii). The data obtained are thus more reliable and closer to reality. Data collection will be done during the performance of standardised exercises to allow for comparison: Exercise 2, 3, 4, 6 and 7.

4.5 Adverse events, side effects

4.5.1 Definitions

The implementation of the tests and interventions (therapy of the intervention groups) can trigger adverse events and side effects. During the therapy, events such as dizziness, shortness of breath or similar signs of stress may occur as a physiological reaction to stress (the training) in both the intervention group and the control group. In the intervention group, slight eye fatigue may occur, but this can be well controlled by short training sequences, variable exercises as well as breaks and exercises without eye movement training.

4.5.1.1 Adverse event (AE)

An adverse event (AE) is any adverse occurrence that happens to a subject during participation in this study that is not necessarily causally related to the intervention. This may include illnesses, signs and symptoms that occur after the subject is included in this study or pre-existing illnesses, signs and symptoms that worsen after inclusion in this study.

Pre-existing conditions that do not worsen during the course of the study and adverse events related to concomitant medication are excluded.

4.5.1.2 Side effect (NW)

An adverse event (AE) is any adverse event, as defined above, for which there is causality to the study intervention.

4.5.1.3 Serious incident

A serious incident is defined as any adverse event or side effect that either

- Leads to death, or
- is life-threatening, or
- Leads to a permanent or serious disability or invalidity, or
- Requires inpatient treatment or its prolongation (unless the inpatient treatment was already planned before participation in this study).

Depending on the causality to the study intervention, a distinction is made between a serious adverse event (SUE) or a serious adverse reaction (SNW).

4.5.1.4 Severity

The severity levels of the incidents ((S)UE and (S)NW) are defined as follows:

- Mild - Intervention must be interrupted due to the incident, but can be continued the same day or the following day. Consultation with a doctor for treatment is not necessary;
- Moderate - Intervention must be interrupted, can be continued on another day but not immediately the following day. Consultation with a doctor for treatment is necessary.
- Severe - The intervention must be terminated and cannot be continued due to the event (in terms of a drop-out according to chapter 5.4)

4.5.1.5 Causality

Causality is defined as follows:

- Treatment-associated - Either the adverse event is known to be caused by the study intervention, or there is reasonable suspicion that the study intervention caused the adverse event, or there is a temporal relationship between the study intervention and the occurrence of the adverse event.
- Non-therapy associated - There is no reasonable suspicion that the adverse event was caused by the study intervention or there is no temporal relationship between the study intervention and the occurrence of the adverse event.

4.5.2 Assessment and documentation

Within the framework of this study, all adverse events and side effects are systematically recorded, assessed and evaluated at the end of the study.

4.5.2.1.1 Assessment process

An authorised doctor must evaluate and confirm the possible association with the intervention, the severity (mild, moderate, severe) and the classification of the incident as serious or non-serious.

4.5.2.1.2 Documentation of adverse events (UE/NW)

During the intervention phase (week 1 - week 4), the occurrence of the above-mentioned incidents will be recorded by the responsible staff at the study centre and assessed by a member of the medical team.

All incidents and findings must be documented in the patient record and subsequently in the UE log of the CRF.

The following information is required:

- Type of Adverse Event (sign, symptom or disease)
- Beginning and end of occurrence
- Severity
- Causality to intervention
- Differentiation (serious/not serious)
- Measures regarding the intervention or actions to restore or improve the patient's well-being.
- Outcome of the event.

4.5.2.1.3 Documentation of serious incidents (SUE/SNW)

Serious incidents must be recorded in the CRF Serious Incident Report Form in addition to the documentation in the UE Log.

The following points must be strictly adhered to when documenting the serious incidents:

- Each SUE/SNW must be reported as completely as possible.
- Findings must be attached to the report in pseudonymised form (using the patient's code).

The investigator of the respective trial site must check the serious incident report form for completeness and ensure that the information entered matches that in the CRF UE log and other data sources.

4.5.3 Reporting of serious incidents (SUE/SNW)

4.5.3.1 Reporting obligations of the examiner

All serious incidents occurring during the study must be reported immediately after becoming aware of them (≤ 24 hours/1 working day) by submitting the Serious Incident Form to the Coordinating Investigator and Sponsor (email: christian.brenneis@reha-muenster.at).

Please note: Personal data (e.g. findings) must be pseudonymised using the patient's code before they are transmitted (DSVO-compliant: in person, analogue fax).

4.5.3.2 Reporting obligations of the sponsor

The sponsor must inform the Ethics Committee of the Medical University of Innsbruck in writing about all serious incidents that occur during the course of this clinical trial immediately after they become known (≤ 24 hours/1 working day).

In the event of (unanticipated) occurrences that may lead to an increase in risk, the Ethics Committee must be referred again.

5 Data documentation , -management and -evaluation

5.1 Data collection methods

The data collection for the evaluation of the endpoints is exclusively collected with valid, reliable and responsive patient-oriented instruments, which are listed and described in chapter 4.4.

5.2 Documentation

5.2.1 Source data and documents

Source data is considered to be all information from original documents and reports and their certified copies that is necessary for the reconstruction and assessment of the study. Source data are contained in source documents.

Source documents in this study are:

e.g. medical records, consent forms, therapy documentation, etc.

5.2.2 Survey questionnaire (CRF)

All patient data and examination results will be entered into the CRFs (Case Report Forms) specially created for this study.

The survey forms may only be filled in **with a biro or fineliner (black or blue).** Corrections must be made in such a way that the old entry remains legible (the use of correcting agents is not permitted). Corrections must be signed and dated by the authorised person making them. Data that are not available or that have not been collected must be clearly identifiable as such (F or ND). The reasons for this should be documented, if applicable.

The investigator shall ensure that all patient data are entered into the CRFs promptly, legibly, completely, accurately and in accordance with the patient records.

The completed original pages are sent to the responsible persons for data entry and evaluation of the data.

5.3 Further treatment of patients after completion of the examination

The patients are subject to the regular care scheme within the framework of their treatment of idiopathic Parkinson's syndrome, according to which further care is also provided after completion of the study.

5.4 Drop-out of patients (drop-out)

Patients may withdraw from the clinical trial at any time at their own request, early and without giving reasons and without consequences for their future treatment. In addition, patients can be excluded from the study by the investigator for reasons of health risk.

The following points or events also lead to the drop-out of the subject after initial inclusion in the study (in the sense of a classification as drop-out for the biometric analysis):

- No questionnaire scores or assessment scores of interpretable quality.
- Withdrawal of consent to study participation by the study participant within their study integration.
- Any change or adjustment in dopaminergic medication that the investigator decides is no longer compatible with the study objectives - see exclusion criteria.

If, after the start of the study intervention, the patient's health is endangered due to an unexpected deterioration in health or general condition and/or an absolute contraindication to physical training, participation in the study will be terminated immediately and a specialist consulted.

The reason for the patient's withdrawal from the study is documented in the CRF. The study must be continued with the next subject number.
All patients who leave the study prematurely are asked to undergo a final examination in the form of post-intervention testing at the next possible opportunity - if this is possible during the rehabilitation period and if they are in good health - and the results of this examination will be documented in the CRF.

5.5 Premature termination of the clinical trial

The sponsor is entitled to terminate the study prematurely due to relevant medical/administrative reasons. The reasons for discontinuation of the trial will be documented in detail. Patients who are still undergoing treatment at the time of discontinuation of the trial are requested to undergo a final examination in the sense of post-intervention testing at the earliest possible time, which will be documented in the CRF. If an investigator has ethical concerns about the continuation of the trial, this must be reported to the sponsor immediately.

The sponsor is entitled to terminate the clinical trial prematurely if

- the participant recruitment rate is inadequate,
- serious, unresolvable problems occur with the quality of the data collected,
- unforeseeable circumstances have occurred at the respective trial centre that do not allow the continuation of the clinical trial,
- unacceptable risks have occurred (decision after new risk-benefit assessment),
- new scientific findings during the term of the study do not allow the continuation of the study,

The study management can decide on the discontinuation of the study in consultation with the sponsor.

If an investigator has ethical concerns about continuing the study, this must be reported immediately to the sponsor.

5.6 Data analysis

5.6.1 Statistical data analysis

Statistical data analysis is performed using IBM SPSS software, version 26.0 (IBM Corporation, Armonk, NY, USA) and Tobii Pro Lab Analyer (Tobii, Danderyd, Sweden). The statistical significance level is defined by a p-value of <0.05. An attempt is made to avoid missing data by inspecting the questionnaires after completion and, in case of unanswered items, asking the study participants to complete them. The number of missing data is noted; due to the less sensitive topic of the study, it is assumed that missing variable values can be assigned to "missing completely at random" or "missing at random". An intention-to-treat analysis is performed for all cases analysed in their originally assigned group.

Descriptive statistics are used for demographic data and primary and secondary outcomes. Counted and nominal scaled data (gender, fall rate, recruitment, retention and adherence rate, number of missing data if applicable, number of adverse events) are reported as whole numbers or percentages. Ordinal scaled variables (MMSE, UPDRS, H & Y, FGA, BBS, PDQ-39, FOGQ, FES-I, BDI-II) are expressed as median (minimum, maximum or interquartile range) and metric variables (TUG, TUGman, FSST, 10MWT, eye movements: Saccade velocity, amplitude, latency, fixation duration) by mean (95% confidence interval, CI or standard deviation, SD). The data is also presented graphically (e.g. by bar charts, box plot or line graphs).

The eligibility rate (%) is the percentage of patients suitable for the study after applying the inclusion and exclusion criteria from the Parkinson's patients treated at the Reha Zentrum Münster during the study period.

The recruitment rate (%) is determined as follows: $(N_{Consent}/N_{eligible})*100$; where $N_{consent}$ is the number of participants who signed the informed consent form; $N_{eligible}$ is the number of patients eligible for the study based on the inclusion and exclusion criteria.

The retention rate (%) is calculated as follows: $(N_{completed}/_{Total})*100$; where $N_{completed}$ is the number of participants who completed the study; N_{total} is the total number of participants in the study.

Adherence rate (%) is determined as follows: (Actual number of exercise sessions/planned number of training sessions)*100 (Osterberg & Blaschke, 2005).

Eligibility, recruitment and adherence rates are calculated using the Wilson 'score' method propagated by Newcombe together with its 95% CI (Newcombe, 1998)In the case of a proportion close to 0 or 1, a Poisson approximation according to Brown is used. (Brown, Cai, & DasGupta, 2001)..

A test for statistically significant differences between the groups at baseline: for nominal data the Fisher's Exact Test is used, for ordinal data the Mann Whitney-U test and for metric data the T-test for independent samples. A preliminary inferential statistical analysis to detect trends in the effectiveness of the intervention and as a basis for sample size calculation for a randomised controlled trial with sufficient power of at least 80% (based on mean differences (SD) between groups) will be performed. For inferential statistics, metric data are first tested for normal distribution and significant data outliers using the Shapiro-Wilk test, Q-Q plots and histograms. Non-normally distributed data are transformed using suitable transformation (e.g. ln, square root), checked again and analysed non-parametrically if normal distribution is missing.

Differences between the two groups and test times are calculated for ordinal data as follows: New variables are generated from the difference between the post-intervention and baseline data. The difference between group 1 and 2 is determined using the Mann Whitney-U test. For the ordinal variables FES-I and FOGQ, which are also collected at the follow-up call, a Kruskal Wallis test is performed across all groups and test time points. Changes in fall rate (nominal) between baseline, post-intervention and follow-up will be calculated using Chi-Square test. Metric data will be tested for homogeneity of variance (Levene test) as the basic assumption of a 2 x 2 ANOVA, with "time (baseline, post-intervention)" as the within-factor and "group (1, 2)" as the between-factor. If there is no homogeneity of variance, an alternative F-statistic (e.g. according to Welch or Brown-Forsythe) is used. If the basic assumption of sphericity is not met, corrected values are used, e.g. according to Greenhouse-Geisser or Huynh-Feldt. To calculate the differences between the groups and measurement times, a 2 x 2 ANOVA (analysis of variance) is performed, followed by pairwise comparisons (Bonferroni correction for all comparisons) between the groups at measurement times 1 and 2 if statistical significance is present. In the case of failure of the adaptive measures, non-parametric tests are used as described for ordinal scaled data.

The effect size is given by partial Eta squared and standardised effect size (Cohen's d).

5.6.2 Qualitative data analysis

The interview data regarding the acceptance of the intervention will be analysed with a Qualitative Content Analysis (Berelson, 1952; Lasswell, 1948; Mayring, 2003).. The advantage of this method is the possibility of an initially qualitative and subsequently quantitative data analysis. includes the following work steps. (Bryman, 2012, 2007; Denzin et al. 2018; Schreier, 2012):

- Verbatim transcription of the semi-structured interviews (f4 transcript software)
- Develop a list of themes and objective categories (independent, complete, mutually exclusive and adequate to answer the research questions).
- Development of a coding framework
- Marking and segmentation of the text sections based on thematic criteria
- Marking the content to identify main categories
- Progressive summation of the data by grouping the codes into subcategories, classification and comparison
- Double coding of the data set after 10-14 days (reliability testing)
- Illustration of the content of the main and sub-categories through quotations
- Creating a data matrix, also quantitative analysis of the data (frequencies, descriptive statistics)
- Creation of the report

5.7 Auditor and data management

The documents required for the clinical trial shall be kept in the trial folder. The sponsor and investigator shall take appropriate measures to ensure the careful and confidential handling of all data generated in the course of a clinical trial.

5.7.1 Retention of study documents, data storage & deletion

Records and documents related to the trial (e.g. consent forms, data collection forms, and other relevant documents) shall be retained by the investigators for at least 15 years after the end or termination of the clinical trial. The investigator as well as the coordinating investigator shall ensure that the documents concerning pseudonymisation are retained for a period of 15 years after the end or termination of the clinical trial. The trial-related data, records and documents shall be stored in secure premises and on secure servers. The medical records and other original data shall be retained for the longest possible period permitted by the

institution. Access to the documents must be restricted to the study team members. After the 15-year period, the documents will be destroyed.

5.7.2 Data management

5.7.2.1 Data entry

For the statistical analysis, the data from questionnaires and survey protocols are compiled and entered by an authorised and trained person into the data collection form (CRF) for each measurement point (baseline, post-intervention testing, focus groups (see Qualitative Data Analysis), follow-up calls). The CRF only collects data that are specified in the protocol and that are needed to interpret the study results. Subsequently, the data are entered into the Excel database by two study staff members independently of each other, from where they can be imported into the statistics programme.

5.7.2.2 Quality assurance measures

The following measures are carried out to ensure the plausibility and integrity of the data:

- Documentation of protocol deviations and their evaluation as to whether there is an influence on the data quality
- Data verification - Random value range and field type checks as well as logical checks are performed on certain data fields. In the case of value range checks, the fields are checked for permitted values or permitted number of response options (in the case of multiple responses). Field type controls check whether the values entered match the definition of the field (e.g. numeric fields). Logical controls check time sequences (e.g. the order of visits/therapy units) or therapy and protocol compliance (e.g. by checking the free text fields).
- Double data entry - The data of the CRFs are transferred to the database by two independent verifiers. Subsequently, a data reconciliation takes place to detect possible transmission errors during manual entry as well as missing data. The discrepancies identified are each eliminated by a third authorised person until there are no more differences.
- Data check by the PI - prior to data analysis regarding the primary endpoint and the secondary endpoints, a recheck for plausibility takes place.

The review of the data focuses on the data on target criteria, patient safety and protocol deviations.

5.7.3 Dealing with queries

The data is checked for completeness and medical plausibility by means of the above-mentioned checks. If necessary, queries may arise, which are forwarded to the examiners in a structured form. Using the questionnaires, the examiner must check and answer the discrepancies that have arisen. These questionnaires are then forwarded back to the data management team, where these discrepancies are corrected accordingly in the database. The questionnaires are kept together with the survey forms at the test centre and with the data management team.

At the end of the study, the database is closed after all entries have been entered and queries have been clarified. This process is documented.

6 Ethical, legal and administrative aspects

6.1 Compliance with ethical and regulatory requirements

The study was planned in accordance with the requirements of the Tyrolean Hospitals Act (Tir KAG), the Declaration of Helsinki and the ICH-E6 guidelines, the OeAWI guidelines for good scientific practice, as well as the requirements of the General Data Protection Regulation (DSGVO) and the Austrian Data Protection Act (DSG).

The protocol was written according to the SPIRIT 2013 guidelines (Standard Protocol Items: Recommendations for Interventional Trials) and the TIDieR 2014 guidelines (Template for Intervention Description and Replication).

6.2 Vote of the Ethics Committee (ethical approval)

Prior to the start of the study, approval of the study plan, the informed consent form, amendments to these documents and other relevant study documents will be obtained from the Ethics Committee (EC) of the Medical University of Innsbruck.

The EC must be informed of any subsequent changes to the above documents.

6.3 Patient information and consent form

Prior to the start of the trial, all patients must declare their consent in writing to the investigators after they have been fully informed in verbal and written form about the nature, significance and implications of the clinical trial in a manner that is comprehensible to them. The content of this information shall be documented on the informed consent form. Patients will be informed if significant new findings about the tested intervention arise during the trial.

The patient's declaration of consent to participate in the clinical trial is dated and signed by the patient and the doctor. The patient is given one copy of the signed information letter and consent form for participation in a clinical trial for patients. The second copy is filed by the doctor in the trial folder.

Participants will also be provided with a consent form for audio recording during the focus groups. If several participants refuse, one or more focus groups will be conducted without audio recordings, with field notes only.

It is expressly pointed out that no examinations of any kind may be carried out in connection with the study until a legally valid declaration of consent has been obtained from the patient.

6.4 Patient insurance

On behalf of the sponsor, personal injury insurance has been taken out for all patients. This insurance covers all possible injuries suffered by the patients directly or indirectly as a result of the study intervention or interventions in connection with the clinical study.

In order not to jeopardise insurance coverage, patients must strictly follow the instructions of the investigators. Furthermore, patients must not undergo any other medical treatment during the clinical trial without the consent of the investigator (except in emergencies). Patients must inform the investigators immediately of any emergency treatment. Patients must immediately notify the investigators and the insurance company of any damage to health that may have occurred as a result of the clinical study. In addition, patients must take all appropriate measures to clarify the cause and extent of the damage that has occurred.

Patients can consult the insurance conditions with the examiners and receive a copy if they wish.

6.5 Data protection and confidentiality

The collection, transfer, storage and analysis of personal data within this clinical trial is carried out in accordance with the legal provisions of the General Data Protection Regulation (EU) 20216/679 (DSGVO) and the Austrian Data Protection Act (DSG). A prerequisite for this is the voluntary consent of the patients within the framework of the declaration of consent prior to participation in the clinical trial. Patients will be informed about the following during the information about this clinical trial:

1. data collected in the course of this clinical trial will be recorded on paper or electronic data carriers, will be treated as strictly confidential and will only be passed on without naming the subjects (pseudonymised) to

die (coordinating) investigators of the study for the assessment of adverse events,

den Sponsor of the study in case of serious adverse events,

die Biometrician of the study for scientific evaluation,

die responsible ethics committee of the Medical University of Innsbruck to review the proper conduct of the study and to evaluate study results and adverse events.

2. documents containing personal data (e.g. the signed information letter and consent form for participation in a clinical trial for patients, identification list for patients) remain at the study centre and are subject to the investigator's duty of confidentiality.

Insofar as this is necessary for the review of the clinical trial, authorised representatives of the competent supervisory authority who are bound to secrecy may inspect the personal data held by the investigators. For this measure, the investigators are released from their medical confidentiality obligation.

The consent to the collection and processing of personal data in the context of this clinical trial is irrevocable. Patients are informed that they can terminate their participation in the clinical trial at any time - without giving reasons and without any subsequent disadvantages. In the event of withdrawal of the declaration of consent, the data stored up to this point will continue to be used without naming the patient, insofar as this is necessary to determine the effects of the study intervention and to ensure that the data subject's interests worthy of protection are not impaired.

7 Changes during the course of the study

7.1 Amendments to the protocol

In order to ensure comparable conditions as well as in the interest of a proper data evaluation, a change of the agreed test conditions laid down in the test plan is not foreseen.

In exceptional cases, however, changes to the examination conditions are possible. These will only be made after mutual agreement between the Coordinating Investigator and the sponsor. Any change to the study procedure specified in the protocol must be made in writing, stating the respective reasons, and must be signed by all persons responsible for the study. The changes are then considered part of the protocol. If necessary (e.g. in the case of significant changes that have a direct impact on the safety of the participants in the study), the approval of the responsible ethics committee and/or authorities as well as of the patients must be obtained for the protocol changes.

The investigator has the possibility to order a general study stop in urgent situations between the request for a protocol amendment and its rejection or entry into force. This is mandatory if there is a trial protocol amendment or a change in the state of knowledge about the study-specific investigations with a potential re-evaluation of the benefit-risk calculation for the trial subject. In this case, the study will only be continued after a new vote by the ethics committee.

7.2 Deviations from the test plan

Deviations from the protocol should be avoided as far as possible. If deviations do occur, they should be documented immediately and reported to the sponsor and the funding agency. The deviation must be assessed by the sponsor in terms of its impact on patient safety and data quality and, if necessary, appropriate action taken. A list of protocol deviations is provided to the person performing the data analysis along with the data export.

7.3 Informing the study participants about the results of the study

After completion of the study, the final data analysis, the writing of the initial and final study report, the participants of the study will be informed by post about the results of the study. This information contains only anonymised data.

8 Funding, conflicts of interest and compensation of participants

8.1 Funding of the study

The planned study is an academic self-study without external funding.

8.2 Possible conflicts of interest

The investigators' conflicts of interest are enclosed in a separate form. This is a prospective randomised controlled pilot study with physiotherapy intervention and blinded data collection. None of the investigators receives a fee for their work.

8.3 Fees and compensation for participants

The participants of the study do not receive any fee or compensation for their participation in the study.

9 References

Alcock, L., Galna, B., Hausdorff, J.M., Lord, S., Rochester, L., 2020. Enhanced Obstacle

Contrast to Promote Visual Scanning in Fallers with Parkinson's Disease: Role of Executive Function. Neuroscience 436, 82-92. https://doi.org/10.1016/j.neuroscience.2020.03.032

Ambati, V.N.P., Saucedo, F., Murray, N.G., Powell, D.W., Reed-Jones, R.J., 2016. Constraining eye movement in individuals with Parkinson's disease during walking turns. Exp. Brain Res. 234, 2957-2965. https://doi.org/10.1007/s00221-016-4698-1

Andlin-Sobocki, P., Jonsson, B., Wittchen, H.-U., Olesen, J., 2005. Cost of disorders of the brain in Europe. Eur. J. Neurol. 12, 1-27. https://doi.org/10.1111/j.1468-1331.2005.01202.x

Baker, T., Pitman, J., MacLellan, M.J., Reed-Jones, R.J., 2020. Visual Cues Promote Head First Strategies During Walking Turns in Individuals With Parkinson's Disease. Front. Sports Act. Living 2, 22. https://doi.org/10.3389/fspor.2020.00022

Barbieri, F.A., Polastri, P.F., Gobbi, L.T.B., Simieli, L., Pereira, V.I.A., Baptista, A.M., Moretto, G.F., Fiorelli, C.M., Imaizumi, L.F.I., Rodrigues, S.T., 2018. Obstacle circumvention and eye coordination during walking to least and most affected side in people with Parkinson's disease. Behav. Brain Res. 346, 105-114. https://doi.org/10.1016/j.bbr.2017.11.032

Beck, A.T., Steer, R.A., Carbin, M.G., 1988. Psychometric properties of the Beck Depression Inventory: Twenty-five years of evaluation. Clin. Psychol. Rev. 8, 77-100. https://doi.org/10.1016/0272-7358(88)90050-5.

Bennie, S., Bruner, K., Dizon, A., Fritz, H., Goodman, B., Peterson, S., 2003. Measurements of Balance: Comparison of the Timed "Up and Go" Test and Functional Reach Test with the Berg Balance Scale. J. Phys. Ther. Sci. 15, 93-97. https://doi.org/10.1589/jpts.15.93

Berg, K., Wood-Dauphine, S., Williams, J.I., Gayton, D., 1989. Measuring balance in the elderly: preliminary development of an instrument. Physiother. Can. 41, 304-311. https://doi.org/10.3138/ptc.41.6.304

Berger, K., Broll, S., Winkelmann, J., Heberlein, I., Müller, T., Ries, V., 1999. Investigation of the reliability of the German version of the PDQ-39: A disease-specific questionnaire to assess the quality of life of Parkinson's disease patients. Current Neurol. 26, 180-184. https://doi.org/10.1055/s-2007-1017628

Brusse, K.J., Zimdars, S., Zalewski, K.R., Steffen, T.M., 2005. Testing functional performance in people with Parkinson disease. Phys. Ther. 85, 134-141.

Camacho, P.B., Carbonari, R., Shen, S., Zadikoff, C., Kramer, A.F., López-Ortiz, C., 2019a. Voluntary Saccade Training Protocol in Persons With Parkinson's Disease and Healthy Adults. Front. Aging Neurosci. 11, 77. https://doi.org/10.3389/fnagi.2019.00077

Camacho, P.B., Carbonari, R., Shen, S., Zadikoff, C., Kramer, A.F., López-Ortiz, C., 2019b. Voluntary Saccade Training Protocol in Persons With Parkinson's Disease and Healthy Adults. Front. Aging Neurosci. 11, 77. https://doi.org/10.3389/fnagi.2019.00077

Carpinella, I., Cattaneo, D., Bonora, G., Bowman, T., Martina, L., Montesano, A., Ferrarin, M., 2017. Wearable Sensor-Based Biofeedback Training for Balance and Gait in Parkinson's Disease: A Pilot Randomized Controlled Trial. Arch. Phys. Med. Rehabil. 98, 622-630.e3. https://doi.org/10.1016/j.apmr.2016.11.003

Cheng, P.-T., Liaw, M.-Y., Wong, M.-K., Tang, F.-T., Lee, M.-Y., Lin, P.-S., 1998. The sit-to-stand movement in stroke patients and its correlation with falling. Arch. Phys. Med. Rehabil.

79, 1043-1046. https://doi.org/10.1016/S0003-9993(98)90168-X

Craig, P., Dieppe, P., Macintyre, S., Michie, s., Nazareth, I., Petticrew, M., 2006. Developing and evaluating complex interventions: new guidance.

Cucca, A., Acosta, I., Berberian, M., Lemen, A.C., Rizzo, J.R., Ghilardi, M.F., Quartarone, A., Feigin, A.S., Di Rocco, A., Biagioni, M.C., 2018. Visuospatial exploration and art therapy intervention in patients with Parkinson's disease: an exploratory therapeutic protocol. Complement. Ther. Med. 40, 70-76. https://doi.org/10.1016/j.ctim.2018.07.011

Dal Bello-Haas, V., Klassen, L., Sheppard, M.S., Metcalfe, A., 2011. Psychometric Properties of Activity, Self-Efficacy, and Quality-of-Life Measures in Individuals with Parkinson Disease. Physiother. Can. 63, 47-57. https://doi.org/10.3138/ptc.2009-08

Dias, N., Kempen, G.I.J.M., Todd, C.J., Beyer, N., Freiberger, E., Piot-Ziegler, C., Yardley, L., Hauer, K., 2006. The German Version of the Falls Efficacy Scale-International Version (FES-I). Z. Für Gerontol. Geriatr. 39, 297-300. https://doi.org/10.1007/s00391-006-0400-8

Dite, W., Temple, V.A., 2002. A clinical test of stepping and change of direction to identify multiple falling older adults. Arch. Phys. Med. Rehabil. 83, 1566-1571. https://doi.org/10.1053/apmr.2002.35469

Duncan, R.P., Earhart, G.M., 2013. Four Square Step Test Performance in People With Parkinson Disease. J. Neurol. Phys. Ther. 37, 2-8. https://doi.org/10.1097/NPT.0b013e31827f0d7a

Flansbjer, U.-B., Lexell, J., Holmbäck, A.M., Downham, D., Patten, C., 2005. RELIABILITY OF GAIT PERFORMANCE TESTS IN MEN AND WOMEN WITH HEMIPARESIS AFTER STROKE. J. Rehabil. Med. 37, 75-82. https://doi.org/10.1080/16501970410017215

Franchignoni, F., Martignoni, E., Ferriero, G., Pasetti, C., 2005. Balance and fear of falling in Parkinson's disease. Parkinsonism Relat. Disord. 11, 427–433. https://doi.org/10.1016/j.parkreldis.2005.05.005

Giladi, N., Shabtai, H., Simon, E.S., Biran, S., Tal, J., Korczyn, A.D., 2000. Construction of freezing of gait questionnaire for patients with Parkinsonism. Parkinsonism Relat. Disord. 6, 165-170. https://doi.org/10.1016/S1353-8020(99)00062-0

Global, regional, and national burden of Parkinson's disease, 1990-2016: a systematic analysis for the Global Burden of Disease Study 2016., 2018. . Lancet Neurol. 17, 939-953. https://doi.org/10.1016/S1474-4422(18)30295-3

Grimes, D., Fitzpatrick, M., Gordon, J., Miyasaki, J., Fon, E.A., Schlossmacher, M., Suchowersky, O., Rajput, A., Lafontaine, A.L., Mestre, T., Appel-Cresswell, S., Kalia, S.K., Schoffer, K., Zurowski, M., Postuma, R.B., Udow, S., Fox, S., Barbeau, P., Hutton, B., 2019. Canadian guideline for Parkinson disease. Can. Med. Assoc. J. 191, E989-E1004. https://doi.org/10.1503/cmaj.181504

Hoehn, M.M., Yahr, M.D., 1967. Parkinsonism: onset, progression, and mortality. Neurology 17, 427-427. https://doi.org/10.1212/WNL.17.5.427

Hofheinz, M., Schusterschitz, C., 2010. Dual task interference in estimating the risk of falls and measuring change: a comparative, psychometric study of four measurements. Clin. Rehabil. 24, 831-842. https://doi.org/10.1177/0269215510367993

Huang, S.-L., Hsieh, C.-L., Wu, R.-M., Tai, C.-H., Lin, C.-H., Lu, W.-S., 2011. Minimal Detectable Change of the Timed "Up & Go" Test and the Dynamic Gait Index in People With Parkinson Disease. Phys. Ther. 91, 114-121. https://doi.org/10.2522/ptj.20090126

Hughes, A.J., Daniel, S.E., Kilford, L., Lees, A.J., 1992. Accuracy of clinical diagnosis of idiopathic Parkinson's disease: a clinico-pathological study of 100 cases. J. Neurol. Neurosurg. Psychiatry 55, 181-184. https://doi.org/10.1136/jnnp.55.3.181

Jenkinson, C., Fitzpatrick, R., Peto, V., Greenhall, R., Hyman, N., 1997. The Parkinson's Disease Questionnaire (PDQ-39): development and validation of a Parkinson's disease summary index score. Ageing 26, 353-357. https://doi.org/10.1093/ageing/26.5.353

Keus, S., Munneke, M., Graziano, M., Paltamaa, J., Pelosin, E., Domingos, J., Brühlmann, S., Ramaswamy, B., Prins, J., Struiksma, C., Rochester, L., Nieuwboer, A., Bloem, B., 2014. European Physiotherapy Guideline in Idiopathic Parkinson's Disease.

Knox, P.C., Wolohan, F.D.A., 2015. Temporal Stability and the Effects of Training on Saccade Latency in "Express Saccade Makers." PLOS ONE 10, e0120437. https://doi.org/10.1371/journal.pone.0120437

Kühner, C., Bürger, C., Keller, F., Hautzinger, M., 2007. Reliability and validity of the revised Beck Depression Inventory (BDI-II): Findings from German-speaking samples. Nervenarzt 78, 651-656. https://doi.org/10.1007/s00115-006-2098-7

Leddy, A.L., Crowner, B.E., Earhart, G.M., 2011. Functional Gait Assessment and Balance Evaluation System Test: Reliability, Validity, Sensitivity, and Specificity for Identifying Individuals With Parkinson Disease Who Fall. Phys. Ther. 91, 102-113. https://doi.org/10.2522/ptj.20100113

Lindholm, B., Nilsson, M.H., Hansson, O., Hagell, P., 2018. The clinical significance of 10-m walk test standardizations in Parkinson's disease. J. Neurol. 265, 1829-1835. https://doi.org/10.1007/s00415-018-8921-9

Mak, M.K.Y., Pang, M.Y.C., 2009. Fear of falling is independently associated with recurrent falls in patients with Parkinson's disease: a 1-year prospective study. J. Neurol. 256, 1689-1695. https://doi.org/10.1007/s00415-009-5184-5

Maranhão-Filho, P.A., Maranhão, E.T., Lima, M.A., Silva, M.M. da, 2011. Rethinking the neurological examination II: dynamic balance assessment. Arq. Neuropsiquiatr. 69, 959-963. https://doi.org/10.1590/S0004-282X2011000700022

Martinez-Martin, P., Reddy, P., Antonini, A., Henriksen, T., Katzenschlager, R., Odin, P., Todorova, A., Naidu, Y., Tluk, S., Chandiramani, C., Martin, A., Chaudhuri, K.R., 2011. Chronic Subcutaneous Infusion Therapy with Apomorphine in Advanced Parkinson's Disease Compared to Conventional Therapy: A Real Life Study of Non Motor Effect. J. Park. Dis. 1, 197-203. https://doi.org/10.3233/JPD-2011-11037

Martinez-Martin, P., Serrano-Dueñas, M., Forjaz, M.J., Serrano, M.S., 2007. Two questionnaires for Parkinson's disease: are the PDQ-39 and PDQL equivalent? Qual. Life Res. 16, 1221-1230. https://doi.org/10.1007/s11136-007-9224-2

Matsumoto, H., Terao, Y., Furubayashi, T., Yugeta, A., Fukuda, H., Emoto, M., Hanajima, R., Ugawa, Y., 2011. Small saccades restrict visual scanning area in Parkinson's disease. Mov. Disord. Off. J. Mov. Disord. Soc. 26, 1619-1626. https://doi.org/10.1002/mds.23683

Mong, Y., Teo, T.W., Ng, S.S., 2010. 5-Repetition Sit-to-Stand Test in Subjects With Chronic Stroke: Reliability and Validity. Arch. Phys. Med. Rehabil. 91, 407-413. https://doi.org/10.1016/j.apmr.2009.10.030

Morris, S., Morris, M.E., Iansek, R., 2001. Reliability of Measurements Obtained With the Timed "Up & Go" Test in People With Parkinson Disease. Phys. Ther. 81, 810-818. https://doi.org/10.1093/ptj/81.2.810

National Institute for Health and Care Excellence (Great Britain), 2017. Parkinson's disease in adults: diagnosis and management : full guideline.

Olson, M., Lockhart, T.E., Lieberman, A., 2019. Motor Learning Deficits in Parkinson's Disease (PD) and Their Effect on Training Response in Gait and Balance: A Narrative Review. Front. Neurol. 10, 62. https://doi.org/10.3389/fneur.2019.00062

Petersen, C., Steffen, T., Paly, E., Dvorak, L., Nelson, R., 2017. Reliability and Minimal Detectable Change for Sit-to-Stand Tests and the Functional Gait Assessment for Individuals With Parkinson Disease. J. Geriatr. Phys. Ther. 40, 223-226. https://doi.org/10.1519/JPT.0000000000000102

Peto, V., Jenkinson, C., Fitzpatrick, R., Greenhall, R., 1995. The development and validation of a short measure of functioning and well being for individuals with Parkinson's disease. Qual. Life Res. 4, 241-248. https://doi.org/10.1007/BF02260863

Podsiadlo, D., Richardson, S., 1991. The Timed "Up & Go": A Test of Basic Functional Mobility for Frail Elderly Persons. J. Am. Geriatr. Soc. 39, 142-148. https://doi.org/10.1111/j.1532-5415.1991.tb01616.x

Reed-Jones, R.J., Powell, D.W., 2017a. The effects of gaze stabilization on gait parameters in individuals with Parkinson's disease. Neurosci. Lett. 655, 156-159. https://doi.org/10.1016/j.neulet.2017.07.013

Reed-Jones, R.J., Powell, D.W., 2017b. The effects of gaze stabilization on gait parameters in individuals with Parkinson's disease. Neurosci. Lett. 655, 156-159. https://doi.org/10.1016/j.neulet.2017.07.013

Scherfer, E., Bohls, C., Freiberger, E., Heise, K.-F., Hogan, D., 2006. Berg Balance Scale - German version. physioscience 2, 59-66. https://doi.org/10.1055/s-2006-926833

Schlenstedt, C., Brombacher, S., Hartwigsen, G., Weisser, B., Möller, B., Deuschl, G., 2016. Comparison of the Fullerton Advanced Balance Scale, Mini-BESTest, and Berg Balance Scale to Predict Falls in Parkinson Disease. Phys. Ther. 96, 494-501. https://doi.org/10.2522/ptj.20150249

Schrag, A., 2000. What contributes to quality of life in patients with Parkinson's disease? J. Neurol. Neurosurg. Psychiatry 69, 308-312. https://doi.org/10.1136/jnnp.69.3.308

Shumway-Cook, A., Brauer, S., Woollacott, M., 2000. Predicting the Probability for Falls in Community-Dwelling Older Adults Using the Timed Up & Go Test. Phys. Ther. 80, 896-903. https://doi.org/10.1093/ptj/80.9.896

Silva de Lima, A.L., Evers, L.J.W., Hahn, T., de Vries, N.M., Daeschler, M., Boroojerdi, B., Terricabras, D., Little, M.A., Bloem, B.R., Faber, M.J., 2018. Impact of motor fluctuations on real-life gait in Parkinson's patients. Gait Posture 62, 388-394. https://doi.org/10.1016/j.gaitpost.2018.03.045

Skelton, D.A., Becker, C., Lamb, S.E., Close, J.C.T., Zijlstra, W., Yardley, L., Todd, C.J., 2004. Prevention of Falls Network Europe: a thematic network aimed at introducing good practice in effective falls prevention across Europe. Eur. J. Ageing 1, 89-94. https://doi.org/10.1007/s10433-004-0008-z

Steffen, T., Seney, M., 2008a. Test-Retest Reliability and Minimal Detectable Change on Balance and Ambulation Tests, the 36-Item Short-Form Health Survey, and the Unified Parkinson Disease Rating Scale in People With Parkinsonism. Phys. Ther. 88, 733-746. https://doi.org/10.2522/ptj.20070214

Steffen, T., Seney, M., 2008b. Test-Retest Reliability and Minimal Detectable Change on Balance and Ambulation Tests, the 36-Item Short-Form Health Survey, and the Unified Parkinson Disease Rating Scale in People With Parkinsonism. Phys. Ther. 88, 733-746. https://doi.org/10.2522/ptj.20070214

Stuart, S., Lord, S., Galna, B., Rochester, L., 2018. Saccade frequency response to visual cues during gait in Parkinson's disease: the selective role of attention. Eur. J. Neurosci. 47, 769-778. https://doi.org/10.1111/ejn.13864

Thieme, H., Ritschel, C., Zange, C., 2009. Functional Gait Assessment - German version. physioscience 5, 5-11. https://doi.org/10.1055/s-0028-1109151

Tinetti, M.E., Richman, D., Powell, L., 1990. Falls Efficacy as a Measure of Fear of Falling. J. Gerontol. 45, P239-P243. https://doi.org/10.1093/geronj/45.6.P239

Tombaugh, T.N., McIntyre, N.J., 1992. The Mini-Mental State Examination: A Comprehensive Review. J. Am. Geriatr. Soc. 40, 922-935. https://doi.org/10.1111/j.1532-5415.1992.tb01992.x

Tönges, L., Bartig, D., Muhlack, S., Jost, W., Gold, R., Krogias, C., 2019. Characteristics and dynamics of inpatient treatment of Parkinson's disease patients in Germany: analysis of 1.5 million patient cases from 2010 to 2015. Nervenarzt 90, 167-174. https://doi.org/10.1007/s00115-018-0590-5

Visser, M., Leentjens, A.F.G., Marinus, J., Stiggelbout, A.M., van Hilten, J.J., 2006. Reliability and validity of the Beck depression inventory in patients with Parkinson's disease. Mov. Disord. 21, 668-672. https://doi.org/10.1002/mds.20792

Vogler, A., Janssens, J., Nyffeler, T., Bohlhalter, S., Vanbellingen, T., 2015. German translation and validation of the "freezing of gait questionnaire" in patients with Parkinson's disease. Park. Dis. 2015, 982058. https://doi.org/10.1155/2015/982058

Wrisley, D.M., Marchetti, G.F., Kuharsky, D.K., Whitney, S.L., 2004. Reliability, internal consistency, and validity of data obtained with the functional gait assessment. Phys. Ther. 84, 906-918.

Yang, Y., Wang, Y., Zhou, Y., Chen, C., Xing, D., 2016. Reliability of functional gait assessment in patients with Parkinson disease: Interrater and intrarater reliability and internal consistency. Medicine (Baltimore) 95, e4545. https://doi.org/10.1097/MD.0000000000004545

Yang, Y., Wang, Y., Zhou, Y., Chen, C., Xing, D., Wang, C., 2014. Validity of the Functional Gait Assessment in Patients With Parkinson Disease: Construct, Concurrent, and Predictive Validity. Phys. Ther. 94, 392-400. https://doi.org/10.2522/ptj.20130019

Yardley, L., Beyer, N., Hauer, K., Kempen, G., Piot-Ziegler, C., Todd, C., 2005. Development

and initial validation of the Falls Efficacy Scale-International (FES-I). Ageing 34, 614-619. https://doi.org/10.1093/ageing/afi196

Effects of an activity-oriented, physiotherapeutic training programme with and gaze movement training on dynamic balance and fall risk in individuals with Idiopathic Parkinson's Syndrome:

A pilot randomised controlled trial

Instructor Manual

for physiotherapists

Intervention group

REHA ZENTRUM MÜNSTER

Klinikum für Rehabilitation in Tirol

Instructor Manual for Physiotherapists

General notes:

- Before the start of the programme, ask about the current health status of the participants.
- The exercises should be performed with the aim of achieving the greatest possible amplitude.
- Participants are allowed to take a break at any time.
- Use physiotherapy equipment as available e. g., chairs, stools, gym mats, gym balls, tennis balls, cones, ropes, step stools, dumbbells, balance boards, coordination hoops, rice, and beans bags.
- Add walking training with different variations (e.g., heel to toe, lateral walking, walking in reverse, crossing obstacles during walking, walking through a slalom course, changing walking directions and speed)
- Add stair climbing training
- The exercises to be carried out are constantly adapted to the participants individually, increasing movement speed as possible, in the course of the 4-week therapy within the given standardised framework.
- The documentation template starts on page 24. As the performing physiotherapist, please document here the exercises performed in the respective weeks with the increase (progression) performed (I-III) and the gaze fixation points performed (A-C).
- If participants feel insecure, they can always hold on to the wall or a chair. Assistance can be given if needed.

Warm up

Exercise 1: Head flexion and extension in the seat

**Exercise setting**		**Gaze fixation point**
Starting position	Sit on a stool/chair in the front area;<br>Feet placed hip-width apart on the floor	Centre of the crosshairs
End position	Maximum flexion of the cervical spine<br>Maximum extension of the cervical spine	View towards the triangle (below)<br>View towards the triangle (top)
Progression options	I: In addition, raise and lower the arms to 90° (GHG) with the movement.<br>II: In addition, raise the arms to 90° (GHG) and hold.<br>III: In addition, raise the arms to 180° (GHG) and hold.	A: View in the direction of the quadrilateral (below & above)<br>B: View in the direction of the point (below & above)
Number of repetitions	5 x each direction; hold each for 5 sec. in the end position	

Exercise 2: Head rotation in the seat

**Exercise setting**		**Gaze fixation point**
Starting position	Sit on a stool/chair in the front area;<br>Feet placed hip-width apart on the floor	Centre of the crosshairs
End position	Rotation of the head to the right<br>Rotation of the head to the left	View towards the triangle (right)<br>View towards the triangle (left)
Progression options	I: In addition, raise and lower the arms to 90° (GHG) with the movement.<br>II: In addition, raise the arms to 90° (GHG) and hold.<br>III: In addition, raise the arms to 180° (GHG) and hold.	A: View in the direction of the quadrilateral (right & left)<br>B: View towards point (right & left)
Number of repetitions	5 x each side; hold each side in the end position for 5 sec.	

Main part

Exercise 3: Sit-to-Stand

**Exercise setting**		**Gaze fixation point**
Starting position	Sit on a stool/chair in the front area;<br>Feet placed hip-width apart on the floor	Centre of the crosshairs
End position	Standing position in front of the chair	Centre of the crosshairs
Progression options	I: right foot forward, left foot further back<br>II: left foot forward, right foot further back<br>III: both feet further back	A: Triangle<br>B: Square<br>C: Point
Number of repetitions	10 x; hold each for 5 sec. in the end position	

Exercise 4: Head flexion and extension in standing position

**Exercise setting**		**Gaze fixation point**
Starting position	Stance: feet hip-width apart on the floor, knees slightly bent	Centre of the crosshairs
End position	Maximum flexion of the cervical spine<br>Maximum extension of the cervical spine	View towards the triangle (below)<br>View towards the triangle (above)
Progression options	I: In addition, raise and lower the arms to 90° (GHG) with the movement.<br>II: In addition, raise the arms to 90° (GHG) and hold.<br>III: In addition, raise the arms to 180° (GHG) and hold.	A: View in the direction of the quadrilateral (below & above)<br>B: View in the direction of the point (below & above)
Number of repetitions	5 x each side; hold each side in the end position for 5 sec.	

Exercise 5: Head rotation in standing position

**Exercise setting**		**Gaze fixation point**
Starting position	Stance: feet placed hip-width apart on the floor, knees slightly bent	Centre of the crosshairs
End position	Rotation of the cervical spine to the right<br>Rotation of the cervical spine to the left	View towards the triangle (right)<br>View towards the triangle (left)
Progression options	I: In addition, raise and lower the arms to 90° (GHG) with the movement.<br>II: In addition, raise and lower the arms with the movement to 90° (GHG) and rotate the torso with it.<br>III: In addition, raise and lower the arms with the movement to 180° (GHG).	A: View in the direction of the quadrilateral (right & left)<br>B: View towards point (right & left)
Number of repetitions	5 x each side; hold each side in the end position for 5 sec.	

Exercise 6: Step forward

**Exercise setting**		**Gaze fixation point**
Starting position	Stance: feet hip-width apart on the floor, knees slightly bent	Centre of the crosshairs
End position	Right foot forward in step position;<br>Left foot forward in step position	Centre of the crosshairs
Progression options	I: larger step<br>II: Lunge forward with arms 90° ABD (GHG)<br>III: Lunge up with arms 90° flexion (GHG) and increase speed	A: Triangle (top)<br>B: Square (top)<br>C: Point (top)
Number of repetitions	5 x each side; hold each side in the end position for 5 sec.	

Exercise 7: Step to the side

**Exercise setting**		**Gaze fixation point**
Starting position	Stance: feet hip-width apart on the floor, knees slightly bent	Centre of the crosshairs
End position	Step to the right side with the right foot;<br>Step to the left side with the left foot	View towards the triangle (right)<br>View towards the triangle (left)
Progression options	I: larger step to the side<br>II: Lunge to the side with arms 90° ABD (GHG)<br>III: Lunge to the side with arms 90° ABD (GHG) and foot pointing in the direction of the lunge	A: Square (right & left)<br>B: Point (right & left)
Number of repetitions	5 x each side; hold each side in the end position for 5 sec.	

Exercise 8: Step backwards

**Exercise setting**		**Gaze fixation point**
Starting position	Stance: feet hip-width apart on the floor, knees slightly bent	Centre of the crosshairs
End position	Right foot backwards,<br>Left foot backwards	Centre of the crosshairs
Progression options	I: larger step backwards<br>II: Lunge backwards with arms 90° ABD (GHG)<br>III: Lunge backwards bringing the arms 180° flexion (GHG)	A: Triangle (top)<br>B: Square (top)<br>C: Point (top)
Number of repetitions	5 x each side; hold each side in the end position for 5 sec.	

Exercise 9: Step diagonally forward and back (Left diagonal)

**Exercise setting**		**Gaze fixation point**
Starting position	Stance: feet hip-width apart on the floor, knees slightly bent	Triangle left
End position	Right foot diagonally forward (left); right foot diagonally back (right)	Triangle left
Progression options	I: larger step forward or backward<br>II: Lunge diagonally forward or backward with arms 90° ABD (GHG)<br>III: Lunge backwards bringing the arms 180° flexion (GHG)	A: Square (left)<br>B: Point (left)<br>C: Cross green (left)
Number of repetitions	5 x each side; hold each side in the end position for 5 sec.	

Exercise 10: Step diagonally forward and back (Right diagonal)

Exercise setting		Gaze fixation point
Starting position	Stance: feet hip-width apart on the floor, knees slightly bent	Triangle right
End position	Left foot diagonally forward (right); left foot diagonally back (left)	Triangle right
Progression options	I: larger step forward or backward<br>II: Lunge forward or backward with arms 90° ABD (GHG)<br>III: Lunge backwards bringing the arms 180° flexion (GHG)	A: Square (right)<br>B: Point (right)<br>C: Cross green (right)
Number of repetitions	5 x each side; hold each side in the end position for 5 sec.	

Exercise 11: Squats

Exercise setting		Gaze fixation point
Starting position	Stance: feet placed wide-legged on the floor, knees bent	Centre crosshairs
End position	Bring buttocks low, back in upright position	Hold on the centre
Progression options	I: go deeper into the squat<br>II: Arms in U-hold (GHG: 90° ABD, HUG: 90° Flex) at the side of the body and stretch forward with the knee bend (GHG 90°).<br>III: Narrow-gauge stand in combination with II	A: Triangle down<br>B: View from triangle (right) to triangle (left)<br>C: View from rectangle (right) to rectangle (left)
Number of repetitions	10 x; hold each for 5 sec. in the end position	

Exercise 12: Archery sideways

**Exercise setting**		**Gaze fixation point**
Starting position	Stance: feet hip-width apart on the floor, knees bent	Centre crosshairs
End position	Right arm in 90° ABD (GHG), left arm touches right hand and slowly goes back; left arm in 90° ABD (GHG), right arm touches left hand and slowly goes back	Triangle (right & left)
Progression options	I: deeper into the knees<br>II: narrow gauge<br>III: Tandem stand	A: Square (right & left)<br>B: View from quadrilateral (right) to quadrilateral (left)<br>C: View from circle (right) to circle (left)
Number of repetitions	5 x each side; hold each side in the end position for 5 sec.	

Exercise 13: Squats wide-legged, head flexion, extension, rotation

Exercise setting		Gaze fixation point
Starting position	Stance: feet placed wide-legged on the floor, knees bent	Centre crosshairs
End position	Bring the buttocks low, back in an upright position, flexion and extension of the head.	Triangle (top& bottom)
Progression options	I: Arms 180° ABD (GHG), flexion and extension head<br>II: Lead right arm in 90° ABD (GHG), hold left arm in 180° ABD (GHG); lead left arm in 90° ABD (GHG), hold right arm in 180° ABD (GHG).	A: Square (top & bottom)<br>B: View from quadrilateral (top) to quadrilateral (right)
Number of repetitions	5 x with each arm; hold each arm in the end position for 5 sec.	

Exercise 14: Squats narrow gauge, head flexion and extension, rotation

**Exercise setting**		**Gaze fixation point**
Starting position	Stance: feet placed narrowly on the floor, knees bent	Centre crosshairs
End position	Bring the buttocks low, back in an upright position, flexion and extension of the head.	Triangle (top& bottom)
Progression options	I: Arms 180° ABD (GHG), flexion and extension head<br>II: Lead right arm in 90° ABD (GHG), hold left arm in 180° ABD (GHG); lead left arm in 90° ABD (GHG), hold right arm in 180° ABD (GHG).	A: Square (top & bottom)<br>B: View from quadrilateral (top) to quadrilateral (right)
Number of repetitions	5 x with each arm; hold each arm in the end position for 5 sec.	

Exercise 15: Raise knee

**Exercise setting**		**Gaze fixation point**
Starting position	Stance: feet hip-width apart on the floor, knees slightly bent	Centre crosshairs
End position	Stance left leg stable, bring right leg into 90° hip flexion;<br>Stance right leg stable, bring left leg into 90° hip flexion	From the triangle (bottom) to the centre of the crosshairs
Progression options	I: bring both arms into 90° ABD (GHG)<br>II: additionally bring the head into flexion with the movement<br>III: Increase the speed	A: Triangle (bottom) to triangle (top)<br>B: Square (bottom) to square (top)<br>C: Circle (bottom) to circle (top)
Number of repetitions	5 x each side; hold each side in the end position for 5 sec.	

Exercise 16: Lift feet off the floor

**Exercise setting**		**Gaze fixation point**
Starting position	Stance: Place feet in a wide-legged stance, knees slightly bent.	Centre crosshairs
End position	Stand with left leg stable, lift right foot slightly off floor;<br>Stance right leg stable, lift left foot slightly off floor	Centre crosshairs
Progression options	I: bring both arms into 90° (GHG) ABD, rotate the head to the right or left with the movement.<br>II: swing the arms far upwards with the movement (GHG: 180° Flex)<br>III: with the movement swing the arms staggered forwards or backwards (right foot, left arm forwards, right arm backwards and vice versa).	A: Triangle (right & left)<br>B: Square (right & left)<br>C: Circle (right & left)
Number of repetitions	5 x each side; hold each side in the end position for 5 sec.	

Exercise 17: Letter Z

**Exercise setting**		**Gaze fixation point**
Starting position	Stance: Bring feet to a hip-width stance, knees slightly bent.	Green cross (top left)
End position	Move a thought Z along the grid with the eyes and the head movement	Green cross (bottom right)
Progression options	I: Standing in lunge<br>II: Turn around at end point and drive off backwards<br>III: In addition, walk the imaginary Z on the floor; step to the right, add second leg; step diagonally back to the left, add second leg; step to the right, add second leg	
Number of repetitions	5 x	

Exercise 18: Letter M

**Exercise setting**		**Gaze fixation point**
Starting position	Stance: Bring feet to a hip-width stance, knees slightly bent.	Green cross (bottom left)
End position	Move an imaginary M along the grid with the eyes and the head movement.	Green cross (bottom right)
Progression options	I: Standing in lunge<br>II: Turn around at end point and drive off backwards<br>III: In addition, walk the imaginary M on the floor; step forward, add second leg; step diagonally back to the right, add second leg; step diagonally forward to the right, add second leg; step back, add second leg.	
Number of repetitions	5 x	

Exercise 19: Letter N

Exercise setting		Gaze fixation point
Starting position	Stance: Bring feet to a hip-width stance, knees slightly bent.	Green cross (bottom left)
End position	Move an imaginary N along the grid with the eyes and the head movement.	Red dot (top)
Progression options	I: Standing in lunge<br>II: Turn around at end point and drive off backwards<br>III: In addition, walk the imaginary N on the floor; step forward, add second leg; step diagonally backwards to the right, add second leg; step forward, add second leg.	
Number of repetitions	5 x	

Exercise 20: Letter W

**Exercise setting**		**Gaze fixation point**
Starting position	Stance: Bring feet to a hip-width stance, knees slightly bent.	Green cross (top left)
End position	Along the grid drive a thought W with the eyes and the head movement	Green cross (top right)
Progression options	I: Standing in lunge<br>II: Turn around at end point and drive off backwards<br>III: In addition, walk the imaginary W on the floor; step diagonally backwards to the right, add second leg; step diagonally forwards to the right, add second leg; step diagonally backwards to the right, add second leg; step diagonally forwards to the right, add second leg.	
Number of repetitions	5 x	

Cool Down / Warm Down

Exercise 21: Shoulders Circling

**Exercise setting**	
Starting position	Sitting relaxed on the chair
End position	Circle shoulders forward and backward

Exercise 22: Loosen arms

**Exercise setting**	
Starting position	Stance: Bring feet to a hip-width stance, knees slightly bent.<br>If necessary, sit on a stool/chair in the front area;<br>Shoulders loose, arms beside the body
End position	Raise arms far upwards with inhalation; swing loosely downwards with exhalation

Exercise 23: Loosen the thoracic spine

**Exercise setting**	
Starting position	Sitting relaxed on the chair
End position	Arms 90° (GHG) ABD; rotation to the right; rotation to the left

Exercise 24: Turning the head

**Exercise setting**	
Starting position	Sitting relaxed on the chair, eyes closed
End position	Rotate head relaxed forwards, backwards, to the side

Exercise 25: Close eyes

**Exercise setting**	
Starting position	Sitting relaxed on the chair
End position	Close your eyes, relax and breathe deeply in and out.

Exercise 26: Eye Relaxation

**Exercise setting**	
Starting position	Sit on a stool/chair in the front area;<br>Eyes closed
End position	Rub the palms together quickly and place the warm palms on the closed eyes

Study Diary - Week 1

From .... to ....

TN ID: ....

**On which days did the training take place?**	**Mo**	**Tue**	**Mi**	**Do**	**Fri**

**Which exercises were carried out? Please indicate the number of the exercises**<br><br>**(e.g.: Exercise 1, I, A)**	Exercise (number)	Level of difficulty (I-III, A-C)	Exercise (number)	Level of difficulty (I-III, A-C)

**Were there any difficulties when doing the exercises?**	yes	no

If so:

- **Write down the number of the exercise**
- **Briefly note the difficulty**

**Details of any falls**	Number of falls	Reason for the fall (e.g. dizziness, black ice, stumbling, etc.)
**During the training**		
**Outside the training**		

Study Diary - Week 2
From .... to ....

TN ID: ....

**On which days did the training take place?**	**Mo**	**Tue**	**Mi**	**Do**	**Fri**
**Which exercises were carried out? Please indicate the number of the exercises**<br>**(e.g.: Exercise 1, I, A)**	Exercise (number)	Level of difficulty (I-III, A-C)	Exercise (number)	Level of difficulty (I-III, A-C)	
**Were there any difficulties when doing the exercises?**	yes		no		
**If so:**<br>- **Write down the number of the exercise**<br>- **Briefly note the difficulty**					
**Details of any falls**	Number of falls	Reason for the fall (e.g. dizziness, black ice, tripping, etc.)			
**During the training**					
**Outside the training**					

Study Diary - Week 3
From .... to ....

TN ID: ....

On which days did the training take place?	Mo	Tue	Mi	Do	Fri

Which exercises were carried out? Please indicate the number of the exercises (e.g.: Exercise 1, I, A)	Exercise (number)	Level of difficulty (I-III, A-C)	Exercise (number)	Level of difficulty (I-III, A-C)

Were there any difficulties when doing the exercises?	yes	no

If so:

- **Write down the number of the exercise**
- **Briefly note the difficulty**

Details of any falls	Number of falls	Reason for the fall (e.g. dizziness, black ice, stumbling, etc.)
**During the training**		
**Outside the training**		

Study Diary - Week 4

From .... to ....

TN ID: ....

**On which days did the training take place?**	**Mo**	**Tue**	**Mi**	**Do**	**Fri**

**Which exercises were carried out? Please indicate the number of the exercises**<br>**(e.g.: Exercise 1, I, A)**	Exercise (number)	Level of difficulty (I-III, A-C)	Exercise (number)	Level of difficulty (I-III, A-C)

**Were there any difficulties when doing the exercises?**	yes	no

If so:

- **Write down the number of the exercise**
- **Briefly note the difficulty**

**Details of any falls**	Number of falls	Reason for the fall (e.g. dizziness, black ice, stumbling, etc.)
**During the training**		
**Outside the training**		

Effects of an activity-oriented physiotherapeutic training programme with and without gaze movement training on dynamic balance and fall risk in people with Idiopathic Parkinson Syndrome:

A pilot randomised controlled trial

Instructor Manual

for physiotherapists

Control group

REHA ZENTRUM MÜNSTER
Klinikum für Rehabilitation in Tirol

Instructor Manual for Physiotherapists

General notes:

- Before the start of the programme, ask about the current health status of the participants.
- The exercises should be performed with the aim of achieving the greatest possible amplitude.
- Participants are allowed to take a break at any time.
- Use physiotherapy equipment as available e. g., chairs, stools, gym mats, gym balls, tennis balls, cones, ropes, step stools, dumbbells, balance boards, coordination hoops, rice, and beans bags.
- Add walking training with different variations (e.g., heel to toe, lateral walking, walking in reverse, crossing obstacles during walking, walking through a slalom course, changing walking directions and speed)
- Add stair climbing training
- The exercises to be carried out are constantly adapted to the participants individually, increasing movement speed as possible, in the course of the 4-week therapy within the given standardised framework.
- The documentation template starts on page 23. As the performing physiotherapist, please document here the exercises performed in the respective weeks with the increase (progression) performed (I-III).
- If participants feel insecure, they can always hold on to the wall or a chair. Assistance can be given if needed.

Warm up

Exercise 1: Head flexion and extension in the seat

**Exercise setting**	
Starting position	Sit on a stool/chair in the front area;<br>Feet placed hip-width apart on the floor
End position	Maximum flexion of the cervical spine<br>Maximum extension of the cervical spine
Progression options	I: In addition, raise and lower the arms to 90° (GHG) with the movement.<br>II: In addition, raise the arms to 90° (GHG) and hold.<br>III: In addition, raise the arms to 180° (GHG) and hold.
Number of repetitions	5 x each direction; hold each for 5 sec. in the end position

Exercise 2: Head rotation in the seat

**Exercise setting**	
Starting position	Sit on a stool/chair in the front area;<br>Feet placed hip-width apart on the floor
End position	Rotation of the cervical spine to the right<br>Rotation of the cervical spine to the left
Progression options	I: In addition, raise and lower the arms to 90° (GHG) with the movement.<br>II: In addition, raise the arms to 90° (GHG) and hold.<br>III: In addition, raise the arms to 180° (GHG) and hold.
Number of repetitions	5 x each side; hold each side in the end position for 5 sec.

Main part

Exercise 3: Sit-to-Stand

Exercise setting	
Starting position	Sit on a stool/chair in the front area;<br>Feet placed hip-width apart on the floor
End position	Standing position in front of the chair
Progression options	I: right foot forward, left foot further back<br>II: left foot forward, right foot further back<br>III: both feet further back
Number of repetitions	10 x; hold each for 5 sec. in the end position

Exercise 4: Diagonal with the arms

**Exercise setting**	
Starting position	Stance: Feet placed hip-width apart on the floor, knees slightly bent;<br>Right or left arm on opposite pelvis (Ex/ADD/IR)
End position	Right or left arm stretched upwards (Flex/ABD/AR)
Progression options	I: Knees bent lower<br>II: Additionally with small dumbbell / weight<br>III: Standing in tandem
Number of repetitions	5 x each direction; hold each for 5 sec. in the end position

Exercise 5: Rotation in standing position

**Exercise setting**	
Starting position	Stance: feet hip-width apart on the floor, knees slightly bent; arms abducted to 90° (GHG).
End position	Rotation of the upper body and arms to the right as far as possible<br>Rotation of the upper body and arms to the left as far as possible
Progression options	I: Arms in U-position (GHG: 90° ABD, HUG: 90° Flex)<br>II: Standing in lunge<br>III: Arms at the beginning 180° flexion (GHG); with the rotation to 90° ABD (GHG).
Number of repetitions	5 x each side; hold each side in the end position for 5 sec.

Exercise 6: Step forward

**Exercise setting**	
Starting position	Stance: feet placed hip-width apart on the floor, knees slightly bent
End position	Right foot forward in step position;<br>Left foot forward in step position
Progression options	I: larger step<br>II: Lunge forward with arms 90° ABD (GHG)<br>III: Lunge up with arms 90° flexion (GHG) and increase speed
Number of repetitions	5 x each side; hold each side in the end position for 5 sec.

Exercise 7: Step to the side

**Exercise setting**	
Starting position	Stance: feet hip-width apart on the floor, knees slightly bent
End position	Right foot Step to the side on the right;<br>left foot Step to the side on the left;
Progression options	I: larger step to the side<br>II: Lunge to the side with arms 90° ABD (GHG)<br>III: Lunge to the side with arms 90° ABD (GHG) and foot pointing in the direction of the lunge.
Number of repetitions	5 x each side; hold each side in the end position for 5 sec.

Exercise 8: Step backwards

**Exercise setting**	
Starting position	Stance: feet hip-width apart on the floor, knees slightly bent
End position	Right foot Step backwards;<br>left foot step backwards
Progression options	I: larger step backwards<br>II: Lunge backwards with arms 90° ABD (GHG)<br>III: Lunge backwards while bringing the arms into 180° flexion (GHG).
Number of repetitions	5 x each side; hold each side in the end position for 5 sec.

Exercise 9: Step diagonally forward and back (Left diagonal)

**Exercise setting**	
Starting position	Stance: feet hip-width apart on the floor, knees slightly bent
End position	Right foot forward; right foot backward
Progression options	I: larger step forward or backward<br>II: Lunge diagonally forward or backward with arms 90° ABD (GHG)<br>III: Lunge backwards while bringing the arms into 180° flexion (GHG).
Number of repetitions	5 x each side; hold each side in the end position for 5 sec.

Exercise 10: Step diagonally forward and back (Right diagonal)

**Exercise setting**	
Starting position	Stance: feet hip-width apart on the floor, knees slightly bent
End position	Left foot forward; left foot backward
Progression options	I: larger step forward or backward<br>II: Lunge forward or backward with arms 90° ABD (GHG)<br>III: Lunge backwards bringing the arms into 180° flexion (GHG).
Number of repetitions	5 x each side; hold each side in the end position for 5 sec.

Exercise 11: Squats

**Exercise setting**	
Starting position	Stance: feet placed wide-legged on the floor, knees bent
End position	Bring buttocks low, back upright position, rotation head (right & left)
Progression options	I: go deeper into the squat<br>II: Arms in U-hold (GHG: 90° ABD, HUG: 90° Flex) at the side of the body and extend forward with the knee bend (GHG: 90° Flex).<br>III: Narrow-gauge stand in combination with II
Number of repetitions	10 x; hold each for 5 sec. in the end position

Exercise 12: Archery sideways

**Exercise setting**	
Starting position	Stance: feet hip-width apart on the floor, knees bent
End position	Right arm in 90° ABD (GHG), left arm touches right hand and slowly goes back; left arm in 90° ABD (GHG), right arm touches left hand and slowly goes back
Progression options	I: further to the knees<br>II: narrow gauge<br>III: Tandem stand
Number of repetitions	5 x each side; hold each side in the end position for 5 sec.

Exercise 13: Squats wide-legged, head flexion, extension, rotation

**Exercise setting**	
Starting position	Stance: feet placed wide-legged on the floor, knees bent
End position	Bring the buttocks low, back in an upright position, flexion and extension of the head.
Progression options	I: Arms 180° ABD (GHG), flexion and extension head<br>IIa): Lead right arm in 90° ABD (GHG), hold left arm in 180° ABD (GHG).<br>IIb): Lead left arm in 90° ABD (GHG), hold right arm in 180° ABD (GHG).
Number of repetitions	5 x with each arm; hold each arm in the end position for 5 sec.

Exercise 14: Squats narrow gauge, head flexion and extension, rotation

**Exercise setting**	
Starting position	Stance: feet placed narrowly on the floor, knees bent
End position	Bring the buttocks low, back in an upright position, flexion and extension of the head.
Progression options	I: Arms 180° ABD (GHG), flexion and extension head<br>IIa): Lead right arm in 90° ABD (GHG), hold left arm in 180° ABD (GHG).<br>IIb): Lead left arm in 90° ABD (GHG), hold right arm in 180° ABD (GHG).
Number of repetitions	5 x with each arm; hold each arm in the end position for 5 sec.

Exercise 15: Raise knee

**Exercise setting**	
Starting position	Stance: feet hip-width apart on the floor, knees slightly bent
End position	Stance left leg stable, bring right leg into 90° hip flexion;<br>Stance right leg stable, bring left leg into 90° hip flexion
Progression options	I: bring both arms into 90° ABD (GHG)<br>II: additionally bring the head into flexion with the movement<br>III: Increase the speed
Number of repetitions	5 x each side; hold each side in the end position for 5 sec.

Exercise 16: Lift feet off the floor

**Exercise setting**	
Starting position	Stance: Place feet in a wide-legged stance, knees slightly bent.
End position	Stand with left leg stable, lift right foot slightly off floor;<br>Stand with right leg stable, lift left foot slightly off floor;
Progression options	I: bring both arms into 90° (GHG) ABD, rotate the head to the right or left with the movement.<br>II: swing the arms far upwards with the movement (GHG: 180° Flex)<br>III: with the movement swing the arms staggered forwards or backwards (right foot, left arm forwards, right arm backwards and vice versa).
Number of repetitions	5 x each side; hold each side in the end position for 5 sec.

Exercise 17: Letter Z

**Exercise setting**	
Starting position	Stance: Bring feet to a hip-width stance, knees slightly bent.
End position	Walk an imaginary Z on the floor; step to the right, add second leg; step diagonally back to the left, add second leg; step to the right, add second leg
Progression options	I: walk thought Z in reverse step sequence
Number of repetitions	5 x

Exercise 18: Letter M

**Exercise setting**	
Starting position	Stance: Bring feet to a hip-width stance, knees slightly bent.
End position	On the floor walk an imaginary st; step forward, add second leg; step diagonally back to the right, add second leg; step diagonally forward to the right, add second leg; step backward, add second leg.
Progression options	I: walk the intended M in reverse step sequence
Number of repetitions	5 x

Exercise 19: Letter N

**Exercise setting**	
Starting position	Stance: Bring feet to a hip-width stance, knees slightly bent.
End position	Walk an imaginary N on the floor; step forward, add second leg; step diagonally back to the right, add second leg; step forward, add second leg.
Progression options	I: walk down thought N in reverse step sequence
Number of repetitions	5 x

Exercise 20: Letter W reversed

**Exercise setting**	
Starting position	Stance: Bring feet to a hip-width stance, knees slightly bent.
End position	On the floor walk an imaginary W; step diagonally backwards to the right, add second leg; step diagonally forwards to the right, add second leg; step diagonally backwards to the right, add second leg; step diagonally forwards to the right, add second leg.
Progression options	I: walk off thought W in reverse step sequence
Number of repetitions	5 x

Cool Down / Warm Down

Exercise 21: Shoulders Circling

**Exercise setting**	
Starting position	Sitting relaxed on the chair
End position	Circle shoulders forward and backward

Exercise 22: Loosen arms

**Exercise setting**	
Starting position	Stance: Bring feet to a hip-width stance, knees slightly bent.<br>If necessary, sit on a stool/chair in the front area;<br>Shoulders loose, arms beside the body
End position	Raise arms far upwards with inhalation; swing loosely downwards with exhalation

Exercise 23: Loosen the thoracic spine

**Exercise setting**	
Starting position	Sitting relaxed on the chair
End position	Arms 90° (GHG) ABD; rotation to the right; rotation to the left

Exercise 24: Turning the head

**Exercise setting**	
Starting position	Sitting relaxed on the chair, eyes closed

End position	Rotate head relaxed forwards, backwards, to the side

Study Diary - Week 1

From .... to ....

TN ID: ....

On which days did the training take place?	Mo	Tue	Mi	Do	Fri

Which exercises were carried out? Please indicate the number of the exercises<br>(e.g.: Exercise 1, I, A)	Exercise (number)	Level of difficulty (I-III, A-C)	Exercise (number)	Level of difficulty (I-III, A-C)

Were there any difficulties when doing the exercises?	yes	no

If so:

- **Write down the number of the exercise**
- **Briefly note the difficulty**

Details of any falls	Number of falls	Reason for the fall (e.g. dizziness, black ice, stumbling, etc.)
**During the training**		
**Outside the training**		

Study Diary - Week 2

From .... to ....

TN ID: ....

On which days did the training take place?	Mo	Tue	Mi	Do	Fri

Which exercises were carried out? Please indicate the number of the exercises (e.g.: Exercise 1, I, A)	Exercise (number)	Level of difficulty (I-III, A-C)	Exercise (number)	Level of difficulty (I-III, A-C)

Were there any difficulties when doing the exercises?	yes	no

If so:

- **Write down the number of the exercise**
- **Briefly note the difficulty**

Details of any falls	Number of falls	Reason for the fall (e.g. dizziness, black ice, stumbling, etc.)
During the training		
Outside the training		

Study Diary - Week 3

From .... to ....

TN ID: ....

**On which days did the training take place?**	**Mo**	**Tue**	**Mi**	**Do**	**Fri**

**Which exercises were carried out? Please indicate the number of the exercises**<br>**(e.g.: Exercise 1, I, A)**	Exercise (number)	Level of difficulty (I-III, A-C)	Exercise (number)	Level of difficulty (I-III, A-C)

**Were there any difficulties when doing the exercises?**	yes	no

If so:

- **Write down the number of the exercise**
- **Briefly note the difficulty**

**Details of any falls**	Number of falls	Reason for the fall (e.g. dizziness, black ice, stumbling, etc.)
**During the training**		
**Outside the training**		

Study Diary - Week 4

From .... to ....

TN ID: ....

**On which days did the training take place?**	**Mo**	**Tue**	**Mi**	**Do**	**Fri**

**Which exercises were carried out? Please indicate the number of the exercises**<br>**(e.g.: Exercise 1, I, A)**	Exercise (number)	Level of difficulty (I-III, A-C)	Exercise (number)	Level of difficulty (I-III, A-C)

**Were there any difficulties when doing the exercises?**	yes	no

If so:

- **Write down the number of the exercise**
- **Briefly note the difficulty**

**Details of any falls**	Number of falls	Reason for the fall (e.g. dizziness, black ice, stumbling, etc.)
**During the training**		
**Outside the training**		
