# Supplementary material for: Effects of activity-oriented physiotherapy with and without eye movement training on dynamic balance, functional mobility, and eye movements in patients with Parkinson’s disease: An assessor-blinded randomised controlled pilot trial
Source: PLoS One. 2024 Jun 14;19(6):e0304788. doi: 10.1371/journal.pone.0304788 (PMC11178185; doi:10.1371/journal.pone.0304788)
Supplement: Attachment — Submitted filename: Response to Reviewers.docx [file pone.0304788.s010.docx]

**PONE-D-23-31439**

Effects of activity-oriented physiotherapy with and without eye movement training on dynamic balance, functional mobility, and eye movements in patients with Parkinson’s disease: an assessor-blinded randomised controlled pilot trial

Dear Dr. Seebacher,

Thank you for submitting your manuscript to PLOS ONE. After careful consideration, we feel that it has merit but does not fully meet PLOS ONE’s publication criteria as it currently stands. Therefore, we invite you to submit a revised version of the manuscript that addresses the points raised during the review process.

As you can see, the reviewers were overall positive about this manuscript. Each reviewer raised several comments/questions that will improve the overall clarity of the manuscript. We look forward to receiving your revised manuscript.

*Dear Editor,*

*Dear Reviewers,*

*We thank the Editor and Reviewers for their valuable comments and feedback and for giving us a chance to submit a revised manuscript. Please find below our point-by-point responses. The main manuscript as well as S1 to S4 tables have been amended accordingly, with any changes highlighted to allow the Editor and Reviewers to check the adaptations made.*

*We thank the Editor and Reviewers for their time and effort and hope we have sufficiently responded to the Editor’s and Reviewers’ requests.*

*With kind regards,*

*Barbara Seebacher, on behalf of all the authors*

We look forward to receiving your revised manuscript.

Kind regards,

Eric R. Anson

Academic Editor

PLOS ONE

**Reviewers' comments:**

**Reviewer #1:**

*Dear Reviewer #1,*

*Thank you for reviewing our manuscript, acknowledging its value, and for offering your suggestions to enhance the quality of our manuscript. Please find our point-by-point response as follows.*

This is a well-written report of a small pilot study of eye movement and Parkinsons. The pilot study was well-designed and the statistical analysis thorough. Randomization was well described and allocation concealment was accomplished, although single blind. The authors did not try to oversell the results or overanalyze the small amount of data. They list the study limitations honestly. However, I was disappointed in the discussion. After a pilot study that is designed to determine if a full-scale study is feasible, I want to know what was learned, statistically, that will inform the future design of a clinical trial.

Point 1: What underlying assumptions can now be made about the sample size? Were the underlying parameters (variability, effect size, etc.) consistent with the other study that was done on progressive supranuclear palsy or other studies mentioned (albeit not on PD).

*Response:*

*Thank you for bringing up this relevant point. In response to your suggestions, we meticulously examined the data and incorporated a discussion on variability, along with a comparison to the comparable studies in patients with progressive supranuclear palsy, multiple sclerosis, and stroke within the discussion section. Additionally, we provided a sample size calculation for a full-scale RCT in the discussion.*

Point 2: The authors opine that they could not do subgroup analyses. Can they do them in a full-scale study? Would the sample size requirements be feasible? What subgroups? In other words, you did a feasibility study to inform you how to design a full-scale trial. Now I want to know how you would design a full-scale trial!

*Response:*

*Thank you for bringing up this important question. In response to your question and to provide clarity for the reader, we have incorporated the following information into the paragraph:*

*[…] it would be crucial to assess disparities in saccades and fixations between these subgroups in a larger RCT with pre-specified subgroup analysis. It would be imperative to gather pilot data on saccade latency and variability following AOPT-E in patients with PD experiencing FOG. This would allow to estimate effect sizes and variability, thus aiding in the sample size calculation for a subsequent RCT. Subgroups would include patients with PD with FOG and those without FOG. Conducting subgroup analyses inherently divides the sample size, reducing the statistical power for detecting effects within each subgroup [1]. Therefore, to ensure sufficient power for planned subgroup analyses, it becomes necessary to increase the total sample size. The extent of this increase depends on various factors unique to each subgroup, including effect sizes and variability.*

**Reviewer #2:**

*Dear Reviewer #2,*

*Dear Dr D'Cruz,*

*Thank you for reviewing our manuscript, recognising its relevance, and for providing valuable suggestions to improve its quality. Please find our point-by-point response as follows.*

In this study, Mildner and colleagues perform a pilot single-blind randomized controlled trial to investigate the added effects of eye movement training to functional gait and balance training. The study is innovative as it embeds technology into clinical therapy for people with Parkinson’s, and this generates feedback from participants that will be very useful for researchers and therapists. Further, training studies over longer periods like this one take considerable effort, so I commend the authors for this! On the other hand there are some inconsistencies with the (pre) registration, and some design issues that may impact the interpretation of the results. Additional clarification and discussion of these issues will be helpful for the subsequent reader. Kindly find detailed comments below:

Point 1. Line 47: The specific aspects of feasibility are not mentioned in the results, while they are included in the objectives.

*Response:*

*Thank you for providing this feedback. The sole reason for the omission of detailed feasibility results in the abstract was the constraint of the word count. Following your recommendations and those of Reviewer #3, we have moderated our statements regarding the results of primary and secondary outcomes. While we initially did not include effect size calculations based on a methods paper [2], we have now incorporated them. As a result, we have thoroughly revised our abstract, results and discussion to include specifics on the feasibility results.*

2. Line 105: There are some inconsistencies with the study registration such as whether it is a single center or multicenter study. Further in the registration the target sample size is 46 and the total sample size is 37. Here the target sample size is said to be 34 and only 25 participants are reported. Kindly clarify.

*Response:*

*Thank you for bringing up this point. We acknowledge that there appear to be inconsistencies with the study registration, particularly concerning the inclusion of a small study within this study, which will be published separately (https://drks.de/search/en/trial/DRKS00024982). In the section regarding the 'Health condition or problem studied,' we provided the information that the secondary study pertains to expert interviews involving professionals from various medical fields, with no specific health issue addressed. Additionally, under 'Interventions, Observational Groups,' we detailed the parameters of the secondary study, specifying a group of 12 experts to be interviewed for 45 minutes each.*

*This was also the reason for the discrepant target sample size. For the present study in people with Parkinson’s disease, the target sample size was 34. For the secondary study, the target sample size was 12. The decision to consolidate the registration for both studies was made because the studies were related insofar, as the secondary study involved qualitative interviews with experts on the barriers and facilitators of RCTs in rehabilitation centres. The secondary study's topic emerged due to the challenging circumstances brought about by the COVID-19 pandemic, particularly the strain it placed on health institutions, including our rehabilitation centre. Under such conditions, preparing for a study was arduous, let alone recruiting patients and involving already short-staffed personnel. Consequently, these two studies became somewhat intertwined, despite addressing different topics. Looking back, it would have been more appropriate to register them separately. We trust that this explanation addresses any lingering uncertainties.*

*Regarding reporting only 25 participants, our Principal Investigator made the decision to conclude the study due to the challenging circumstances at our rehabilitation centre, compounded by staff shortages. We have revised our statement at the beginning of the results section to provide clarity for the reader. The updated statement now reads as follows: ‘Due to COVID-19 restrictions, safety measures, and staffing limitations, recruiting the intended 34 patients with PD was not feasible. Out of the 25 randomised participants, 24 successfully completed the study and were included in the analysis […].’*

3. Line 147: If the participants were receiving many hours of rehabilitation each day besides the half hour for this study, how can one infer that the improvements at the end of the study are attributable to AOPT?

*Response:*

*Thank you for commenting on this There exists a substantial body of literature focusing on interventions implemented alongside either inpatient or outpatient multidisciplinary rehabilitation, such as robot-assisted gait training in neurorehabilitation centres [3]. Moreover, numerous studies have explored the supplementary effects of specific interventions when combined with other therapies, such as the additional benefits of kinesiotape alongside acupuncture [4]. While we acknowledge that inpatient rehabilitation likely exerted an effect, it needs to be considered that both study groups received equal amounts of inpatient rehabilitation. In response to your input and in consideration of comparing our findings with those from a study involving individuals with PSP, we have included the following sentence in the discussion section. ‘Differences between our study and theirs may stem from the settings: ours occurred in an inpatient rehabilitation setting with multidisciplinary rehabilitation, whereas theirs was in a motion analysis laboratory without additional therapies.’*

*In addition, we added the following information as a study limitation: ‘Third, this study took place within an inpatient rehabilitation facility, where patients were supplemented with AOPT-E and AOPT alongside multidisciplinary rehabilitation. Although we recognise that the multidisciplinary approach likely impacted the study outcomes, we presume that this influence was consistent across both groups under investigation.’*

4. Line 174: Was AOPT delivered in the same room or under the same light conditions as the AOPT-E group?

*Response:*

*Yes, AOPT and AOPT-E were delivered in the same room or under the same light conditions. We removed the specific information for the AOPT-E group and incorporated the following details into the intervention section: ‘AOPT-E and AOPT were delivered in a darkened room with white light at 300 lux.’ In addition, we clarified the information in the TIDieR checklist.*

5. Line 188: The AOPT-E group received a training that required dual-tasking – attention to the exercise as well as to the eye movements. Was there a control in terms of dual-task training for the AOPT group as well? Could this impact the results?

*Response:*

*Thank you for pointing out this discrepancy. Reviewer #3 also raised a similar concern. We intended to clarify this in the TIDieR 'Materials' section, where we specified the use of various physiotherapy equipment such as chairs, stools, gym mats, gym balls, tennis balls, cones, ropes, step stools, dumbbells, balance boards, coordination hoops, rice, and bean bags.’*

*Both groups of participants underwent training involving either single-task or dual-task exercises. In fact, both groups engaged in static and dynamic balance and walking exercises, as well as hand-eye coordination activities such as ball tossing and catching. We have now added the information to the manuscript and updated the TIDieR checklist to reflect these corrections.*

6. Line 200: In the trial registration FGA item 10 was to be assessed with the eye tracker, however there is no mention of this.

*Response:*

*Thank you for bringing attention to this oversight. Regrettably, we omitted reporting the results concerning FGA item 10 due to technical complications that impeded our ability to obtain precise data. The suboptimal lighting conditions on the stairway and persistent challenges with calibration during participants' stair climbing contributed to inaccuracies in the recorded gaze data. Consequently, conducting meaningful data analysis was not feasible. To improve transparency in our reporting, we have now incorporated FGA item 10 into the primary outcomes section. Furthermore, we have detailed the encountered problems in the results section for clarity: ‘Regarding FGA item 10, we faced technical challenges that hindered our ability to gather accurate data. Inadequate lighting conditions along the stairway and ongoing difficulties with calibration during participants' stair climbing resulted in inaccuracies in the recorded gaze data. As a result, conducting a meaningful data analysis was not possible.’*

7. Line 262: The way the results are written up, it sure sounds like the groups have been statistically compared. I was confused by phrases like the “superiority of the group”, and “between-group differences showed”, “improvements for AOPT-E were greater than AOPT”, and “statistical analyses showed”. If the groups were not statistically tested, I would temper these statements.

*Response:*

*We greatly appreciate this feedback. Reviewer #3 also expressed similar concerns. In response, we have revised our description throughout the manuscript to reflect the fact that the groups were not statistically compared.*

8. Line 262: I also find it strange that no effect sizes were collected – for the purpose of running a subsequent RCT, an estimate of the effect size between interventions would be helpful.

*Response:*

*Once more, we sincerely value this feedback. Reviewer #1 shared similar suggestions. Consequently, we have incorporated details on the estimation of effect sizes in the data analysis section. Furthermore, we have integrated effect sizes into Tables 2-4. Utilising the effect sizes derived from the primary outcome results, we determined the sample size for a full-scale RCT, with this information now included in the discussion.*

**Reviewer #3:**

*Dear Reviewer #3,*

*Thank you for reviewing our manuscript, recognising its relevance, and for providing valuable suggestions to improve its quality. Please find our point-by-point response as follows.*

This small pilot study (n=25) investigates an extremely under-researched topic area in PD rehab and I commend the authors for exploring the concept of visual training in their study. The manuscript is well written and clearly describes the results of a study comparing activity orientated physiotherapy with and without eye movement training.

My comments and suggestions are listed below:

1. Line 31 – “Outcomes were assessed at baseline, post-intervention, and follow-up…” – the methods paragraph of the abstract would benefit from some further punctuation and clarification of the follow-up timescale, including which outcomes were completed at follow-up and when the interviews were conducted.

*Response:*

*We appreciate your comment. Based on it, we have adjusted the methods section of the abstract, now stating: ‘Outcomes were assessed at baseline and post-intervention, including dynamic balance, walking speed, functional and dual-task mobility, ability to safely balance, health-related quality of life (HRQoL), depression, and eye movements (number and duration of fixations) using a mobile eye tracker. Freezing of gait (FOG), and falls-related self-efficacy were assessed at baseline, post-intervention, and 4-week follow-up. Feasibility was assessed using predefined criteria including recruitment, retention and adherence rates, adverse events, falls, and acceptability of the intervention, the latter evaluated at post-intervention utilising qualitative interviews.’*

2. I appreciate that there is often overlap between pilot and feasibility studies, therefore, it would be useful to maintain some consistency to the order and primary focus of your aims/objectives. For example, in your abstract you talk about describing changes in balance/gait etc and then refer to feasibility outcomes. In your background section, you refer to feasibility outcomes first and then outline hypotheses which you later state are not tested due to the pilot nature of the study. In your results section, you first address co-primary and secondary outcomes and then feasibility outcomes. A separate heading to highlight your feasibility analysis would also help direct the reader.

*Response:*

*Thank you for identifying these inconsistencies. Following your feedback, we have extensively reorganised and revised the manuscript. Throughout both the abstract and main text, we refer to the co-primary outcomes first, followed by the secondary outcomes, with feasibility addressed last, and detailed information on eye tracking provided thereafter. Additionally, in the methods section, we have introduced a new heading titled 'Feasibility criteria'. Within the statistical analysis section, we have included separate headings for 'Feasibility analysis' and 'Eye movement analysis'. Furthermore, within the results section, we have incorporated a heading specifically addressing 'Feasibility'.*

*Regarding our study aims and hypotheses, we acknowledge our previous misconception. In response to your input as well as that of Reviewer #2, we have clarified the study aims and have removed any references to hypotheses. Additionally, we have revised both the results and discussion sections to accurately reflect that hypothesis testing was not conducted.*

3. In addition to the previous point, I would question the inclusion of specific hypotheses in the background/introduction (lines 89-96) as you later acknowledge that hypothesis testing was not conducted due to the small scale/pilot nature of the study.

*Response:*

*Thank you for this comment. We fully agree and have now removed all information and discussion on specific hypotheses and revised the information in the introduction as follows: ‘The aims of this study were to investigate the preliminary effects of activity-oriented physiotherapy combined with and without an eye movement training on dynamic balance, functional mobility, and eye movements in patients with PD. Further aims were to evaluate the feasibility of a full-scale randomised controlled trial (RCT) using pre-defined feasibility criteria of recruitment, retention and adherence rates, adverse events, and the acceptability of the intervention.’*

You also state that you did not conduct hypothesis testing to assess treatment effects within or between groups (line 261), but in your results you do make reference to within group and between-group analyses (e.g. line 374 - ).

The numbers in this study are low and therefore these results should be interpreted with caution.

*Response*

*We greatly appreciate this feedback. Reviewer #2 also expressed similar concerns. In response, we have revised our description throughout the manuscript to reflect the fact that we did not conduct hypothesis testing. Furthermore, recognising the limited number of participants in this study, we have adjusted our description and interpretation of the results accordingly.*

4. An additional paragraph in the background might be useful to highlight the links between attention and saccade frequency/visual function/gait impairment in PD as this is emerging as a significant contributer to falls and falls risk.

*Response:*

*Thank you for bringing these pertinent studies to our attention. We have now integrated the following paragraph into the introduction section: ‘Research has shown that people with PD have lower saccade frequency compared to controls [5, 6]. During turns, saccade frequency increases in both groups, while it decreases under dual-task conditions [5]. In PD, attention deficits directly relate to reduced saccade frequency, impaired visual function, and gait impairment, with significant implications for falls [5]. Although saccade frequency does not directly impact gait impairment in PD, it is indirectly influenced by visual function deficits, which affect gait through fluctuating attention [5, 6]. This highlights the importance of visual observation in maintaining safe gait and suggests a need for increased awareness of environmental scanning frequency for overall safety [5].’*

5. Line 105 – “We intended to recruit 17 patients per group and due to the pilot character of the study, no sample size calculation was performed” – acknowledging that no sample size calculation was performed, what was your initial sample size of 17 per group based on? ie. patient flow/previous studies etc

*Response:*

*We appreciate your inquiry for clarification. We have now included a subheading titled "Sample size" under the section "Participants and randomisation". The following information has been incorporated:*

*‘Given the pilot nature of this study, a formal sample size calculation was not performed. Instead, we relied on guidance from previous research. Julious recommended a minimum of 12 participants per group for pilot studies [7] whereas Browne suggested 30 participants for a two-arm study [8]. Considering potential attrition, our goal was to recruit 17 patients per group.’*

6. Line 147 – It would be useful here to clarify for the reader why your patients were in hospital receiving inpatient rehabilitation in the first place. Was this a specialist PD rehab ward? What were the indications for their admission?

*Response:*

*Thank you for posing this specific question. Yes, patients received inpatient rehabilitation in a specialist PD rehab ward within the Department of Neurology at the rehabilitation clinic. Their indication for their admission at the clinic was PD. In response to your question, in the ‘study design and setting’ section, we added the Department of Neurology to the institution’s name (Clinic for Rehabilitation Münster, Austria). In addition, at the outset of the intervention section, we have amended our description as follows: ‘Participants, admitted to the clinic primarily for PD, received the study intervention alongside inpatient rehabilitation at a specialist PD rehabilitation ward. This comprehensive programme included various therapeutic modalities such as medical training therapy, balance and gait training, strength and endurance exercises, occupational therapy, speech and language therapy, dietary guidance, psychological counselling, and patient education.’*

7. Line 150 – “… the inpatient rehabilitation program ranged from 2,240 to 2,600 minutes” – unclear what these numbers refer to. Your intervention groups received 16 x 30 minutes = 8 hours of intervention. This is probably sufficient information for the reader.

*Response:*

*Thank you for your suggestion. Based on your advice, we have eliminated the statement ‘the inpatient rehabilitation program ranged from 2,240 to 2,600 minutes’.*

8. Line 221 – A reference to justify your list of feasibility criteria would be useful here, particularly as you state that your recruitment rate did not meet the target (line 410). How did you arrive at your target rate?

*Response:*

*Thank you for your comment. We have added a reference [9]. Regarding the target recruitment rate, this had already been described: ‘a target recruitment rate of 40% of 85 eligible patients (or 4-5 participants per month); the total number of eligible PD patients was estimated based on the number patients with PD treated at the study centre in the last 3 years.’ We acknowledge however that the number of 85 eligible patients likely was inflated, due to the fact that more patients with PD were admitted to the clinic in the years 2018 and 2019. To account for this, we have incorporated the following details into the feasibility results section: ‘The recruitment delay was attributed to COVID-19 restrictions at the rehabilitation centre during the trial and potential overestimation of patient admission rates from 2018-2020.’*

9. Table 1 – please check your female:male ratio in column two (AOPT group) – should this be a ratio of 3:9?

*Response:*

*Thank you for bringing this to our attention. We have reviewed our data, and you are correct! We apologise for the error and have now rectified Table 1 accordingly.*

10. You state that both groups received identical physiotherapy interventions (line 162), and refer to eye movement training being conducted in a darkened room from a static seated position (line 174). Did both the groups undertake the “static and dynamic balance and walking exercises, hand-eye coordination activities including ball tossing and catching” or was this only part of the eye movement training?

Although you have included the TIDER summary in supplementary material, it might be useful to clarify exactly what the eye movement training consisted of in the main body of the manuscript.

*Response:*

*Thank you for seeking clarification. Reviewer #2 also raised a similar point. Indeed, both groups participated in the static and dynamic balance and walking exercises, hand-eye coordination activities including ball tossing and catching, and they exercised in the same room under identical lighting conditions. We have now updated the TIDieR checklist to reflect these corrections.*

*Regarding the eye movement training, we have already included a comprehensive description in the main body of the manuscript. If there is any specific detail you feel is missing, please let us know, and we will gladly provide the information. The text in the intervention section reads: ‘In the AOPT-E group, activity-oriented physiotherapy was combined with eye movement training. The focus of the eye movement training was on the saccade size and number of fixations and (increasing) the visual field. For the design of the eye movement training, the parameters of exercise intensity, duration, performance during the ON-phase, and distance of the participants from the exercise poster were derived from previous studies [10, 11] and adapted to the purpose of the present study. For eye movement training, a rectangular exercise poster was placed on the wall at a height of 0.9 metre and a 0.7 metre distance from the participant. The participant was instructed to keep both feet on the floor, sit as upright as possible and move the head as little as possible during the poster eye movement training (the dimensions and a photo of the poster are shown in S2 File, S2.1 Fig).* *The training commenced with an eye warm-up to minimise the risk of injury. Eye movement training involved performing visual search tasks from the poster centre outwards either in a fixed or random order. We aimed to alternate saccades and fixations i.e., during the training patients were instructed to direct their gaze towards specific colour dots embedded in the poster, which also included several differently coloured distractor dots. Each eye movement began with a black coloured central fixation dot. Of the 16 dots, 4 were in the corners of the poster and 3 each were aligned on a vertical or horizontal axis from the central fixation point to the top, bottom, left, and right. Additionally, walking training was performed on a hallway with wall markings; using a crosshair through adhesive tape and adhesive dots on the wall (S2 File, S2.2 Fig) participants were asked to perform various* *balance, coordination, and functional mobility exercises, combined with visual fixation or search tasks utilising the wall markings.’*

11. The final paragraph of the discussion section (line 551 onwards) is more descriptive than discursive. Given your findings, this section could be strengthened, for example, with reference to the potential impact of eye movement training on FOG, acknowledging existing literature in this field.

*Response:*

*Thank you for your valuable suggestion. We have implemented your feedback by removing the descriptive segment from the discussion and have included the following paragraph. This new addition discusses the potential influence of eye movement training on FOG, recognising and integrating existing literature on this topic:*

*‘**Our eye tracking results indicate that eye movement training shows promise in addressing FOG in people with PD. For instance, in FGA item 6, the AOPT-E group exhibited a remarkable shift in focus, reducing fixations on the ground before stepping over an obstacle and instead directing attention towards the obstacle itself and the surrounding terrain. This shift indicates improved balance and expanded visual awareness during walking, echoing recent findings that people with PD and FOG tend to concentrate more on immediate steps [12]. Furthermore, our study surpasses prior research revealing that people with PD exhibit fewer relevant fixations during regular walking but increase their focus on task-relevant elements during obstacle negotiation [13]. Cueing, known to aid walking in PD, especially for those with FOG [14] is supported by our results suggesting that eye movement training improves walking by increasing task-relevant fixation. Recent research underscores the profound impact of FOG on visual attention, with PD patients experiencing FOG showing prolonged fixation durations and a tendency to focus on nearby ground areas rather than their intended path, contrasting with the fixation patterns of those without FOG and healthy controls [15]. Our findings provide evidence for the potential benefits of AOPT-E for people with PD, particularly those with FOG. In FGA item No. 5, our analysis revealed a significant reduction in maximum fixation duration towards the ground post-intervention for the AOPT-E group, while the AOPT group exhibited the opposite trend. This suggests the potential efficacy of AOPT-E in modifying visual attention patterns, offering a promising avenue for enhancing mobility and potentially reducing FOG in people with PD.’*

12. There are a lot of qualitative data relating to the interview responses in the Supplementary files – while I acknowledge these data are not the focal point of this study, the “five themes” (line 540) could be more clearly presented (i.e. as it reads presently, it is hard to determine what the five themes actually are ? training organisation; variety and challenge; training intensity; rest; targeted patient education; performance feedback; walking safety; strategies for optimal training; therapeutic relationship; pleasure and meaning).

(While the S1 file is comprehensive, the data are also a little confusing in that the themes read more like statements or key findings).

*Response:*

*We appreciate your feedback. In response, we have incorporated the information provided below at the conclusion of the results section. If you feel that this is insufficient, we are happy to add more details.*

*Five themes were identified through the thematic analysis. (1) Participants appreciate the study's organisation, varied content, and appropriate challenge level, expressing a need for breaks. (2) Participants find the training intense and suggest that future sessions could benefit from a more balanced approach, potentially incorporating rest days. (3) Participants prefer targeted patient education, a home exercise programme, and progress feedback from therapists. (4) Main goals include increasing walking safety, coping strategies for the disease, active training opportunities, preferred training times, and a positive patient-therapist relationship. (5) Participants enjoy the intervention, reporting subjective improvements in daily activities, and find it meaningful (see S1 File for detailed findings). These themes underscore the feasibility of an eye movement training for people with PD.*

*In the discussion, the text is now formulated as follows: ‘Furthermore, regarding the acceptability of the AOPT-E intervention, our thematic analysis of interview data confirmed a high level of acceptability. Participants expressed satisfaction with the study's well-structured and diversified approach, offering an appropriate level of challenge. Participants perceived the training as intense, suggest that future sessions could benefit from a more balanced approach, potentially incorporating rest days. Participants prioritised targeted patient education, a home exercise programme, and feedback from therapists. Goals centred around enhancing walking safety, developing coping strategies, offering active training options, scheduling preferred training times, and fostering a positive patient-therapist relationship. They reported finding the intervention enjoyable and meaningful, noting enhancements in activities of daily living. Overall, the feedback suggests that eye movement training holds promise as a feasible and potentially beneficial intervention for people with PD, warranting further exploration.’*

**References**

1.	Wang X, Piantadosi S, Le-Rademacher J, Mandrekar SJ. Statistical Considerations for Subgroup Analyses. Journal of Thoracic Oncology. 2021;16(3):375-80. doi: <https://doi.org/10.1016/j.jtho.2020.12.008>.

2.	Sim J. Should treatment effects be estimated in pilot and feasibility studies? Pilot Feasibility Stud. 2019;5:107. Epub 20190828. doi: 10.1186/s40814-019-0493-7. PubMed PMID: 31485336; PubMed Central PMCID: PMCPMC6712606.

3.	Capecci M, Pournajaf S, Galafate D, Sale P, Le Pera D, Goffredo M, et al. Clinical effects of robot-assisted gait training and treadmill training for Parkinson's disease. A randomized controlled trial. Ann Phys Rehabil Med. 2019;62(5):303-12. Epub 2019/08/05. doi: 10.1016/j.rehab.2019.06.016. PubMed PMID: 31377382.

4.	Shin JC, Kim JH, Nam D, Park GC, Lee JS. Add-on effect of kinesiotape in patients with acute lateral ankle sprain: a randomized controlled trial. Trials. 2020;21(1):176. Epub 2020/02/14. doi: 10.1186/s13063-020-4111-z. PubMed PMID: 32051009; PubMed Central PMCID: PMCPMC7017523.

5.	Stuart S, Galna B, Delicato LS, Lord S, Rochester L. Direct and indirect effects of attention and visual function on gait impairment in Parkinson's disease: influence of task and turning. The European journal of neuroscience. 2017;46(1):1703-16. Epub 2017/04/27. doi: 10.1111/ejn.13589. PubMed PMID: 28444834.

6.	Stuart S, Lord S, Galna B, Rochester L. Saccade frequency response to visual cues during gait in Parkinson's disease: the selective role of attention. The European journal of neuroscience. 2018;47(7):769-78. Epub 2018/02/13. doi: 10.1111/ejn.13864. PubMed PMID: 29431890.

7.	Julious SA. Sample size of 12 per group rule of thumb for a pilot study. Pharmaceutical statistics. 2005;4:287-91. doi: 10.1002/pst.185.

8.	Browne RH. On the use of a pilot sample for sample size determination. Stat Med. 1995;14(17):1933-40. PubMed PMID: 8532986.

9.	El-Kotob R, Giangregorio LM. Pilot and feasibility studies in exercise, physical activity, or rehabilitation research. Pilot Feasibility Stud. 2018;4:137. Epub 2018/08/21. doi: 10.1186/s40814-018-0326-0. PubMed PMID: 30123527; PubMed Central PMCID: PMCPMC6090705.

10.	Camacho PB, Carbonari R, Shen S, Zadikoff C, Kramer AF, López-Ortiz C. Voluntary Saccade Training Protocol in Persons With Parkinson’s Disease and Healthy Adults. Frontiers in Aging Neuroscience. 2019;11. doi: 10.3389/fnagi.2019.00077.

11.	Hebert JR, Corboy JR, Vollmer T, Forster JE, Schenkman M. Efficacy of Balance and Eye-Movement Exercises for Persons With Multiple Sclerosis (BEEMS). Neurology. 2018;90(9):e797-e807. Epub 2018/02/02. doi: 10.1212/wnl.0000000000005013. PubMed PMID: 29386274.

12.	Hardeman LES, Kal EC, Young WR, van der Kamp J, Ellmers TJ. Visuomotor control of walking in Parkinson's disease: Exploring possible links between conscious movement processing and freezing of gait. Behav Brain Res. 2020;395:112837. Epub 2020/08/03. doi: 10.1016/j.bbr.2020.112837. PubMed PMID: 32739286.

13.	Hunt D, Stuart S, Nell J, Hausdorff JM, Galna B, Rochester L, et al. Do people with Parkinson's disease look at task relevant stimuli when walking? An exploration of eye movements. Behav Brain Res. 2018;348:82-9. Epub 2018/03/22. doi: 10.1016/j.bbr.2018.03.003. PubMed PMID: 29559336.

14.	Cosentino C, Putzolu M, Mezzarobba S, Cecchella M, Innocenti T, Bonassi G, et al. One cue does not fit all: A systematic review with meta-analysis of the effectiveness of cueing on freezing of gait in Parkinson's disease. Neuroscience and biobehavioral reviews. 2023;150:105189. Epub 2023/04/23. doi: 10.1016/j.neubiorev.2023.105189. PubMed PMID: 37086934.

15.	Vanegas-Arroyave N, Chen DF, Lauro PM, Norato G, Lungu C, Hallett M. Where Do Parkinson's Disease Patients Look While Walking? Mov Disord. 2022;37(4):864-9. Epub 2022/01/09. doi: 10.1002/mds.28917. PubMed PMID: 34997620; PubMed Central PMCID: PMCPMC9018475.
