# Supplementary material for: Effects of activity-oriented physiotherapy with and without eye movement training on dynamic balance, functional mobility, and eye movements in patients with Parkinson’s disease: An assessor-blinded randomised controlled pilot trial
Source: PLoS One. 2024 Jun 14;19(6):e0304788. doi: 10.1371/journal.pone.0304788 (PMC11178185; doi:10.1371/journal.pone.0304788)
Supplement: Attachment — Submitted filename: Response to Reviewers.docx [file pone.0304788.s011.docx]

**PONE-D-23-31439R1**

Effects of activity-oriented physiotherapy with and without eye movement training on dynamic balance, functional mobility, and eye movements in patients with Parkinson’s disease: an assessor-blinded randomised controlled pilot trial

Dear Dr. Seebacher,

Thank you for submitting your manuscript to PLOS ONE. After careful consideration, we feel that it has merit but does not fully meet PLOS ONE’s publication criteria as it currently stands. Therefore, we invite you to submit a revised version of the manuscript that addresses the points raised during the review process.

Overall the reviewers were very positive concerning this revision. There is only 1 minor revision requested at this time and I believe that if this is addressed within the methods paragraph on statistics that will be sufficient. Please also provide a citation justifying the effect size breakdown. "The only minor comment I have is in relation to your use of effect sizes in the revised manuscript- I wonder if it might be useful to add a little clarity in your abstract/statistics paragraph to help the reader interpret what your r values mean e.g. small/medium/large effect sizes."

On receipt of this minor revision, your manuscript will be acceptable for publication at PLOS.

*Dear Editor,*

*Dear Reviewers,*

*We thank the Editor and Reviewer #3 for their valuable minor comment and feedback and for giving us a chance to submit a revised manuscript. Please find below our point-by-point responses. The main manuscript has been amended accordingly, with any changes highlighted to allow the Editor and Reviewers to check the adaptations made.*

*We thank the Editor and Reviewers for their time and effort and hope we have sufficiently responded to the Editor’s and Reviewers’ requests.*

*With kind regards,*

*Barbara Seebacher, on behalf of all the authors*

We look forward to receiving your revised manuscript.

Kind regards,

Eric R. Anson

Academic Editor

PLOS ONE

**Review Comments to the Author**

**Reviewer #1:** (No Response)

*Dear Reviewer #1, Thank you for your previous valuable suggestions and for acknowledging the changes we made.*

**Reviewer #2:** Thank you for the clear and transparent responses to the comments and for the adaptations to the manuscript. This work now seems fit for publication. Congratulations, and all the best!

*Dear Reviewer #2, Thank you for your previous valuable suggestions, for acknowledging the changes we made, and for your good wishes.*

**Reviewer #3:** Thank you for your detailed response to the feedback from reviewers and providing clarity on the comments I raised. I have enjoyed reading your paper in its revised format and I think the discussion is much stronger now. I wish you all the best for your future research in this exciting area.

*Dear Reviewer #3, Thank you for your previous valuable suggestions, for acknowledging the changes we made, and best wishes.*

Point 1: The only minor comment I have is in relation to your use of effect sizes in the revised manuscript- I wonder if it might be useful to add a little clarity in your abstract/statistics paragraph to help the reader interpret what your r values mean e.g. small/medium/large effect sizes.

*Response 1: Thank you for your advice. We have now included the following sentence in the abstract: ‘Effect sizes of 0.10 were considered weak, 0.30 moderate, and ≥0.50 strong.’ In the statistical analysis section, we have included the following statement: ‘According to Cohen (1988), a correlation coefficient of 0.10 is considered weak, 0.30 is considered moderate, and 0.50 or higher is considered strong. [1].’*

**Reference**

1.	Cohen J. Statistical power analysis for the behavioral sciences. 2^nd^ ed ed. Hillsdale: Lawrence Erlbaum Associates; 1988.
